# The Induction of the Isoflavone Biosynthesis Pathway Is Associated with Resistance to Common Bacterial Blight in *Phaseolus vulgaris* L.

**DOI:** 10.3390/metabo11070433

**Published:** 2021-07-01

**Authors:** Laura D. Cox, Seth Munholland, Lili Mats, Honghui Zhu, William L. Crosby, Lewis Lukens, Karl Peter Pauls, Gale G. Bozzo

**Affiliations:** 1 Department of Plant Agriculture, University of Guelph, 50 Stone Road East, Guelph, ON N1G 2W1, Canada; lcox05@uoguelph.ca (L.D.C.); llukens@uoguelph.ca (L.L.); ppauls@uoguelph.ca (K.P.P.); 2 Department of Biological Sciences, University of Windsor, 401 Sunset Ave, Windsor, ON N9B 3P4, Canada; munholl@uwindsor.ca (S.M.); bcrosby@uwindsor.ca (W.L.C.); 3 Guelph Research and Development Centre, Agriculture and Agri-Food Canada, 93 Stone Road West, Guelph, ON N1G 5C9, Canada; lili.mats@canada.ca (L.M.); honghui.zhu@canada.ca (H.Z.)

**Keywords:** common bacterial blight, disease resistance, flavonoid, isoflavone, *Phaseolus vulgaris*, pathogen defense, *Xanthomonas axonopodis*

## Abstract

*Xanthomonas axonopodis* infects common bean (*Phaseolus vulgaris* L.) causing the disease common bacterial blight (CBB). The aim of this study was to investigate the molecular and metabolic mechanisms underlying CBB resistance in *P. vulgaris*. Trifoliate leaves of plants of a CBB-resistant *P. vulgaris* recombinant inbred line (RIL) and a CBB-susceptible RIL were inoculated with *X. axonopodis* or water (mock treatment). Leaves sampled at defined intervals over a 48-h post-inoculation (PI) period were monitored for alterations in global transcript profiles. A total of 800 genes were differentially expressed between pathogen and mock treatments across both RILs; approximately half were differentially expressed in the CBB-resistant RIL at 48 h PI. Notably, there was a 4- to 32-fold increased transcript abundance for isoflavone biosynthesis genes, including several isoflavone synthases, isoflavone 2′-hydroxylases and isoflavone reductases. Ultra-high performance liquid chromatography-tandem mass spectrometry assessed leaf metabolite levels as a function of the PI period. The concentrations of the isoflavones daidzein and genistein and related metabolites coumestrol and phaseollinisoflavan were increased in CBB-resistant RIL plant leaves after exposure to the pathogen. Isoflavone pathway transcripts and metabolite profiles were unaffected in the CBB-susceptible RIL. Thus, induction of the isoflavone pathway is associated with CBB-resistance in *P. vulgaris*.

## 1. Introduction

Common bean (*Phaseolus vulgaris* L.) is one of the most highly cultivated edible legumes, with global production in 2019 at 28.9 million tonnes [1]. Most bean producing regions are quite susceptible to common bacterial blight (CBB), a disease caused by the bacterium *Xanthomonas axonopodis* and its *fuscans* relative [2]. CBB symptoms initiate as water-soaked lesions of the foliage and seed pods [3]; and references therein], and the disease culminates in a reduction in seed yield in the range of 35% to 75% of total field production [4,5,6]. Infected seed material can result in the reoccurrence of the disease in field operations across several growing seasons. Most *P. vulgaris* cultivars are susceptible to CBB [7]. By contrast, the white bean cultivar ‘OAC Rex’ is resistant to CBB, as it carries resistance loci from a member of its pedigree that was derived from an interspecific cross with *Phaseolus acutifolius* A. Gray [8,9,10]. Alleles linked to/associated with CBB resistance are highly conserved between the ‘OAC Rex’ genome and that of the Andean *P. vulgaris* line G18933 [11]. Molecular markers for CBB resistance have been identified on several chromosomes, including *Pv*-CTT001 on chromosome 4, SU91 on chromosome 8, and SAP6 on chromosome 10 [11]. A region within the ‘OAC Rex’ genome associated with CBB-resistance loci includes a gene with homology to a chalcone reductase (CHR) from soybean (*Glycine max* [L]. Merr.) [11]. CHR is an early step in the isoflavone biosynthesis pathway whereby it converts naringenin chalcone to isoliquiritigenin, a precursor of isoflavones and their derived phytoalexins [12].

**Figure 1:**
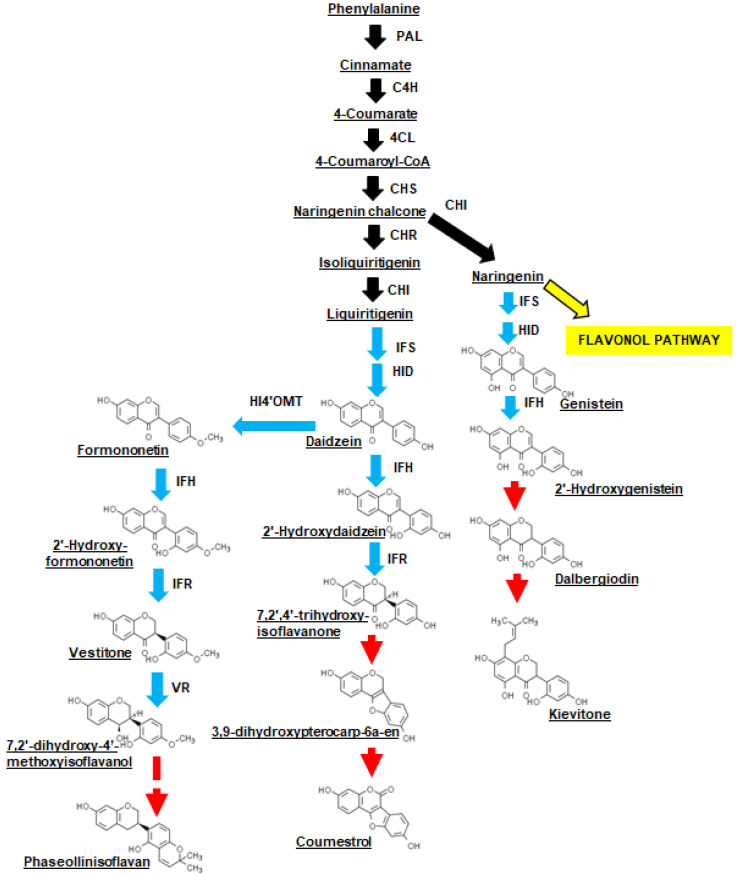
Proposed isoflavone biosynthesis and conversion in *P. vulgaris* leaves in response to *X. axonopodis*. The scheme was adapted from isoflavone biosynthetic pathways described for various legumes [23,26] and metabolites that are known to occur in *P. vulgaris*, specifically coumestrol, daidzein, dalbergiodin, genistein, 2′-hydroxygenistein, kievitone and phaseollinisoflavan [24]. General flavonoid pathway reactions are represented by black solid arrows; a yellow arrow represents the divergence to the flavonol pathway. Isoflavone pathway reactions are represented by blue arrows. Red arrows are used to denote proposed and/or unknown reactions leading to the accumulation of hydroxyisoflavanones (e.g., kievitone), coumestans (i.e., coumestrol) and isoflavans (i.e., phaseollinisoflavan). Abbreviations include: CHI, chalcone isomerase; CHR, chalone reductase; CHS, chalcone synthase; C4H, cinnamate 4-hydroxylase; 4CL, 4-coumaroyl: coenzyme A ligase; HID, 2-hydroxyisoflavanone dehydratase; HI4′OMT, 2-hydroxyisoflavanone 4′-*O*-methyltransferase; IFH, isoflavone 2′-hydroxylase; IFR, isoflavone reductase; IFS, isoflavone synthase; PAL, phenylalanine ammonia-lyase; VR, vestitone reductase.

In most legume crops, resistance to fungal-related diseases is associated with the accumulation of isoflavones in plant tissues challenged with the pathogen [13,14,15,16,17]. The levels of isoflavones, such as genistein and its methylated derivative prunetin, are elevated in *P. vulgaris* roots within 24 h of exposure to the fungus that causes *Fusarium* wilt [18]. Isoflavones are C6-C3-C6 derivatives of the phenylpropanoid pathway that are prevalent within legumes. Isoflavone biosynthesis has been well characterized in soybean [19] but little explored in *P. vulgaris*. The key step in the biosynthesis of isoflavones and related isoflavonoids involves aryl migration and hydroxylation of flavanones, in a reaction catalyzed by isoflavone synthase (IFS, also known as 2-hydroxyisoflavanone synthase), although the latter step could require a second enzyme, 2-hydroxyisoflavanone dehydratase (Figure 1) [19,20]. IFS converts the respective flavanones naringenin and liquiritigenin to genistein and daidzein [20,21]. In various legumes, these isoflavones can be converted to pterocarpans (e.g., medicarpin) through a process that requires the sequential action of isoflavone 2’-hydroxylase (IFH), a NADPH-dependent isoflavone reductase (IFR) [19,22], a vestitone reductase (VR) and finally a pterocarpan synthase [23]. 

Isoflavones and derived compounds that are known to accumulate in *P. vulgaris* include daidzein and genistein, as well as hydroxyisoflavanones (e.g., dalbergiodin and kievitone), related compounds such as the coumestan coumestrol, the isoflavan phaseollinisoflavan and pterocarpans (e.g., phaseollin) (Figure 1) [24]. In fact, several isoflavones, coumestrol, and phaseollinisoflavan accumulate in seedlings of a related species *Phaseolus coccineus* L. treated with the fungus *Rhizopus oryzae* [25]. Coumestrol is proposed to be derived from daidzein by way of the oxidation products of pterocarpans [26]. A soybean VR converts the IFR product 2′-hydroxyisoflavanone to a 3,9-dihydroxypterocarpan [27], with the latter compound proposed as an intermediate for coumestrol biosynthesis by a hitherto unknown enzyme [26]. By comparison, an alfalfa (*Medicago sativa* L.) VR converts vestitone to 7,2′-dihydroxy-4′-isoflavanol, which is subsequently dehydrated to medicarpin (Figure 1) [28]. In addition to isoflavones, kaempferol and quercetin glycosides also occur in vegetative tissues including leaves of Andean and Mesoamerican *P. vulgaris* plants [29]. To date, it is unknown whether flavonol glycoside and/or isoflavone metabolism is altered in *P. vulgaris* plants challenged by *X. axonopodis*. 

The objective of our study was to identify metabolic and molecular mechanisms involved in *P. vulgaris* resistance to CBB caused by *X. axonopodis*. Specifically, the objective was to test the hypothesis that isoflavone biosynthesis is linked with CBB-resistance in *P. vulgaris*. For these investigations, RNA sequencing (RNA-seq) was used to monitor transcriptome alterations in the first trifoliate leaves of white bean plants, specifically a CBB-resistant recombinant inbred line (RIL) and a CBB-susceptible RIL following *X. axonopodis* infiltration relative to plants inoculated with sterile water (mock treatment). Bioinformatic analysis was used to identify genes that were differentially expressed in both RILs within the 48-h period post-inoculation (PI). In addition, temporal shifts in metabolite profiles in *X. axonopodis* and mock-inoculated leaves of the white bean plants were determined with a specific emphasis on isoflavone profiles.

## 2. Results

### 2.1. CBB Symptoms Were Evident in Leaves of the CBB-Susceptible P. vulgaris but Not CBB-Resistant Plants

**Figure 2:**
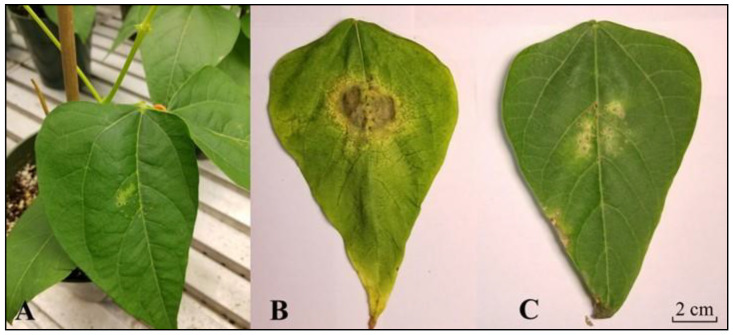
CBB disease development on first trifoliate leaves of CBB-susceptible and CBB-resistant *P. vulgaris* RILs. (**A**) On the third d after *X. axonopodis* (*Xap*) inoculation, symptoms were evident as lighter green patches within the inoculation area of CBB-susceptible RIL leaflets. (**B**) At the end of the disease rating period disease symptoms developed into lesions with necrotic tissue in the center of the CBB-susceptible RIL leaflet. (**C**) For the CBB-resistant RIL leaf treated with *Xap*, smaller lesions were apparent in the inoculated leaflet by the end of the disease rating period. Photos B and C are representative images of final disease ratings taken for plants from experiment #1 of three separate disease inoculation experiments. These images were taken on d 11 post-inoculation with *Xap*, and the accumulation of necrotic symptoms was more advanced than for the other two experiments which were assessed on d 14.

**Figure 3:**
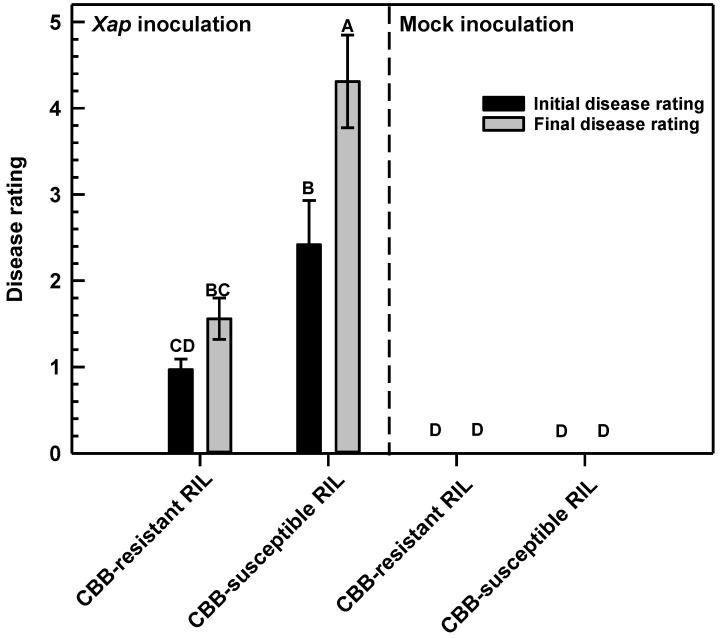
Disease ratings of *P. vulgaris* RILs following inoculation with *X. axonopodis*. For both RILs, the first trifoliate leaf of each plant was inoculated with *X. axonopodis* (*Xap*) or sterile water (mock). Disease ratings were scored on a 0 to 5 subjective scale as described under Materials and Methods Section 4.2. For each RIL, its disease treatment and PI evaluation period, data represent the mean ± standard error of three experimental replicates. Disease rating data sharing the same letter are not statistically different at a *p*-value ≤ 0.05. Initial disease ratings are represented by the black bars, which is calculated from data collected on d 8 PI for disease inoculation experiment #1, and d 7 PI for disease inoculation experiments #2 and #3. Final disease ratings are represented by the grey bars and were assessed on d 11 PI for the experiment #1, and on d 14 PI for the other two disease inoculation experiments.

In this study, we evaluated CBB disease symptoms in a CBB-resistant RIL and a CBB-susceptible RIL, both of which were previously derived from a cross between the CBB-resistant white bean cultivar ‘OAC Rex’ and the CBB-susceptible cultivar ‘OAC Seaforth’ [8]. Visual symptoms of CBB were first apparent 3 d after *X. axonopodis* inoculation of the first trifoliate leaf of a CBB-susceptible RIL. These initial CBB symptoms were evident as pale green patches within the inoculation area of *X. axonopodis*-treated leaflets (Figure 2A). Necrosis and tissue yellowing were apparent on pathogen-treated leaves of the CBB-susceptible RIL within 11 to 14 d PI (Figure 2B), whereas these symptoms were less evident in the CBB-resistant RIL (Figure 2C). This corresponded to final disease ratings between 4 and 5, which are respectively equivalent to 51% to 80% and 80% or more of the leaf showing CBB symptoms in the susceptible RIL, whereas average disease ratings were lower than a score of 2 (i.e., 11–30% of the leaf displaying symptoms) in the CBB-resistant RIL (Figure 3). No symptoms were apparent in mock-inoculated leaves of both RILs.

### 2.2. P. vulgaris RIL Leaf Transcriptomes Changed Following Treatment with X. axonopodis

RNA-seq was performed to assess alterations in leaf transcriptomes in both RILs as a function of the time period following inoculation with *X. axonopodis*. Forty-eight cDNA libraries were generated from mRNA-enriched samples prepared from *P. vulgaris* first trifoliate leaf tissue, representing three separate disease inoculation experiments. Each experiment comprised both RILs, two inoculation treatments (*X. axonopodis* and mock) and four sampling periods, specifically at 0, 8, 24 and 48 h PI. The number of raw sequence reads that were generated ranged from 84.6 to 99.9 million reads across the 48 cDNA libraries (Supplementary Information, Table S1). Of the trimmed paired reads, an average of 79% mapped to the *P. vulgaris* G19833 reference genome with a mean of 9% mapping to more than one location within the reference genome. Cufflinks was used to assemble aligned sequence reads into transcripts, which yielded an average of 43,196 gene transcripts mapped per library across all three experiments, RILs, inoculum treatments and PI sampling times. This transcript information, recorded as fragments per kilobase of exon per million fragments mapped (FPKM), was analyzed to determine the number of differentially expressed genes (DEGs) across both RILs as a function of the PI time period.

### 2.3. Differental Gene Expression Was Most Evident in the CBB-Resistant RIL at 48 h Following Inoculation with X. axonopodis

**Figure 4:**
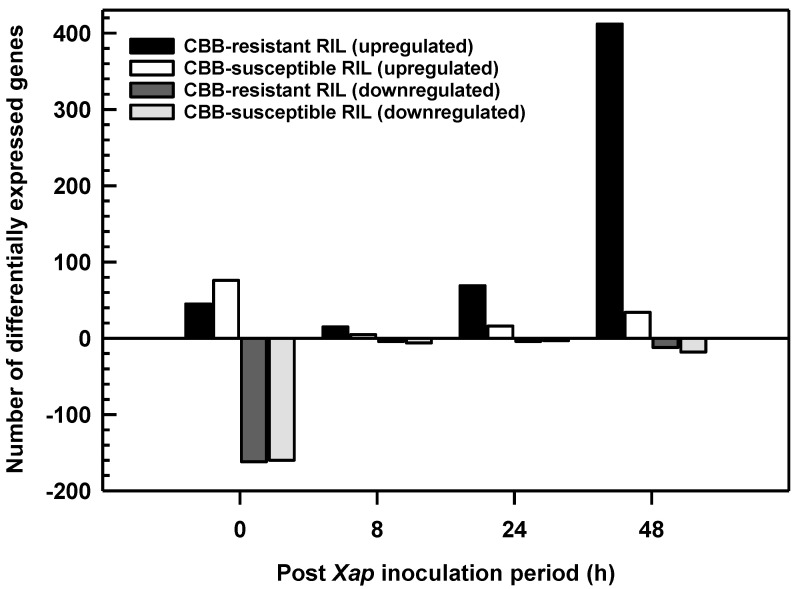
Upregulated and downregulated differentially expressed genes (DEGs) for the *P. vulgaris* CBB-resistant RIL and the CBB-susceptible RIL as a function of the period following inoculation with *X. axonopodis* (*Xap*). All DEGs were identified based on alterations in transcript abundance relative to the mock inoculation across all three disease inoculation experiments.

For each RIL, a gene was considered differentially expressed if there was a log2 fold change ≤−2 and/or ≥2 when transcript abundance levels between *X. axonopodis*-inoculated leaves were compared to those of mock-inoculated leaves at the same PI sampling time, and had an adjusted false discovery *p* value (q value) ≤0.05 (Supplementary Information, Excel Spreadsheets S1 and S2). There were 800 DEGs, including those genes having transcript alterations at multiple PI sampling periods or in both RILs (Supplementary Information, Tables S2–S51). For each RIL, these DEGs were categorized into upregulated and downregulated genes for each PI sampling time (Figure 4). Most of the transcriptome alterations in the first trifoliate leaves were apparent in the CBB-resistant RIL, comprising approximately 69% of the total cases of differential gene expression (Figure 4). For this genotype, 75% of the DEGs were elevated in response to *X. axonopodis*. In contrast, 31% of the total DEGs were in *X. axonopodis*-inoculated leaves of the CBB-susceptible RIL, with 41% of these upregulated and 59% downregulated. The greatest number of DEGs were evident in the leaves of the CBB-resistant RIL 48 h after *X. axonopodis* inoculation, with 97% of 424 genes upregulated in this germplasm. By comparison, 5% of the total DEGs were evident 48 h PI in the CBB-susceptible RIL. Large numbers of DEGs were also apparent at the time of inoculation in both RILs, where the majority were downregulated. Approximately 10% of all cases of differential expression occurred at 8 and 24 h after pathogen inoculation in the CBB-resistant RIL; few DEGs were apparent at these times in the CBB-susceptible RIL. 

**Figure 5:**
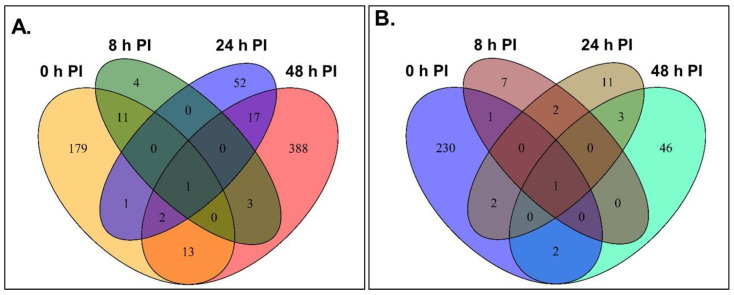
Venn diagram of *P. vulgaris* genes that were differentially expressed in the first trifoliate leaf at one or more sampling times following *X. axonopodis* inoculation in the (**A**) CBB-resistant RIL and the (**B**) CBB-susceptible RIL. In both plots, the label above each Venn diagram represents the post-inoculation (PI) period.

Dynamic changes in gene expression were evident across infection with *X. axonopodis* (Figure 5). Most of the transcriptome changes occurred in *X. axonopodis* treated CBB-resistant RIL sampled 48 h PI. In addition, most differential gene expression was evident at a single PI sampling time, although there were 5 and 24 DEGs occurring at multiple PI sampling times in the CBB-susceptible RIL and in the CBB-resistant RIL, respectively (Supplementary Information, Tables S18–S51). These DEGs included isoflavone biosynthesis genes such as IFS and IFR. In contrast, the greatest number of DEGs in the CBB-susceptible RIL were evident at 0 h PI. A total of 176 DEGs were shared between the CBB-resistant RIL and CBB-susceptible RIL, with the majority of these evidenced at 0 h PI (Supplementary Information, Tables S18, S19, S23, S24, S26–S30, S33 and S35–S47).

### 2.4. Isoflavone Biosynthesis Gene Transcripts Were More Abundant in the CBB-Resistant RIL at 48 h Following Inoculation with X. axonopodis

**Figure 6:**
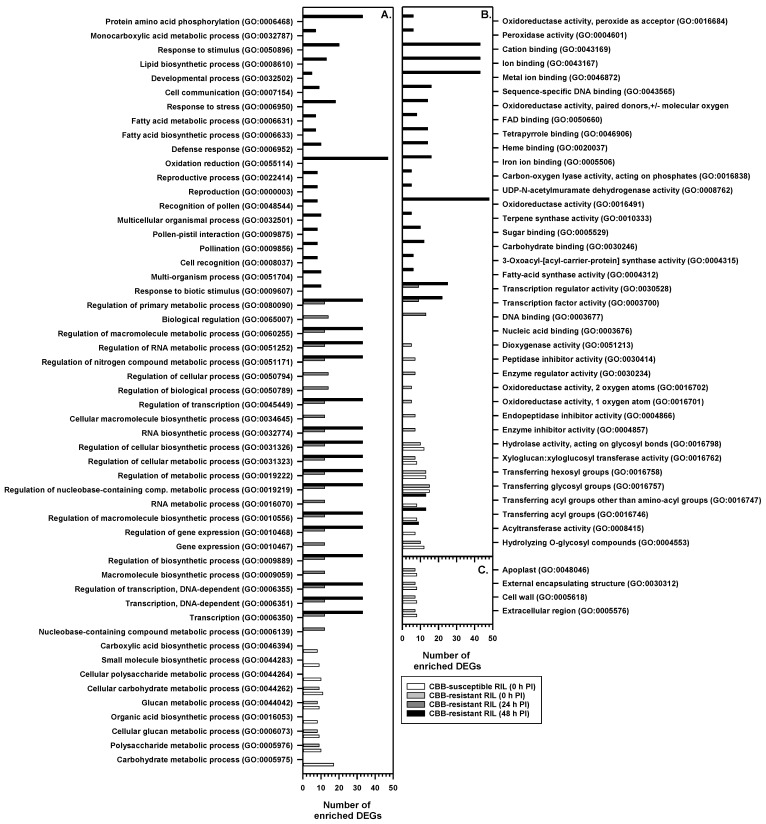
Gene ontology (GO) enrichment analysis of differentially expressed genes (DEGs) in the first trifoliate leaf of the CBB-resistant RIL and the CBB-susceptible RIL at one or more sampling periods following *X. axonopodis* inoculation. All plots represent the number of enriched DEGs in the CBB-susceptible RIL at 0 h post-inoculation (PI) (open bar), and in the CBB-resistant RIL at 0 h PI (light grey bar), 24 h PI (dark grey bar) and 48 h PI (black bar). No enriched genes were apparent in either RIL at 8 h PI, and at 24 and 48 h in the CBB-susceptible RIL. Plots (**A**–**C**) respectively represent biological process, molecular function and cellular components GO terms.

A GO enrichment analysis was performed with the online platform agriGO V2 together with GO terms and functional annotations from the *P. vulgaris* V2.1 G19833 reference genome (Figure 6; Supplementary Information, Tables S52–S55). Several GO terms were enriched in both RILs at 0 h PI with *X. axonopodis*, including xyloglucan endotransglucosylase activity, glucosyltransferase and cell wall hydrolase activity (Supplementary Information, Tables S52 and S53). There were several enriched GO terms that were only apparent in the CBB-susceptible RIL at 0 h PI, including processes related to carboxylic acid biosynthesis, organic acid biosynthesis, or metabolism of carbohydrates and polysaccharides. For example, transcripts mapping multiple CHS genes were associated with GO term GO:0004315 (3-oxoacyl-[acyl-carrier-protein] synthase activity) and enriched at 0 h PI in the CBB-susceptible RIL; these were downregulated by approximately 75% in the pathogen-treated plants relative to the mock treatment (Supplementary Information, Table S41). Similarly, various GO terms were solely enriched in the CBB-resistant RIL at 0 h PI, such as peptidase inhibitor activity, enzyme regulatory activity or oxidoreductase activity. For the latter molecular function, this included the downregulated expression of putative lineolate lipoxygenase genes, such as *Phvul.005G156800*, and a UDP-glucose 4,6-dehydratase (*Phvul.006G034000*) (Supplementary Information, Tables S10 and S53). 

In the CBB-resistant RIL, biological and molecular GO terms were enriched at 24 and 48 h PI with *X. axonopodis*; no cellular GO terms were enriched at either time point. At 24 h PI, a large number of DEGs were associated with GO terms linked to regulation of biological process and regulation of cellular process. Included were various upregulated genes that encode transcription factors, such as WRKY, those with AP2 binding domains that are involved in ethylene-mediated responses and a putative MYB (*Phvul.007G273400*) (Figure 6; Supplementary Information, Tables S4 and S54). The GO analysis revealed that 166 DEGs were associated with the enriched GO terms for the CBB-resistant RIL at 48 h PI; 29% of the DEGs were classified as either oxidation reduction (GO:0055114) or oxidoreductase activity (GO:0016491) GO terms (Supplementary Information, Table S55). Many DEGs within the oxidation and reduction category were associated with general flavonoid biosynthesis and isoflavone pathway genes.

**Figure 7:**
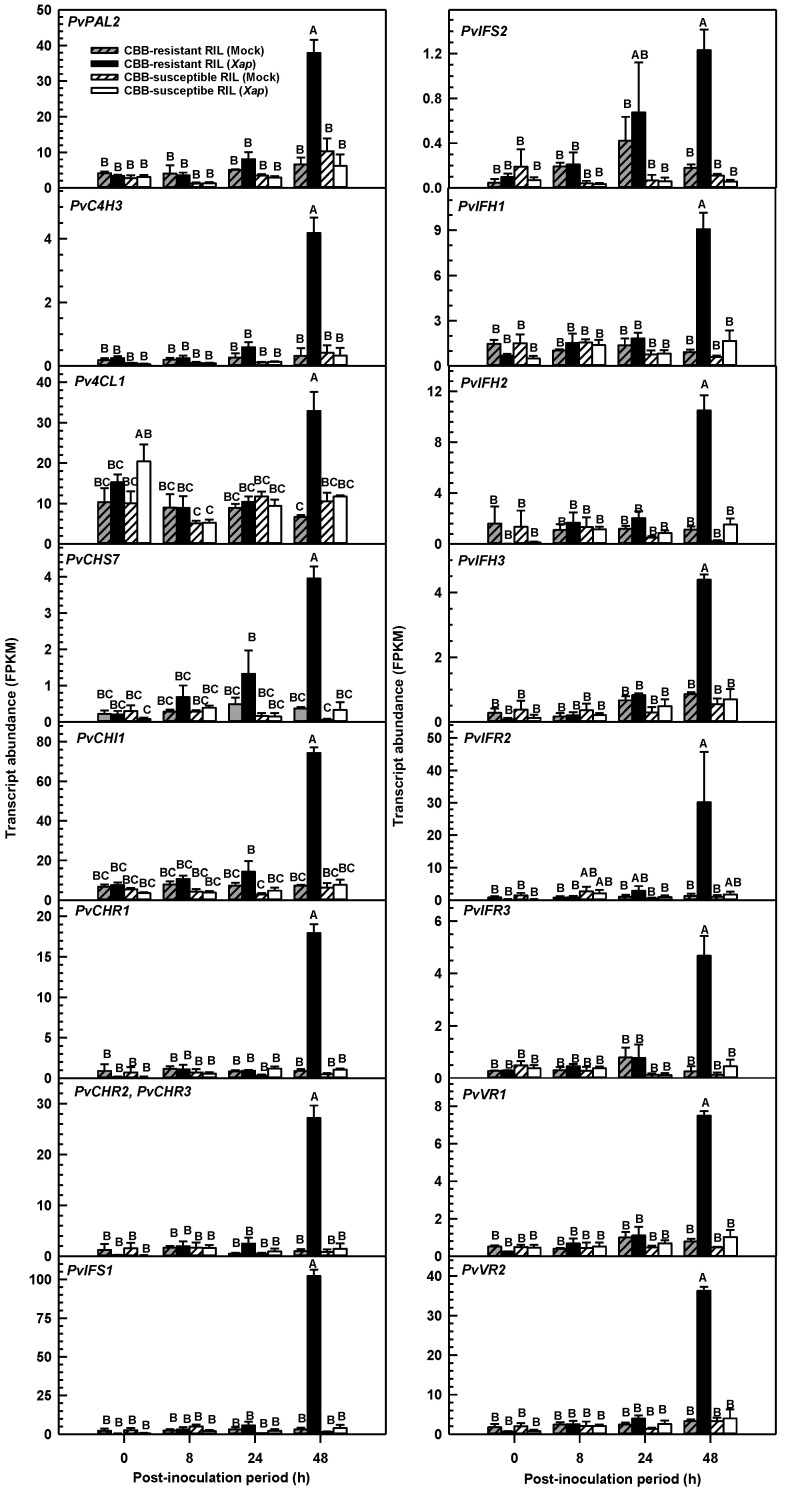
Flavonoid and isoflavone pathway gene transcript abundance in the first trifoliate leaves of CBB-resistant and CBB-susceptible *P. vulgaris* RIL plants as a function of the time period PI with *X. axonopodis* (*Xap*) relative to mock inoculation. For each gene, transcript levels are represented as FPKM. In each plot, transcript levels in mock-inoculated leaves of the CBB-resistant RIL are represented by cross-hatched grey-shaded bars; transcript levels in *Xap*-inoculated leaves of the CBB-resistant RIL are represented by black bars; transcript levels in mock-inoculated leaves of the CBB-susceptible RIL are represented by cross-hatched bars; transcript levels in *Xap*-inoculated leaves of the CBB-susceptible RIL are represented by open bars. Each datum represents the mean ± standard error of three separate experiments. Data sharing the same letter within each plot are not statistically different at a *p*-value ≤ 0.05. For each transcript, the corresponding gene accession number is provided in parentheses and is accompanied by the functional annotation as provided in the *P. vulgaris* G19833 (Version 2.1) genome (https://phytozome.jgi.doe.gov/pz/portal.html#!info?alias=Org_Pvulgaris) (last accessed on 7 June 2021), or those identified previously [30]. *PHENYLALANINE AMMONIA-LYASE 2*, *PvPAL2* (*Phvul.001G177700*); *CINNAMATE 4-HYDROXYLASE 3*, *PvC4H3* (*Phvul.006G079700*); *4 COUMAROYL: COENZYME A LIGASE 1*, *Pv4CL1* (*Phvul.002G040100*); *CHALCONE SYNTHASE 7*, *PvCHS7* (*Phvul.002G184300*); *CHALCONE ISOMERASE 1*, *PvCHI1 (Phvul.007G008600*); *CHALCONE REDUCTASE 1*, *PvCHR1* (*Phvul.008G015800*); *CHALCONE REDUCTASE 2* and *CHALCONE REDUCTASE 3, PvCHR2* and *PvCHR3* (*Phvul.008G287200*, *Phvul.008G287300*); *ISOFLAVONE SYNTHASE 1*, *PvIFS1* (*Phvul.003G051700*); *ISOFLAVONE SYNTHASE 2*, *PvIFS2* (*Phvul.003G074000*); *ISOFLAVONE 2’-HYDROXYLASE 1*, *PvIFH1* (*Phvul.002G014700*); *ISOFLAVONE 2’-HYDROXYLASE 2*, *PvIFH2* (*Phvul.009G244100*); *ISOFLAVONE 2’-HYDROXYLASE 3*, *PvIFH3* (*Phvul.009G244200*); *ISOFLAVONE REDUCTASE 2*, *PvIFR2* (*Phvul.002G032866*); *ISOFLAVONE REDUCTASE 3*, *PvIFR3* (*Phvul.011G044500*); *VESTITONE REDUCTASE 1*, *PvVR1* (*Phvul.008G076500*); *VESTITONE REDUCTASE 2*, *PvVR2* (*Phvul.008G076600*). *PvIFR2* and *PvIFR3* are annotated as different genes to *PvIFR1* (*Phvul.002G033300*) identified previously [31].

Transcripts detected for general flavonoid and isoflavone biosynthesis genes via the RNA-seq approach were evident across both RILs and their disease treatments, although the greatest abundance was apparent 48 h after *X. axonopodis* inoculation in the CBB-resistant RIL relative to all other RIL/ treatment/ PI sampling period *P. vulgaris* leaf libraries (Figure 7). The level of transcript accumulation for general flavonoid biosynthesis genes in pathogen-treated CBB-resistant RIL leaves was 570 to 3200% that of mock-inoculated plants. These genes were upregulated at 48 h PI, but not at earlier PI periods. Genes for early biosynthetic steps (e.g., *PvPAL2*) included those that were previously determined to be phylogenetically similar to known phenylpropanoid genes for other legume organisms, such as barrel clover (*Medicago truncatula* Gaertn.) and soybean [13,26]. In addition, increased transcript levels were apparent for a *C4H*, a *4CL1*, a *CHI*, two *PAL*s (i.e., *Phvul.001G177700* and *Phvul.008G289500*), several *CHS*s including a transcript that mapped to three genes (i.e., *Phvul.002G039100*, *Phvul.002G039166*, *Phvul.002G039232*), a transcript mapping to six genes (i.e., *Phvul.002G038600*, *Phvul.002G038700*, *Phvul.002G038800*, *Phvul.002G038900*, *Phvul.002G039000*, *Phvul.002G039300*), a transcript aligning to many CHR genes, and a transcript mapping to both *PvCHR2* and *PvCHR3* (Figure 7; Supplementary Information, Tables S5 and S41). There was no impact of *X. axonopodis* on the transcript abundance of the aforementioned genes in the CBB-susceptible RIL, regardless of the PI time period. 

*X. axonopodis* induced the accumulation of gene transcripts for several isoflavone biosynthesis enzymes in the CBB-resistant RIL 48 h PI, with detected levels in the range of approximately 4- to 32-fold those of mock-inoculated plants. Two putative IFS genes were induced by *X. axonopodis* in this genotype, although *PvIFS1* was the most prominent and its transcript levels in the CBB-resistant RIL at 48 h PI were 3200% greater than those detected in the mock-treated plants. *PvIFS2* transcript levels were also induced in this same tissue but were relatively less abundant than *PvIFS1* transcripts and increased 584% relative to the mock treatment. Finally, other putative isoflavone and isoflavan biosynthesis genes, including those annotated as *IFH***,** *IFR* and *VR* were upregulated in the CBB-resistant RIL following *X. axonopodis* treatment. In this genotype, *PvIHF1* (*Phvul.002G014700*), *PvIHF2* (*Phvul.009G244100*) and *PvIHF3* (*Phvul.009G244200*) transcript levels were enhanced in first trifoliate leaves at 48 h PI relative to earlier time points, and 825 to 2688% greater than the mock treatment. Of the *IFR*s upregulated by *X. axonopodis* in the CBB-resistant RIL, *PvIFR2* (*Phvul.002G032866*) transcripts were more abundant at 48 h PI than those of *PvIFR3* (*Phvul.011G044500*). Again, little change in *PvIFR* transcripts levels was evident at earlier PI periods, but these were 1680 to 2159% more abundant in pathogen-infected plants of the CBB-resistant RIL relative to the mock treatment. Similar transcript alteration phenomena were apparent for two VR genes, *PvVR1* (*Phvul.008G076500*) and *PvVR2* (*Phvul.008G076600*). Transcript levels for these genes were 858 to 986% greater in the CBB-resistant RIL at 48 h PI relative to the mock treatment. There was also a respective 403% and 1567% increase in gene transcripts for the isoflavone modifying steps, *ISOFLAVONE 7-O-GLUCOSYLTRANSFERASE* (*Phvul.004G103900*), and *ISOFLAVONE-7-O-β-GLUCOSIDE 6″-O-MALONYLTRANSFERASE* (*Phvul.008G032200*) (Supplementary Information, Tables S5 and S41). The levels of isoflavone pathway transcripts were unaffected in the CBB-susceptible RIL, regardless of the presence of the disease agent.

### 2.5. Isoflavones and Phytoalexins Accumulated in the Leaves of CBB-Resistant Plants Treated with X. axonopodis

**Figure 8:**
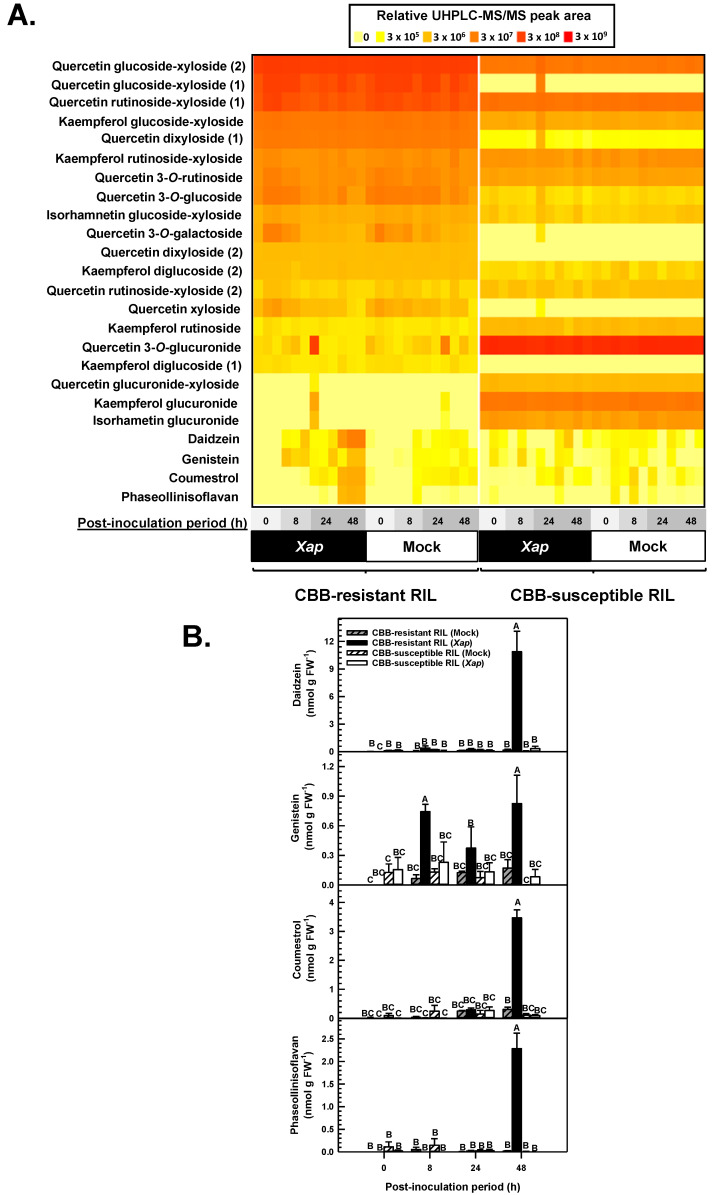
UHPLC-MS/MS analysis revealed metabolite levels were altered in the first trifoliate leaves of the CBB-resistant *P. vulgaris* RIL in response to *X. axonopodis* (*Xap*). (**A**) Heat map representing relative peak areas of acidified methanol extracted flavonol glycosides and isoflavones from the first trifoliate leaf of CBB-resistant and CBB-susceptible RIL plants. For each RIL/ inoculation treatment/ post-inoculation (PI) sampling time, three separate experimental replicates were analyzed and are denoted by three separate heat map squares. The horizontal axis represents leaves that were sampled at 0, 8, 24 and 48 h PI with *Xap* (all squares above the black bar labeled *Xap*) or sterile water (all replicate squares above the white bar labeled mock). Metabolite profiles for treatment replicates and PI sampling periods corresponding to the CBB-resistant RIL and CBB-susceptible RIL are provided on the left and right side of the heat map, respectively. The UHPLC-MS/MS peak area for each RIL/ inoculation treatment/ PI sampling time replicate is represented by a yellow to red square that corresponds to the peak area scale at the top of the heat map. (**B**) Effect of *X. axonopodis* on the levels of isoflavones, coumestrol and phaseolliniosflavan in *P. vulgaris* leaves of plants with varying susceptibility to CBB as a function of the PI period. Metabolite levels are expressed as quercetin 3-*O*-glucoside equivalents per gram of fresh weight (FW). Each datum represents the mean ± standard error of three separate experiments. For each plot, data sharing the same letter are not statistically different at a *p*-value ≤ 0.05.

Ultra-high performance liquid chromatography-tandem mass spectrometry (UHPLC-MS/MS) identified 24 separate flavonoids and derived metabolites in the first trifoliate leaf of CBB-resistant and CBB-susceptible *P. vulgaris* RIL plants, including flavonol glycosides, isoflavones, isoflavans and coumestans (Figure 8A). There was evidence for flavonol glycoside profiles that were RIL-specific although these were little affected by *X. axonopodis*. For example, quercetin 3-*O*-glucuronide was present at 98% greater levels in the CBB-susceptible RIL, whereas the levels for a quercetin glucoside-xyloside type compound were 84% greater in the CBB-resistant RIL than the CBB-susceptible RIL. This genotype was associated with more pronounced levels of isorhamnetin glucuronide and kaempferol glucuronide as well as kaempferol rutinoside relative to the CBB-resistant RIL, but on the whole these profiles were not impacted by *X. axonopodis*. An effect of the pathogen treatment was most apparent for the relative peak areas of daidzein, genistein, coumestrol and phaseollinisoflavan. The retention times and/or MS/MS fragmentation patterns of daidzein, genistein, coumestrol and phaseollinisoflavan matched those of authentic standards or previously identified metabolites as described in Section 4.5. The peak areas of these compounds were compared to a standard range of quercetin 3-*O*-glucoside that was co-eluted with all *P. vulgaris* methanolic leaf extracts (Figure 8B). Daidzein levels were dramatically increased in *X. axonopodis* treated leaves of the CBB-resistant RIL 48 h PI relative to levels apparent at the time of inoculation, whereas the levels of this metabolite were unaffected in mock-treated leaves. The pathogen treatment had a similar impact on the levels of genistein, coumestrol and phaseollinisoflavan in the CBB-resistant RIL. At 48 h PI, genistein, coumestrol and phaseollinisoflavan levels were respectively 3.8-, 10.3- and 160-fold those detected in the leaves of mock-inoculated plants of this RIL. The pathogen did not alter isoflavone profiles at earlier PI periods. Moreover, in the susceptible RIL, daidzein, genistein, coumestrol and phaseollinisoflavan levels were not altered by *X. axonopodis*.

## 3. Discussion

In this study, the molecular and metabolic determinants of CBB-resistance were investigated in two RILs derived from the CBB-susceptible parent ‘OAC Seaforth’ and CBB-resistant parent ‘OAC Rex’ [8]. CBB symptoms including necrotic lesions were widely apparent on *X. axonopodis*-inoculated leaves of the CBB-susceptible RIL, whereas symptoms were dramatically lower in the CBB-resistant RIL. The occurrence of severe symptoms in the CBB-susceptible RIL within 14 d of pathogen inoculation mirrors the disease development timeline for other *P. vulgaris* genotypes [4,32,33]. The accelerated development of disease symptoms in the CBB-susceptible RIL is likely because disease inoculation experiments were performed under a constant high humidity in a controlled environment. This contrasts with studies performed under field or greenhouse conditions, where humidity conditions are not well controlled [34,35,36]. The disease severity ratings in the CBB-resistant RIL were below the subjective scale rating of 2, which is like that reported for field-grown ‘OAC Rex’ [10]. 

The comparative transcriptome analysis of inoculated leaves uncovered 800 DEGs in CBB-resistant and CBB-susceptible RILs treated with *X. axonopodis* relative to the mock treatment. The total number of DEGs across both RILs over the 48-h PI period fits with the 581 DEGs detected in the leaves of anthracnose-resistant and anthracnose–susceptible black bean near-isogenic lines within 72 h of inoculation with the hemi-biotroph *Colletotrichum lindemuthianum* [37]. At the point of inoculation with *X. axonopodis*, many DEGs were downregulated in both RILs. This matches the initial pathogen transcriptional response in Chinese cabbage following treatment with *Plasmodiophora brassicae* [38]. The number of total DEGs across the 48 h PI period for both the CBB-resistant and CBB-susceptible RIL investigated in our study is well below the 2576 and 4502 DEGs respectively identified in BAT93 and JaloEEP558 leaf discs infiltrated with *X. phaseoli* pv. *phaseoli* [32]. The greater number of DEGs in the previous study could be due to the use of leaf discs for pathogen infiltration and subsequent incubation in water-agar for up to 48 h, conditions yielding approximately 200 DEGs across BAT93 and JaloEEP558 associated with nitrogen metabolism, as well as 10 photosynthesis genes. By comparison, only one nitrogen metabolism gene with the same GO term (GO:0006807) was enriched in our study, and no photosynthesis related DEGs were enriched. The smaller complement of DEGs in our study may also be because we investigated the impact of the disease pathogen on two related RILs, rather than two unrelated *P. vulgaris* landraces, as was done in the previous study [32]. 

Phytoalexins that originate from isoflavones play a role in legumes challenged with fungal pathogens [25,39]. In our study, the levels of coumestrol and phaseollionisoflavan were enriched in the leaves of the CBB-resistant white bean RIL following infiltration with the bacterial pathogen *X. axonopodis*. This metabolic phenomenon coincided with the accumulation of their hypothetical precursor daidzein as well as the related isoflavone genistein. Similarly, various coumestans and isoflavans have been shown to accumulate in seedlings of several Phaseoleae species, including *P. vulgaris*, following treatment with *R. oryzae* [25]. Single molecule elicitors such as salicylic acid also induce the accumulation of these phytoalexins in *P. vulgaris* [24,40]. The maximal levels detected for daidzein, genistein, coumestrol and phaseollinisoflavan in *X. axonopodis*-inoculated leaves of the CBB-resistant RIL were on par to an order of magnitude lower than the concentrations detected in the cotyledons of various Colombian *P. vulgaris* cultivars following treatment with salicylic acid [24]. This slight discrepancy may be a consequence of the different cultivars and alternate elicitors used across the two studies. In fact, varying levels of isoflavones are apparent in the leaves of Mesomerican and Andean genotypes [29] and coumestrol levels in soybean are also affected by genotype [26]. 

The chalcone precursors of isoflavones are shared by enzymes operating in the flavonol biosynthesis pathway. It is known that *A. thaliana* leaves containing various flavonol glycosides can be toxic to the fungal necrotroph *Sclerotinia sclerotiorum* when the pathogen’s flavonol oxidation capabilities are genetically impaired [41]. Various flavonol glycosides were present across both white bean RILs tested in our study, but their levels were little impacted by *X. axonopodis*. Thus, flavonol glycosides appear to play no role in mitigating the resistance of *P. vulgaris* to CBB, although the possibility remains that their occurrence in the CBB-susceptible RIL is required for pathogenesis. There was little impact of *X. axonopodis* infiltration on flavonol glycoside levels in this genotype over the 48 h PI window. If the accumulation of flavonol glycosides or a subset thereof is crucial to CBB-susceptibility, it is likely that leaf tissues sampled after 48 h PI would contain decreased levels of these metabolites. In fact, a delay in the decline of flavonol glycoside concentrations occurs in *A. thaliana* 72 h PI with *S. sclerotiorum* [41]. The lack of a *X. axonopodis* effect on flavonol glycoside composition in both RILs studied here fits with the absence of an overall enrichment of differentially expressed flavonol biosynthesis genes. Conversely, there was an upregulation of the competing isoflavone biosynthesis pathway in the CBB-resistant RIL plants.

The alteration of isoflavone profiles in plants is largely dependent upon the upregulated expression of their biosynthetic genes [42], and the availability of their metabolic precursors, specifically flavanones (e.g., naringenin) [43]. Here, transcripts corresponding to genes involved in the conversion of phenylalanine to flavanones such as *PvPAL2* and *PvC4H3* were elevated in the CBB-resistant RIL in response to the invading disease bacteria. The induced transcription of *PAL* and/or increased PAL activity is associated with immunity to pathogens including those promoting bacterial blight symptoms in rice [44,45]. Several CHS genes and a CHI were also upregulated in the CBB-resistant RIL in response to *X. axonopodis*. This is not surprising as CHS multi-gene families exist in other plants species, with expression increased for some genes in response to microbial pathogens and/or their elicitors [46]. The reaction catalyzed by CHS is the first committed step of the flavonoid pathway, including the isoflavones.

Apart from the upregulation of early flavonoid biosynthesis genes, genes encoding enzymes involved in naringenin chalcone and naringenin conversion to isoflavones and their derivatives were also induced in the CBB-resistant white bean RIL in response to *X. axonopodis*. One of the initial steps in the production of isoflavones is the conversion of liquiritigenin to daidzein or naringenin to genistein by IFS (Figure 1). Prior research shows increased *IFS* expression in soybean challenged by *Pseudomonas syringae* leads to higher levels of daidzein and genistein [47]. Two putative IFS genes (*Phvul.003G051700* and *Phvul.003G074000*) were upregulated in the CBB-resistant RIL 48 h after *X. axonopodis* treatment, and in a temporal pattern that matched the accumulation of daidzein and genistein in these same leaf tissues. Thus, it is possible that one or both of these IFS genes encode for the enzyme involved in isoflavone production in resistant *P. vulgaris* challenged with the CBB pathogen. The dynamics of the accumulation of daidzein and genistein in our study fits with research showing phytoalexin levels increase in *P. vulgaris* within 16 to 48 h of infection with *Sclerotinia sclerotiorum*, and these metabolic phenomena are most pronounced in the resistant variety A195 [48]. Although the expression of an *ISOFLAVONE 7-O-GLUCOSYLTRANSFERASE* (*Phvul.004G103900*) was induced in the CBB-resistant RIL in response to the pathogen, no isoflavone glucosides were apparent with the UHPLC-MS/MS analysis. The possibility remains that isoflavone glucosides would accumulate beyond the 48 h PI period. Isoflavone glucosides, rather than isoflavans and coumestans, have been shown to occur at various stages of development in *P. vulgaris* plants grown under non-biotic stress [29], whereas the opposite trend was apparent in *X. axonopodis*-infiltrated *P. vulgaris* in our study, a trend that agrees with previous elicitor-based studies [24,25].

Coumestrol and phaseollinisoflavan are isoflavone derivatives that are known to occur in *P. vulgaris* and soybean [24,25,26] and are more prevalent in anthracnose-resistant Colombian bean cultivars as compared to anthracnose-susceptible cultivars [49]. Similarly, the levels for these phytoalexins accrued in *X. axonopodis* treated leaves of the CBB-resistant RIL in this study. Unfortunately, the full suite of enzymatic steps for their production in plants are unknown. The conversion of isoflavones to phytoalexins requires the coordinated induction of IFH, IFR and VR gene expression and activity of their encoded enzyme proteins [19,20,21,22,42]. Three IFH genes *(PvIFH1*, *PvIFH2*, *PvIFH3*) were upregulated in leaves of the CBB-resistant RIL. This mirrors the upregulation of IFH in barrel clover and alfalfa plants infected with *Rhizoctonia solani* and *Phoma medicaginis*, respectively [16,50]. IFH converts isoflavones to 2′-dihydroxyisoflavones [51]. The putative *P. vulgaris IFH*s detected in the current RNA-seq study potentially encode for enzymes involved in this same metabolism and could provide precursors for IFR-mediated reactions. *PvIFR2* and *PvIFH3* gene expression was induced by *X. axonopodis* in the CBB-resistant RIL, but it remains to be determined whether their gene products are involved in the formation of trihydroxyisoflavanones from IFH generated 2’-dihydroxyisoflavones. IFR is crucial for isoflavone metabolism and the interaction between legumes and microbes. For instance, a reduction in *PvIFR1 t*ranscripts by RNAi culminates in less nodulation of *P. vulgaris* roots by rhizobial bacteria [31]. Conversely, *IFR* overexpression in soybean promotes greater resistance to *Phytophthora sojae* [52]. In alfalfa, the production of the major phytoalexin medicarpin relies on the sequential activity of VR and 7,2′-dihydroxy-4′-methoxyisoflavanol dehydratase [28]. Interestingly, two putative VR genes (*Phvul.008G076500* and *Phvul.008G076600*) were upregulated in the CBB-resistant RIL upon inoculation with *X. axonopodis*. Similar transcriptional phenomena occur in alfalfa during its interaction with *P. medicaginis* [53] and barrel clover leaves responding to *Phakopsora pachyrhizi* [15]. 

The possibility remains that one or both upregulated *VR*s is pivotal to the biosynthesis of this phaseollinisoflavan in *X. axonopodis*-treated plants, as well as the subsequent action of pterocarpan synthase. In other legumes some pterocarpan synthases with protein sequence similarity to dirigent proteins are involved in the biosynthesis of pterocarpans like medicarpin, whereas others are involved in the production of pterocarpan precursors known as isoflav-3-enes [23,54] such as 7,2′-dihydroxy-4′-methoxyisoflav-3-ene (Figure 1). It is tempting to speculate that dirigent proteins with pterocarpan synthase activity play a role in isoflav-3-ene and/or phaseollinisoflavan production in *P. vulgaris* as transcript abundance for three dirigent-related protein genes (*Phvul.001G145600*, *Phvul.001G145700*, *Phvul.001G145800*) were elevated in the CBB-resistant RIL 48 h after *X. axonopodis* inoculation. Dirigent proteins play a role in many different plant-pathogen interactions [55,56,57]. Apart from medicarpin production, dirigent proteins are postulated to be involved in biosynthesis of lignans. Lignin and lignans can suppress disease symptoms in a variety of plant-pathogen interactions [58]. These pterocarpan synthases are also proposed to be involved in coumestrol biosynthesis, as an increase in the gene transcript levels for a dirigent protein (*Glyma.03G147700*) is evident in coumestrol-accumulating soybean [26]. Moreover, the upregulated expression of the soybean dirigent protein occurs together with the accumulation of transcripts for a carboxyesterase (*Glyma.02G134000*), an alpha/beta hydrolase (*Glyma.11G004200*) and a cation/carnitine transporter (*Glyma.13G284900*) in the high coumestrol containing leaves of the soybean genotype ‘Daewonkong’ relative to the low coumestrol genotype ‘SS0903-2B-21-1-2′ [26]. BLAST searches revealed in silico *P. vulgaris* protein homologs of the carboxyesterase (i.e., *Phvul.003G295200*) and alpha/beta hydrolase (i.e., *Phvul.004G133000*), but these are unlikely associated with coumestrol accumulation in the white bean resistance response to *X. axonopodis* due to the absence of their strict upregulation in the CBB-resistant RIL (Supplementary Information, Tables S18 and S23). Thus, coumestrol biosynthesis in *P. vulgaris* is likely dependent upon alternate enzymes to those proposed previously [26]. 

In addition to the upregulation of structural isoflavone pathway genes, there is also evidence for the induction of positive transcriptional regulators of this pathway with biotic stress. To this end, *GmMYB29A2* overexpression in soybean hairy roots is associated with increased expression of isoflavone biosynthesis genes such as *GmIFS2* and the accumulation of glyceollin based phytoalexins following treatment with *P. sojae* [59]. This pathogen response in soybean is also regulated by the transcription factor *Gm*NAC42 to some extent [60]. In our study, various MYB and NAC transcription factor genes were upregulated in the CBB-resistant white bean RIL with *X. axonopodis*, but a single MYB (*Phvul.007G273400*) with protein sequence similarity to *Gm*MYB29A2 homolog was associated with the enriched GO term DNA binding in the CBB-resistant RIL at 24 h PI relative to all other expressed genes (Supplementary Information, Table S54). By comparison, several *Gm*NAC42 homologs (*Phvul.001G192000*, *Phvul.006G188900*, *Phvul.008G194600*) were associated with the enriched DNA binding GO term in this same genotype but at 48 h PI (Supplementary Information, Table S55). Thus, the possibility remains that the putative MYB *Phvul.007G273400* and one of the abovementioned NAC transcription factors are positive regulators of the isoflavone pathway in response to the CBB pathogen in *P. vulgaris* resistant genotypes, and the absence of their upregulation may explain in part the susceptibility phenomenon in the CBB-susceptible RIL.

The research reported here indicates that the accumulation of isoflavones and related phytoalexins, as well as the upregulation of isoflavone pathway genes, are involved in the defense response of the *P. vulgaris* white bean RILs having resistance to *X. axonopodis*. These transcriptional and biochemical responses are otherwise absent in the CBB-susceptible RILs investigated in this study. Although the degree to which these individual mechanisms contribute to CBB resistance has not yet been assessed, they represent important targets for future studies aimed at increasing CBB resistance in *P. vulgaris*, including cultivars produced in geographical regions that are highly prone to this field disease.

## 4. Materials and Methods 

### 4.1. Plant Material

The study utilized a CBB-resistant RIL and a CBB-susceptible RIL. The RILs were generated at the University of Guelph (Guelph, ON, Canada) from a cross between the CBB-resistant inbred line ‘OAC Rex’ and the CBB-susceptible inbred line ‘OAC Seaforth’. In either case, the F_4_ RIL populations were produced via single seed descent from F_2_ seed and were tested for their response to CBB in a previously published paper [8]. A CBB-resistant RIL (BT6) and a CBB-susceptible RIL (BT44) were chosen from the non-segregating lines within the F_2_ derived F_4_ population and selfed twice to produce their respective F_6_ RILs.

### 4.2. Disease Inoculation

Seeds (F6 progeny) of both RILs were sown in 15 cm diameter pots containing approximately 5 cm of Sunshine^®^ Mix #4 soil (SunGro Horticulture, St. Catharines, ON, Canada) overlaid with 1.25 to 2.5 cm of Turface MVP mix (Plant Products, Leamington, ON, Canada) and then additional Sunshine^®^ Mix #4 to fill the pot. Turface was added to increase soil drainage capacity and to prevent over-saturation of soil following irrigation. The soil was saturated with a 100 times diluted fertilizer solution prepared from a concentrate (118 g L^−1^) of a water soluble Complete N:P:K (17:5:17) fertilizer (Plant Products, Leamington, ON, Canada). For each disease inoculation experiment, a minimum of 14 plants of each CBB-susceptible and CBB-resistant RIL were cultivated in each of two separate Conviron PGC20 controlled environment chambers (Controlled Environments Ltd. Winnipeg, MB, Canada). Plants were grown at a fixed light intensity of 300 µmol photons m^−2^ s^−1^ for a 16 h photoperiod, and under darkness for the 8 h night period. Chamber temperatures were 25 °C during the photoperiod and 20 °C in between photoperiods, and a constant relative humidity of 75%. Plants of both RILs were fertigated once per week with the aforementioned fertilizer solution, provided supplemental watering as required and grown to the first trifoliate leaf stage (17 d post-sowing) prior to the disease inoculation experiment. 

For each disease inoculation experiment, first trifoliate leaf stage plants inoculated with *X. axonopodis* and those inoculated with sterile water (mock) were spatially separated across separate controlled environment growth chambers to avoid cross-contamination. The *X. axonopodis* inoculation experiment was performed with a split-split plot design. Thus, within each experiment, the main plot (i.e., controlled environment growth chamber) was represented by the presence or absence of the pathogen, the sub-plot included CBB-susceptible and CBB-resistant RILs, and the sub-sub plot was the PI sampling times. The experiment was performed three separate times. For each disease inoculation experiment, a highly virulent *X. axonopodis* isolate (i.e., isolate 98) sampled from Ontario dry bean fields [61] was cultivated on yeast salt agar medium plates at 28 °C for 48 h. A single colony of *X. axonopodis* isolate 98 was resuspended in 15 mL of liquid yeast salt medium, and then 0.5 mL was transferred to ten separate fresh yeast salt agar plates and incubated at 28 °C for an additional 48 h. Thereafter, the inoculum was collected from all yeast salt agar plates and resuspended in with sterile water to achieve an *A_600_* of 0.25, which corresponds to 2.5 × 10^8^ colony forming units mL^−1^ based on an equivalence to *Escherichia coli* cell culture concentrations [62]. Fully opened first trifoliate leaves were inoculated using the multiple needle method [63]. Briefly, for each RIL plant within the *X. axonopodis* inoculation chamber, a sterilized multi-pronged device (i.e., Ashland^®^ Pin frog, 2.8 cm diameter) was used to puncture holes in all three leaflets of the first trifoliate leaf. Thereafter, sponges soaked in the *X. axonopodis* inoculum were placed on either side of the punctured leaf and gently pressed to force inoculum into the punctured leaflets. In a second chamber, an equivalent number of plants were inoculated with sterile water (mock-inoculated) using the same multiple-needle method. A separate sterile plant frog and sponges were used for the mock treatment. In both chambers, the inoculated plants were subjected to the same photoperiod and light intensity conditions as described above but included high humidity (90.5 ± 0.6%) at 28 °C continuously for a 48-h period. Thereafter, in both chambers the humidity and temperatures were returned to those conditions used prior to the inoculation period. All inoculated leaves were sampled at the 0, 8, 24 and 48 h PI. For each RIL and its respective inoculation treatment, a single inoculated leaflet was sampled from each of three randomly chosen plants. For each RIL/ inoculation treatment/ PI sampling time replicate, the three leaflet sub-samples were pooled, immediately transferred to liquid N_2_ and then stored at −80 °C. Thereafter, the pooled frozen leaflets were pulverized into a fine powder with a mortar and pestle under liquid N_2_ and used for gene expression and metabolite analyses as described in Section 4.3 and Section 4.5. All inoculated plants were periodically monitored for disease symptoms, with initial assessment at 7 to 8 d PI, and then monitored every 2 to 3 d thereafter up to 14 d PI. Disease ratings were based on the percentage of infection on inoculated or mock inoculated leaf on a 0–5 scale: 0 = 0% infection, 1 = 1–10%, 2 = 11–30%, 3 = 31–50%, 4 = 51–80% and 5 = 80% or greater as described previously [64]. During the 14-d PI period, a subset of non-inoculated leaves was removed to prevent accelerated senescence of the inoculated leaves due to shading. 

### 4.3. RNA Extraction and Sequencing

RNA-seq analysis was used to identify DEGs in *X. axonopodis*-inoculated versus mock-inoculated plants of both *P. vulgaris* RILs. For each inoculated sample, total RNA was extracted from approximately 100 mg of frozen pooled leaf powder using the RNeasy Plant Mini Kit (Qiagen, Toronto, ON, Canada) as per the manufacturer’s protocol. Each total RNA preparation was assessed for quality and quantity using a NanoDrop 1000 UV/Vis spectrophotometer (NanoDrop Technologies, Wilmington, DE, USA) and Agilent 2100 Bioanalyzer (Agilent Technologies, Mississauga, ON, Canada) following the manufacturers’ instructions. Total RNA was deemed acceptable if the RNA integrity number value was ≥7, the *A*_260_/*A*_230_ ratios were between 2 and 2.2, and the concentration was greater than or equal to 20 ng μL^−1^. Thereafter, mRNA enrichment, cDNA library preparation and sequencing were performed at the Centre for Applied Genomics at the Hospital for Sick Children (Toronto, ON, Canada). Briefly, for each sample, poly(A) mRNA was enriched from total RNA (800 ng) using a NEBNext Poly(A) mRNA Magnetic Isolation module as per the manufacturer’s instructions (New England Biolabs, Whitby, ON, Canada). The cDNA libraries were synthesized from the mRNA enriched samples using a NEBNext Ultra II directional RNA library prep kit (New England Biolabs, Whitby, ON, Canada) and sequenced in a single lane of the Illumina NovaSeq 6000 platform generating 150 bp paired end reads.

### 4.4. Transcript Estimation and Analysis 

Trimming of paired-end reads was performed with the FASTQ Quality Trimmer (FASTX-Toolkit, http://hannonlab.cshl.edu/fastx_toolkit/) toolkit; a standalone program was used (software downloaded January 2014). Reads with a minimum of 80% of the original sequence length (i.e., 120 bp) were maintained. Trimmed sequences were analyzed with the Cufflinks pipeline [65]. Briefly, using default parameters, Bowtie 2 and TopHat (version 2.1.1) were used to map the trimmed read pairs to the complete *P. vulgaris* G19833 genome (assembly version 2.1; https://phytozome.jgi.doe.gov/pz/portal.html#!info?alias=Org_Pvulgaris) (accessed on 30 August 2019). Cufflinks, Cuffmerge, and Cuffcompare run with the multi read correct option were used to estimate transcript abundances and annotate transcripts with their gene model IDs. For all mapped genes, we report genes’ transcript abundances. For each RIL, Cuffdiff was employed to identify DEGs between *X. axonopodis*-treated and mock-treated *P. vulgaris* plants at each PI sampling time (0, 8, 24 and 48 h). For example, for each RIL genes that were expressed in *X. axonopodis* inoculated plants at the time 0 of the PI period across all three experimental replicates were compared to genes expressed in all three experimental replicates of mock-inoculated plants of the same RIL at the time 0 of the PI period. Significant gene expression was determined using a false discovery rate of 0.01 after Benjamin-Hochberg correction for multiple testing. Thereafter, genes exhibiting a Log2-fold-change value >2 and/or <−2 and a q value ≤ 0.05 were selected with cummeRbund in the R programing package version 3.6.1 (https://www.r-project.org/) (last accessed on 12 July 2019) [66] was used to access Cuffdiff results. 

GO enrichment analysis was performed for all DEGs for both RILs at each PI sampling period. The web-based platform, agriGoV2 (http://systemsbiology.cau.edu.cn/agriGOv2/) (last accessed on 15 June 2020) [67] and its singular enrichment analysis tool was used to identify significantly enriched GO terms for all DEGs [68]. Molecular functions, cellular component, and biological process GO terms were evaluated. Fisher’s exact tests evaluated whether GO terms were enriched in the DEGs relative to the full suite of expressed genes in each RIL and their PI sampling periods. For the analysis, DEGs having transcript abundance levels >2 FPKM were evaluated. P values were corrected using a false discovery rate cut-off value of 0.05. All transcript-related datasets supporting the results and conclusions of this article are included as Supplementary Materials.

### 4.5. Metabolite Analysis 

For each RIL/ inoculation treatment/ PI sampling time combination replicate, flavonols and phenolic acids were extracted from approximately 100 mg of frozen inoculated leaf tissue according to a previously published protocol [69]. Briefly, the frozen leaflet tissue was combined with 500 μL of methanol: acetic acid: Milli-Q water (9:1:10, *v*/*v*/*v*) containing 20 μM naringenin 7-*O*-glucoside as an internal standard, and transferred to a rotary shaker (Adams™ Nutator; Becton, Dickinson and Company, Franklin Lakes, NJ, USA) for 20 min, followed by centrifugation at 10,000× *g* for 10 min. The residue was re-extracted twice as described above. Supernatants form each successive extraction were pooled and passed through a 0.45 μm syringe filter (Mandel Scientific Company Inc., Guelph, ON, Canada) prior to UHPLC-MS/MS analysis. 

The leaf metabolite extracts were analyzed by UHPLC-MS/MS. Briefly, 5 μL of each sample was injected onto a Kinetex XB-C18 100A HPLC column (100 × 4.6 mm, 2.6 µm, Phenomenex, Torrence, CA, USA) connected to a Vanquish Flex Binary UPLC System (Waltham, MA, USA), with column temperature set at 40 °C and a flow rate of 0.7 mL min^−1^. The column was pre-equilibrated in solvent A, MilliQ H_2_O: formic acid (99.9: 0.1, *v*/*v*). The metabolites were eluted in a step-wise gradient with solvent B, methanol: acetonitrile: formic acid (94.4: 5: 0.1, *v*/*v*/*v*) as follows: 0–5 min, 0–12% B; 5–15 min, 12–23% B; 15–30 min, 23–50% B; 30–40 min, 50–80% B; 40–42 min, 80–100% B; 42–45 min, 100% B; 45–46 min, 100–0% B; 46–52 min, 0% B. The masses and fragmentation patterns of the eluted flavonoids (including isoflavones) and phenolic acids were detected with a Thermo Scientific Q-Exactive Orbitrap mass spectrometer. The mass spectrometer was operated in negative electrospray ionization mode utilizing the following settings: peak mass to width resolution of 35,000, a spray voltage of 2.8 kV, capillary temperature of 300 °C, sheath gas set at 55 units, and auxiliary gas set at 15 units. The data were acquired using Thermo Scientific Standard Integration Software and Thermo Scientific Xcalibur 4.2 using Full-MS mode for samples, and Full-MS/DDMS2 (TopN = 15, normalized collision energy = 30) for mixed quality control samples. To extract more fragmentation data, samples containing isoflavones, isoflavans and coumestans were reanalyzed at a normalized collision energy = 50. Data were visualized and analyzed using Thermo FreeStyle 1.5 software; Compound Discoverer 3.1 (CD) software was used for automated data processing in an untargeted analysis approach to look for compositional differences between the metabolite profiles of CBB-resistant and CBB-susceptible leaves, and their disease inoculation treatments over the 48-h PI period. Mass fragmentation patterns of compounds of interest within the leaf profiles were identified by comparison to MS/MS fragment data contained within an in-house library of flavonol and isoflavone compounds, as well as online databases (mzcloud [70], and METLIN [71]) (Supplementary Information, Table S56). The identity of the coumestrol peak in metabolite profiles of *P. vulgaris* leaves was confirmed by co-elution with an authentic standard (Sigma-Aldrich, Mississauga, ON, Canada). The MS/MS data for a peak eluting at 38.2 min (Supplementary Information, Figure S1) matched that of phaseollinisoflavan [25]. The acidified methanolic extraction of *P. vulgaris* leaves was deemed acceptable as the recovery of the spiked internal standard naringenin 7-*O*-glucoside was in the range of 91.8% to 95.6% across representative sample replicates of each RIL, inoculation treatment and PI sampling time. The UHPLC-MS/MS generated metabolite data were not corrected for the recovery of the internal standard. For quantification of metabolite levels in *P. vulgaris* leaves, their respective peak areas were compared to known amounts (0.54 to 5.39 pmol) of an authentic quercetin 3-*O*-glucoside standard; this commercially available flavonol glycoside was used as most of the extracted compounds from *P. vulgaris* leaves were identified as flavonols [29]. 

### 4.6. Statistical Analysis

Analysis of variance (ANOVA) was performed in SAS 9.4 (SAS Institute Inc., Cary, NC, USA) to determine significant differences at the α = 0.05 level for data related to disease ratings and transcript abundance (FPKM) levels of general flavonoid pathway and isoflavone pathway genes. For the statistical analysis, disease ratings and transcript data were from 48 different *P. vulgaris* leaf samples representing both RILs, their disease treatments (mock or *X. axonopodis*) and PI sampling period replicates across three separate experiments described under Section 4.2. The same statistical analysis was used to determine differences in metabolite levels for the aforementioned experimental replicates. All data were evaluated for normality, homogeneity, and independence of errors prior to statistical analysis. For data where the residuals did not fit normality, a lognormal distribution was used; for this a constant of 0.0001 was added to the data. Least squared means were backtransformed for final presentation. In addition, data for the metabolite daidzein was normally distributed after lognormal transformation in the absence of the zero-value data corresponding to all *X. axonopodis*-treated CBB-resistant RIL plants at the 0 h PI sampling period; thereafter the statistical analysis of least squared means was performed for the remaining RIL/ inoculation treatment/ PI sampling time replicates, including whether they were statistically different from the zero data for the aforementioned experimental replicates. For phaseollinisoflavan data, 95% confidence interval limits were used to assess differences between RIL/ inoculation treatment/ PI sampling time means. A Kenwood and Roger adjustment that corrects for multi-level models (i.e., split-split plot) was used for the mix model statement. Multiple means comparison with a Tukey-Kramer adjustment was used to test for significant differences within the gene expression and metabolite data for all 48 RIL/ inoculation treatment/ PI sampling time replicates.

## Data Availability

The RNA-seq experimental data presented for this study are from sequence information for mock and *X. axonopodis*-inoculated leaves of CBB-resistant and CBB-susceptible *P. vulgaris* RILs which are openly available at the National Center for Biotechnology Information Sequence Read Archive (https://www.ncbi.nlm.nih.gov/sra, accessed on 28 June 2021) under Bioproject PRJNA741786. All other data are presented within this article and its Supplementary Materials.

## Supplementary Materials

The following are available online at https://www.mdpi.com/article/10.3390/metabo11070433/s1, Table S1: Metrics for transcript generated by Illumina sequencing mapping to the *P. vulgaris* genome, Excel S1: Statistical analysis of DEGs in the CBB-resistant *Phaseolus vulgaris* RIL identified via Cuffdiff, Excel S2: Statistical analysis of DEGs in the CBB-susceptible *Phaseolus vulgaris* RIL identified via Cuffdiff, Table S2: Transcript abundance for genes upregulated in the CBB-resistant RIL at 0 h post-inoculation (PI) with *Xanthomonas axonopodis* (*Xap*), Table S3: Transcript abundance for genes upregulated in the CBB-resistant RIL at 8 h post-inoculation (PI) with *Xanthomonas axonopodis* (*Xap*), Table S4: Transcript abundance for genes upregulated in the CBB-resistant RIL at 24 h post-inoculation (PI) with *Xanthomonas axonopodis* (*Xap*), Table S5: Transcript abundance for genes upregulated in the CBB-resistant RIL at 48 h post-inoculation (PI) with *Xanthomonas axonopodis* (*Xap*); Table S6: Transcript abundance for genes upregulated in the CBB-susceptible RIL at 0 h post-inoculation (PI) with *Xanthomonas axonopodis* (*Xap*), Table S7: Transcript abundance for genes upregulated in the CBB-susceptible RIL at 8 h post-inoculation (PI) with *Xanthomonas axonopodis* (*Xap*), Table S8: Transcript abundance for genes upregulated in the CBB-susceptible RIL at 24 h post-inoculation (PI) with *Xanthomonas axonopodis* (*Xap*), Table S9: Transcript abundance for genes upregulated in the CBB-susceptible RIL at 48 h post-inoculation (PI) with *Xanthomonas axonopodis* (*Xap*), Table S10: Transcript abundance for genes downregulated in the CBB-resistant RIL at 0 h post-inoculation (PI) with *Xanthomonas axonopodis* (*Xap*), Table S11: Transcript abundance for genes downregulated in the CBB-resistant RIL at 8 h post-inoculation (PI) with *Xanthomonas axonopodis* (*Xap*), Table S12: Transcript abundance for genes downregulated in the CBB-resistant RIL at 24 h post-inoculation (PI) with *Xanthomonas axonopodis* (*Xap*), Table S13: Transcript abundance for genes downregulated in the CBB-resistant RIL at 24 h post-inoculation (PI) with *Xanthomonas axonopodis* (*Xap*), Table S14: Transcript abundance for genes downregulated in the CBB-susceptible RIL at 0 h post-inoculation (PI) with *Xanthomonas axonopodis* (*Xap*); Table S15: Transcript abundance for genes downregulated in the CBB-susceptible RIL at 8 h post-inoculation (PI) with *Xanthomonas axonopodis* (*Xap*), Table S16: Transcript abundance for genes downregulated in the CBB-susceptible RIL at 24 h post-inoculation (PI) with *Xanthomonas axonopodis* (*Xap*), Table S17: Transcript abundance for genes downregulated in the CBB-susceptible RIL at 48 h post-inoculation (PI) with *Xanthomonas axonopodis* (*Xap*), Table S18: Transcript abundance for genes downregulated in the CBB-resistant RIL and the CBB-susceptible RIL at 0 h post-inoculation (PI) with *Xanthomonas axonopodis* (*Xap*), Table S19: Transcript abundance for genes upregulated in the CBB-resistant RIL and the CBB-susceptible RIL at 0 h post-inoculation (PI) with *Xanthomonas axonopodis* (*Xap*), Table S20: Transcript abundance for a gene upregulated in the CBB-resistant RIL at 0 h post-inoculation (PI) with *Xanthomonas axonopodis* (*Xap*) and 8 h thereafter, Table S21: Transcript abundance for a gene downregulated in the CBB-resistant RIL at 0 h post-inoculation (PI) with *Xanthomonas axonopodis* (*Xap*) and upregulated 24 h thereafter, Table S22: Transcript abundance for genes downregulated in the CBB-resistant RIL at 0 h post-inoculation (PI) with *Xanthomonas axonopodis* (*Xap*) and upregulated 48 h PI, Table S23: Transcript abundance for a gene downregulated in the CBB-resistant RIL at 0 h post-inoculation (PI) with *Xanthomonas axonopodis* (*Xap*) and upregulated in the CBB-susceptible RIL 24 h PI, Table S24: Transcript abundance for a gene downregulated in the CBB-resistant RIL at 0 h post-inoculation (PI) with *Xanthomonas axonopodis* (*Xap*) and upregulated in the CBB-susceptible RIL 48 h PI, Table S25: Transcript abundance for a gene downregulated in the CBB-resistant RIL at 0 h post-inoculation (PI) with *Xanthomonas axonopodis* (*Xap*) and upregulated 24 and 48 h PI, Table S26: Transcript abundance for genes upregulated in both RILs at 0 h post-inoculation (PI) with *Xanthomonas axonopodis* (*Xap*), and 8 h PI in the CBB-resistant RIL, Table S27: Transcript abundance for genes downregulated at 0 h post-inoculation (PI) with *Xanthomonas axonopodis* (*Xap*) in both RILs, and upregulated 48 h PI in the CBB-resistant RIL, Table S28: Transcript abundance for a gene upregulated in the CBB-resistant and CBB-susceptible RIL at 0 h post-inoculation (PI) with *Xanthomonas axonopodis* (*Xap*) and downregulated in the CBB-resistant RIL 24 and 48 h PI, Table S29: Transcript abundance for a gene downregulated in the CBB-resistant RIL at 0 h post-inoculation (PI) with *Xanthomonas axonopodis* (*Xap*) and upregulated 48 h PI, and downregulated in the CBB-susceptible RIL at 0 h PI and upregulated 24 h PI, Table S30: Transcript abundance for a gene upregulated in the CBB-resistant RIL at 0 h post-inoculation (PI) with *Xanthomonas axonopodis* (*Xap*) and at all sampling times thereafter, and upregulated in the CBB-susceptible RIL at 0 h PI, Table S31: Transcript abundance for a gene upregulated in the CBB-resistant RIL at 8 h and 48 h post-inoculation (PI) with *Xanthomonas axonopodis* (*Xap*), Table S32: Transcript abundance for a gene downregulated in the CBB-resistant RIL at 8 h post-inoculation (PI) with *Xanthomonas axonopodis* (*Xap*) and upregulated at 48 h PI, Table S33: Transcript abundance for a gene upregulated in the CBB-resistant RIL at 8 and 48 h post-inoculation (PI) with *Xanthomonas axonopodis* (*Xap*) and upregulated in the CBB-susceptible RIL at 0 h PI, Table S34: Transcript abundance for genes respectively upregulated and downregulated in the CBB-resistant RIL at 24 and 48 h post-inoculation (PI) with *Xanthomonas axonopodis* (*Xap*), Table S35: Transcript abundance for genes upregulated in the CBB-resistant RIL at 24 h post-inoculation (PI) with *Xanthomonas axonopodis* (*Xap*) and at 0 h PI in the CBB-susceptible RIL, Table S36: Transcript abundance for genes upregulated in the CBB-resistant RIL at 24 h post-inoculation (PI) with *Xanthomonas axonopodis* (*Xap*) and in the CBB-susceptible RIL at 48 h PI, Table S37: Transcript abundance for genes upregulated in the CBB-resistant RIL at 24 and 48 h post-inoculation (PI) with *Xanthomonas axonopodis* (*Xap*) and at 0 h PI in the CBB-susceptible RIL, Table S38: Transcript abundance for a gene upregulated in the CBB-resistant RIL at 24 and 48 h post-inoculation (PI) with *Xanthomonas axonopodis* (*Xap*) and downregulated at 48 h PI in the CBB-susceptible RIL, Table S39: Transcript abundance for a gene upregulated in the CBB-resistant RIL at 24 h post-inoculation (PI) with *Xanthomonas axonopodis* (*Xap*) and in the CBB-susceptible RIL at 24 and 48 h PI, Table S40: Transcript abundance for a gene upregulated in both RILs at 24 and 48 h post-inoculation (PI) with *Xanthomonas axonopodis* (*Xap*), Table S41: Transcript abundance for genes upregulated in the CBB-resistant RIL at 48 h post-inoculation (PI) *Xanthomonas axonopodis* (*Xap*) and downregulated in the CBB-susceptible RIL at 0 h PI, Table S42: Transcript abundance for genes upregulated in the CBB-resistant RIL at 48 h post-inoculation (PI) with *Xanthomonas axonopodis* (*Xap*) and downregulated in the CBB-susceptible RIL at 8 h PI, Table S43: Transcript abundance for genes upregulated in the CBB-resistant RIL at 48 h post-inoculation (PI) with *Xanthomonas axonopodis* (*Xap*) and in the CBB-susceptible RIL at 24 h PI, Table S44: Transcript abundance for genes that were adversely regulated across both RILs at 48 h post-inoculation (PI) with *Xanthomonas axonopodis* (*Xap*), Table S45: Transcript abundance for a gene upregulated in the CBB-resistant RIL at 48 h post-inoculation (PI) with *Xanthomonas axonopodis* (*Xap*) and downregulated at 0 h PI and upregulated at 24 h PI in the CBB-susceptible RIL, Table S46: Transcript abundance for a gene upregulated in the CBB-resistant RIL at 48 h post-inoculation (PI) with *Xanthomonas axonopodis* (*Xap*) and downregulated at 0 h PI and upregulated at 48 h PI in the CBB-susceptible RIL, Table S47: Transcript abundance for a gene downregulated in the CBB-resistant RIL at 48 h post-inoculation (PI) with *Xanthomonas axonopodis* (*Xap*) and downregulated at 24 h PI and upregulated at 48 h PI in the CBB-susceptible RIL, Table S48: Transcript abundance for a gene downregulated in the CBB-susceptible RIL at 0 h post-inoculation (PI) with *Xanthomonas axonopodis* (*Xap*) and upregulated at 8 h PI, Table S49: Transcript abundance for a gene upregulated in the CBB-susceptible RIL at 0 h and 48 h post-inoculation (PI) with *Xanthomonas axonopodis* (*Xap*), Table S50: Transcript abundance for a gene upregulated in the CBB-susceptible RIL at 0 h post-inoculation (PI) with *Xanthomonas axonopodis* (*Xap*) and at 8 and 24 h PI and downregulated at 48 h PI, Table S51: Transcript abundance for genes adversely regulated (up or up and down) in the CBB-susceptible RIL at 8 h and 24 h post-inoculation (PI) with *Xanthomonas axonopodis* (*Xap*), Table S52: Gene ontology (GO) terms enriched across the differentially expressed genes of the CBB-susceptible RIL at 0 h post-inoculation with *Xanthomonas axonopodis* (*Xap*), Table S53: Gene ontology (GO) terms enriched across the differentially expressed genes of the CBB-resistant RIL at 0 h post-inoculation with *Xanthomonas axonopodis* (*Xap*), Table S54: Gene ontology (GO) terms enriched across the differentially expressed genes of the CBB-resistant RIL at 24 h post-inoculation with *Xanthomonas axonopodis* (*Xap*), Table S55: Gene ontology (GO) terms enriched across the differentially expressed genes of the CBB-resistant RIL at 48 h post-inoculation with *Xanthomonas axonopodis* (*Xap*), Table S56: UHPLC-MS/MS identification of flavonols and isoflavones in acidified methanolic extracts of leaves sampled from CBB-resistant and CBB-susceptible *P. vulgaris* leaves, Figure S1: Negative ion UHPLC-MS/MS analysis of the leaves of the CBB-resistant RIL following *X. axonopodis* inoculation detected a peak (retention time = 38.2 min) with MS/MS fragmentation data matching phaseollinisoflavan.
