# Supplementary material for: The Induction of the Isoflavone Biosynthesis Pathway Is Associated with Resistance to Common Bacterial Blight in Phaseolus vulgaris L
Source: Metabolites. 2021 Jul 1;11(7):433. doi: 10.3390/metabo11070433 (PMC8306140; doi:10.3390/metabo11070433)

Table S1. Metrics for transcripts generated by Illumina sequencing mapping to the *P. vulgaris* genome. Transcript mapping is for mRNA libraries from CBB-susceptible and CBB-resistant RIL plant leaves sampled post-inoculation (PI) with water (mock) and *X. axonopodis* (*Xap*).

RIL	Treatment	PI (h)	Raw reads	Trimmed reads	Total mappings	Single mappings	Multi mappings	Alignment (%)	Transcripts
**CBB-susceptible**	Mock	0	89002269 (±6.3%)	88980236 (±6.3%)	70938331 (±6.0%)	61690532 (±6.1%)	9247798 (±6.8%)	79.8 (±0.4%)	44182 (±0.7%)
	Mock	8	98043419 (±6.2%)	98018530 (±6.2%)	77433697 (±5.8%)	68355937 (±5.1%)	9077760 (±10.9%)	79.0 (±0.6%)	43538 (±2.5%)
	Mock	24	92254389 (±8.2%)	92229654 (±8.2%)	73059843 (±8.1%)	65787819 (±8.2%)	7272024 (±7.3%)	79.3 (±0.2%)	44632 (±0.8%)
	Mock	48	97238219 (±4.6%)	97212573 (±4.6%)	77152959 (±4.6%)	70954344 (±4.4%)	6198615 (±12.2%)	79.4 (±0.1%)	44663 (±0.4%)
	Xap	0	81708983 (±3.4%)	81686076 (±3.4%)	64649642 (±3.6%)	55136256 (±3.5%)	9513386 (±4.7%)	79.1 (±0.2%)	43583 (±0.2%)
	Xap	8	99910895 (±5.0%)	99883764 (±5.0%)	78824174 (±5.4%)	70007671 (±4.7%)	8816503 (±11.1%)	78.9 (±0.5%)	42190 (±6.1%)
	Xap	24	89059555 (±2.5%)	89035464 (±2.5%)	69870807 (±2.8%)	63016977 (±3.7%)	6853830 (±7.5%)	78.5 (±0.6%)	44580 (±0.5%)
	Xap	48	85520810 (±4.8%)	85499423 (±4.8%)	66745366 (±3.5%)	60319696 (±4.2%)	6425670 (±13.8%)	78.7 (±0.6%)	44452 (±0.3%)
**CBB-resistant**	Mock	0	89306333 (±6.0%)	89285555 (±6.0%)	70294844 (±5.8%)	60446036 (±5.5%)	9848808 (±12.8%)	78.8 (±0.5%)	44022 (±0.1%)
	Mock	8	104029850 (±10.9%)	104004500 (±10.9%)	82477919 (±11.3%)	71323134 (±11.2%)	11154785 (±16.2%)	79.2 (±0.5%)	44590 (±0.4%)
	Mock	24	97594783 (±6.4%)	97572905 (±6.4%)	76965956 (±6.3%)	69518533 (±7.4%)	7447423 (±4.8%)	78.9 (±0.2%)	44897 (±0.5%)
	Mock	48	87986621 (±7.6%)	87966646 (±7.6%)	68734223 (±7.2%)	62959382 (±8.3%)	5774841 (±4.7%)	78.2 (±0.6%)	44044 (±2.0%)
	Xap	0	77550060 (±8.7%)	77532011 (±8.7%)	61824489 (±8.0%)	52424394 (±7.3%)	9400096 (±13.0%)	79.8 (±0.6%)	35826 (±22.0%)
	Xap	8	94459242 (±5.6%)	94236153 (±5.7%)	74577706 (±5.9%)	64493749 (±6.8%)	10083957 (±7.0%)	79.1 (±0.3%)	43219 (±2.3%)
	Xap	24	84607080 (±3.7%)	84586474 (±3.7%)	60158244 (±10.3%)	54746523 (±9.9%)	5411721 (±14.8%)	79.3 (±1.1%)	37638 (±19.0%)
	Xap	48	90716979 (±1.6%)	90692987 (±1.6%)	70755116 (±2.1%)	65649321 (±1.6%)	5105795 (±12.8%)	78.0 (±0.9%)	45085 (±0.02%)

[1]Data represents the mean ± percent standard error (in parentheses) of three experimental replicates.

Tables S2 to S51 list upregulated and/or downregulated *P. vulgaris* genes in leaves of a CBB-resistant RIL and/or a CBB-susceptible RIL following inoculation with *Xanthomonas axonopodis* (*Xap*). Significant DEGs had a q value ≤ 0.05; this statistical information is provided in Excel S1 and Excel S2 spreadsheets of the Supplementary Information. **In Tables S2 to S51, the significant upregulated DEGs (as statistically determined in Excel S1 & S2) are shaded dark grey for the *Xap* treatment relative to the light grey mock treatment at the specified PI sampling time; the significant downregulated DEGs (as statistically determined in Excel S1 & S2) are shaded dark grey for the mock treatment relative to light grey for the *Xap* treatment at the specified PI period. Non-shaded data are not significantly different at the specified PI sampling period.**

Table S2. Transcript abundance for genes upregulated in the CBB-resistant RIL at 0 h post-inoculation (PI) with *Xanthomonas axonopodis* (*Xap*). Transcript levels for individual genes corresponding to each RIL/ inoculation treatment/ sampling time PI are represented as FPKM ± SE of three experimental replicates.

		Transcript levels (FPKM)							
		Mock inoculation				*Xap* inoculation			
**Gene ID**	**Gene Annotation**	**0 h PI**	**8 h PI**	**24 h PI**	**48 h PI**	**0 h PI**	**8 h PI**	**24 h PI**	**48 h PI**
Phvul.002G024200	Domain of unknown function	0.78 ±0.21	0.75 ±0.36	1.58 ±0.24	2.61 ±0.85	3.44 ±0.47	0.37 ±0.23	1.88 ±0.07	1.06 ±0.31
Phvul.002G231600	17.6 kDa class I heat shock protein 1-related	2.71 ±0.83	2.89 ±0.29	15.88 ±0.93	24.34 ±2.67	28.86 ±14.77	10.36 ±6.6	20.35 ±1.8	27.81 ±5.07
Phvul.002G240100	Receptor like protein 55	0.62 ±0.37	0.4 ±0.24	0.81 ±0.17	0.62 ±0.1	2.75 ±0.54	0.56 ±0.38	0.75 ±0.17	0.31 ±0.12
Phvul.003G203400	No functional annotation	2.15 ±0.38	1.8 ±0.69	5.56 ±0.76	4.57 ±1.4	9.25 ±3.77	3.82 ±2.37	5.37 ±0.96	5.23 ±0.19
Phvul.004G107700	Heat shock protein 90	51.05 ±18.33	29.26 ±7.86	121.17 ±10.06	112.91 ±13.09	222.51 ±80.19	81.13 ±60.3	130.61 ±8.42	126.16 ±20.74
Phvul.007G060900	Protein IQ-domain 19	0.26 ±0.12	0.38 ±0.08	0.34 ±0.09	0.74 ±0.02	1.07 ±0.18	0.29 ±0.2	0.48 ±0.18	0.46 ±0.15
Phvul.008G000200	Protein tyrosine kinase / Di-glucose binding within endoplasmic reticulum	0.39 ±0.06	0.41 ±0.28	0.44 ±0.08	0.48 ±0.09	1.64 ±0.59	0.51 ±0.27	0.37 ±0.09	0.6 ±0.31
Phvul.009G072700	No functional annotation	0.98 ±0.42	0.48 ±0.16	1.65 ±0.53	0.53 ±0.12	3.95 ±1.59	1.16 ±0.65	1 ±0.02	0.14 ±0.05
Phvul.011G183500	Pathogenesis-related protein Bet v I family	0.51 ±0.14	0.97 ±0.45	0.21 ±0.15	0.06 ±0.06	2.82 ±1.51	0.56 ±0.23	0.19 ±0.18	0 ±0

Table S3. Transcript abundance for genes upregulated in the CBB-resistant RIL at 8 h post-inoculation (PI) with *Xanthomonas axonopodis* (*Xap*). Transcript levels for individual genes corresponding to each RIL/ inoculation treatment/ sampling time PI are represented as FPKM ± SE of three experimental replicates.

		**Transcript levels (FPKM)**							
		**Mock inoculation**				***Xap* inoculation**			
**Gene ID**	**Gene Annotation**	**0 h PI**	**8 h PI**	**24 h PI**	**48 h PI**	**0 h PI**	**8 h PI**	**24 h PI**	**48 h PI**
Phvul.006G075600	Peroxidase / Lactoperoxidase	0.22 ±0.11	0.34 ±0.23	0.16 ±0.07	0.24 ±0.12	0.28 ±0.12	1.76 ±0.81	0.5 ±0.32	0.93 ±0.26

Table S4. Transcript abundance for genes upregulated in the CBB-resistant RIL at 24 h post-inoculation (PI) with *Xanthomonas axonopodis* (*Xap*). Transcript levels for individual genes corresponding to each RIL/ inoculation treatment/ sampling time PI are represented as FPKM ± SE of three experimental replicates.

		Transcript levels (FPKM)							
		Mock inoculation				*Xap* inoculation			
**Gene ID**	**Gene Annotation**	**0 h PI**	**8 h PI**	**24 h PI**	**48 h PI**	**0 h PI**	**8 h PI**	**24 h PI**	**48 h PI**
Phvul.001G002200	No functional annotation	3.06 ±1.25	1.34 ±0.5	1.53 ±0.35	7.96 ±7.03	3.43 ±0.67	1.48 ±0.72	12.16 ±5.77	21.46 ±12.11
Phvul.001G170800	Zinc finger protein, ZAT11	1.39 ±0.58	0.49 ±0.3	0.47 ±0.15	4.1 ±3.99	1.17 ±0.29	0.53 ±0.1	2.54 ±1.4	7.92 ±5.38
Phvul.001G203300	Glutaredoxin-C11-related	0.19 ±0.18	0 ±0	0 ±0	0.44 ±0.44	0.45 ±0.45	0.41 ±0.23	1.11 ±0.64	0.15 ±0.16
Phvul.001G243600	Serine/threonine-protein kinase-like protein	0.57 ±0.22	0.15 ±0.04	0.21 ±0.03	0.88 ±0.79	0.51 ±0.17	0.61 ±0.14	0.99 ±0.44	3.44 ±1.88
Phvul.002G275000	NAC domain-containing protein 2	3.1 ±0.5	3.35 ±0.58	2.37 ±0.45	9.52 ±7.16	3.01 ±0.57	4.14 ±0.6	10.58 ±4.02	30.27 ±9.17
Phvul.002G297100	WRKY DNA -binding domain	3.53 ±0.96	1.55 ±0.5	0.89 ±0.07	8.22 ±6.7	2.09 ±0.47	1.89 ±0.12	17.8 ±8.44	20.63 ±14.86
Phvul.003G292400	AP2 domain	0.09 ±0.09	0 ±0	0 ±0	0.14 ±0.14	0.09 ±0.05	0 ±0	0.67 ±0.41	0.72 ±0.43
Phvul.003G030601	VQ motif	0.29 ±0.04	0.26 ±0.13	0.45 ±0.21	2.07 ±1.39	0.25 ±0.12	0.38 ±0.1	2.22 ±0.93	4.22 ±1.42
Phvul.003G034400	Protein of unknown function	4.07 ±0.55	3.27 ±0.8	2.15 ±0.17	8.42 ±5.9	4.61 ±1.07	4.24 ±0.44	17.2 ±7.6	29.46 ±15.72
Phvul.003G048800	No functional annotation	3.6 ±1.27	2.57 ±0.55	2.42 ±0.26	10.53 ±6.3	5.73 ±1	4.13 ±2.02	11.55 ±4.4	22.15 ±12
Phvul.003G251200	Glycosyltransferase 8 domain-containing protein	0.41 ±0.21	0.28 ±0.13	0.29 ±0.02	0.2 ±0.05	0.56 ±0.14	0.42 ±0.38	1.41 ±0.83	1.11 ±0.88
Phvul.004G162500	Arabinogalactan peptide 16-related	6.83 ±1.52	5.92 ±1.15	2.7 ±0.51	16.81 ±13.18	10.82 ±2.16	11.1 ±2.01	21.87 ±10.18	65.9 ±35.02
Phvul.005G057400	Expressed protein	1.24 ±0.8	1.45 ±0.55	0.56 ±0.29	2.17 ±2.03	1.61 ±0.54	1.83 ±0.59	8.08 ±4.05	15.97 ±12.08

Table S4 continued

		Transcript levels (FPKM)							
		Mock inoculation				*Xap* inoculation			
**Gene ID**	**Gene Annotation**	**0 h PI**	**8 h PI**	**24 h PI**	**48 h PI**	**0 h PI**	**8 h PI**	**24 h PI**	**48 h PI**
Phvul.005G079650	No functional annotation	0.91 ±0.57	0.17 ±0.17	0 ±0	0.31 ±0.31	0 ±0	0.3 ±0.3	1.43 ±0.88	3.62 ±3.11
Phvul.005G100600	No functional annotation	6.32 ±0.38	5.91 ±0.6	2.5 ±0.16	6.74 ±3.48	10.15 ±2	7.66 ±1.71	13.02 ±4.95	25.24 ±12.62
Phvul.005G122501	NAC domain-containing protein 61-related	0.54 ±0.23	0.23 ±0.2	0.14 ±0.06	0.39 ±0.32	0.52 ±0.15	0.22 ±0.2	1.96 ±0.85	2.02 ±1.77
Phvul.006G165800	Protein TIFY 5A-related	0.09 ±0.01	0.27 ±0.14	0 ±0	0.1 ±0.06	0.1 ±0.06	0.16 ±0.08	0.71 ±0.53	0.97 ±0.37
Phvul.006G183100	AP2 domain	1.76 ±0.56	1.49 ±0.23	1.17 ±0.13	6.42 ±4.31	1.84 ±0.64	2.03 ±1.03	7.49 ±3.21	22.23 ±8.83
Phvul.006G111700	WRKY DNA-binding domain	5.77 ±1.3	3.91 ±1.18	3.4 ±0.3	9.6 ±6.1	5.47 ±1.06	4.57 ±1.31	15.74 ±5.96	27.1 ±15.65
Phvul.007G035500	Domain of unknown function	1.19 ±0.3	1.01 ±0.38	0.3 ±0.07	2.71 ±2.25	1.37 ±0.54	2 ±0.55	2.13 ±1.07	7.06 ±3.85
Phvul.007G273400	MYB-like DNA-binding protein	2.83 ±0.26	2.69 ±0.04	1.44 ±0.13	6.65 ±4.36	2.29 ±0.87	3.28 ±0.47	8.62 ±3.74	19.2 ±5.59
Phvul.007G066500	Dehydration-responsive element-binding protein 1A-related	0.21 ±0.13	0 ±0	0 ±0	0.07 ±0.08	0.16 ±0.16	0 ±0	1.15 ±1	0.89 ±0.72
Phvul.008G169800	No functional annotation	3.53 ±1.42	2.48 ±0.9	2.49 ±0.39	10.1 ±7.84	4.55 ±1.84	4.05 ±1.78	12.75 ±5.35	38.82 ±19.14
Phvul.008G225500	Hs1pro-1 protein C-terminus	3.31 ±0.66	2.36 ±0.43	1.23 ±0.3	9.65 ±6.92	1.5 ±0.28	2.54 ±0.42	10.38 ±4.99	28.69 ±14.66
Phvul.008G235100	Calcium-binding protein CML24-related	11.71 ±4.09	5.83 ±0.94	4.64 ±0.38	10.07 ±5.42	20.69 ±5.54	12.34 ±3.43	22.38 ±9.39	27.71 ±20.37
Phvul.008G238500	U-Box domain-containing protein 54-related	0.47 ±0.1	0.23 ±0.13	0.23 ±0.07	2.61 ±2.46	0.25 ±0.03	0.2 ±0.06	2.33 ±1.03	6.17 ±3.34
Phvul.008G259800	Protein phosphatase 2C-like protein-related	5.72 ±1.65	3.74 ±0.19	4.7 ±0.42	13.69 ±8.36	4.4 ±0.72	4.21 ±1.07	19.06 ±8.08	33.16 ±20.24

Table S4 continued

Gene ID	Gene Annotation	Transcript levels (FPKM)							
		Mock inoculation				*Xap* inoculation			
**Gene ID**	**Gene Annotation**	**0 h PI**	**8 h PI**	**24 h PI**	**48 h PI**	**0 h PI**	**8 h PI**	**24 h PI**	**48 h PI**
Phvul.009G250400	E3 ubiquitin-protein ligase RNF38/44	10.87 ±2.96	5.59 ±1.15	3.35 ±0.57	10.02 ±5.94	16.63 ±4.79	14.3 ±8.04	21.43 ±9.45	37.04 ±21.33
Phvul.009G045601	No functional annotation	6.03 ±2.59	2.93 ±1.26	0.99 ±0.12	21.51 ±19.06	3.99 ±0.64	3.8 ±1.25	15.24 ±8.21	54.97 ±33.5
Phvul.009G045700	No functional annotation	3.06 ±1.27	1.33 ±0.58	1.72 ±0.1	12.02 ±10.56	2.26 ±0.31	1.95 ±0.59	15.44 ±7.61	42.79 ±26.05
Phvul.009G070800	C2H2-type zinc finger	22.26 ±9.12	8.81 ±3.67	11.06 ±1.33	59.09 ±42.64	21.58 ±4.56	15.63 ±8.53	51.39 ±20.43	142.33 ±63.62
Phvul.009G143800	Solute carrier family 25 (mitochondrial oxoglutarate transporter), member 11	0.44 ±0.01	0.69 ±0.33	0.16 ±0.06	3.21 ±3.03	0.5 ±0.07	0.77 ±0.13	4.83 ±2.61	8.22 ±5.75
Phvul.009G253300	COBRA-like protein 7-related	0.25 ±0.06	0.16 ±0.07	0.16 ±0.02	0.49 ±0.03	0.23 ±0.07	0.2 ±0.01	0.67 ±0.29	0.83 ±0.39
Phvul.010G062500	WRKY transcription factor 33	0.63 ±0.17	0.39 ±0.09	0.27 ±0.09	2.2 ±1.81	0.17 ±0.05	0.44 ±0.1	4.24 ±1.96	5 ±3.24
Phvul.010G120700	F11O4.3-related	0.42 ±0.06	0.45 ±0.13	0.16 ±0.03	0.38 ±0.13	0.87 ±0.1	0.54 ±0.25	0.71 ±0.08	1.09 ±0.38
Phvul.011G003100	Serine/threonine-protein kinase-like protein CCR4	2.76 ±0.88	1.47 ±0.37	0.9 ±0.22	4.46 ±3.59	4.93 ±0.89	2.8 ±1.41	13.81 ±6.67	12.45 ±9.68
Phvul.011G095500	NAC domain-containing protein 61-related	0.92 ±0.25	0.66 ±0.06	1.09 ±0.14	4.19 ±2.01	0.95 ±0.21	0.72 ±0.22	9.54 ±3.71	13.87 ±9.57
Phvul.011G097400	CCR4-associated factor 1 homolog 11-related	34.55 ±5.48	25.29 ±2.22	21.44 ±2.59	63.7 ±39.05	49.54 ±13.17	42.66 ±18.23	90.1 ±32.27	138.53 ±61.98
Phvul.011G036600	SAUR family protein	1.43 ±0.55	0.73 ±0.35	1.06 ±0.36	2.19 ±0.41	1.8 ±0.06	0.98 ±0.28	5.21 ±1.78	1.55 ±0.44
Phvul.011G048800	Domain of unknown function	11.61 ±3.38	5.27 ±1.59	3.84 ±0.83	19.13 ±14.99	6.23 ±1.36	7.9 ±1.93	15.52 ±5.75	37.94 ±21.02

Table S4 continued

		Transcript levels (FPKM)							
		Mock inoculation				*Xap* inoculation			
**Gene ID**	**Gene Annotation**	**0 h PI**	**8 h PI**	**24 h PI**	**48 h PI**	**0 h PI**	**8 h PI**	**24 h PI**	**48 h PI**
Phvul.007G074600, Phvul.007G074800, Phvul.007G074900, Phvul.007G075000	Copper transport family protein-related	19.06 ±2.85	13.92 ±3.49	7.49 ±0.83	21.02 ±12.89	20.46 ±3.67	15.55 ±2.8	36.77 ±13.54	58.03 ±33.06
XLOC_025168	No functional annotation	0 ±0	0.26 ±0.26	0 ±0	0.57 ±0.57	0 ±0	0.2 ±0.2	1.41 ±0.29	5.45 ±1.62

Table S5. Transcript abundance for genes upregulated in the CBB-resistant RIL at 48 h post-inoculation (PI) with *Xanthomonas axonopodis* (*Xap*). Transcript levels for individual genes corresponding to each RIL/ inoculation treatment/ sampling time PI are represented as FPKM ± SE of three experimental replicates.

		Transcript levels (FPKM)							
		Mock inoculation				*Xap* inoculation			
**Gene ID**	**Gene Annotation**	**0 h PI**	**8 h PI**	**24 h PI**	**48 h PI**	**0 h PI**	**8 h PI**	**24 h PI**	**48 h PI**
Phvul.001G019200	MYB-like DNA-binding protein	0.2 ±0.1	0.4 ±0.09	0.04 ±0.04	0.45 ±0.21	0.05 ±0.05	0.15 ±0.09	0.57 ±0.02	2.51 ±0.64
Phvul.001G019300	AR781	0.28 ±0.1	0.14 ±0.08	0.44 ±0.09	1.04 ±0.65	0.19 ±0.13	0.27 ±0.02	0.79 ±0.21	5.14 ±1.14
Phvul.001G039900	WRKY DNA-binding domain	2.49 ±0.5	3.36 ±0.68	2.11 ±0.35	2.83 ±0.95	2.56 ±0.7	3.39 ±0.17	4.01 ±1.16	16.61 ±5.46
Phvul.001G040000	L-type lectin-domain containing receptor kinase ix.1-related	0.17 ±0.13	0.16 ±0.05	0.02 ±0.01	0.07 ±0.05	0 ±0	0.15 ±0.05	0.09 ±0.02	0.52 ±0.05
Phvul.001G040700	L-type lectin-domain containing receptor kinase ix.1-related	1.61 ±0.32	1.99 ±0.85	2.47 ±0.54	2.99 ±0.72	1.95 ±0.18	1.51 ±0.33	2.96 ±0.31	23.94 ±3.27
Phvul.001G042100	WRKY DNA-binding domain	0.82 ±0.33	0.65 ±0.27	0.37 ±0.18	0.63 ±0.31	0.19 ±0.11	0.74 ±0.23	1.34 ±0.69	10.69 ±4.35
Phvul.001G042200	WRKY transcription factor 40-related	1.58 ±0.24	1.84 ±0.74	1.04 ±0.22	1.82 ±0.79	0.75 ±0.32	1.45 ±0.18	2.98 ±1.22	30.93 ±17.3
Phvul.001G051000	No functional annotation	0.08 ±0.05	0.19 ±0.1	0.12 ±0.02	0.1 ±0.03	0.01 ±0.01	0.06 ±0.02	0.19 ±0.13	1.28 ±0.76
Phvul.001G073600	E3 ubiquitin-protein ligase ATL42-related	0.03 ±0.01	0.05 ±0.04	0.1 ±0.03	0.18 ±0.05	0 ±0	0.14 ±0.04	0.09 ±0.09	0.98 ±0.23
Phvul.001G074200	Regulator of chromosome condensation	0 ±0	0.01 ±0.01	0.08 ±0.04	0.08 ±0.02	0.05 ±0.03	0.03 ±0.02	0.05 ±0.02	0.56 ±0.24
Phvul.001G088200	WRKY transcription factor 45-related	1.46 ±0.14	1.61 ±0.52	3.47 ±1.35	5.99 ±1.05	1.02 ±0.13	0.78 ±0.05	4.15 ±1.87	39.03 ±5.24
Phvul.001G095600	EF-hand calcium-binding domain containing protein	0.06 ±0.06	0.09 ±0.09	0.46 ±0.37	0.58 ±0.18	0.09 ±0.09	0.43 ±0.16	1.06 ±0.11	9.72 ±2.1

Table S5 continued

		Transcript levels (FPKM)							
		Mock inoculation				*Xap* inoculation			
**Gene ID**	**Gene Annotation**	**0 h PI**	**8 h PI**	**24 h PI**	**48 h PI**	**0 h PI**	**8 h PI**	**24 h PI**	**48 h PI**
Phvul.001G102300	Tetrahydrofolate dehydrogenase	4.13 ±1.1	3.62 ±1.66	2.46 ±0.15	1.61 ±0.21	5.17 ±0.79	2.95 ±0.84	2.75 ±0.69	7.63 ±0.69
Phvul.001G108101	MATE efflux family protein	1.91 ±0.36	1.86 ±0.83	2.97 ±0.68	2.76 ±0.54	2.44 ±0.18	1.43 ±0.29	2.65 ±0.16	11.25 ±3.38
Phvul.001G112400	Shikimate *O*-hydroxycinnamoyltransferase	0.14 ±0.07	0.05 ±0.04	0.31 ±0.13	0.18 ±0.09	0 ±0	0.15 ±0.08	0.22 ±0.07	5.38 ±0.78
Phvul.001G124700, Phvul.001G124800	Trans-aconitate 3-methyltransferase	4.17 ±1.29	4.04 ±2.26	2.6 ±0.57	2.13 ±0.35	4.59 ±0.59	3.55 ±0.99	4.57 ±1.4	30.48 ±15.91
Phvul.001G130200	Glycosyltransferase	0.3 ±0.16	0.55 ±0.14	0.39 ±0.18	0.57 ±0.29	0.28 ±0.09	0.45 ±0.1	1.05 ±0.41	6.58 ±2.93
Phvul.001G131000	Heat shock transcription factor, other eukaryote	0.14 ±0.06	0.1 ±0.05	0.19 ±0.08	0.18 ±0.05	0.05 ±0.03	0.09 ±0.03	0.33 ±0.16	3 ±0.42
Phvul.001G135200	Raffinose synthase	2.09 ±0.24	2.16 ±0.12	2.33 ±0.2	3.2 ±0.92	1.38 ±0.32	1.64 ±0.24	2.68 ±0.18	18.72 ±11.32
Phvul.001G142000	Late embryogenesis abundant protein 4-5	1.57 ±1.47	2.42 ±1.4	0.17 ±0.06	0 ±0	0.03 ±0.03	2.88 ±1.77	0.42 ±0.29	3.53 ±1.6
Phvul.001G155150, Phvul.001G155201	Cation transporting ATPase, C-terminus / Haloacid dehalogenase-like hydrolase	4.84 ±0.26	5.36 ±1.64	3.88 ±0.81	5.93 ±1.45	3.89 ±0.44	5.09 ±0.16	8.59 ±2.2	32.33 ±8.23
Phvul.001G155400	Calcium binding protein	1.43 ±0.33	1.09 ±0.05	1.5 ±0.09	1.63 ±0.59	0.52 ±0.12	1.75 ±0.39	2.19 ±0.07	12.17 ±2.2
Phvul.001G160100	Ethylene-responsive transcription factor, ERF096	0.26 ±0.18	0.37 ±0.09	0.61 ±0.27	1.48 ±1.3	0.37 ±0.13	0.27 ±0.2	1.26 ±0.54	8.24 ±6.77
Phvul.001G164900	Auxin-responsive protein, IAA10-related	0.64 ±0.14	0.53 ±0.31	0.81 ±0.24	0.68 ±0.03	0.35 ±0.06	0.43 ±0.14	0.97 ±0.14	3.5 ±1.24
Phvul.001G169300	Cation/$H^{(+)}$ antiporter 20	0.07 ±0.04	0.08 ±0.07	0.31 ±0.13	0.17 ±0.08	0.28 ±0.12	0.07 ±0.05	0.14 ±0.03	2.26 ±0.42

Table S5 continued

		Transcript levels (FPKM)							
		Mock inoculation				*Xap* inoculation			
**Gene ID**	**Gene Annotation**	**0 h PI**	**8 h PI**	**24 h PI**	**48 h PI**	**0 h PI**	**8 h PI**	**24 h PI**	**48 h PI**
Phvul.001G177700	Phenylalanine ammonia-lyase	4.14 ±0.49	4.02 ±2.34	5.06 ±0.21	6.61 ±1.93	3.4 ±0.35	3.53 ±0.79	8.09 ±1.95	37.89 ±3.72
Phvul.001G192000	No apical meristem (NAM) protein	0.47 ±0.18	0.58 ±0.39	0.38 ±0.06	0.7 ±0.27	0.82 ±0.2	0.43 ±0.18	0.75 ±0.16	3.42 ±1.54
Phvul.001G194600	Harpin-induced protein-like-related	3.23 ±0.54	2.73 ±0.46	2.31 ±0.48	3.88 ±1.63	2.39 ±0.11	3.05 ±0.29	6.67 ±2.68	34.47 ±10.59
Phvul.001G223700	Inositol 3-α-galactosyltransferase / UDP-α-D-galactose: myo-inositol 3-α-D-galactosyltransferase	0.57 ±0.18	0.26 ±0.05	0.53 ±0.25	0.58 ±0.2	0.42 ±0.06	0.57 ±0.07	0.52 ±0.19	8.94 ±2.69
Phvul.001G226000	Calmodulin binding protein-like	0.52 ±0.06	0.69 ±0.39	0.42 ±0.1	0.73 ±0.25	0.7 ±0.09	0.52 ±0.22	1.53 ±0.49	13.36 ±3.72
Phvul.001G226100	Calmodulin binding protein-like	3.01 ±0.53	2.66 ±0.13	4.03 ±0.14	3.87 ±1.31	5.37 ±1.22	3.98 ±0.56	8.59 ±2.51	17.12 ±9.05
Phvul.001G258900	NADH-ubiquinone reductase complex 1 MLRQ subunit (B12D)	1.17 ±0.17	1.28 ±0.42	2.12 ±0.49	1.19 ±0.44	1.22 ±0.48	1.48 ±0.81	3 ±1.32	12.66 ±1.82
Phvul.001G264400	K13899 - Cystatin-C (CST3)	0.34 ±0.35	0 ±0	1.41 ±0.59	1.07 ±0.41	0.36 ±0.36	0.08 ±0.08	0.46 ±0.18	4.88 ±2.13
Phvul.002G009700	VQ motif	0.34 ±0.18	0.12 ±0.12	0.31 ±0.18	0 ±0	0 ±0	0.39 ±0.27	0.78 ±0.16	1.43 ±0.59
Phvul.002G014700	Isoflavone 2'-hydroxylase	1.46 ±0.27	1.02 ±0.08	1.37 ±0.45	0.91 ±0.18	0.68 ±0.12	1.52 ±0.63	1.82 ±0.38	9.06 ±1.09
Phvul.002G019100	Heat stress transcription factor B-1	0.49 ±0.05	0.41 ±0.16	1.15 ±0.21	1.3 ±0.23	0.38 ±0.06	0.46 ±0.12	1.53 ±0.45	10.84 ±3.52
Phvul.002G021800	Reticulon-like protein B13	0 ±0	0.02 ±0.02	0 ±0	0 ±0	0 ±0	0.05 ±0.03	0.08 ±0.08	0.72 ±0.69

Table S5 continued

		Transcript levels (FPKM)							
		Mock inoculation				*Xap* inoculation			
**Gene ID**	**Gene Annotation**	**0 h PI**	**8 h PI**	**24 h PI**	**48 h PI**	**0 h PI**	**8 h PI**	**24 h PI**	**48 h PI**
Phvul.002G025000	Premnaspirodiene oxygenase	0.1 ±0.03	0.07 ±0.05	0.24 ±0.17	0.27 ±0.08	0.02 ±0.02	0.08 ±0.06	0.61 ±0.28	2.75 ±0.36
Phvul.002G029900	No functional annotation	0.08 ±0.08	0.11 ±0.03	0.14 ±0.09	0.24 ±0.12	0.08 ±0.08	0.05 ±0.02	0.11 ±0.06	10.94 ±9.62
Phvul.002G039100, Phvul.002G039166, Phvul.002G039232	Chalcone synthase	0.4 ±0.13	0.52 ±0.24	0.67 ±0.2	0.43 ±0.15	0.17 ±0.08	0.44 ±0.22	1.64 ±0.87	10.8 ±1.31
Phvul.002G040100	4-Coumaroyl: coenzyme A ligase 1	10.37 ±3.44	8.98 ±3.32	8.9 ±1	6.64 ±0.54	15.25 ±1.89	8.9 ±2.92	10.43 ±1.33	32.89 ±4.72
Phvul.002G044100	Diamine N-acetyltransferase / spermidine N(1)-acetyltransferase	1.04 ±0.27	1.87 ±0.51	1.34 ±0.43	2.58 ±0.64	0.98 ±0.1	1.51 ±0.41	2.95 ±0.78	13.23 ±3.3
Phvul.002G046100	Protein kinase domain // S-locus glycoprotein domain // D-mannose binding lectin	0.16 ±0.05	0.12 ±0.03	0.11 ±0.01	0.23 ±0.03	0.09 ±0.05	0.07 ±0.01	0.19 ±0.08	0.94 ±0.05
Phvul.002G046500	Protein kinase domain // S-locus glycoprotein domain // D-mannose binding lectin	0.22 ±0.02	0.23 ±0.11	0.16 ±0.01	0.56 ±0.34	0.16 ±0.01	0.18 ±0.04	0.46 ±0.14	2.84 ±0.64
Phvul.002G048100	Tryptophan synthase β chain-like protein	1.53 ±0.3	1.99 ±1.46	1.64 ±0.39	1.34 ±0.09	0.99 ±0.18	0.92 ±0.24	2.01 ±0.31	19.36 ±10.16
Phvul.002G075600	No functional annotation	0 ±0	0 ±0	0 ±0	0 ±0	0 ±0	0 ±0	0 ±0	0.61 ±0.33
Phvul.002G075900	No functional annotation	0.02 ±0.02	0 ±0	0.04 ±0.02	0.07 ±0.01	0 ±0	0.11 ±0.03	0.35 ±0.14	0.93 ±0.08
Phvul.002G076500	Neutrophil collagenase / Matrix metalloproteinase 8	0 ±0	0.02 ±0.02	0.03 ±0.03	0 ±0	0 ±0	0 ±0	0.13 ±0.06	3.37 ±0.37

Table S5 continued

		Transcript levels (FPKM)							
		Mock inoculation				*Xap* inoculation			
**Gene ID**	**Gene Annotation**	**0 h PI**	**8 h PI**	**24 h PI**	**48 h PI**	**0 h PI**	**8 h PI**	**24 h PI**	**48 h PI**
Phvul.002G076600	Matrilysin / Uterine metalloendopeptidase	0.2 ±0.08	0.02 ±0.02	0.56 ±0.4	0.29 ±0.16	0.09 ±0.05	0.13 ±0.09	0.44 ±0.25	5.64 ±0.88
Phvul.002G082300	Regulator of Vps4 activity in the MVB pathway protein	2.11 ±0.3	2.47 ±0.64	2.17 ±0.12	3.11 ±1.24	3.47 ±0.41	3.16 ±0.39	6.21 ±2.04	17.96 ±8.89
Phvul.002G083900	Calcium-binding protein CML30-related	0.27 ±0.06	0.3 ±0.09	0.37 ±0.14	0.08 ±0.01	0.2 ±0.08	0.4 ±0.08	0.5 ±0.18	3.82 ±0.67
Phvul.002G113000	Amino acid transporter	3.71 ±2.04	6.02 ±1.7	4.66 ±1.31	6.14 ±0.28	1.63 ±0.32	6.41 ±2.39	7.71 ±1.97	27.13 ±0.53
Phvul.002G144600	Cinnamyl alcohol dehydrogenase 6-related	0.21 ±0.09	0.17 ±0.12	0.24 ±0.12	0.3 ±0.17	0 ±0	0.05 ±0.03	0.16 ±0.07	4.41 ±1.04
Phvul.002G154600	MATE efflux family protein	0.01 ±0.01	0.01 ±0.01	0.02 ±0.02	0.14 ±0.14	0.01 ±0.01	0.04 ±0.01	0.17 ±0.12	1.09 ±0.62
Phvul.002G155500	Thaumatin family	172.86 ±40.76	185.61 ±21.54	133.5 ±9.89	165.9 ±29.15	162.42 ±39.58	233.18 ±13.23	176.62 ±35.67	941.24 ±138.86
Phvul.002G160600	Protein LURP-one-related 17	1.85 ±0.44	0.96 ±0.23	1.27 ±0	2.25 ±1.18	2.13 ±0.49	1.57 ±0.47	2.27 ±0.67	11.17 ±6.51
Phvul.002G180900	BON1-associated protein 1-related	0.03 ±0.03	0.03 ±0.03	0.22 ±0.17	0.25 ±0.13	0 ±0	0.19 ±0.1	0.39 ±0.2	6.29 ±0.76
Phvul.002G184300	Chalcone synthase	0.22 ±0.09	0.28 ±0.06	0.49 ±0.18	0.37 ±0.04	0.2 ±0.1	0.68 ±0.32	1.32 ±0.65	3.95 ±0.33
Phvul.002G188800	LYSM domain-containing GPI-anchored protein 2	9.97 ±0.54	10.16 ±3.52	8.05 ±0.06	9.97 ±0.59	11.34 ±0.72	11.06 ±1.19	12.56 ±1.65	45.08 ±5.1
Phvul.002G197800	Stress up-regulated Nod 19	3.14 ±1.09	3.23 ±0.9	3.89 ±0.53	4.54 ±0.44	1.67 ±0.38	4.09 ±1.45	4.7 ±0.88	26.44 ±9.34
Phvul.002G199800	Tetrahydroberberine oxidase	0.17 ±0.09	0.04 ±0.02	0.49 ±0.17	0.32 ±0.09	0 ±0	0.06 ±0.01	0.75 ±0.39	22.13 ±1.24
Phvul.002G209400	Pathogenesis-related protein Bet v I family	1.23 ±0.57	1.7 ±0.3	3.24 ±1.24	1.86 ±0.84	0.74 ±0.14	1.49 ±0.6	5.01 ±1.6	56.05 ±1.95

Table S5 continued

		Transcript levels (FPKM)							
		Mock inoculation				*Xap* inoculation			
**Gene ID**	**Gene Annotation**	**0 h PI**	**8 h PI**	**24 h PI**	**48 h PI**	**0 h PI**	**8 h PI**	**24 h PI**	**48 h PI**
Phvul.002G209500	Pathogenesis-related protein Bet v I family	3.42 ±1.7	3.55 ±0.1	11.8 ±2.73	9.59 ±2.36	1.46 ±0.21	5.99 ±2.06	16.15 ±2.88	167.31 ±10.04
Phvul.002G209600	Protein of unknown function	0.19 ±0.02	0.29 ±0.09	0.15 ±0.03	0.15 ±0.04	0.44 ±0.12	0.31 ±0.09	0.27 ±0.05	1.42 ±1.03
Phvul.002G215000	Legume lectin domain / Protein tyrosine kinase	0.18 ±0.03	0.17 ±0.03	0.26 ±0.05	0.25 ±0.1	0.13 ±0.04	0.16 ±0.06	0.53 ±0.35	3.3 ±1.62
Phvul.002G215100	Protein tyrosine kinase / Leucine rich repeat N-terminal domain (LRRNT_2)	0.06 ±0.02	0.05 ±0.02	0.11 ±0.03	0.16 ±0.02	0.02 ±0.01	0.06 ±0.01	0.15 ±0.04	1.2 ±0.29
Phvul.002G217900	Protein kinase domain // Leucine rich repeat N-terminal domain (LRRNT_2)	0.08 ±0.05	0.1 ±0.03	0.1 ±0.08	0.36 ±0.11	0.02 ±0.02	0.04 ±0.02	0.18 ±0.08	1.6 ±0.12
Phvul.002G219300	(3S,6E)-Nerolidol synthase	1.85 ±0.16	2.68 ±1.51	2.66 ±0.78	2.27 ±0.56	1.72 ±0.43	1.32 ±0.33	3.22 ±0.7	10.74 ±3.27
Phvul.002G223400	NADH: ubiquinone reductase (non-electrogenic)	0.41 ±0.09	0.36 ±0.15	0.41 ±0.03	0.4 ±0.08	0.39 ±0.08	0.4 ±0.08	1.1 ±0.36	2.78 ±0.65
Phvul.002G239400	Leucine-rich repeat receptor-like protein kinase	1.3 ±0.05	1.44 ±0.26	1.25 ±0.09	1.31 ±0.08	1.28 ±0.32	1.66 ±0.06	1.63 ±0.33	5.26 ±1.56
Phvul.002G258600	Copine (calcium-dependent phospholipid-binding protein) family protein	2.63 ±0.13	3.46 ±0.66	1.74 ±0.07	2.51 ±0.23	2.46 ±0.25	2.92 ±0.28	3.63 ±0.49	11.14 ±2.65
Phvul.002G259900	Protein of unknown function	0.39 ±0.06	0.31 ±0.1	0.22 ±0.05	0.29 ±0.08	0.17 ±0.08	0.48 ±0.13	0.28 ±0.05	1.48 ±0.42
Phvul.002G265400	WRKY transcription factor 50-related	3.76 ±0.21	4.89 ±1.3	3.48 ±0.34	5.48 ±1.73	5.71 ±0.47	5.03 ±0.85	7.63 ±3.24	38.57 ±11.44
Phvul.002G268100	No functional annotation	0.69 ±0.27	0.78 ±0.28	0.32 ±0.13	0.89 ±0.39	1.23 ±0.26	0.9 ±0.35	0.7 ±0.27	4.61 ±2.73

Table S5 continued

		Transcript levels (FPKM)							
		Mock inoculation				*Xap* inoculation			
**Gene ID**	**Gene Annotation**	**0 h PI**	**8 h PI**	**24 h PI**	**48 h PI**	**0 h PI**	**8 h PI**	**24 h PI**	**48 h PI**
Phvul.002G269300	Histone H3	8.51 ±1.66	10.18 ±1.74	3.91 ±0.06	2.93 ±0.73	10.44 ±2.1	13.72 ±0.42	5 ±0.98	15.03 ±2.7
Phvul.002G271900	No functional annotation	0.07 ±0.03	0.03 ±0.03	0.03 ±0.02	0 ±0	0 ±0	0 ±0	0 ±0	1.24 ±0.93
Phvul.002G285800	WRKY transcription factor 45-related	0.15 ±0.09	0.13 ±0.01	0.63 ±0.27	1.32 ±0.43	0.12 ±0.13	0.18 ±0.1	1.52 ±0.88	13.62 ±7.11
Phvul.002G286500, Phvul.002G286600	Thaumatin family	36.94 ±5.61	40.16 ±14.23	20.53 ±3.79	17.93 ±3.06	42.08 ±7.34	48.86 ±8.22	22.22 ±2.92	121.64 ±6.39
Phvul.002G317000	MYB-like DNA-binding protein	0.23 ±0.08	0.8 ±0.43	1.42 ±0.47	1.42 ±0.63	0.22 ±0.12	0.51 ±0.11	1.92 ±0.72	17.6 ±5.65
Phvul.002G318000	Core-2/I-branching β-1,6-N-acetylglucosaminyltransferase family protein	1 ±0.17	0.93 ±0.08	0.8 ±0.05	0.71 ±0.2	0.86 ±0.27	1.26 ±0.2	1.03 ±0.06	3.04 ±0.51
Phvul.002G318100	Protein SAR deficient 1	3.62 ±0.16	4.43 ±1.45	2.44 ±0.16	3.36 ±1.02	4.28 ±0.51	4.24 ±0.46	5.91 ±1.46	31.67 ±13.13
Phvul.002G318200	Protein tyrosine kinase // Wall-associated receptor kinase C-terminal	1 ±0.06	0.99 ±0.05	0.79 ±0.09	0.83 ±0.19	0.77 ±0.13	0.88 ±0.2	1.03 ±0.17	3.63 ±1.21
Phvul.002G326600	Aminocyclopropane-carboxylate oxidase	6.96 ±1.99	8.24 ±3.67	5.47 ±1.17	8.04 ±3.48	8.25 ±1.63	7.07 ±1.65	12.12 ±4.17	77.29 ±30.27
Phvul.002G329200	RAS-related protein RABA6A-related	0.48 ±0.16	0.27 ±0.08	0.55 ±0.14	0.38 ±0.08	0.47 ±0.12	0.35 ±0.02	0.54 ±0.06	2.15 ±1.1
Phvul.003G012800	AR781	0.2 ±0.06	0.15 ±0.05	0.37 ±0.17	0.33 ±0.18	0.06 ±0.03	0.15 ±0.08	0.37 ±0.17	2.19 ±0.08
Phvul.003G019800	Nudix hydrolase 17, mitochondrial-related	0 ±0	0 ±0	0 ±0	0 ±0	0 ±0	0 ±0	0.05 ±0.05	0.61 ±0.44
Phvul.003G022400	Copper transport protein ATOX1-related	0.3 ±0.08	0.99 ±0.31	0.51 ±0.11	0.56 ±0.17	0.25 ±0.08	0.66 ±0.12	0.74 ±0.32	3.63 ±0.75

Table S5 continued

		Transcript levels (FPKM)							
		Mock inoculation				*Xap* inoculation			
**Gene ID**	**Gene Annotation**	**0 h PI**	**8 h PI**	**24 h PI**	**48 h PI**	**0 h PI**	**8 h PI**	**24 h PI**	**48 h PI**
Phvul.003G022500	No functional annotation	8.75 ±1.1	11.88 ±5.05	5 ±0.93	6.72 ±0.49	7.34 ±2.29	8.85 ±1.07	8.42 ±1.26	43.1 ±8.5
Phvul.003G024200	Phospholipid-transporting ATPase 10-related	0.37 ±0.09	0.37 ±0.14	0.46 ±0.07	0.57 ±0.05	0.39 ±0.03	0.37 ±0.05	0.58 ±0.09	3.93 ±0.32
Phvul.003G029400	No functional annotation	0.41 ±0.19	0.23 ±0.18	0.45 ±0.09	0.39 ±0.15	0.09 ±0.02	0.13 ±0.02	0.28 ±0.09	1.7 ±0.96
Phvul.003G034000	E3 ubiquitin-protein ligase RHA2	1.11 ±0.38	1.45 ±0.17	0.59 ±0.23	0.99 ±0.2	0.97 ±0.29	0.27 ±0.14	1.28 ±0.64	5.57 ±2.96
Phvul.003G051900	Auxin response factor 30-related	0.06 ±0.06	0.15 ±0.15	0 ±0	0 ±0	0 ±0	0.42 ±0.36	0.46 ±0.25	1.55 ±0.43
Phvul.003G074000	Isoflavone synthase	0.05 ±0.03	0.19 ±0.03	0.42 ±0.21	0.18 ±0.03	0.1 ±0.03	0.21 ±0.1	0.67 ±0.45	1.23 ±0.18
Phvul.003G079800	Adenylyl-sulfate reductase / Thioredoxin-dependent 5'-adenylylsulfate reductase	0.16 ±0.02	0.16 ±0.04	0.24 ±0.14	0.13 ±0.05	0.29 ±0.12	0.13 ±0.02	0.21 ±0.04	0.96 ±0.48
Phvul.003G088000	Adenine nucleotide α-hydrolases-like superfamily protein	0.09 ±0.02	0.23 ±0.11	0.35 ±0.17	0.22 ±0.08	0.12 ±0.09	0.15 ±0.06	0.28 ±0.09	2.23 ±0.29
Phvul.003G096700	Late embryogenesis abundant 3 family protein	3.13 ±0.21	1.72 ±0.44	7.96 ±2	9.41 ±2.88	2.31 ±0.96	1.68 ±0.18	8.23 ±2.04	89.58 ±60.55
Phvul.003G104400	C2 domain-containing protein-related	14.7 ±9.62	25.73 ±10.15	4.23 ±0.46	9.62 ±2.52	5.04 ±0.86	23.55 ±8.86	11.04 ±2.88	38.95 ±5.38
Phvul.003G107100	E3 ubiquitin-protein ligase ATL41-related	1.13 ±0.32	0.95 ±0.25	1.18 ±0.26	1.46 ±0.14	1.32 ±0.13	1.4 ±0.5	1.19 ±0.35	7.57 ±2.36
Phvul.003G109000	Pathogenesis-related protein Bet V I family	0.51 ±0.12	0.75 ±0.11	1.2 ±0.62	1.24 ±0.38	0.46 ±0.09	1.27 ±0.36	3.2 ±1.65	27.88 ±5.69
Phvul.003G109200	Pathogenesis-related protein Bet V I family	0.49 ±0.48	0.9 ±0.31	0.91 ±0.38	0.73 ±0.11	0.07 ±0.03	0.69 ±0.34	1.46 ±0.76	12.18 ±0.92

Table S5 continued

		Transcript levels (FPKM)							
		Mock inoculation				*Xap* inoculation			
**Gene ID**	**Gene Annotation**	**0 h PI**	**8 h PI**	**24 h PI**	**48 h PI**	**0 h PI**	**8 h PI**	**24 h PI**	**48 h PI**
Phvul.003G109300	Pathogenesis-related protein Bet V I family	0 ±0	0.06 ±0.06	0 ±0	0 ±0	0 ±0	0.04 ±0.03	0.02 ±0.02	0.78 ±0.09
Phvul.003G109600	Pathogenesis-related protein Bet V I family	0.1 ±0.05	0.08 ±0.05	0.37 ±0.2	0.2 ±0.11	0 ±0	0.16 ±0.08	0.36 ±0.18	4.18 ±0.33
Phvul.003G109602	Pathogenesis-related protein Bet V I family	0.06 ±0.06	0.08 ±0.04	0.25 ±0.07	0.22 ±0.12	0 ±0	0.14 ±0.08	0.32 ±0.17	5.29 ±0.15
Phvul.003G109603	Pathogenesis-related protein Bet V I family	0.28 ±0.22	0.18 ±0.14	0.14 ±0.08	0.22 ±0.08	0 ±0	0.22 ±0.11	0.43 ±0.23	6.07 ±0.24
Phvul.003G109800	Pathogenesis-related protein Bet V I family	0.03 ±0.03	0.02 ±0.02	0.21 ±0.1	0.19 ±0.1	0 ±0	0.03 ±0.03	0.43 ±0.16	3.8 ±0.47
Phvul.003G126300	Saccharopine dehydrogenase ($NADP^{(+)}$, L-lysine-forming) / Lysine-ketoglutarate reductase	0.69 ±0.08	0.74 ±0.09	0.9 ±0.08	1.52 ±0.19	0.58 ±0.16	0.88 ±0.1	1.34 ±0.34	8.44 ±3.79
Phvul.003G129200	Protein TIFY 10a-related	0.49 ±0.21	0.5 ±0.23	0.92 ±0.11	0.85 ±0.31	0.95 ±0.2	0.76 ±0.25	1.12 ±0.28	3.7 ±1.57
Phvul.003G136400	Gulonolactone oxidase	0.08 ±0.03	0.08 ±0.07	0.12 ±0.04	0.11 ±0.05	0.1 ±0.05	0.1 ±0.06	0.27 ±0.1	2.13 ±0.43
Phvul.003G140800	Helix-loop-helix DNA-binding domain	1.95 ±0.27	3.42 ±0.68	2.42 ±0.06	2.25 ±0.29	2.46 ±0.29	2.31 ±0.18	4.86 ±1.29	9.64 ±2.44
Phvul.003G146700	β-1,3-N-acetylglucosaminyltransferase	0.21 ±0.04	0.1 ±0.02	0.18 ±0.08	0.85 ±0.43	0.45 ±0.12	0.38 ±0.18	1.13 ±0.31	3.48 ±1.23
Phvul.003G154800	Heat shock 70 kDa protein 5	1.42 ±0.62	0.68 ±0.12	5.52 ±1.28	4.69 ±1.69	2.13 ±0.18	0.99 ±0.55	4.69 ±0.55	32.56 ±19.83
Phvul.003G160900	No functional annotation	1.53 ±0.45	1.91 ±0.53	1.01 ±0.13	3.58 ±2.37	0.75 ±0.12	1.84 ±0.42	3.7 ±1.64	20.72 ±11.53
Phvul.003G166700, Phvul.003G166800	Blue copper protein	3.39 ±0.16	4.14 ±0.16	2.97 ±0.3	3.16 ±0.51	2.91 ±0.19	5.02 ±0.69	5.34 ±0.98	19.19 ±5.14

Table S5 continued

		Transcript levels (FPKM)							
		Mock inoculation				*Xap* inoculation			
**Gene ID**	**Gene Annotation**	**0 h PI**	**8 h PI**	**24 h PI**	**48 h PI**	**0 h PI**	**8 h PI**	**24 h PI**	**48 h PI**
Phvul.003G182600	Adenine nucleotide α-hydrolases-like protein-related	0.31 ±0.11	0.38 ±0.03	0.37 ±0.08	0.46 ±0.06	0.1 ±0.06	0.31 ±0.05	0.73 ±0.24	2.5 ±1.89
Phvul.003G184200	Ninja-family protein AFP1-related	0.06 ±0.03	0.39 ±0.26	0.21 ±0.02	0.22 ±0.09	0.09 ±0.09	0.04 ±0.04	0.29 ±0.17	3.27 ±1.56
Phvul.003G187200	Protein kinase domain // Leucine rich repeat (LRR_1) // Leucine rich repeat N-terminal domain (LRRNT_2) // Leucine rich repeat (LRR_8)	0.16 ±0.01	0.21 ±0.05	0.2 ±0.06	0.2 ±0.09	0.22 ±0.07	0.24 ±0.02	0.39 ±0.12	1.83 ±0.57
Phvul.003G209101	Malate synthase / Malic-condensing enzyme	0.08 ±0.05	0.2 ±0.07	0.39 ±0.16	0.78 ±0.53	0.01 ±0.02	0.11 ±0.07	1.1 ±0.61	13.73 ±9.05
Phvul.003G212600	MATE efflux family protein	0.09 ±0.02	0.16 ±0.08	0.12 ±0.04	0.21 ±0.18	0.06 ±0.06	0.22 ±0.11	0.45 ±0.27	1.87 ±0.94
Phvul.003G217100	Probable lipid transfer (LTP_2)	61.06 ±3.62	64.15 ±7.3	25.41 ±2.92	20.88 ±2.46	68.14 ±6.93	69.47 ±2.25	37.52 ±5.91	99.28 ±12.11
Phvul.003G237400	Expressed protein	28.25 ±15.35	9.09 ±7.95	26.34 ±8.13	21.15 ±9.94	31.69 ±4.55	11.07 ±10.4	21.35 ±1.99	95.47 ±56.71
Phvul.003G238700	Auxin responsive GH3 gene family	0.36 ±0.22	0.47 ±0.21	0.66 ±0.17	1.06 ±0.17	0.09 ±0.03	0.41 ±0.17	1.48 ±0.85	11.47 ±1.77
Phvul.003G239800	No functional annotation	1.03 ±0.58	0.66 ±0.33	0.93 ±0.35	1.63 ±0.91	0.46 ±0.32	0.65 ±0.27	2.09 ±0.58	32.86 ±9.66
Phvul.003G247500	Leucine-rich repeat-containing protein	0.06 ±0.02	0.01 ±0.01	0.18 ±0.06	0.12 ±0.05	0.02 ±0.02	0.04 ±0.02	0.19 ±0.12	9.3 ±1.4
Phvul.003G249400	No functional annotation	4.47 ±1.72	3.92 ±1.44	1.63 ±0.31	0.55 ±0.28	5.59 ±1.07	3.33 ±1.1	1.21 ±0.1	2.72 ±1.56
Phvul.003G251000	Calcium-binding protein CML41-related	3.4 ±0.14	5.27 ±1.32	5.04 ±0.77	4.71 ±0.21	2.74 ±0.56	2.89 ±0.28	4.83 ±0.18	18.85 ±1.92

Table S5 continued

		Transcript levels (FPKM)							
		Mock inoculation				*Xap* inoculation			
**Gene ID**	**Gene Annotation**	**0 h PI**	**8 h PI**	**24 h PI**	**48 h PI**	**0 h PI**	**8 h PI**	**24 h PI**	**48 h PI**
Phvul.003G255300	No functional annotation	0 ±0	0 ±0	0 ±0	0 ±0	0 ±0	0 ±0	0.12 ±0.08	1.29 ±0.45
Phvul.003G256600	BCS1 AAA-type ATPase	4.93 ±0.88	4.58 ±2.26	2.56 ±0.42	3.96 ±0.69	4.81 ±0.43	4 ±0.69	4.45 ±0.96	20.16 ±7.82
Phvul.003G272900	(-)-Lariciresinol reductase	2.75 ±0.37	3.8 ±0.65	2.46 ±0.22	2.75 ±0.42	2.34 ±0.18	4.02 ±1.17	4.45 ±0.99	12.89 ±1.99
Phvul.003G287400, *Phvul.003G287500*	Alcohol dehydrogenase related	10.63 ±4.35	8.63 ±5.7	8.51 ±1.26	6.6 ±0.54	11.58 ±1.15	4.87 ±2.32	9.8 ±1.39	39.19 ±21.39
Phvul.004G018900	α-Dioxygenase (DOX1)	0.12 ±0.04	0.1 ±0.05	0.77 ±0.33	0.31 ±0.09	0.01 ±0.01	0.04 ±0.03	0.68 ±0.57	2.52 ±1.05
Phvul.004G044800, *Phvul.004G044900*	Leucine rich repeat (LRR_1) // Leucine rich repeat N-terminal domain (LRRNT_2) // Leucine rich repeat (LRR_8)	0.15 ±0.03	0.22 ±0.04	0.19 ±0.02	0.14 ±0.05	0.11 ±0.03	0.15 ±0.02	0.35 ±0.01	0.75 ±0.27
Phvul.004G056700	Amino acid transporter	0.63 ±0.18	0.46 ±0.16	0.75 ±0.07	0.61 ±0.06	0.63 ±0.21	0.5 ±0.12	0.93 ±0.32	9.55 ±1.47
Phvul.004G071700	MLO-like protein 12-related	0.14 ±0.08	0.14 ±0.04	0.25 ±0.11	0.35 ±0.05	0.15 ±0.02	0.15 ±0.05	0.46 ±0.12	2.46 ±0.34
Phvul.004G077400	No apical meristem (NAM) protein	0.12 ±0.07	0.21 ±0.1	0.23 ±0.05	0.75 ±0.16	0.07 ±0.03	0.41 ±0.23	0.53 ±0.1	4.72 ±2
Phvul.004G088400	Alliin lyase / L-cysteine sulfoxide lyase	0.46 ±0.16	0.29 ±0.08	1.05 ±0.55	0.54 ±0.21	0.03 ±0.03	0.17 ±0.07	0.98 ±0.33	29.28 ±2.25
Phvul.004G092100	Ethylene-responsive transcription factor 15-related	0.27 ±0.15	0.36 ±0.09	0.45 ±0.17	0.44 ±0.14	0.07 ±0.04	0.61 ±0.33	0.83 ±0.32	2.85 ±1.36

Table S5 continued

		Transcript levels (FPKM)							
		Mock inoculation				*Xap* inoculation			
**Gene ID**	**Gene Annotation**	**0 h PI**	**8 h PI**	**24 h PI**	**48 h PI**	**0 h PI**	**8 h PI**	**24 h PI**	**48 h PI**
Phvul.004G099100	Leucine rich repeat (LRR_1) // Leucine rich repeat N-terminal domain (LRRNT_2) // Leucine rich repeat (LRR_8)	1.35 ±0.58	1.85 ±0.46	0.68 ±0.09	1.26 ±0.08	0.78 ±0.24	1.45 ±0.5	1.28 ±0.22	9.37 ±2.55
Phvul.004G103900	Isoflavone 7-*O*-glucosyltransferase	0.34 ±0.12	0.38 ±0.13	0.54 ±0.03	0.26 ±0.02	0.47 ±0.17	0.41 ±0.06	0.69 ±0.36	1.32 ±0.27
Phvul.004G107200	Copper transport family protein-related	8.8 ±0.7	8.99 ±3.99	3.68 ±0.63	5.64 ±1.21	12.74 ±2.11	7.37 ±0.54	7.98 ±1.25	30.51 ±16.6
Phvul.004G115600	Leucine rich repeat (LRR_1) // Leucine rich repeat (LRR_8)	0.22 ±0.04	0.28 ±0.07	0.23 ±0.06	0.22 ±0.04	0.07 ±0.02	0.38 ±0.18	0.23 ±0.03	1.34 ±0.03
Phvul.004G129300, *Phvul.004G129400*	PPR repeat (PPR) // PPR repeat (PPR_1) // PPR repeat family (PPR_2) // Pentatricopeptide repeat domain (PPR_3)	0.48 ±0.33	0.98 ±0.31	0.82 ±0.14	0.33 ±0.12	0.47 ±0.2	0.98 ±0.39	0.69 ±0.15	1.4 ±0.62
Phvul.004G141200	ADP-ribosylation factor GTPase-activating protein AGD15-related	0.29 ±0.08	0.26 ±0.08	0.27 ±0.06	0.53 ±0.18	0.54 ±0.15	0.34 ±0.07	1.04 ±0.32	2.44 ±0.66
Phvul.004G153056	No functional annotation	0.73 ±0.73	1.48 ±0.77	3.89 ±1.47	2.03 ±1.22	0.31 ±0.31	0.64 ±0.64	7.1 ±2.15	53.29 ±6.4
Phvul.004G155900	Protein SAR deficient 1	0.13 ±0.05	0.2 ±0.08	0.1 ±0.01	0.28 ±0.07	0.08 ±0.08	0.17 ±0.09	0.31 ±0.04	1.2 ±0.17
Phvul.004G157900	Protein NIM1-interacting 2	2.89 ±0.99	3.79 ±0.92	0.88 ±0.08	0.76 ±0.17	1.16 ±0.32	3.04 ±0.87	0.77 ±0.25	3.12 ±0.31
Phvul.005G011100	Phospholipid-transporting ATPase	0.1 ±0.03	0.09 ±0.02	0.28 ±0.05	0.25 ±0.05	0.09 ±0.03	0.06 ±0.01	0.39 ±0.16	1.59 ±0.17

Table S5 continued

		Transcript levels (FPKM)							
		Mock inoculation				*Xap* inoculation			
**Gene ID**	**Gene Annotation**	**0 h PI**	**8 h PI**	**24 h PI**	**48 h PI**	**0 h PI**	**8 h PI**	**24 h PI**	**48 h PI**
Phvul.005G024800	VQ motif	1.83 ±0.15	2.33 ±0.59	1.57 ±0.09	2.3 ±1.76	2.27 ±0.42	2.97 ±0.95	3.38 ±0.32	13.41 ±3.57
Phvul.005G042200	No functional annotation	0.33 ±0.18	0.03 ±0.03	0.4 ±0.14	0.54 ±0.46	0 ±0	0.07 ±0.04	0.57 ±0.29	5.27 ±2.15
Phvul.005G057200	Expressed protein	0.82 ±0.42	0.54 ±0.43	0.6 ±0.31	1.31 ±0.89	1.12 ±0.24	1 ±0.17	2.18 ±0.96	17.1 ±11.51
Phvul.005G077501	Oxidoreductase, 2-oxoglutarate-Fe II oxygenase family protein	0.43 ±0.13	0.23 ±0.05	0.78 ±0.13	0.54 ±0.16	0.35 ±0.06	0.25 ±0.1	1.05 ±0.55	3.18 ±1.05
Phvul.005G097500	E3 ubiquitin-protein ligase ATL41	0.58 ±0.2	1.16 ±0.3	0.4 ±0.25	0.65 ±0.19	0.31 ±0.07	0.9 ±0.29	0.89 ±0.21	2.69 ±0.09
Phvul.005G109000	Transferase family	1.35 ±0.13	1.57 ±0.72	0.9 ±0.24	1.57 ±0.46	1.19 ±0.18	0.95 ±0.2	1.73 ±0.44	19.43 ±4.61
Phvul.005G111500	Geranyl diphosphate diphosphatase	0.46 ±0.26	0.29 ±0.21	0.61 ±0.12	0.3 ±0.12	0.6 ±0.14	0.16 ±0.09	0.71 ±0.32	1.59 ±0.72
Phvul.005G136700	MLO protein	0.11 ±0.06	0.19 ±0.03	0.08 ±0.02	0.2 ±0.02	0.14 ±0.08	0.14 ±0.1	0.1 ±0.03	1.49 ±0.18
Phvul.005G137300	LOB domain-containing protein 1-related	0.18 ±0.02	0.29 ±0.06	0.63 ±0.1	0.36 ±0.04	0.16 ±0.06	0.23 ±0.11	0.45 ±0.09	1.66 ±0.12
Phvul.005G155800	Chitinase-related	10.85 ±1.26	10.96 ±3.19	11.57 ±0.56	13.63 ±1.16	12 ±1.04	12.99 ±0.7	19.76 ±1.98	119.1 ±11.06
Phvul.005G158500	β-Fructofuranosidase, insoluble isoenzyme CWINV1-related	1.15 ±0.55	1.28 ±0.15	2.16 ±0.34	2.6 ±0.65	0.66 ±0.03	1.49 ±0.59	2.55 ±0.32	11.17 ±0.72
Phvul.005G166500	ABC transporter G family member 29	0.12 ±0.03	0.17 ±0.06	0.2 ±0.05	0.34 ±0.11	0.16 ±0.01	0.18 ±0.02	0.42 ±0.07	3.87 ±0.32

Table S5 continued

		Transcript levels (FPKM)							
		Mock inoculation				*Xap* inoculation			
**Gene ID**	**Gene Annotation**	**0 h PI**	**8 h PI**	**24 h PI**	**48 h PI**	**0 h PI**	**8 h PI**	**24 h PI**	**48 h PI**
Phvul.005G171900	U5 SNRNP-specific protein-like factor and related proteins // G protein β subunit-like protein	2.14 ±0.5	2.53 ±1.43	0.52 ±0.08	0.73 ±0.29	3.01 ±0.8	2.22 ±0.55	1.09 ±0.33	14.86 ±4.66
Phvul.005G173000	Auxin-responsive protein IAA15	0.56 ±0.04	0.57 ±0.19	0.86 ±0.16	0.8 ±0.42	0.65 ±0.12	0.6 ±0.16	0.89 ±0.09	3.96 ±3.04
Phvul.005G173600	Sulfate-transporting ATPase	1.13 ±0.25	1.51 ±0.39	1.93 ±0.78	1.58 ±0.23	0.81 ±0.14	1.7 ±0.43	2.76 ±0.98	17.38 ±2.49
Phvul.006G002700	Genomic DNA, chromosome 3, P1 clone: MKA23	1.92 ±0.43	2.18 ±0.51	1.96 ±0.24	4.21 ±2.07	0.72 ±0.15	1.89 ±0.32	4.46 ±1.05	17.43 ±3.6
Phvul.006G020700	Interleukin-1 receptor-associated kinase 4	1.03 ±0.1	1.11 ±0.23	0.93 ±0.21	1.98 ±0.78	0.9 ±0.24	1.11 ±0.21	2.72 ±1	8.21 ±0.95
Phvul.006G022800	Aspartyl protease-like protein	0.6 ±0.14	0.43 ±0.09	0.43 ±0.08	1.3 ±0.83	1.15 ±0.18	0.56 ±0.16	1.61 ±0.54	6.01 ±2.79
Phvul.006G058700	X-box transcription factor-related	0.1 ±0.06	0.12 ±0.03	0.13 ±0.05	0.12 ±0.05	0.08 ±0.02	0.18 ±0.08	0.22 ±0.08	1.12 ±0.06
Phvul.006G074600	WRKY transcription factor 33	0.08 ±0.02	0.11 ±0.05	0.18 ±0.1	0.09 ±0.02	0.07 ±0.03	0.04 ±0.03	0.14 ±0.08	2.68 ±0.13
Phvul.006G078300	No functional annotation	0.27 ±0.14	0.26 ±0.14	1.1 ±0.61	0.84 ±0.58	0 ±0	0.71 ±0.43	1.35 ±0.73	50.32 ±1.95
Phvul.006G079500	Oxalate-CoA ligase / Oxalyl-CoA synthetase	0.03 ±0.03	0.01 ±0.01	0.1 ±0.06	0 ±0	0.01 ±0.01	0.05 ±0.02	0.22 ±0.13	0.91 ±0.53
Phvul.006G079700	Trans-cinnamate 4-monooxygenase / Cinnamic acid 4-monooxygenase	0.18 ±0.07	0.2 ±0.06	0.26 ±0.14	0.32 ±0.24	0.25 ±0.06	0.24 ±0.09	0.59 ±0.16	4.18 ±0.49
Phvul.006G085200	P-loop containing nucleoside triphosphate hydrolases superfamily protein	0.81 ±0.25	0.68 ±0.48	0.58 ±0.06	0.51 ±0.07	0.98 ±0.1	0.48 ±0.27	0.49 ±0.08	3.69 ±0.8

Table S5 continued

		Transcript levels (FPKM)							
		Mock inoculation				*Xap* inoculation			
**Gene ID**	**Gene Annotation**	**0 h PI**	**8 h PI**	**24 h PI**	**48 h PI**	**0 h PI**	**8 h PI**	**24 h PI**	**48 h PI**
Phvul.006G086100	AMP-binding enzyme	3.5 ±0.68	5.15 ±0.95	2.01 ±0.22	2.23 ±0.62	2.74 ±0.64	4.98 ±0.66	3.61 ±0.82	18.34 ±7.39
Phvul.006G086200, Phvul.006G086211	No functional annotation	0.82 ±0.28	1.06 ±0.44	0.62 ±0.03	1.25 ±0.46	0.69 ±0.21	1 ±0.25	1.33 ±0.27	6.04 ±1.98
Phvul.006G090200	Respiratory burst oxidase homolog protein B	0.83 ±0.18	0.8 ±0.06	0.76 ±0.15	1.21 ±0.43	1.36 ±0.09	1.44 ±0.22	2.34 ±0.81	6.96 ±3.08
Phvul.006G102200	Hevein-like preproprotein	21.03 ±4.55	28.96 ±4.85	14.63 ±2.89	21.22 ±2.73	16.78 ±2.63	28.09 ±5.79	21.47 ±5.8	127.81 ±17.59
Phvul.006G102300	Hevein-like preproprotein	91.09 ±11.4	92.58 ±21.55	91.71 ±5.23	84.31 ±10.74	81.62 ±18.82	94.07 ±11.93	99.37 ±14.05	513.25 ±9.39
Phvul.006G129500	Peroxidase 22-related	0.4 ±0.31	0.41 ±0.16	1.37 ±0.46	0.87 ±0.04	0.21 ±0.05	0.15 ±0.09	1.79 ±0.53	53.74 ±4.05
Phvul.006G135600	Copper transport protein, ATOX1-related	1.34 ±0.31	1.64 ±1.17	0.54 ±0.13	0.75 ±0.2	1.28 ±0.35	1.02 ±0.35	0.92 ±0.31	11.97 ±6.18
Phvul.006G142300	Auxin-induced in root cultures protein 12	4.62 ±0.53	4.62 ±0.66	4.19 ±0.63	6.16 ±1.74	3.55 ±0.4	5.71 ±0.78	7.83 ±1.73	29.32 ±6.52
Phvul.006G154700	Epididymal membrane protein E9-related	0.73 ±0.1	0.6 ±0.03	1.53 ±0.35	2.53 ±0.43	0.85 ±0.18	0.85 ±0.11	2.58 ±0.73	14.55 ±2.3
Phvul.006G173000	No functional annotation	0.78 ±0.24	1.09 ±0.33	0.37 ±0.13	1 ±0.58	0.21 ±0.11	1.62 ±0.48	1.3 ±0.46	8.35 ±3.12
Phvul.006G178800	E3 ubiquitin-protein ligase ATL41	2.33 ±0.1	2.12 ±0.21	2.6 ±0.37	2.61 ±0.72	2.13 ±0.49	2.28 ±0.06	4.13 ±0.55	11.9 ±1.69
Phvul.006G181300	Glutaredoxin-C9	0.88 ±0.32	0.78 ±0.21	0.56 ±0.06	1.42 ±1.1	0.46 ±0.15	1.4 ±0.07	2.22 ±0.37	12.78 ±6.21
Phvul.006G185300	Lipoxygenase	0.19 ±0.14	0.43 ±0.09	0.11 ±0.05	0.05 ±0.01	0.11 ±0.04	0.3 ±0.08	0.13 ±0.08	2.94 ±2.41
Phvul.006G188900	No apical meristem (NAM) protein	0.02 ±0.02	0.05 ±0.02	0.23 ±0.12	0.27 ±0.2	0.02 ±0.02	0.07 ±0.05	0.13 ±0.05	2 ±1.37

Table S5 continued

		Transcript levels (FPKM)							
		Mock inoculation				*Xap* inoculation			
**Gene ID**	**Gene Annotation**	**0 h PI**	**8 h PI**	**24 h PI**	**48 h PI**	**0 h PI**	**8 h PI**	**24 h PI**	**48 h PI**
Phvul.006G192400	EF hand (EF-hand_1) / Pyridine nucleotide-disulphide oxidoreductase	0.31 ±0.1	0.32 ±0.09	0.47 ±0.1	0.43 ±0.06	0.24 ±0.08	0.39 ±0.1	0.73 ±0.22	1.77 ±0.16
Phvul.006G194200	β-Amyrin synthase (LUP4)	1.11 ±0.16	0.68 ±0.19	0.61 ±0.21	0.25 ±0.13	0.8 ±0.06	0.52 ±0.06	0.58 ±0.07	1.17 ±0.97
Phvul.006G194600	Receptor-like serine/ Threonine-protein kinase SD1-6-related	0.18 ±0.08	0.26 ±0.09	0.28 ±0.02	0.26 ±0.08	0.23 ±0.03	0.33 ±0.06	0.42 ±0.13	2.06 ±0.38
Phvul.006G195600, Phvul.006G195700	*S*-Linalool synthase	5.9 ±1.06	7.77 ±1.41	1.35 ±0.28	0.78 ±0.12	10.4 ±0.63	9.15 ±1.04	2.57 ±1.33	7.22 ±3.2
Phvul.006G198200	Protein kinase domain // Leucine rich repeat N-terminal domain (LRRNT_2) // Leucine rich repeat (LRR_8)	0.58 ±0.16	0.86 ±0.14	0.81 ±0.08	0.84 ±0.05	0.53 ±0.06	0.94 ±0.05	1.08 ±0.17	3.42 ±0.57
Phvul.007G008600	Chalcone isomerase	6.74 ±1.26	7.98 ±1.57	7.3 ±1.5	7.39 ±0.35	7.66 ±1.29	10.73 ±1.64	14.37 ±5.18	74.24 ±2.89
Phvul.007G016100	EamA-like transporter family	0.17 ±0.05	0.25 ±0.12	0.16 ±0.06	0.26 ±0.08	0.14 ±0.12	0.12 ±0.04	0.33 ±0.03	1.32 ±0.65
Phvul.007G022600	Replication factor A3	2.16 ±0.92	3.81 ±0.65	1.98 ±0.29	0.94 ±0.54	2.31 ±0.38	4.11 ±0.62	1.94 ±0.14	3.77 ±0.4
Phvul.007G024900	Monocarboxylate transporter	0.08 ±0.04	0.23 ±0.03	0.36 ±0.08	0.36 ±0.09	0.05 ±0.03	0.3 ±0.13	0.44 ±0.1	6.22 ±0.38
Phvul.007G026700	Genomic DNA, chromosome 3, P1 clone: MPE11	4.22 ±2.18	5.52 ±1.56	1.89 ±0.2	3.45 ±1.15	2.03 ±0.28	5.31 ±1.79	4.06 ±1.15	16.51 ±7.4
Phvul.007G040900	Peptidase of plants and bacteria	37.05 ±3.99	35.39 ±11.93	27.25 ±1.55	33.58 ±9.83	40.3 ±8.69	41.34 ±6.95	41.44 ±9.05	240.01 ±32.57
Phvul.007G041000	Peptidase of plants and bacteria	18.42 ±3.21	16.7 ±6.21	5.6 ±0.32	6.97 ±1.33	17.77 ±4.35	16.75 ±2.32	8.85 ±1.18	36.8 ±2.45

Table S5 continued

		Transcript levels (FPKM)							
		Mock inoculation				*Xap* inoculation			
**Gene ID**	**Gene Annotation**	**0 h PI**	**8 h PI**	**24 h PI**	**48 h PI**	**0 h PI**	**8 h PI**	**24 h PI**	**48 h PI**
Phvul.007G048500, *Phvul.007G048600*	Cysteine-rich receptor-like protein kinase 28-related	1.87 ±0.16	2.08 ±0.28	1.28 ±0.11	1.66 ±0.14	2.1 ±0.27	2.45 ±0.44	1.96 ±0.18	10.4 ±2.67
Phvul.007G048800	Cysteine-rich receptor-like protein kinase 28-related	0.31 ±0.06	0.2 ±0.04	0.22 ±0.05	0.27 ±0.1	0.23 ±0.08	0.28 ±0.05	0.37 ±0.12	1.6 ±0.49
Phvul.007G048900, *Phvul.007G049100*, *Phvul.007G049200*	Cysteine-rich receptor-like protein kinase 28-related; salt stress response/antifungal	5.06 ±2.01	7.25 ±1.23	2.46 ±0.7	3.25 ±0.61	6 ±0.53	7.58 ±1.1	4.28 ±1.05	14.15 ±1.81
Phvul.007G049700	Cysteine-rich repeat secretory protein 1-related	0.41 ±0.22	0.19 ±0.16	1.89 ±0.88	1.05 ±0.51	0 ±0	0.17 ±0.17	1.74 ±1.18	59.51 ±3.16
Phvul.007G052500	Cysteine-rich receptor-like protein kinase 28-related	0.06 ±0.01	0.12 ±0.05	0.15 ±0.05	0.09 ±0.03	0.13 ±0.01	0.1 ±0.05	0.07 ±0.02	1 ±0.06
Phvul.007G091000	Isoliquiritigenin 2'-*O*-methyltransferase	0.26 ±0.13	0.43 ±0.09	0.31 ±0.12	0.23 ±0.09	0.11 ±0.06	0.25 ±0.08	0.76 ±0.25	3.77 ±0.11
Phvul.007G101400	No functional annotation	0.43 ±0.13	0.27 ±0.16	0.88 ±0.29	0.84 ±0.13	0.38 ±0.23	0.62 ±0.1	0.92 ±0.21	3.6 ±0.34
Phvul.007G123958	Endoglucanase 7	0.42 ±0.08	0.21 ±0.05	0.39 ±0.08	0.46 ±0.05	0.2 ±0.03	0.25 ±0.01	0.43 ±0.07	3.31 ±0.43
Phvul.007G134700	Glutathione S-transferase, GST, superfamily, GST domain containing	0.92 ±0.61	1.4 ±0.23	1.43 ±0.47	0.84 ±0.28	0.69 ±0.13	1.28 ±0.38	2.02 ±0.25	13.81 ±0.74
Phvul.007G160901	Leucine-rich repeat-containing protein	0.31 ±0.13	0.48 ±0.1	0.12 ±0.03	0.16 ±0.06	0.32 ±0.03	0.44 ±0.14	0.3 ±0.09	0.8 ±0.4
Phvul.007G186900	No functional annotation	0.56 ±0.08	0.49 ±0.18	0.48 ±0.11	0.82 ±0.23	0.71 ±0.18	0.6 ±0.25	0.67 ±0.23	11.68 ±0.72
Phvul.007G211600	Protein NIM1-interacting 1	2.53 ±0.87	2.24 ±0.65	1.02 ±0.09	1.38 ±0.42	1.38 ±0.27	2.61 ±0.88	3.72 ±1.91	22.17 ±13.28

Table S5 continued

		Transcript levels (FPKM)							
		Mock inoculation				*Xap* inoculation			
**Gene ID**	**Gene Annotation**	**0 h PI**	**8 h PI**	**24 h PI**	**48 h PI**	**0 h PI**	**8 h PI**	**24 h PI**	**48 h PI**
Phvul.007G220400	No functional annotation	2.11 ±0.84	1.74 ±1.39	5.08 ±0.29	4.85 ±1.54	2.29 ±0.38	1.14 ±0.12	6.8 ±1.11	27.99 ±9.22
Phvul.007G222500	Ethylene-responsive transcription factor ERF016-related	0.15 ±0.15	0.23 ±0.12	0 ±0	0 ±0	0 ±0	0.05 ±0.02	0 ±0	0.77 ±0.48
Phvul.007G252500	PR5-like receptor kinase-related	0.26 ±0.08	0.19 ±0.06	0.25 ±0.06	0.28 ±0.03	0.18 ±0.04	0.24 ±0.04	0.24 ±0.06	1.21 ±0.36
Phvul.007G259400	Late embryogenesis abundant protein 4-5	0.31 ±0.1	0.18 ±0.07	0.11 ±0.06	0.25 ±0.09	0.48 ±0.12	0.18 ±0.02	0.21 ±0.1	8.3 ±7.11
Phvul.007G260400	L-type lectin-domain containing receptor kinase S.5-related	0.14 ±0.12	0.09 ±0.02	0.18 ±0.05	0.52 ±0.16	0.08 ±0.04	0.23 ±0.1	0.24 ±0.12	2.13 ±0.32
Phvul.007G273000	Ethylene-responsive transcription factor 1B	1.51 ±0.22	1.94 ±0.37	1.12 ±0.14	2.49 ±1.36	1.64 ±0.43	1.88 ±0.32	2.26 ±0.6	14.24 ±2.62
Phvul.007G278900	Calcium binding protein	3.36 ±0.72	3.22 ±1.4	2.97 ±0.88	2.93 ±1.24	3.86 ±0.69	3.89 ±0.59	6.77 ±1.02	31.71 ±11.13
Phvul.008G008800	No functional annotation	0.14 ±0.1	0.08 ±0.08	0.33 ±0.17	0.13 ±0.02	0 ±0	0.13 ±0.07	0.15 ±0.08	1.16 ±0.18
Phvul.008G011400	Heat shock protein 70 kDa	0.27 ±0.03	0.29 ±0.13	0.23 ±0.09	0.29 ±0.04	0.33 ±0.11	0.29 ±0.01	0.3 ±0.05	1.59 ±0.29
Phvul.008G011500	Thioredoxin	0.15 ±0.08	0.17 ±0.09	0.28 ±0.15	0.35 ±0.06	0.31 ±0.03	0.29 ±0.11	0.64 ±0.09	4.07 ±0.4
Phvul.008G011800	Thioredoxin	0.06 ±0.03	0.05 ±0.05	0.14 ±0.03	0.13 ±0.02	0.08 ±0.01	0.13 ±0.05	0.4 ±0.05	4.07 ±0.42
Phvul.008G011900	Heat shock protein 70 kDa	0.29 ±0.08	0.29 ±0.1	0.24 ±0.03	0.2 ±0.03	0.26 ±0.06	0.32 ±0.08	0.36 ±0.09	1.48 ±0.23

Table S5 continued

		Transcript levels (FPKM)							
		Mock inoculation				*Xap* inoculation			
Gene ID	Gene Annotation	0 h PI	8 h PI	24 h PI	48 h PI	0 h PI	8 h PI	24 h PI	48 h PI
Phvul.008G014600	C2H2-type zinc finger	0.07 ±0.03	0.1 ±0.06	0.37 ±0.03	0.63 ±0.23	0 ±0	0.07 ±0.06	0.5 ±0.24	2.85 ±0.48
Phvul.008G015800	6'-Deoxychalcone synthase	0.89 ±0.83	1.15 ±0.34	0.83 ±0.13	0.9 ±0.23	0.14 ±0.09	1.11 ±0.57	0.92 ±0.09	17.93 ±1.1
Phvul.008G028900	UDP-arabinopyranose mutase	3.46 ±1.37	5.31 ±0.99	1.34 ±0.16	1.73 ±0.56	2.7 ±0.43	5.97 ±1.88	2.24 ±0.26	9.31 ±2.36
Phvul.008G037300	Calmodulin	5.3 ±1.33	3.3 ±1.15	3 ±0.2	8.85 ±5.39	4.35 ±1.43	4.02 ±1.19	9.38 ±2.44	37.89 ±14.74
Phvul.008G040800	ATP-dependent zinc metalloprotease FTSH 6, chloroplastic	0 ±0	0.01 ±0.01	0.07 ±0.01	0.05 ±0.04	0.07 ±0.03	0.02 ±0.02	0.12 ±0.04	0.64 ±0.4
Phvul.008G043400	Receptor like protein 54	0.11 ±0.02	0.07 ±0.01	0.1 ±0.06	0.06 ±0.03	0.03 ±0.03	0.05 ±0.01	0.03 ±0.01	1.04 ±0.63
Phvul.008G044900	AAA-type ATPase family protein-related	1.46 ±0.08	2.38 ±1.02	1.69 ±0.18	2.26 ±0.93	1.22 ±0.24	1.93 ±0.17	2.7 ±0.99	15.86 ±3.86
Phvul.008G048466	Prenylated RAB acceptor 1-related	13.29 ±2.09	13.27 ±4.81	7.31 ±0.28	14.62 ±3.05	9.16 ±3.68	11.2 ±2.44	15.72 ±3.79	59.83 ±23.74
Phvul.008G048532	Prenylated RAB acceptor 1-related	6.56 ±1.32	6.99 ±2.96	5.09 ±0.88	5.97 ±2.06	7.38 ±2.15	7.74 ±0.94	6.18 ±1.55	35.87 ±16.82
Phvul.008G076500	Vestitone reductase	0.52 ±0.06	0.4 ±0.05	1 ±0.3	0.78 ±0.14	0.24 ±0.01	0.68 ±0.27	1.11 ±0.46	7.5 ±0.24
Phvul.008G076600	Vestitone reductase	1.76 ±0.81	2.45 ±0.54	2.43 ±0.52	3.34 ±0.42	0.67 ±0.1	2.54 ±0.82	3.92 ±0.83	36.27 ±0.99
Phvul.008G094400	GRAS domain family	0.05 ±0.04	0.05 ±0.04	0.05 ±0.03	0.15 ±0.1	0.05 ±0.05	0.03 ±0.02	0.11 ±0.06	1.95 ±0.77
Phvul.008G094500	Protein kinase domain / Legume lectin domain	0.38 ±0.14	0.32 ±0.16	0.39 ±0.09	0.82 ±0.41	0.27 ±0.04	0.18 ±0.05	0.6 ±0.08	3.35 ±1.31
Phvul.008G094900	Ammonium transporter 2	1.34 ±0.27	1.19 ±0.16	1.1 ±0.08	1.79 ±0.6	0.98 ±0.22	1.29 ±0.31	3.17 ±1.02	11.57 ±3.68

Table S5 continued

		Transcript levels (FPKM)							
		Mock inoculation				*Xap* inoculation			
**Gene ID**	**Gene Annotation**	**0 h PI**	**8 h PI**	**24 h PI**	**48 h PI**	**0 h PI**	**8 h PI**	**24 h PI**	**48 h PI**
Phvul.008G098500	Flavonol synthase	0.02 ±0.02	0.07 ±0.03	0.07 ±0.07	0.09 ±0.06	0 ±0	0.06 ±0.04	0.17 ±0.16	1.22 ±0.55
Phvul.008G112200	Laccase-7-related	0.52 ±0.12	0.6 ±0.2	1.67 ±0.69	0.69 ±0.21	0.24 ±0.05	0.71 ±0.34	1.48 ±0.84	11.42 ±2.63
Phvul.008G114766	No functional annotation	0.4 ±0.27	0.38 ±0.31	0.44 ±0.44	1.14 ±0.76	0.46 ±0.35	0.22 ±0.14	1.95 ±1.03	8.13 ±6.03
Phvul.008G123900	SNARE proteins	0.24 ±0.09	0.21 ±0.08	0.55 ±0.2	0.43 ±0.27	0.27 ±0.11	0.15 ±0.05	1.02 ±0.29	9.73 ±1.48
Phvul.008G127200	Deacetoxyvindoline 4-hydroxylase / Desacetyoxyvindoline-17-hydroxylase	0.95 ±0.25	0.7 ±0.14	1.18 ±0.21	1.16 ±0.31	0.63 ±0.1	0.96 ±0.12	1.57 ±0.49	4.71 ±1.41
Phvul.008G139900	No functional annotation	175.27 ±67.05	232.13 ±35.8	116.11 ±20.67	137.25 ±10.28	113.33 ±33.12	261.54 ±64.33	171.45 ±53.6	1151.16 ±76.66
Phvul.008G140113	No functional annotation	7.68 ±4.32	11.59 ±2.25	6.37 ±1.67	7.5 ±1.25	4.61 ±1.62	13.87 ±3.29	10.05 ±3.59	142.24 ±18.43
Phvul.008G140800	Hydroquinone glucosyltransferase	1.06 ±0.3	1.78 ±0.61	2.16 ±0.55	2.31 ±0.59	1.1 ±0.25	1.69 ±0.32	3.64 ±0.93	15.9 ±1.8
Phvul.008G169600	LL-diaminopimelate aminotransferase	0.86 ±0.42	1.54 ±0.33	1.46 ±0.48	1.96 ±1.15	0.44 ±0.15	1.46 ±0.52	2.65 ±1.41	19.04 ±2.33
Phvul.008G172400	Trehalose-phosphate phosphatase D-related	0.23 ±0.04	0.22 ±0.02	0.13 ±0.03	0.14 ±0.05	0.29 ±0.03	0.18 ±0.02	0.29 ±0.07	4.65 ±0.47
Phvul.008G176900	Protein of unknown function	0.64 ±0.2	0.28 ±0.06	0.12 ±0.04	0.45 ±0.24	0.36 ±0.14	0.55 ±0.29	0.36 ±0.03	3.51 ±0.91
Phvul.008G186200	No functional annotation	7.45 ±4.71	2.61 ±0.88	10.46 ±2.42	11.82 ±7.06	5.72 ±1.82	3.62 ±1.69	8.22 ±1.65	73.58 ±44.86
Phvul.008G193900	Aspartyl protease-like protein	1.34 ±0.2	1.11 ±0.09	0.66 ±0.13	1.74 ±0.81	1.16 ±0.23	1.29 ±0.09	2.43 ±0.67	7.57 ±4.05

Table S5 continued

		Transcript levels (FPKM)							
		Mock inoculation				*Xap* inoculation			
**Gene ID**	**Gene Annotation**	**0 h PI**	**8 h PI**	**24 h PI**	**48 h PI**	**0 h PI**	**8 h PI**	**24 h PI**	**48 h PI**
Phvul.008G194600	NAC domain-containing protein 6-related	0.28 ±0.13	0.11 ±0.06	0.37 ±0.22	0.25 ±0.07	0.17 ±0.09	0.24 ±0.08	0.36 ±0.06	2.51 ±0.53
Phvul.008G194828	Leucine-rich repeat-containing protein	0.37 ±0.06	0.16 ±0.05	0.34 ±0.11	0.43 ±0.21	0.32 ±0.1	0.19 ±0.08	0.87 ±0.33	1.74 ±1.09
Phvul.008G213914	Aminocyclopropane-carboxylate oxidase	0 ±0	0.04 ±0.01	0.1 ±0.06	0.07 ±0.02	0.05 ±0.05	0.03 ±0.02	0.01 ±0.01	0.86 ±0.43
Phvul.008G223500	Leucoanthocyanidin dioxygenase	0.11 ±0.05	0.09 ±0.06	0.17 ±0.02	0.28 ±0.2	0.05 ±0.05	0.03 ±0.02	0.38 ±0.17	1.14 ±0.49
Phvul.008G224400	α/β-hydrolase related protein	0.13 ±0.02	0.26 ±0.03	0.28 ±0.14	0.84 ±0.05	0.14 ±0.08	0.34 ±0.1	1.3 ±0.21	3.41 ±1.2
Phvul.008G232700	Extended synaptotagmin-related	0.21 ±0.03	0.29 ±0.08	0.35 ±0.08	0.44 ±0.39	0.56 ±0.22	0.28 ±0.15	1.19 ±0.66	5.35 ±2.22
Phvul.008G238200	No functional annotation	36.82 ±4.95	28.34 ±10.25	17.67 ±1.47	39.65 ±21.12	15.9 ±9.1	24.54 ±9.77	30.36 ±13.57	160 ±42.82
Phvul.008G242900	Late embryogenesis abundant (LEA) hydroxyproline-rich glycoprotein-related	0.26 ±0.26	0.32 ±0.12	0.33 ±0.16	0.36 ±0.2	0.11 ±0.07	0.26 ±0.03	0.8 ±0.03	5.77 ±0.28
Phvul.008G248900	CBL-interacting serine/threonine-protein kinase 2	1.17 ±0.51	0.46 ±0.36	0.66 ±0.09	0.71 ±0.16	0.84 ±0.04	0.59 ±0.38	0.79 ±0.17	3.34 ±1.91
Phvul.008G285000	Calcium transporting ATPase	1 ±0.08	0.76 ±0.06	0.89 ±0.02	1 ±0.28	0.61 ±0.12	0.77 ±0.09	1.13 ±0.07	5.1 ±1.25
Phvul.008G288600, Phvul.008G288700	Exosome complex component RRP42; nudix hydrolase related	2.75 ±0.85	3.07 ±0.52	1.69 ±0.02	1.62 ±0.36	2.34 ±0.28	3.55 ±0.78	2.52 ±0.33	6.78 ±0.65
Phvul.008G289200	No functional annotation	0.36 ±0.1	0.63 ±0.25	0.18 ±0.01	0.35 ±0.15	0.25 ±0.13	0.42 ±0.12	0.38 ±0.17	1.59 ±0.27

Table S5 continued

		Transcript levels (FPKM)							
		Mock inoculation				*Xap* inoculation			
**Gene ID**	**Gene Annotation**	**0 h PI**	**8 h PI**	**24 h PI**	**48 h PI**	**0 h PI**	**8 h PI**	**24 h PI**	**48 h PI**
Phvul.008G289500	Phenylalanine ammonia-lyase	0.96 ±0.12	0.88 ±0.24	0.88 ±0.24	0.69 ±0.12	0.76 ±0.09	0.94 ±0.1	1.31 ±0.21	4.47 ±0.25
Phvul.009G014800	F14l17.20 protein	0.12 ±0.05	0.1 ±0.04	0.18 ±0.12	0.15 ±0.05	0.1 ±0.05	0.12 ±0.02	0.07 ±0.04	1.07 ±0.16
Phvul.009G032900	No functional annotation	4.95 ±0.24	5.19 ±2.44	3.18 ±0.93	12.1 ±7	4.4 ±0.28	3.51 ±0.43	10.87 ±3.16	74.76 ±28.24
Phvul.009G042800	No functional annotation	0.32 ±0.07	0.17 ±0.1	0.29 ±0.15	0.17 ±0.12	0.13 ±0.13	0.19 ±0.1	0.3 ±0.15	4.79 ±2.37
Phvul.009G043100	WRKY DNA-binding domain	11.61 ±2.04	13.04 ±1.94	6.22 ±0.66	6.25 ±1.33	8.6 ±0.25	12.67 ±2.38	8.61 ±1.51	39.4 ±15.01
Phvul.009G046800	No functional annotation	23.85 ±2.96	17.53 ±2.05	17.8 ±2.3	16.56 ±3.59	15.31 ±2.47	15.04 ±1.1	19.51 ±2.83	205.74 ±4.75
Phvul.009G046900	Leucine rich repeat (LRR_1) // Protein tyrosine kinase // Leucine rich repeat N-terminal domain (LRRNT_2) // Leucine rich repeat (LRR_8)	0.24 ±0.14	0.12 ±0.06	0.18 ±0.04	0.27 ±0.06	0.07 ±0.03	0.2 ±0.1	0.34 ±0.09	6.91 ±0.98
Phvul.009G080000	WRKY DNA-binding domain	0.57 ±0.18	1.07 ±0.16	0.7 ±0.12	0.95 ±0.23	0.41 ±0.17	1.16 ±0.19	1.54 ±0.44	9.84 ±1.87
Phvul.009G087400	WRKY transcription factor 40-related	0.69 ±0.12	0.56 ±0.17	0.64 ±0.02	0.62 ±0.29	0.49 ±0.11	0.84 ±0.09	0.78 ±0.19	10.32 ±2.1
Phvul.009G118800	Expressed protein-related	0.06 ±0.02	0.08 ±0.02	0.16 ±0.08	0.23 ±0.02	0.05 ±0.01	0.12 ±0.03	0.29 ±0.12	2.61 ±0.54
Phvul.009G138900	WRKY DNA-binding domain	5.18 ±0.59	4.85 ±1.41	3.49 ±0.69	7.3 ±1.13	4.91 ±0.33	4.77 ±0.71	7.51 ±1.82	30.67 ±5.29

Table S5 continued

		Transcript levels (FPKM)							
		Mock inoculation				*Xap* inoculation			
**Gene ID**	**Gene Annotation**	**0 h PI**	**8 h PI**	**24 h PI**	**48 h PI**	**0 h PI**	**8 h PI**	**24 h PI**	**48 h PI**
Phvul.009G154100	Amino acid transporter	0.26 ±0.19	0.74 ±0.21	0.51 ±0.08	0.42 ±0.05	0.14 ±0.05	0.74 ±0.35	0.59 ±0.21	1.76 ±0.34
Phvul.009G155800	No functional annotation	0.58 ±0.08	0.58 ±0.31	0.53 ±0.1	0.58 ±0.29	0.37 ±0.05	0.5 ±0.01	0.72 ±0.23	3.34 ±1.31
Phvul.009G156300	No apical meristem (NAM) protein	0.78 ±0.47	1.1 ±0.44	0.17 ±0.09	0.54 ±0.1	0.29 ±0.12	1.19 ±0.49	0.51 ±0.16	2.22 ±1.44
Phvul.009G180501	Leucine rich repeat N-terminal domain (LRRNT_2) // Leucine rich repeat (LRR_8)	5.42 ±3.87	9.57 ±4.28	1.21 ±0.43	0.62 ±0.29	1.7 ±0.37	10.52 ±5.47	3.26 ±1.66	2.74 ±1.09
Phvul.009G182300	Aminobutyraldehyde dehydrogenase / γ-guanidinobutyraldehyde dehydrogenase	10.84 ±5.25	12.95 ±3.63	7.68 ±1.02	6.19 ±0.64	6.18 ±0.5	14.36 ±3.83	10.37 ±1.02	40.16 ±16.72
Phvul.009G189300	Molybdate-transporting ATPase	0.19 ±0.03	0.22 ±0.05	0.3 ±0.06	0.3 ±0.12	0.1 ±0.03	0.23 ±0.13	0.34 ±0.04	2.06 ±0.22
Phvul.009G210100	Protein phosphatase 2C	1.58 ±0.28	1.04 ±0.4	1.75 ±0.35	1.73 ±0.33	1.56 ±0.25	0.89 ±0.13	2.05 ±0.26	7.97 ±2.37
Phvul.009G211000	Extensin-like protein repeat	18.39 ±6.69	25.36 ±4.21	51.92 ±9.86	45.25 ±10.73	11.8 ±3.14	31.54 ±9.55	51.53 ±9.19	241.75 ±7.85
Phvul.009G225300	Protein TIFY 10a-related	12.66 ±1.22	10.84 ±1.71	15.8 ±0.98	16.3 ±2.94	14.84 ±2.53	11.56 ±0.76	24.67 ±5.2	81.37 ±35.7
Phvul.009G227001	SER/THR protein kinase ACIK1B	0.04 ±0.02	0.06 ±0.02	0.14 ±0.06	0.24 ±0.03	0.06 ±0.04	0.06 ±0.03	0.2 ±0.06	1.15 ±0.36
Phvul.009G231600	Sterol regulatory element-binding protein	0.69 ±0.17	0.65 ±0.12	0.47 ±0.13	0.75 ±0.11	0.53 ±0.15	0.68 ±0.03	0.86 ±0.2	4.5 ±0.37

Table S5 continued

		Transcript levels (FPKM)							
		Mock inoculation				*Xap* inoculation			
**Gene ID**	**Gene Annotation**	**0 h PI**	**8 h PI**	**24 h PI**	**48 h PI**	**0 h PI**	**8 h PI**	**24 h PI**	**48 h PI**
Phvul.009G235700	Helix-loop-helix DNA-binding domain (HLH)	1.43 ±0.06	2.14 ±0.93	1.77 ±0.2	1.15 ±0.2	1.47 ±0.24	1.44 ±0.05	2.86 ±0.91	17.83 ±7.19
Phvul.009G240750	No functional annotation	2.92 ±1.07	2.23 ±1.6	1.89 ±0.53	4.46 ±1.91	1.83 ±1.19	3.33 ±0.44	3.58 ±1.81	30.01 ±9.09
Phvul.009G241900	VQ motif	1.19 ±0.33	1.41 ±0.23	1.61 ±0.32	1.66 ±0.14	0.69 ±0.14	1.56 ±0.47	2.84 ±0.86	14.82 ±1.78
Phvul.009G244200	Isoflavone 2'-hydroxylase	0.28 ±0.14	0.17 ±0.11	0.67 ±0.13	0.86 ±0.06	0.08 ±0.05	0.21 ±0.09	0.83 ±0.06	4.4 ±0.16
Phvul.009G256400	Glucan endo-1,3-β-d-glucosidase / Laminarinase	49.3 ±24.32	72.4 ±26.75	43.1 ±13.16	104.6 ±6.97	25.95 ±6.85	70.12 ±23.73	76.07 ±23.3	547.25 ±144.34
Phvul.009G258600	Anthranilate N-methyltransferase	3.94 ±1.39	4.01 ±0.37	4.55 ±1.18	4.52 ±0.91	1.8 ±0.19	4.02 ±0.91	6.81 ±1.25	64.16 ±2.2
Phvul.009G262300	GDSL esterase/lipase 5-related	0.33 ±0.05	1.03 ±0.5	0.36 ±0.1	0.68 ±0.17	0.82 ±0.21	0.67 ±0.01	0.69 ±0.06	3.23 ±0.36
Phvul.009G262900	Lipoxygenase 3, chloroplastic-related	0.19 ±0.08	0.13 ±0.02	0.24 ±0.09	0.15 ±0.05	0.07 ±0.02	0.27 ±0.1	0.25 ±0.03	2.01 ±0.09
Phvul.010G000300	Leucocyanidin oxygenase	1.43 ±0.48	2.2 ±0.79	1.89 ±0.61	1.43 ±0.13	1 ±0.43	1.49 ±0.4	3.34 ±1.5	6.66 ±2.77
Phvul.010G003400	No functional annotation	0.08 ±0.08	0.13 ±0.08	0.11 ±0.08	0.16 ±0.05	0.09 ±0.05	0.03 ±0.02	0.41 ±0.24	2.75 ±0.45
Phvul.010G005900	Laccase-7-related	0.15 ±0.05	0.02 ±0.02	0.17 ±0.15	0.16 ±0.08	0.02 ±0.02	0.13 ±0.13	0.18 ±0.14	1.25 ±0.29
Phvul.010G006000	PAR1 protein	4.12 ±0.98	3.47 ±1.28	2.88 ±0.4	3.33 ±1.43	3.59 ±0.52	3.49 ±0.57	5.89 ±1.82	33.94 ±11.78
Phvul.010G007400	*O*-Fucosyltransferase family protein	0.13 ±0.04	0.07 ±0.05	0.1 ±0.06	0.1 ±0.06	0.42 ±0.05	0.18 ±0.06	0.21 ±0.07	0.88 ±0.51
Phvul.010G008800	Glyceollin synthase	0.17 ±0.09	0.12 ±0.06	0.2 ±0.16	0.19 ±0.11	0.03 ±0.03	0.17 ±0.09	0.39 ±0.2	2.14 ±0.68

Table S5 continued

		Transcript levels (FPKM)							
		Mock inoculation				*Xap* inoculation			
**Gene ID**	**Gene Annotation**	**0 h PI**	**8 h PI**	**24 h PI**	**48 h PI**	**0 h PI**	**8 h PI**	**24 h PI**	**48 h PI**
Phvul.010G016200	Domain of unknown function	0.3 ±0.09	0.21 ±0.1	0.27 ±0.06	0.35 ±0.13	0.35 ±0.14	0.21 ±0.1	0.36 ±0.06	2.19 ±1.31
Phvul.010G021001	GDSL esterase/lipase 5-related	0.38 ±0.17	0.32 ±0.08	0.69 ±0.36	1.16 ±0.39	0.08 ±0.05	0.27 ±0.02	0.83 ±0.38	33.9 ±1.49
Phvul.010G021700	Membrane protein of ER body 2	0.23 ±0.18	0.17 ±0.06	0.44 ±0.16	0.33 ±0.21	0.12 ±0.1	0.42 ±0.12	0.51 ±0.06	2.35 ±0.25
Phvul.010G032000	Protein kinase domain //D-mannose binding lectin	0.2 ±0.04	0.16 ±0.05	0.1 ±0.03	0.12 ±0.03	0.45 ±0.07	0.31 ±0.04	0.11 ±0.01	0.58 ±0.31
Phvul.010G044900, Phvul.010G045200	No functional annotation	2.49 ±0.24	2.75 ±1.06	2.12 ±0.53	2.54 ±0.1	2.11 ±0.42	2.15 ±0.05	3.14 ±0.6	17.96 ±4.46
Phvul.010G045000	No functional annotation	0.65 ±0.15	0.97 ±0.43	0.86 ±0.14	1.13 ±0.1	0.48 ±0.18	0.96 ±0.18	1.1 ±0.1	10.69 ±1.16
Phvul.010G057300, Phvul.010G057500, Phvul.010G057600	Protein kinase domain // S-locus glycoprotein domain // D-mannose binding lectin	0.51 ±0.13	0.6 ±0.07	0.5 ±0.03	0.35 ±0.03	0.37 ±0.1	0.56 ±0.06	0.48 ±0.07	1.56 ±0.14
Phvul.010G059000	Had superfamily, subfamily IIIB acid phosphatase	3.91 ±0.69	4.22 ±1.85	2.39 ±0.2	3.57 ±0.7	4.05 ±0.49	4.4 ±0.79	5.18 ±0.59	19.85 ±4.32
Phvul.010G063600	Dirigent protein 19	7.14 ±3.78	12.75 ±2.53	4.9 ±0.14	5.27 ±0.63	5.03 ±1.29	12.92 ±3.38	12.42 ±1.7	28.7 ±2.59
Phvul.010G063800	Dirigent protein 19	6.7 ±2.04	8.83 ±1.09	9 ±2.14	7.37 ±2.08	3.43 ±0.16	10.32 ±3.47	15.47 ±3.89	72.32 ±13.91
Phvul.010G070250	PTHR36766:SF1 - HR3	1.28 ±0.18	1.11 ±0.32	0.68 ±0.24	1.27 ±0.27	1.41 ±0.59	1.53 ±0.19	1.63 ±0.11	5.34 ±2.45
Phvul.010G073500	Uncharacterized protein	0.19 ±0.1	0.14 ±0.08	0.35 ±0.21	0.28 ±0.14	0.13 ±0.07	0.09 ±0.03	0.32 ±0.06	5.43 ±4.42
Phvul.010G075400	Glutathione S-transferase U1-related	2.85 ±0.55	3.9 ±0.35	3.16 ±0.39	3.92 ±0.61	2.98 ±0.31	3.53 ±0.73	4.4 ±0.77	15.93 ±0.44
Phvul.010G079900	Pectinesterase/pectinesterase inhibitor 17-related	0.76 ±0.1	0.54 ±0.13	1 ±0.33	0.93 ±0.12	0.36 ±0.13	0.69 ±0.15	1 ±0.27	12.4 ±0.65

Table S5 continued

		Transcript levels (FPKM)							
		Mock inoculation				*Xap* inoculation			
**Gene ID**	**Gene Annotation**	**0 h PI**	**8 h PI**	**24 h PI**	**48 h PI**	**0 h PI**	**8 h PI**	**24 h PI**	**48 h PI**
Phvul.010G089900	BON1-associated protein 1-related	0.49 ±0.03	0.24 ±0.07	0.39 ±0.14	0.51 ±0.1	0.75 ±0.25	0.37 ±0.19	0.67 ±0.13	6.76 ±1.87
Phvul.010G090000	BON1-associated protein 1-related	0.74 ±0.39	1.02 ±0.62	1.03 ±0.07	1.79 ±1.22	0.95 ±0.38	0.78 ±0.24	3.08 ±0.98	19.25 ±10.07
Phvul.010G093400	BON1-associated protein 1-related	0.21 ±0.12	0.17 ±0.13	0.06 ±0.06	0.2 ±0.03	0.33 ±0.08	0.03 ±0.03	0.51 ±0.26	3.53 ±1.04
Phvul.010G112600	CGI-141-related/lipase containing protein	0 ±0	0.01 ±0.01	0.07 ±0.07	0.16 ±0.06	0 ±0	0.01 ±0.01	0.04 ±0.02	0.82 ±0.42
Phvul.010G117200	Homeobox-leucine zipper protein ATHB-12-related	1.58 ±0.79	1.09 ±0.23	1.47 ±0.42	2.89 ±1.64	0.57 ±0.09	1.13 ±0.24	2.34 ±0.42	23.02 ±15.06
Phvul.010G120200	Anthranilate N-methyltransferase	0.14 ±0.09	0.22 ±0.08	0.18 ±0.11	0.14 ±0.09	0.02 ±0.02	0.22 ±0.11	0.37 ±0.2	6.33 ±0.9
Phvul.010G128900	Flavonoid 3'-monooxygenase / Flavonoid 3'-hydroxylase	39.67 ±9	51.76 ±5.85	21.25 ±1.94	21.35 ±2.09	33.68 ±3.12	54.42 ±6.47	33.76 ±7.48	100.49 ±31.46
Phvul.010G134000	Domain of unknown function	5.37 ±2.59	23.37 ±5.46	10.82 ±2.23	15.82 ±5.67	2.14 ±0.96	9.29 ±1.68	23.52 ±8.48	107 ±20.35
Phvul.010G137300	Calmodulin-binding protein	0.08 ±0.03	0.09 ±0.05	0.1 ±0.01	0.14 ±0.06	0.2 ±0.08	0.11 ±0.05	0.14 ±0.04	0.79 ±0.4
Phvul.010G137800, Phvul.010G137900	β-Fructofuranosidase, insoluble isoenzyme CWINV1-related	1.42 ±0.04	1.48 ±0.44	1.8 ±0.36	1.77 ±0.28	1.25 ±0.16	1.45 ±0.23	2.94 ±0.31	43.53 ±3.94
Phvul.010G155100	No functional annotation	2.05 ±0.48	1.91 ±0.55	1.7 ±0.21	2.35 ±1.02	2.32 ±0.92	2.15 ±0.51	3.83 ±1.22	12.2 ±4.05
Phvul.011G013600	α-Farnesene synthase	0.79 ±0.39	0.73 ±0.73	1.28 ±0.22	0.59 ±0.13	1.18 ±0.09	0.24 ±0.24	1.11 ±0.12	2.38 ±1.73
Phvul.011G025100	Amino acid transporter	9.22 ±1.37	8.98 ±4.61	8.45 ±0.62	9.36 ±3.17	13.18 ±2.28	8.37 ±2.73	15.19 ±3.24	41.28 ±9.45
Phvul.011G034800	Senescence-associated protein	1.22 ±0.21	0.72 ±0.06	0.88 ±0.05	0.99 ±0.33	0.6 ±0.06	0.89 ±0.28	3.38 ±1.43	4.47 ±2.71

Table S5 continued

Gene ID	Gene Annotation	Transcript levels (FPKM)							
		Mock inoculation				*Xap* inoculation			
		0 h PI	8 h PI	24 h PI	48 h PI	0 h PI	8 h PI	24 h PI	48 h PI
Phvul.011G035600	Adenine nucleotide α-hydrolases-domain containing protein kinase-related	0.16 ±0.14	0.16 ±0.05	0.14 ±0.06	0.16 ±0.05	0.03 ±0.03	0.22 ±0.16	0.21 ±0.09	1.24 ±0.21
Phvul.011G041400	S-locus glycoprotein domain // D-mannose binding lectin // protein tyrosine kinase	0.26 ±0.03	0.54 ±0.16	0.15 ±0.02	0.25 ±0.09	0.28 ±0.03	0.46 ±0.08	0.3 ±0.06	1.61 ±0.47
Phvul.011G043100	BCS1 AAA-type ATPase	5.84 ±0.29	6.62 ±1.64	3.59 ±0.23	4.8 ±0.83	5.81 ±0.18	5.74 ±0.38	6.73 ±1.49	23.38 ±7.74
Phvul.011G044500	NAD dependent epimerase/dehydratase	0.28 ±0.02	0.31 ±0.12	0.79 ±0.38	0.26 ±0.2	0.29 ±0.02	0.46 ±0.09	0.78 ±0.51	4.68 ±0.76
Phvul.011G051600	No functional annotation	0.18 ±0.09	0.25 ±0.09	0.25 ±0.12	0.64 ±0.64	0 ±0	0.15 ±0.08	0.45 ±0.31	5.6 ±1.43
Phvul.011G060300	Glucosyltransferase-like protein-related	0.06 ±0.06	0.06 ±0.02	0.14 ±0.08	0.17 ±0.05	0.03 ±0.01	0.05 ±0.03	0.21 ±0.08	1.67 ±0.4
Phvul.011G062900	Putative hydrolase of the HAD superfamily	0.45 ±0.18	0.53 ±0.25	0.51 ±0.1	0.18 ±0.03	0.35 ±0.03	0.36 ±0.03	0.5 ±0.17	1.46 ±0.54
Phvul.011G072200	Lob domain-containing protein 1-related	0.85 ±0.36	1.31 ±0.28	1.33 ±0.17	1.51 ±0.4	0.45 ±0.08	1.74 ±0.79	1.62 ±0.27	8.54 ±3.1
Phvul.011G077900	Glucan endo-1,3-β-D-glucosidase / Laminarinase	113.58 ±47.26	113.85 ±21.75	89.63 ±16.63	80.81 ±21.81	78.16 ±23.53	134.92 ±24.91	115.59 ±21.84	671.13 ±21.22
Phvul.011G089500	γ-Aminobutyrate transporter 1	0.32 ±0.12	0.33 ±0.15	0.38 ±0.15	0.27 ±0.1	0.15 ±0.02	0.37 ±0.08	0.41 ±0.13	3.6 ±0.44
Phvul.011G096300	F-box-like	0.4 ±0.17	0.78 ±0.15	0.4 ±0.12	0.49 ±0.18	0.33 ±0.06	0.67 ±0.17	0.87 ±0.38	3 ±0.43
Phvul.011G108600	EF-hand calcium-binding domain containing protein	2.2 ±0.22	2.25 ±0.94	1.67 ±0.52	3.33 ±0.51	3.27 ±0.86	4.21 ±1.83	3.66 ±1.05	18.08 ±2.4

Table S5 continued

		Transcript levels (FPKM)							
		Mock inoculation				*Xap* inoculation			
**Gene ID**	**Gene Annotation**	**0 h PI**	**8 h PI**	**24 h PI**	**48 h PI**	**0 h PI**	**8 h PI**	**24 h PI**	**48 h PI**
Phvul.011G109600	MYB-like DNA-binding protein	0.18 ±0.13	0.22 ±0.12	0.13 ±0.06	0.2 ±0.2	0.02 ±0.02	0.09 ±0.05	0.12 ±0.03	3.84 ±1.86
Phvul.011G147800	NAC domain-containing protein 19-related	0.05 ±0.03	0.08 ±0.06	0.02 ±0.01	0.58 ±0.28	0.03 ±0.03	0.11 ±0.04	0.33 ±0.14	6.47 ±3.31
Phvul.011G148500	No functional annotation	0.09 ±0.09	0.03 ±0.03	0.03 ±0.03	0.61 ±0.54	0 ±0	0.13 ±0.13	0.59 ±0.28	3.13 ±1.81
Phvul.011G150400	Cysteine-rich receptor-like protein kinase 27-related	0.34 ±0.09	0.59 ±0.09	0.28 ±0.05	0.17 ±0.05	0.37 ±0.03	0.5 ±0.02	0.38 ±0.12	1.55 ±0.4
Phvul.011G166500	Trypsin and protease inhibitor	0.95 ±0.23	0.64 ±0.13	1.99 ±0.68	1.18 ±0.07	1.09 ±0.44	0.83 ±0.21	1.53 ±0.55	41.53 ±2.24
Phvul.011G167300	Chitinase	13.43 ±4.41	15.61 ±2.15	13.54 ±2.48	12.81 ±2.57	10.54 ±1.62	16.53 ±1.38	16.43 ±2.67	199.32 ±2.61
Phvul.011G169900	Trypsin and protease inhibitor	0.38 ±0.19	0.3 ±0.11	0.5 ±0.04	0.53 ±0.04	0.43 ±0.08	0.15 ±0.06	0.28 ±0.12	14.3 ±0.89
Phvul.011G176950	Glucan endo-1,3-β-D-glucosidase / Laminarinase	41.53 ±3.63	57.12 ±29.51	23.69 ±0.25	24.91 ±6.13	45.91 ±14.23	55.25 ±7.93	37.77 ±9.5	295.71 ±30.8
Phvul.011G179600	Caspase regulator, RING finger domain-containing	0.4 ±0.08	0.62 ±0.1	0.42 ±0.06	0.37 ±0.1	0.61 ±0.03	0.45 ±0	0.51 ±0.12	2.92 ±0.28
Phvul.011G211600	No functional annotation	0 ±0	0 ±0	0 ±0	0 ±0	0.12 ±0.06	0 ±0	0 ±0	0.87 ±0.79
Phvul.011G214400	Protein kinase domain /D-mannose binding lectin	0.06 ±0.03	0.05 ±0.02	0.06 ±0.03	0.11 ±0.06	0.06 ±0.02	0.04 ±0.02	0.09 ±0.02	1.17 ±0.15
Phvul.L000770, Phvul.L001078	No functional annotation	1.06 ±0.45	1.39 ±0.65	1.53 ±0.54	3.2 ±0.16	1.29 ±0.14	2.01 ±0.73	3.62 ±0.87	28.35 ±15.29
Phvul.L003043	C2H2-type zinc finger family protein-related	0.6 ±0.29	0.7 ±0.2	0.3 ±0.11	0.68 ±0.14	1.18 ±0.34	0.79 ±0.43	0.74 ±0.26	7.24 ±3.97
XLOC_016664	No functional annotation	0.25 ±0.18	0.24 ±0.09	0.22 ±0.03	0.41 ±0.08	0.02 ±0.02	0.28 ±0.14	0.56 ±0.02	2.89 ±0.2

Table S5 continued

		Transcript levels (FPKM)							
		Mock inoculation				*Xap* inoculation			
**Gene ID**	**Gene Annotation**	**0 h PI**	**8 h PI**	**24 h PI**	**48 h PI**	**0 h PI**	**8 h PI**	**24 h PI**	**48 h PI**
XLOC_023524	No functional annotation	0 ±0	1.29 ±1.29	2.03 ±1.3	2 ±0.28	0.71 ±0.71	1.03 ±0.52	1.83 ±1.17	36.2 ±3.05
XLOC_027462	No functional annotation	0 ±0	0 ±0	0.32 ±0.32	0.57 ±0.14	0 ±0	0 ±0	0.24 ±0.08	5.03 ±0.74
XLOC_027490	No functional annotation	0.32 ±0.1	0.52 ±0.09	0.14 ±0.1	0.4 ±0.05	0.13 ±0.01	0.32 ±0.17	0.47 ±0.08	2.75 ±0.54

Table S6. Transcript abundance for genes upregulated in the CBB-susceptible RIL at 0 h post-inoculation (PI) with *Xanthomonas axonopodis* (*Xap*). Transcript levels for individual genes corresponding to each RIL/ inoculation treatment/ sampling time PI are represented as FPKM ± SE of three experimental replicates.

		**Transcript levels (FPKM)**							
		**Mock inoculation**				***Xap* inoculation**			
**Gene ID**	**Gene Annotation**	**0 h PI**	**8 h PI**	**24 h PI**	**48 h PI**	**0 h PI**	**8 h PI**	**24 h PI**	**48 h PI**
Phvul.002G076200	F3I6.5 protein	4.98 ±1.36	2.76 ±0.2	5.19 ±1.14	8.5 ±3.05	20.63 ±3.59	2.44 ±0.25	6.21 ±1.04	9 ±3.26
Phvul.002G165800	BCS1 AAA-type ATPase	0.47 ±0.16	0.1 ±0.06	0.2 ±0.04	0.29 ±0.14	1.88 ±0.17	0.04 ±0.04	0.25 ±0.02	0.27 ±0.16
Phvul.003G075300	No functional annotation	11 ±2.47	8.96 ±0.18	7.68 ±1.25	4.46 ±0.76	48.57 ±4.26	9.46 ±1.71	6.03 ±0.65	6.39 ±2.14
Phvul.003G079900	Glucan endo-1,3-β-glucosidase 1	4.37 ±1.08	2.21 ±0.09	2.25 ±0.23	1.04 ±0.09	20.32 ±8.19	2.26 ±0.44	1.42 ±0.34	2.11 ±0.84
Phvul.003G110200	COBRA-like protein 7-related	3.71 ±0.57	2.83 ±0.32	1.66 ±0.25	5.29 ±3.48	16.3 ±2.63	2.52 ±0.07	1.87 ±0.17	7.14 ±4.73
Phvul.003G138226	Very-long-chain 3-oxoacyl-CoA synthase	3.19 ±0.69	2.07 ±0.39	3.49 ±0.69	3.36 ±0.51	17.13 ±6.83	1.9 ±0.28	2.82 ±0.77	3.31 ±0.52
Phvul.003G140426	No functional annotation	5.51 ±1.18	4.37 ±0.35	5.65 ±0.4	5.11 ±0.62	23.1 ±4.88	3.91 ±0.78	5.08 ±0.33	4.87 ±1.09
Phvul.003G147300	Xyloglucan endotransglucosylase/ Hydrolase protein 21-related	0.15 ±0.08	0.01 ±0.01	0.1 ±0.03	0.21 ±0.13	1.14 ±0.67	0 ±0	0.03 ±0.03	0.04 ±0.03
Phvul.003G152300	NAD dependent epimerase/dehydratase	36.19 ±4.07	27.86 ±0.9	31.2 ±3.91	39.41 ±18.99	146.5 ±30.89	27.39 ±1.24	29.13 ±5.24	57.35 ±23.34

Table S6 continued

		Transcript levels (FPKM)							
		Mock inoculation				*Xap* inoculation			
**Gene ID**	**Gene Annotation**	**0 h PI**	**8 h PI**	**24 h PI**	**48 h PI**	**0 h PI**	**8 h PI**	**24 h PI**	**48 h PI**
Phvul.004G019600	Xyloglucan glycosyltransferase 4	1.97 ±0.31	1.33 ±0.12	1.3 ±0.09	1.12 ±0.21	8.95 ±2.21	1.41 ±0.1	1.5 ±0.17	1.88 ±0.58
Phvul.004G132000	Peroxidase 47	8.04 ±3.45	3.82 ±0.96	4.13 ±0.81	1.45 ±0.2	54.64 ±19.76	2.98 ±1.06	5.9 ±0.94	5.52 ±1.89
Phvul.004G173101	Protein of unknown function	4.65 ±0.16	5.01 ±0.25	2.71 ±0.65	6.11 ±1.85	24.27 ±7.84	3.73 ±0.63	2.3 ±0.17	5.12 ±2.11
Phvul.005G068500	Basic helix-loop-helix domain-containing protein	3.16 ±1.09	3.18 ±0.69	0.45 ±0.24	0.18 ±0.02	20.23 ±11.99	3.54 ±1.13	0.63 ±0.18	0.51 ±0.23
Phvul.005G111300	Xyloglucan endotransglucosylase / Hydrolase protein 9	22.23 ±6.44	17.23 ±0.46	11.41 ±2.32	7.29 ±1.71	139.23 ±91.53	17.06 ±1.01	10.8 ±0.95	10.14 ±2.29
Phvul.006G023000	Zinc finger FYVE domain containing protein	0.39 ±0.2	0.21 ±0.09	0.2 ±0.05	0.05 ±0.05	1.63 ±0.16	0.18 ±0.04	0.09 ±0.07	0.07 ±0.02
Phvul.006G167400	BCL2-associated athanogene	4.63 ±0.58	5.25 ±0.5	3.41 ±0.72	1.47 ±0.19	19.39 ±6.03	4.5 ±0.48	3.01 ±1.1	3.81 ±1.06
Phvul.006G204100	No functional annotation	7.81 ±1.87	4.93 ±0.98	4.05 ±0.24	30.76 ±20.77	35.96 ±8.97	3.86 ±0.95	3.54 ±0.69	20.34 ±12.31
Phvul.007G021700	Cupin domain (Cupin_2)	0.69 ±0.39	0.53 ±0.01	0.56 ±0.08	0.49 ±0.06	3.28 ±0.44	1.37 ±0.03	0.53 ±0.16	0.64 ±0.17
Phvul.007G198400	F-Box protein PP2-B13-related	2.42 ±0.76	0.57 ±0.29	2.85 ±0.48	3.51 ±0.21	10.94 ±1.91	0.92 ±0.11	2.98 ±0.18	3.42 ±0.49
Phvul.008G049600	Cyclin-U4-1	1.46 ±0.79	0.5 ±0.28	1.94 ±0.47	0.52 ±0.23	8.44 ±3.88	0.47 ±0.03	1.16 ±0.28	1.38 ±0.53
Phvul.008G081000	Receptor-like protein kinase feronia	5.86 ±0.52	3.11 ±1.56	4.14 ±0.45	3.73 ±0.44	25.19 ±7.19	4.96 ±0.4	3.89 ±0.29	5.67 ±1.64

Table S6 continued

		Transcript levels (FPKM)							
		Mock inoculation				*Xap* inoculation			
**Gene ID**	**Gene Annotation**	**0 h PI**	**8 h PI**	**24 h PI**	**48 h PI**	**0 h PI**	**8 h PI**	**24 h PI**	**48 h PI**
Phvul.008G174100	Predicted RNA-binding protein SEB4 (RRM superfamily)	0.23 ±0.05	0.02 ±0.02	0.09 ±0.05	0.01 ±0.01	1.01 ±0.85	0.04 ±0.02	0 ±0	0 ±0
Phvul.008G237500	Glutamine synthetase	0.67 ±0.05	0.68 ±0.4	0.22 ±0.03	0.14 ±0.05	4.21 ±2.71	1.02 ±0.23	0.14 ±0.05	0.13 ±0.08
Phvul.009G074400	Xyloglucan glycosyltransferase 12-related	4.36 ±0.39	2.48 ±1.24	2.21 ±0.36	2.03 ±0.44	18.13 ±6.41	4.07 ±0.45	1.81 ±0.35	3.17 ±1.52
Phvul.009G075801	No functional annotation	683.63 ±56.18	443.46 ±225.4	356.53 ±73.1	206.74 ±7.14	2865.96 ±646.99	590.81 ±71.25	241.43 ±24.29	337.75 ±80.59
Phvul.009G113400	F2J10.8 protein-related	12.99 ±2.38	6.95 ±3.48	9.59 ±1.77	6.11 ±1.24	62.11 ±7.73	11.94 ±0.96	9.04 ±0.85	9.19 ±2.68
Phvul.009G234900	No functional annotation	0 ±0	0.55 ±0.55	3.09 ±0.47	1.97 ±1.13	10.23 ±1.86	0.66 ±0.66	1.61 ±1.61	2.44 ±1.29
Phvul.010G061500	Wound-induced protein WI12	1.03 ±0.53	0.25 ±0.13	2.33 ±1.25	0.79 ±0.12	6.65 ±2.15	0.17 ±0.11	1.17 ±0.4	1.24 ±0.51
Phvul.010G152200	Gibberellin 3-β-dioxygenase 1-related	0.38 ±0.05	0.39 ±0.21	0.35 ±0.08	0.18 ±0.13	2.52 ±1.43	0.39 ±0.07	0.39 ±0.13	0.44 ±0.13
Phvul.011G054100	Calmodulin-like protein 6-related	7.02 ±1.93	2.55 ±1.39	7.15 ±0.92	14.32 ±4.38	37.3 ±4.83	4.14 ±0.66	7.14 ±0.37	14.26 ±4.21
Phvul.011G107000	Xyloglucan endotransglucosylase/ Hydrolase protein 9	5.15 ±2.12	5.36 ±2.8	2.17 ±0.29	1.97 ±0.39	34.64 ±27.35	7.76 ±1.44	2.29 ±0.66	2.69 ±0.68
XLOC_027363	No functional annotation	1.67 ±0.76	0.76 ±0.38	1.44 ±0.16	1.06 ±0.55	7.44 ±0.65	1.66 ±0.31	1.28 ±0.32	1.1 ±0.28

Table S7. Transcript abundance for genes upregulated in the CBB-susceptible RIL at 8 h post-inoculation (PI) with *Xanthomonas axonopodis* (*Xap*). Transcript levels for individual genes corresponding to each RIL/ inoculation treatment/ sampling time PI are represented as FPKM ± SE of three experimental replicates.

		**Transcript levels (FPKM)**							
		**Mock inoculation**				***Xap* inoculation**			
**Gene ID**	**Gene Annotation**	**0 h PI**	**8 h PI**	**24 h PI**	**48 h PI**	**0 h PI**	**8 h PI**	**24 h PI**	**48 h PI**
Phvul.011G127300	Cysteine protease family C1-related	0.48 ±0.46	0.32 ±0.18	0.14 ±0.11	0.05 ±0.01	0.06 ±0.03	1.42 ±0.73	0.22 ±0.04	0.11 ±0.03
XLOC_001894	No functional annotation	0.09 ±0.05	0 ±0	0 ±0	0.03 ±0.03	0.08 ±0.08	1.07 ±0.57	0.18 ±0.1	0.08 ±0.08

Table S8. Transcript abundance for genes upregulated in the CBB-susceptible RIL at 24 h post-inoculation (PI) with *Xanthomonas axonopodis* (*Xap*). Transcript levels for individual genes corresponding to each RIL/ inoculation treatment/ sampling time PI are represented as FPKM ± SE of three experimental replicates.

		**Transcript levels (FPKM)**							
		**Mock inoculation**				***Xap* inoculation**			
**Gene ID**	**Gene Annotation**	**0 h PI**	**8 h PI**	**24 h PI**	**48 h PI**	**0 h PI**	**8 h PI**	**24 h PI**	**48 h PI**
Phvul.003G012600	Auxin response factor 30-related	0.18 ±0.02	0.18 ±0.03	0 ±0	0.08 ±0.04	0.06 ±0.06	0.82 ±0.49	0.56 ±0.15	0.31 ±0.23
Phvul.003G143400	Peroxidase 16-related	0 ±0	0 ±0	0 ±0	0.13 ±0.07	0 ±0	0 ±0	0.6 ±0.55	0.26 ±0.25
Phvul.004G059200	Pectate lyase 11-related	1.27 ±0.46	2 ±0.11	3.17 ±0.47	3.75 ±0.87	0.73 ±0.31	1.67 ±0.13	13.34 ±7.33	11.84 ±7.47
Phvul.007G021300	Protein trichome birefringence-like 36	0.33 ±0.05	0.49 ±0.1	0.19 ±0.05	0.22 ±0.06	0.24 ±0.03	0.4 ±0.05	0.97 ±0.5	0.77 ±0.42
Phvul.009G108300	Adenosylhomocysteine nucleosidase	1.61 ±0.97	1.82 ±1.02	2.45 ±0.49	13.17 ±2.83	0.53 ±0.29	1.82 ±0.4	10.65 ±6.96	6.96 ±2.74

Table S9. Transcript abundance for genes upregulated in the CBB-susceptible RIL at 48 h post-inoculation (PI) with *Xanthomonas axonopodis* (*Xap*). Transcript levels for individual genes corresponding to each RIL/ inoculation treatment/ sampling time PI are represented as FPKM ± SE of three experimental replicates.

		**Transcript levels (FPKM)**							
		**Mock inoculation**				***Xap* inoculation**			
**Gene ID**	**Gene Annotation**	**0 h PI**	**8 h PI**	**24 h PI**	**48 h PI**	**0 h PI**	**8 h PI**	**24 h PI**	**48 h PI**
Phvul.001G001500	Multi-copper oxidase type I family protein-related	6.48 ±0.72	5.54 ±0.94	1.46 ±0.79	0.42 ±0.09	8.77 ±3.13	5.11 ±1.6	2.13 ±0.39	1.71 ±0.16
Phvul.001G146800	Receptor-like cytoplasmic kinase VIA5	6.06 ±0.7	5.74 ±0.27	1.17 ±0.24	0.2 ±0.06	10.6 ±0.43	4.62 ±0.68	1 ±0.52	1.23 ±0.35
Phvul.002G000900	Taxadien-5-α-ol *O*-acetyltransferase	5.1 ±1.86	1.59 ±0.21	4.1 ±0.18	0.62 ±0.19	6.69 ±1.06	1.48 ±0.14	2.72 ±1.25	2.59 ±1.25
Phvul.002G083600	Pollen allergen /Rare lipoprotein A (RlpA)-like double-psi β-barrel	68.8 ±11.82	57.44 ±14.71	16.78 ±6.39	3.84 ±1.61	38.81 ±4.27	70.09 ±4.12	9.09 ±4.43	18.04 ±8.05
Phvul.002G153900	Dehydration-responsive element-binding protein 1E-related	0 ±0	0 ±0	0 ±0	0 ±0	0.39 ±0.24	0 ±0	0.05 ±0.05	0.84 ±0.84
Phvul.003G080100	Multi-copper oxidase	3.6 ±0.81	2.1 ±0.59	1.24 ±0.25	0.22 ±0.05	5.94 ±2.17	1.51 ±0.2	0.54 ±0.23	0.87 ±0.4
Phvul.004G093200	Light-harvesting complex II chlorophyll a/b binding protein 1 (LHCB1)	53.17 ±21.56	11.06 ±3.83	19.7 ±5.46	2.23 ±0.29	80.64 ±3.96	5.44 ±2.44	26.74 ±13.12	21.01 ±16.71
Phvul.006G207900	Copper transport protein ATOX1-related	12.05 ±2.66	12.74 ±1.92	4.47 ±1.33	0.35 ±0.03	13.46 ±2.35	13.77 ±1.21	2.36 ±1.18	3.92 ±1.71
Phvul.007G196500	No functional annotation	0.38 ±0.39	0 ±0	0 ±0	0 ±0	0.54 ±0.27	0.16 ±0.16	0.12 ±0.12	0.87 ±0.28
Phvul.008G030300	No functional annotation	45.29 ±8.15	37.29 ±19.56	11.61 ±2.29	1.02 ±0.7	45.75 ±8.17	52.63 ±12.53	6.67 ±3.44	7.41 ±3.81
Phvul.008G100300	Embryo-specific protein 3	3.4 ±0.17	1.62 ±0.92	4.27 ±0.51	0.5 ±0.04	3.13 ±0.06	2.57 ±0.4	2.46 ±0.98	2.42 ±0.73

Table S9 continued

		Transcript levels (FPKM)							
		Mock inoculation				*Xap* inoculation			
**Gene ID**	**Gene Annotation**	**0 h PI**	**8 h PI**	**24 h PI**	**48 h PI**	**0 h PI**	**8 h PI**	**24 h PI**	**48 h PI**
Phvul.008G137700	Epidermal patterning factor-like protein 9	11.38 ±2.52	9.36 ±5.91	3.18 ±1.5	0.67 ±0.25	12.41 ±3.56	11.44 ±1.81	1.83 ±0.8	2.8 ±1.45
Phvul.008G248000	Expansin-A8	1.66 ±0.45	1.06 ±0.57	0.49 ±0.09	0 ±0	2.45 ±0.81	1.69 ±0.46	0.71 ±0.52	0.63 ±0.32
Phvul.008G261200	Protein ELF4-like 1	24.67 ±24.54	47.59 ±24.03	0.37 ±0.25	0 ±0	0.34 ±0.07	73.97 ±1.7	0.06 ±0.06	0.69 ±0.15
Phvul.009G187400	Gibberellin-regulated protein 4	161.56 ±64.34	193.6 ±96.9	15.6 ±7.07	2.03 ±0.92	104.6 ±4.81	269.09 ±42.92	8.67 ±4.82	9.62 ±3.6
Phvul.010G129900	Germin-like protein subfamily 3	634.04 ± 469.3	977.85 ± 497.08	51.89 ± 22.68	7.28 ± 1.77	441.73 ± 150.71	1701.17 ± 159.9	24.56 ± 10.55	34.04 ± 13.51
Phvul.011G063800	Expansin-A6	50.07 ±20.99	8.09 ±4.22	9.72 ±2.7	2.26 ±0.35	28.96 ±1.33	9.69 ±2.32	7.71 ±4.27	10.23 ±4.41
Phvul.011G209600	Zinc finger protein-related	585.72 ±142.68	250.2 ±134.06	600.85 ±34.67	91.94 ±4.43	835.51 ±40.86	365.09 ±78.45	437.27 ±209.54	407.22 ±167.51
XLOC_009121	No functional annotation	0 ±0	0.68 ±0.57	0.15 ±0.15	0.06 ±0.06	0.97 ±0.68	0.09 ±0.09	0.58 ±0.2	0.33 ±0.17
XLOC_020711	No functional annotation	0 ±0	0.54 ±0.54	1.13 ±0.57	0 ±0	0 ±0	0.46 ±0.46	1.21 ±0.62	1.89 ±0.99

Table S10. Transcript abundance for genes downregulated in the CBB-resistant RIL at 0 h post-inoculation (PI) with *Xanthomonas axonopodis* (*Xap*). Transcript levels for individual genes corresponding to each RIL/ inoculation treatment/ sampling time PI are represented as FPKM ± SE of three experimental replicates.

		Transcript levels (FPKM)							
		Mock inoculation				*Xap* inoculation			
**Gene ID**	**Gene Annotation**	**0 h PI**	**8 h PI**	**24 h PI**	**48 h PI**	**0 h PI**	**8 h PI**	**24 h PI**	**48 h PI**
Phvul.001G020100	Galactolipase	31.65 ±23.06	51.76 ±20.87	5.26 ±0.63	5.38 ±0.67	7.79 ±0.84	51.22 ±22.05	7.29 ±1.51	11.57 ±5.4
Phvul.001G033700	Cytosolic resiniferatoxin binding protein RBP-26	4.12 ±3.04	10.22 ±5.15	1.41 ±0.28	1.29 ±0.08	0.85 ±0.19	8.2 ±3.82	1.29 ±0.5	0.86 ±0.06
Phvul.001G087300	Zinc finger protein CONSTANS-like 14-related	2.03 ±1.89	3.22 ±1.54	0.1 ±0.03	0.12 ±0.04	0.42 ±0.03	3.2 ±1.35	0.24 ±0.06	0.16 ±0.04
Phvul.001G154400	Auxin canalisation	0.8 ±0.61	1.45 ±0.63	0.06 ±0.02	0.11 ±0.03	0.16 ±0.02	1.17 ±0.48	0.12 ±0.02	0.08 ±0.01
Phvul.001G156900	Galactolipase / Triacylglycerol lipase	108.88 ±83.07	117.59 ±73.13	73.66 ±28.83	41.58 ±25.8	21.21 ±11.89	85.58 ±41.27	72.25 ±9.1	61.64 ±29.15
Phvul.001G255400	SufE-like protein 2, chloroplastic	0.9 ±0.36	0.67 ±0.24	1.27 ±0.35	2.38 ±1.39	0 ±0	0.59 ±0.28	0.81 ±0.31	5.63 ±0.23
Phvul.001G263300	No functional annotation	4.52 ±3.74	7.79 ±3.06	4.82 ±1.1	11.37 ±4.4	0.9 ±0.18	7.98 ±4.1	6.57 ±0.99	10.46 ±1.76
Phvul.001G234700, Phvul.001G234800	Tropinone reductase I	8.84 ±7.03	11.74 ±4.92	2.83 ±0.29	2.53 ±0.14	2.12 ±0.42	12.97 ±5.55	2.68 ±0.25	2.59 ±0.62
Phvul.002G028500	Polyol transporter 3-related	5.23 ±4.13	5.11 ±1.61	0.43 ±0.1	0.66 ±0.2	0.82 ±0.39	5.82 ±2.42	0.72 ±0.16	1.03 ±0.11
Phvul.002G100501	LOB domain-containing protein 4	1.14 ±0.69	1.25 ±0.51	0.73 ±0.1	0.62 ±0.28	0.18 ±0.08	1.7 ±0.9	2.31 ±0.69	0.94 ±0.2
Phvul.002G168400	Glycogen phosphorylase	9.21 ±7.78	17.11 ±8.79	0.74 ±0.13	0.59 ±0.1	1.39 ±0.16	13.5 ±6.21	0.83 ±0.08	0.74 ±0.1
Phvul.002G240900	WRKY transcription factor 65-related	0.68 ±0.57	1.18 ±0.52	0.12 ±0.02	0.19 ±0.09	0 ±0	1.11 ±0.51	0.1 ±0.03	0.51 ±0.1
Phvul.002G303700	SAUR family protein	9.69 ±4.23	15.36 ±5.16	7.97 ±1.7	8.44 ±0.97	1.71 ±0.19	12.66 ±5.37	6.84 ±0.98	7.44 ±1.37

Table S10 continued

		Transcript levels (FPKM)							
		Mock inoculation				*Xap* inoculation			
**Gene ID**	**Gene Annotation**	**0 h PI**	**8 h PI**	**24 h PI**	**48 h PI**	**0 h PI**	**8 h PI**	**24 h PI**	**48 h PI**
Phvul.002G044500	PPR repeat (PPR) // PPR repeat family (PPR_2) // DYW family of nucleic acid deaminases	0.74 ±0.59	0.79 ±0.35	0.3 ±0.06	0.45 ±0.12	0.12 ±0.02	0.98 ±0.44	0.35 ±0.08	0.55 ±0.16
Phvul.003G157900	No functional annotation	4.41 ±1.61	1.53 ±0.79	9.26 ±2.11	0.75 ±0.74	0 ±0	3.77 ±1.44	5.92 ±4.83	2.48 ±0.05
Phvul.003G206400	Cytochrome P450 CYP2 subfamily	1.67 ±1.45	4.84 ±2.48	0.19 ±0.13	0.24 ±0.07	0.14 ±0.05	3.96 ±1.95	0.23 ±0.08	0.28 ±0.02
Phvul.003G255700	No functional annotation	9.62 ±8.27	11.72 ±5.87	1.07 ±0.49	2.04 ±0.57	1.32 ±0.47	12.58 ±5.67	1.49 ±0.43	1.18 ±0.4
Phvul.003G047100	Glucosyl/glucuronosyl transferases	4.06 ±3.06	4.05 ±2.33	14.77 ±4.93	5.3 ±3.07	0.46 ±0.17	4.44 ±2.47	13.2 ±2.74	3.05 ±1.29
Phvul.003G162800	Aspartate racemase	0.7 ±0.44	0.56 ±0.39	0.55 ±0.06	0.42 ±0.19	0.17 ±0.06	0.87 ±0.38	0.33 ±0.18	0.41 ±0.08
Phvul.003G219200	Bifunctional inhibitor/ Lipid-transfer protein/seed storage 2S albumin superfamily protein	1.28 ±1.02	1.57 ±0.61	1.48 ±0.78	1.44 ±0.3	0.23 ±0.07	2.45 ±1.11	3.03 ±0.32	0.91 ±0.48
Phvul.004G137600	DR4 protein-related	22.04 ±18.58	23.79 ±14.25	54.85 ±16.75	20.05 ±10.36	2.78 ±1.15	22.39 ±12.29	59.21 ±15.85	13.59 ±5.68
Phvul.004G135700	PPR repeat (PPR) // PPR repeat family (PPR_2)	1.3 ±0.87	1.51 ±0.61	0.46 ±0.05	0.49 ±0.07	0.18 ±0.02	1.4 ±0.5	0.61 ±0.13	0.93 ±0.11
Phvul.004G160800	α/β Hydrolase fold-containing protein	36.74 ±30.46	57.75 ±26.56	11.51 ±1.43	9.38 ±1.94	5.65 ±1.08	58.1 ±27.15	9.1 ±1.1	5.43 ±0.7
Phvul.005G001000	X-Box transcription factor-related	7.39 ±5.89	6.66 ±3.98	21.15 ±7.97	9.03 ±5.16	1.7 ±0.96	7.93 ±4.38	20.28 ±4.91	5.37 ±1.61
Phvul.005G032600	Dirigent protein 20-related	7.52 ±5.04	6.84 ±3.62	31.04 ±9.23	14.7 ±6.08	1.4 ±0.72	7.16 ±3.01	32.1 ±6.03	6.91 ±2.71

Table S10 continued

		Transcript levels (FPKM)							
		Mock inoculation				*Xap* inoculation			
**Gene ID**	**Gene Annotation**	**0 h PI**	**8 h PI**	**24 h PI**	**48 h PI**	**0 h PI**	**8 h PI**	**24 h PI**	**48 h PI**
Phvul.005G075200	Cathepsin H / N-benzoylarginine-β-naphthylamide hydrolase	0.85 ±0.22	0.66 ±0.16	0.82 ±0.23	0.76 ±0.21	0.16 ±0.05	0.43 ±0.12	0.66 ±0.16	0.85 ±0.11
Phvul.005G120400	PPR repeat (PPR) // PPR repeat (PPR_1)	0.88 ±0.79	1.26 ±0.61	0.38 ±0.08	0.45 ±0.14	0.2 ±0.07	1.24 ±0.59	0.56 ±0.09	0.7 ±0.14
Phvul.005G156900	Linoleate 9S-lipoxygenase	181.7 ±141.39	207.98 ±119.73	290.11 ±136.69	112.24 ±69.14	39.2 ±15.97	233.48 ±113.2 2	288.57 ±71.01	59.56 ±27.01
Phvul.005G080600	γ-Glutamyl hydrolase	15.27 ±13.03	16.13 ±10.37	6.19 ±3.51	1.4 ±0.29	1.86 ±0.33	14.45 ±8.2	4.02 ±0.96	1.24 ±0.22
Phvul.005G156800	Linoleate 9S-lipoxygenase	76.8 ±65.79	79.03 ±49.09	140.86 ±44.72	76.64 ±38.7	9.12 ±5.13	97.09 ±49.93	167.41 ±43.87	68.4 ±15.99
Phvul.003G188300, *Phvul.003G188400*	PPR repeat (PPR) // PPR repeat family (PPR_2) // Pentatricopeptide repeat domain (PPR_3)	11.17 ±9.38	17.67 ±8.12	1.55 ±0.18	1.94 ±0.08	2.06 ±0.29	21.38 ±9.73	1.82 ±0.12	1.51 ±0.04
Phvul.006G034000	UDP-glucose 4,6-dehydratase	1.88 ±1.52	1.54 ±1.04	5.05 ±1.76	2.19 ±1.28	0.17 ±0.08	1.71 ±1.11	5.31 ±0.87	2.15 ±1.26
Phvul.006G146400	Chitin elicitor receptor kinase 1	1.17 ±0.88	2.32 ±1.11	0.27 ±0.09	0.21 ±0.05	0.21 ±0.05	1.83 ±0.87	0.33 ±0.03	0.25 ±0.05
Phvul.007G017600	No functional annotation	1.15 ±0.48	0.97 ±0.57	0.19 ±0.1	0.14 ±0.08	0.24 ±0.08	0.66 ±0.27	0.16 ±0.05	0.02 ±0.02
Phvul.007G135800	Glucosyl/glucuronosyl transferases	1.39 ±1.24	2.98 ±1.57	0.17 ±0.06	0.1 ±0.02	0.2 ±0.03	1.91 ±0.81	0.24 ±0.05	0.09 ±0.03
Phvul.007G050900	Cysteine-rich receptor-like protein kinase 9-related	7.55 ±6.18	12.15 ±5.03	1.78 ±0.15	3.24 ±0.39	1.38 ±0.22	10 ±4.33	3 ±0.32	3.74 ±0.5

Table S10 continued

		Transcript levels (FPKM)							
		Mock inoculation				*Xap* inoculation			
**Gene ID**	**Gene Annotation**	**0 h PI**	**8 h PI**	**24 h PI**	**48 h PI**	**0 h PI**	**8 h PI**	**24 h PI**	**48 h PI**
Phvul.008G257300	CCT motif	17.09 ±16.99	38.71 ±19.34	0.06 ±0.04	0.06 ±0.03	0.23 ±0.04	34.57 ±17.25	0.09 ±0.09	0.05 ±0.03
Phvul.008G079600	PPR repeat (PPR) // PPR repeat (PPR_1) // Pentatricopeptide repeat domain (PPR_3) // DYW family of nucleic acid deaminases	0.66 ±0.44	1.11 ±0.55	0.4 ±0.08	0.44 ±0.13	0.08 ±0.02	0.95 ±0.43	0.34 ±0.06	0.67 ±0.02
Phvul.008G128700	β-Amyrin 24-hydroxylase / Sophoradiol 24-hydroxylase	9.04 ±7.5	8.51 ±5.32	30.75 ±11.85	8.9 ±4.92	1.61 ±1.01	10.02 ±5.48	27.86 ±5.24	3.63 ±1.55
Phvul.008G152900	Permease of the drug/metabolite transporter (DMT) superfamily	18.65 ±10.57	28.74 ±10.17	8.74 ±1.15	9.11 ±0.7	4.58 ±0.68	29.19 ±11.31	9.85 ±0.08	8.38 ±1.52
Phvul.008G211800	Niemann pick type C2 protein NPC2-related	5.22 ±5.01	5.73 ±4.5	7.79 ±4.61	3.5 ±1.74	0.23 ±0.13	5.22 ±3.03	8.87 ±1.16	1.07 ±0.6
Phvul.009G002200	Amidophosphoribosyl-transferase / Phosphoribosyldiphosphate 5-amidotransferase	39.43 ±28.42	68.34 ±28.52	13.52 ±1.68	21.89 ±1.18	8.53 ±1.25	69.99 ±31.74	16.67 ±1.44	27.26 ±1.09
Phvul.009G146800	Alginate lyase	20.59 ±17.51	45.57 ±20.52	6.15 ±0.98	9.23 ±3.94	2.62 ±0.43	43.42 ±20.38	6.53 ±0.28	14.43 ±6.4
Phvul.010G143100	Leucine-rich repeat protein kinase-related	0.97 ±0.81	1.95 ±0.96	0.13 ±0.01	0.2 ±0.03	0.12 ±0.04	1.67 ±0.74	0.15 ±0.02	0.3 ±0.03
Phvul.010G162600	DnaJ homolog subfamily C member	64.65 ±41.73	129.07 ±52.28	71.22 ±19.4	121.05 ±17.36	10.17 ±1.37	100.59 ±52.37	95.07 ±3.44	128.38 ±26.56
Phvul.011G085200	Xyloglucan:xyloglucosyl transferase	18.66 ±11.69	16.01 ±5.57	17.56 ±2.86	8.62 ±3.06	3.29 ±0.8	18.32 ±6.81	23.96 ±2.08	2.99 ±0.95
Phvul.011G125700	E3 ubiquitin-protein ligase RNF1/2	26.3 ±21.83	39.01 ±18.63	6.07 ±0.47	5.65 ±0.45	4.23 ±0.21	37.29 ±18.37	5.82 ±0.19	7.15 ±0.28

Table S10 continued

		Transcript levels (FPKM)							
		Mock inoculation				*Xap* inoculation			
**Gene ID**	**Gene Annotation**	**0 h PI**	**8 h PI**	**24 h PI**	**48 h PI**	**0 h PI**	**8 h PI**	**24 h PI**	**48 h PI**
Phvul.011G167000	Chitinase	7.32 ±7.06	6.03 ±4.42	14 ±9.98	2.99 ±2.23	0.17 ±0.1	3.48 ±1.85	9.19 ±1.77	2.54 ±0.46
Phvul.011G018000	Alcohol dehydrogenase related	7 ±5.46	15.56 ±7.1	0.77 ±0.05	0.89 ±0.13	1.61 ±0.31	12.43 ±5.21	0.93 ±0.28	0.7 ±0.16
Phvul.007G276500, Phvul.007G276600	No functional annotation	89.3 ±73.34	75.74 ±47.75	151.45 ±91.16	39.43 ±23.99	15.6 ±6.73	70.5 ±46.32	119.51 ±51.52	137.18 ±62.5
Phvul.004G129600, Phvul.004G129700, Phvul.004G129800, Phvul.004G129900, Phvul.004G130000	DR4 protein-related	25.05 ±21.45	17.98 ±9.37	28.58 ±11.88	12.56 ±7.33	1.81 ±0.96	18.56 ±9.14	32.63 ±7.77	14.44 ±6.13
Phvul.010G134900, Phvul.010G135000	Linoleate 13S-lipoxygenase / Lipoxidase	84.61 ±73.62	77.78 ±49.63	123.84 ±40.64	60.48 ±35.79	12.28 ±6.45	99.21 ±50.37	141.3 ±44.79	45.94 ±19.79
Phvul.008G228814, Phvul.008G229000, Phvul.008G229400	Proprotein convertase subtilisin/ Kexin	16.57 ±14.8	20.03 ±14.98	9.97 ±6.51	2.08 ±0.1	1.7 ±0.47	10.76 ±5.29	7.67 ±0.57	0.94 ±0.23
XLOC_015034	No functional annotation	0.83 ±0.53	0.76 ±0.39	1.25 ±0.44	1.06 ±0.96	0 ±0	0.27 ±0.17	2.48 ±0.31	2.03 ±0.28
XLOC_025196	No functional annotation	1.1 ±0.79	1.88 ±1.14	1.36 ±0.69	3.55 ±2.4	0 ±0	1.19 ±0.79	4.04 ±1.3	6.81 ±0.39

Table S11. Transcript abundance for genes downregulated in the CBB-resistant RIL at 8 h post-inoculation (PI) with *Xanthomonas axonopodis* (*Xap*). Transcript levels for individual genes corresponding to each RIL/ inoculation treatment/ sampling time PI are represented as FPKM ± SE of three experimental replicates.

		**Transcript levels (FPKM)**							
		**Mock inoculation**				***Xap* inoculation**			
**Gene ID**	**Gene Annotation**	**0 h PI**	**8 h PI**	**24 h PI**	**48 h PI**	**0 h PI**	**8 h PI**	**24 h PI**	**48 h PI**
Phvul.004G142500, Phvul.004G142600, Phvul.004G142700	Protein phosphatase 1, regulatory subunit, and related proteins; Leucine Rich Repeat (LRR_1) // Leucine rich repeat (LRR_8)	1.38 ±0.89	0.72 ±0.25	1.01 ±0.24	0.57 ±0.3	0.23 ±0.18	0.16 ±0.06	0.64 ±0.47	1.39 ±0.45
XLOC_015051	No functional annotation	1.63 ±0.84	0.84 ±0.42	1.91 ±0.23	2.8 ±0.41	0.41 ±0.21	0 ±0	2.78 ±0.35	1.78 ±0.95
XLOC_011019	No functional annotation	0.35 ±0.35	1.41 ±1.15	0.71 ±0.4	0 ±0	0.78 ±0.39	0 ±0	0.58 ±0.58	0.23 ±0.24

Table S12. Transcript abundance for genes downregulated in the CBB-resistant RIL at 24 h post-inoculation (PI) with *Xanthomonas axonopodis* (*Xap*). Transcript levels for individual genes corresponding to each RIL/ inoculation treatment/ sampling time PI are represented as FPKM ± SE of three experimental replicates.

		Transcript levels (FPKM)							
		Mock inoculation				*Xap* inoculation			
**Gene ID**	**Gene Annotation**	**0 h PI**	**8 h PI**	**24 h PI**	**48 h PI**	**0 h PI**	**8 h PI**	**24 h PI**	**48 h PI**
Phvul.003G281200	Strictosidine synthase-related	2.87 ±1.24	4.35 ±1.85	0.83 ±0.54	0.12 ±0.05	5.01 ±2.16	3.38 ±0.62	0.21 ±0.07	0.07 ±0.02
XLOC_002678	No functional annotation	0.51 ±0.26	0.67 ±0.43	2.07 ±1.72	0.34 ±0.33	0.35 ±0.35	0.81 ±0.54	0 ±0	0.45 ±0.23

Table S13. Transcript abundance for genes downregulated in the CBB-resistant RIL at 48 h post-inoculation (PI) with *Xanthomonas axonopodis* (*Xap*). Transcript levels for individual genes corresponding to each RIL/ inoculation treatment/ sampling time PI are represented as FPKM ± SE of three experimental replicates.

		Transcript levels (FPKM)							
		Mock inoculation				*Xap* inoculation			
**Gene ID**	**Gene Annotation**	**0 h PI**	**8 h PI**	**24 h PI**	**48 h PI**	**0 h PI**	**8 h PI**	**24 h PI**	**48 h PI**
Phvul.002G296700	Upstream-binding transcription factor	0.57 ±0.1	0.52 ±0.15	0.5 ±0.04	0.64 ±0.11	0.51 ±0.09	0.52 ±0.05	0.49 ±0.07	0.12 ±0.04
Phvul.005G113800	Peroxidase 3-related	4.79 ±1.66	3.25 ±0.92	2.31 ±0.97	2.22 ±0.66	7.45 ±0.64	3.56 ±1.58	1.74 ±0.28	0.46 ±0.18
Phvul.006G202700	No functional annotation	912.7 ±153.94	873.97 ±58.41	211.87 ±86.13	128.81 ±29.43	993.88 ±101.26	788.51 ±36.1	120.08 ±5.05	31.84 ±23.56
Phvul.008G037500	Pollen allergen / Rare lipoprotein A (RlpA)-like double-psi β-barrel	3.34 ±1.28	3.41 ±0.81	2.78 ±0.5	1.49 ±0.14	6.68 ±2.04	3.52 ±0.52	1.59 ±0.2	0.31 ±0.07
Phvul.008G174800	No functional annotation	0.98 ±0.4	0.69 ±0.25	0.31 ±0.05	1.44 ±0.62	1.56 ±1.26	1.19 ±0.16	0.76 ±0.16	0.17 ±0.06
Phvul.008G218500	Peroxidase 52	0.4 ±0.15	0.23 ±0.16	1.74 ±0.51	2.2 ±1.18	0.46 ±0.04	0.23 ±0.13	1.41 ±0.46	0.32 ±0.15

Table S14. Transcript abundance for genes downregulated in the CBB-susceptible RIL at 0 h post-inoculation (PI) with *Xanthomonas axonopodis* (*Xap*). Transcript levels for individual genes corresponding to each RIL/ inoculation treatment/ sampling time PI are represented as FPKM ± SE of three experimental replicates.

		Transcript levels (FPKM)							
		Mock inoculation				*Xap* inoculation			
**Gene ID**	**Gene Annotation**	**0 h PI**	**8 h PI**	**24 h PI**	**48 h PI**	**0 h PI**	**8 h PI**	**24 h PI**	**48 h PI**
Phvul.001G022200	Cold regulated protein 27	17.31 ±17.05	40.72 ±2.78	0.09 ±0.06	0.05 ±0.03	0.1 ±0.02	55.98 ±4.99	0.08 ±0.08	0.09 ±0.03
Phvul.001G124300	Pyrroline-5-carboxylate reductase	9.89 ±7.92	27.61 ±0.72	1.77 ±0.4	1.35 ±0.17	1.9 ±0.04	26.29 ±0.28	1.92 ±0.32	1.59 ±0.39
Phvul.001G130900	ATP-binding cassette transporter	2.65 ±1.63	6.01 ±0.35	1.84 ±0.14	3 ±0.69	0.56 ±0.17	5.87 ±0.73	2.29 ±0.92	2.43 ±1.11
Phvul.001G162800	Long-chain-alcohol oxidase	7.22 ±6.35	20.9 ±1.26	0.87 ±0.21	0.71 ±0.1	0.92 ±0.18	23.25 ±0.98	0.93 ±0.14	1.1 ±0.25
Phvul.001G221600	PPR repeat (PPR) / DYW family of nucleic acid deaminases	0.82 ±0.57	1.51 ±0.12	0.42 ±0.05	0.55 ±0.14	0.11 ±0.05	1.83 ±0.18	0.48 ±0.19	0.59 ±0.24
Phvul.001G227500	PPR repeat (PPR) / DYW family of nucleic acid deaminases	0.79 ±0.65	1.92 ±0.06	0.29 ±0.06	0.53 ±0.13	0.15 ±0.02	2.58 ±0.41	0.57 ±0.11	0.62 ±0.11
Phvul.001G247500	SEL1 repeat	7.09 ±5.66	18.62 ±1.01	0.71 ±0.2	0.93 ±0.21	1.4 ±0.27	19.47 ±0.29	1.01 ±0.17	1.17 ±0.16
Phvul.002G211500	Oxidoreductase, 2-oxoglutarate-Fe II oxygenase family protein	1.08 ±0.39	1.2 ±0.06	0.73 ±0.05	3.54 ±1.07	0.23 ±0.01	1.02 ±0.23	2.38 ±1.19	2.58 ±1.5
Phvul.002G283000	Polynucleotide adenylyltransferase domain and RNA recognition motif-containing protein-related	8.21 ±6.48	18.62 ±1.51	4.43 ±0.27	4.74 ±0.79	1.99 ±0.14	20.95 ±1.97	4.66 ±0.3	5.62 ±1.28
Phvul.003G004400	Nicotinamidase 3	8.07 ±5.91	22.42 ±0.76	2.56 ±0.34	2.52 ±0.21	1.84 ±0.15	28.23 ±3.21	2.37 ±0.17	2.05 ±0.28

Table S14 continued

		Transcript levels (FPKM)							
		Mock inoculation				*Xap* inoculation			
**Gene ID**	**Gene Annotation**	**0 h PI**	**8 h PI**	**24 h PI**	**48 h PI**	**0 h PI**	**8 h PI**	**24 h PI**	**48 h PI**
Phvul.003G101100	Nitrate, formate, iron dehydrogenase	3.15 ±1.93	7.36 ±1.09	2.72 ±0.92	8.34 ±1.74	0.55 ±0.16	5.49 ±1.45	8.82 ±6.33	7.69 ±4.1
Phvul.003G157500	PPR repeat (PPR) / DYW family of nucleic acid deaminases	0.57 ±0.36	1.25 ±0.12	0.3 ±0.03	0.54 ±0.04	0.13 ±0.08	1.25 ±0.18	0.35 ±0.1	0.31 ±0.06
Phvul.003G162300	PPR repeat (PPR) // PPR repeat (PPR_1) // PPR repeat family (PPR_2) // DYW family of nucleic acid deaminases	4.23 ±3.14	10.52 ±0.34	0.88 ±0.3	0.75 ±0.18	0.89 ±0.13	11.33 ±0.48	1 ±0.17	1.01 ±0.22
Phvul.003G189800	PPR repeat (PPR) // PPR repeat family (PPR_2) // DYW family of nucleic acid deaminases	1.09 ±0.77	2.27 ±0.08	0.46 ±0.07	0.81 ±0.21	0.19 ±0.03	2.29 ±0.46	0.62 ±0.2	0.52 ±0.09
Phvul.003G229500	PPR repeat (PPR) // PPR repeat family (PPR_2)	36.46 ±29.38	98.5 ±3.26	9.24 ±1.4	11.13 ±2.66	8.84 ±0.23	104.06 ±2.37	12.29 ±2.55	11.2 ±1.32
Phvul.004G047900	Proline dehydrogenase	1.12 ±0.8	2.47 ±0.38	0.57 ±0.23	1.01 ±0.46	0.24 ±0.03	2.88 ±0.1	1.11 ±0.39	0.9 ±0.13
Phvul.004G083200	Acyl-CoA N-acyltransferases superfamily protein	13.38 ±10.01	32.92 ±0.55	1.26 ±0.1	1.4 ±0.25	2.85 ±0.32	33.55 ±0.61	1.28 ±0.18	1.14 ±0.19
Phvul.004G128500	APO protein 1, chloroplastic	5.26 ±4.21	13.62 ±1.02	1.29 ±0.16	2.13 ±0.21	0.94 ±0.08	14.85 ±0.66	1.22 ±0.23	1.51 ±0.21
Phvul.004G133300	No functional annotation	1.27 ±1.11	2.8 ±0.25	0.44 ±0.04	0.6 ±0.16	0.26 ±0.01	3.38 ±0.4	0.61 ±0.22	0.65 ±0.21
Phvul.004G151100	Zinc finger protein	4.66 ±3.97	9.81 ±1.46	2.94 ±0.62	4 ±0.3	1.06 ±0.22	11.69 ±2.02	2.43 ±0.51	3.87 ±0.88
Phvul.004G151525	EF-hand calcium-binding domain containing protein	15.77 ±11.48	34.1 ±2.7	3.12 ±0.58	1.81 ±0.42	3.35 ±0.8	31.25 ±1.19	3.31 ±0.29	1.11 ±0.43

Table S14 continued

		Transcript levels (FPKM)							
		Mock inoculation				*Xap* inoculation			
**Gene ID**	**Gene Annotation**	**0 h PI**	**8 h PI**	**24 h PI**	**48 h PI**	**0 h PI**	**8 h PI**	**24 h PI**	**48 h PI**
Phvul.005G039200	Vicianin β-glucosidase	39.58 ±32.27	95.77 ±12.14	2.5 ±0.26	1.64 ±0.21	8.95 ±1.64	91.11 ±3.44	2.38 ±0.2	2.18 ±0.57
Phvul.005G124400	PPR repeat (PPR) / DYW family of nucleic acid deaminases	2.66 ±1.97	6.26 ±0.55	0.76 ±0.22	0.82 ±0.21	0.46 ±0.1	6.75 ±0.38	0.75 ±0.24	0.94 ±0.08
Phvul.005G159300	PPR repeat (PPR) // PPR repeat family (PPR_2)	1.02 ±0.83	2.16 ±0.14	0.37 ±0.02	0.64 ±0.18	0.24 ±0.08	2.07 ±0.19	0.48 ±0.08	0.53 ±0.13
Phvul.005G174500	Protein of unknown function	1.85 ±1.32	3.34 ±1.22	0.22 ±0.07	0 ±0	0.23 ±0.07	1.67 ±0.35	0.03 ±0.01	0.16 ±0.09
Phvul.006G036400	PPR repeat (PPR) /DYW family of nucleic acid deaminases	0.54 ±0.46	0.97 ±0.15	0.18 ±0.03	0.35 ±0.04	0.09 ±0.02	1.13 ±0.17	0.29 ±0.02	0.3 ±0.09
Phvul.007G093600	MLO-like protein 12-related	40.43 ±28.57	98.75 ±9.36	7.09 ±1.42	29.97 ±8.76	10.04 ±0.54	107.29 ±2.22	10.96 ±2.32	19.69 ±5.5
Phvul.007G125500	Potato inhibitor I family	1.94 ±1.94	5.7 ±0.98	5.74 ±1.54	2.48 ±0.94	0 ±0	6.01 ±0.27	5.77 ±2.23	6.06 ±2.6
Phvul.007G161300	PPR repeat (PPR) // PPR repeat family (PPR_2)	0.87 ±0.7	1.09 ±0.59	0.39 ±0.03	0.63 ±0.06	0.22 ±0.08	1.71 ±0.01	0.47 ±0.16	0.57 ±0.05
Phvul.007G169400	No functional annotation	8.21 ±6.19	13.04 ±6.55	5.07 ±0.31	7.92 ±0.62	1.27 ±0.3	20.88 ±4.23	5.91 ±1.35	6.75 ±1.26
Phvul.007G216700	No functional annotation	16.46 ±15.17	26.93 ±13.87	0.16 ±0.08	0.03 ±0.03	3.38 ±1.11	39.89 ±5.24	0.11 ±0.06	0.04 ±0.04
Phvul.007G278100	Chlorophyllase	27.09 ±23.72	57.23 ±28.8	3.33 ±0.57	3.46 ±0.4	4.32 ±0.35	84.81 ±3.71	4.39 ±0.51	2.97 ±0.39
Phvul.008G025900	PPR repeat (PPR) / DYW family of nucleic acid deaminases	4.5 ±4	9.64 ±4.88	0.62 ±0.05	0.72 ±0.03	0.45 ±0.13	13.46 ±0.95	0.65 ±0.13	0.94 ±0.09

Table S14 continued

Gene ID	Gene Annotation	Transcript levels (FPKM)							
		Mock inoculation				*Xap* inoculation			
		**0 h PI**	**8 h PI**	**24 h PI**	**48 h PI**	**0 h PI**	**8 h PI**	**24 h PI**	**48 h PI**
Phvul.008G046100	PPR repeat (PPR) // PPR repeat (PPR_1) // PPR repeat family (PPR_2)	1.05 ±0.76	1.73 ±0.88	0.71 ±0.04	1.12 ±0.19	0.24 ±0.07	2.68 ±0.28	0.89 ±0.3	0.72 ±0.17
Phvul.008G103900	Cis-zeatin *O*-glucosyltransferase	0.84 ±0.7	1.07 ±0.55	0.15 ±0.03	0.1 ±0.03	0.06 ±0.02	1.8 ±0.41	0.11 ±0.03	0.13 ±0.06
Phvul.008G105000	Mitochondrial inner membrane protease subunit 2	2.67 ±1.4	3.87 ±1.95	1.25 ±0.35	2.42 ±0.32	0.54 ±0.16	6.02 ±0.91	1.83 ±0.4	1.82 ±0.49
Phvul.008G201000	No functional annotation	0.9 ±0.26	0.15 ±0.15	0.24 ±0.12	0.38 ±0.19	0 ±0	0.26 ±0.15	0.45 ±0.14	0.3 ±0.17
Phvul.008G279800	X-box transcription factor-related	12.28 ±9.2	23.37 ±11.8	4.37 ±0.32	5.16 ±0.47	2.58 ±0.39	38.63 ±2.42	6.81 ±1.62	4.25 ±0.32
Phvul.009G004200	B-box zinc finger	1.16 ±1.02	2.37 ±1.22	0.02 ±0.02	0.02 ±0.02	0 ±0	4.36 ±0.65	0 ±0	0 ±0
Phvul.009G081600	No functional annotation	1.07 ±0.82	1.6 ±0.83	0.24 ±0.12	0 ±0	0.16 ±0.08	2.35 ±0.09	0.08 ±0.02	0.23 ±0.1
Phvul.009G159500	PPR repeat (PPR) / DYW family of nucleic acid deaminases	0.85 ±0.5	1.36 ±0.69	0.37 ±0.1	0.6 ±0.03	0.19 ±0.03	1.66 ±0.15	0.51 ±0.19	0.65 ±0.11
Phvul.009G169000	Coproporphyrinogen dehydrogenase	3.46 ±2.89	7 ±3.56	0.74 ±0.13	1.35 ±0.24	0.58 ±0.09	10.32 ±1.08	0.93 ±0.16	0.84 ±0.21
Phvul.010G077634	SCY1-like protein 1	11.07 ±8.8	19.06 ±9.69	2.67 ±0.39	2.23 ±0.23	2.18 ±0.16	29.56 ±0.83	2.79 ±0.29	2.34 ±0.42
Phvul.010G093200	PPR repeat (PPR) // PPR repeat (PPR_1) // PPR repeat family (PPR_2)	0.71 ±0.44	1.05 ±0.53	0.35 ±0.12	0.5 ±0.09	0.14 ±0.05	1.47 ±0.06	0.34 ±0.13	0.44 ±0.12
Phvul.010G102700	Domain of unknown function	5.13 ±4.51	10.01 ±5.06	0.35 ±0.09	1.21 ±0.27	0.76 ±0.23	14.42 ±0.24	0.88 ±0.18	0.67 ±0.06
Phvul.010G159900	Complement component 1	1.71 ±0.47	1.57 ±0.79	1.61 ±0.13	2.86 ±0.12	0.38 ±0.14	2.71 ±0.31	2.24 ±0.63	2.56 ±0.23

Table S14 continued

		Transcript levels (FPKM)							
		Mock inoculation				*Xap* inoculation			
**Gene ID**	**Gene Annotation**	**0 h PI**	**8 h PI**	**24 h PI**	**48 h PI**	**0 h PI**	**8 h PI**	**24 h PI**	**48 h PI**
Phvul.011G006400	PPR repeat (PPR) // PPR repeat (PPR_1) // Pentatricopeptide repeat domain (PPR_3)	0.76 ±0.61	1.22 ±0.63	0.35 ±0.1	0.3 ±0.09	0.09 ±0.05	1.89 ±0.47	0.43 ±0.17	0.31 ±0.16
Phvul.011G033200	Uncharacterized conserved protein	17.33 ±14.86	31.63 ±15.82	2.14 ±0.33	3.34 ±0.43	3.89 ±0.36	50.7 ±2.9	2.49 ±0.5	2.87 ±0.54
Phvul.011G060800	PPR repeat (PPR) // Pentatricopeptide repeat domain (PPR_3)	4.09 ±3.06	6.84 ±3.45	0.81 ±0.14	1.69 ±0.33	0.74 ±0.14	11.46 ±2.01	1.29 ±0.63	1.8 ±0.37
Phvul.011G096800	(+)-Borneol dehydrogenase	6.71 ±6.67	14.44 ±7.22	0.06 ±0.03	0.24 ±0.14	0 ±0	26.75 ±6.91	0.38 ±0.38	0.04 ±0.04
XLOC_012819	No functional annotation	1.01 ±0.25	1.85 ±1.09	0.65 ±0.07	2.14 ±1.07	0 ±0	1.63 ±0.96	0.68 ±0.39	0.72 ±0.46
XLOC_012875	No functional annotation	2.38 ±1.9	3.95 ±0.7	2.25 ±0.93	2.51 ±0.06	0.17 ±0.08	2.93 ±0.66	2.66 ±1.19	2.6 ±0.38
XLOC_022362	No functional annotation	1.08 ±0.37	1.1 ±0.55	0.39 ±0.06	0.35 ±0.14	0.12 ±0.04	2.49 ±0.26	0.61 ±0.02	0.42 ±0.17
XLOC_027366	No functional annotation	1.5 ±1.5	2.44 ±1.28	0 ±0	0 ±0	0 ±0	4.6 ±1.28	0 ±0	0 ±0

Table S15. Transcript abundance for genes downregulated in the CBB-susceptible RIL at 8 h post-inoculation (PI) with *Xanthomonas axonopodis* (*Xap*). Transcript levels for individual genes corresponding to each RIL/ inoculation treatment/ sampling time PI are represented as FPKM ± SE of three experimental replicates.

		Transcript levels (FPKM)							
		Mock inoculation				*Xap* inoculation			
**Gene ID**	**Gene Annotation**	**0 h PI**	**8 h PI**	**24 h PI**	**48 h PI**	**0 h PI**	**8 h PI**	**24 h PI**	**48 h PI**
Phvul.005G026900, *Phvul.005G026966*	No functional annotation	0 ±0	0.69 ±0.25	0 ±0	0.23 ±0.12	0 ±0	0 ±0	0.25 ±0.13	0.72 ±0.39
Phvul.005G038300	No functional annotation	2.33 ±1.57	0.76 ±0.14	0.63 ±0.63	1.26 ±0.63	1.21 ±0.61	0 ±0	0.69 ±0.37	0.46 ±0.46
Phvul.005G071400	γ-Thionin family	0.15 ±0.15	0.84 ±0.46	0 ±0	0.22 ±0.14	0.16 ±0.08	0 ±0	0.05 ±0.05	0.3 ±0.22

Table S16. Transcript abundance for genes downregulated in the CBB-susceptible RIL at 24 h post-inoculation (PI) with *Xanthomonas axonopodis* (*Xap*). Transcript levels for individual genes corresponding to each RIL/ inoculation treatment/ sampling time PI are represented as FPKM ± SE of three experimental replicates.

		Transcript levels (FPKM)							
		Mock inoculation				*Xap* inoculation			
**Gene ID**	**Gene Annotation**	**0 h PI**	**8 h PI**	**24 h PI**	**48 h PI**	**0 h PI**	**8 h PI**	**24 h PI**	**48 h PI**
Phvul.006G070100	RING-H2 finger protein ATL66	0.18 ±0.1	0 ±0	0.76 ±0.47	0.63 ±0.21	0.34 ±0.19	0.21 ±0.12	0 ±0	0.73 ±0.3
Phvul.008G028100	Hyoscyamine (6S)-dioxygenase	0.11 ±0.08	0.02 ±0.02	1.32 ±0.26	0.58 ±0.11	0.19 ±0.12	0.17 ±0.09	0.28 ±0.16	0.82 ±0.12

Table S17. Transcript abundance for genes downregulated in the CBB-susceptible RIL at 48 h post-inoculation (PI) with *Xanthomonas axonopodis* (*Xap*). Transcript levels for individual genes corresponding to each RIL/ inoculation treatment/ sampling time PI are represented as FPKM ± SE of three experimental replicates.

		**Transcript levels (FPKM)**							
		**Mock inoculation**				***Xap* inoculation**			
**Gene ID**	**Gene Annotation**	**0 h PI**	**8 h PI**	**24 h PI**	**48 h PI**	**0 h PI**	**8 h PI**	**24 h PI**	**48 h PI**
Phvul.001G246300	Abscisic acid receptor PYL5	0.02 ±0.02	0.27 ±0.27	0.83 ±0.39	2.15 ±2.01	0 ±0	0.14 ±0.05	2.12 ±1.55	0.3 ±0.12
Phvul.003G030000	Protein kinase-like protein	2.29 ±0.91	0.56 ±0.06	1.02 ±0.1	8.16 ±3.2	1.35 ±0.12	0.63 ±0.12	1.81 ±0.51	2 ±0.73
Phvul.006G001500	Leucine Rich Repeat (LRR_1) / Leucine rich repeat N-terminal domain (LRRNT_2) / Leucine rich repeat (LRR_8)	2.14 ±0.87	0.86 ±0.2	0.74 ±0.03	4.48 ±2.45	1.81 ±0.1	0.86 ±0.03	0.85 ±0.18	1.1 ±0.55
Phvul.006G151500	Glucose-6-phosphate/phosphate translocator 1, chloroplastic-related	0.57 ±0.24	0.48 ±0.26	0.64 ±0.06	5.74 ±4.98	1.62 ±0.46	0.36 ±0.08	0.59 ±0.14	1.13 ±0.47
Phvul.007G049900	Cysteine-rich receptor-like protein kinase 28-related	0.44 ±0.06	0.3 ±0.06	0.12 ±0.04	1.53 ±0.94	0.3 ±0.08	0.2 ±0.08	0.23 ±0.1	0.26 ±0.06
Phvul.009G133500	Expressed protein	6.4 ±2.13	4.3 ±2.19	1.48 ±0.2	12.9 ±6.97	2.25 ±0.48	5.3 ±0.45	2.49 ±1.52	2.53 ±1.18
XLOC_020687	No functional annotation	0.26 ±0.2	0.1 ±0.1	0.03 ±0.03	0.61 ±0.61	0.02 ±0.02	0.01 ±0.01	0 ±0	0 ±0

Table S18. Transcript abundance for genes downregulated in the CBB-resistant RIL and the CBB-susceptible RIL at 0 h post-inoculation (PI) with *Xanthomonas axonopodis* (*Xap*). Transcript levels for individual genes corresponding to each RIL/treatment/sampling time PI are represented as FPKM ± SE of three experimental replicates.

		**Transcript levels (FPKM)**							
		**Mock inoculation**				***Xap* inoculation**			
**Gene ID**	**Gene Annotation**	**0 h PI**	**8 h PI**	**24 h PI**	**48 h PI**	**0 h PI**	**8 h PI**	**24 h PI**	**48 h PI**
**CBB-resistant RIL**									
Phvul.001G085200	*A. thaliana* mRNA (ORF19) from chromosome 3	673.81 ±649.02	1421.87 ±692.45	50.54 ±7.56	102.02 ±31.39	25.22 ±3.08	1127.76 ±565.04	89.94 ±7.03	76.61 ±34.87
Phvul.001G220100	Cathepsin B / Cathepsin B1	38.09 ±30.52	72.69 ±32.65	8.44 ±0.7	11.63 ±1.79	7.81 ±0.54	64.64 ±29.19	10.46 ±0.36	14.33 ±1.88
Phvul.001G226300	DnaJ homolog subfamily C member	35.58 ±34.81	67.28 ±32.78	1.72 ±0.07	2.06 ±0.2	1 ±0.16	62.46 ±30.26	1.99 ±0.2	1.97 ±0.1
Phvul.001G242900	Protein early flowering 4	129.46 ±127.77	197.2 ±103.54	2.89 ±0.8	2.92 ±0.65	3.13 ±0.16	255.71 ±130.32	3.07 ±0.18	5.29 ±0.5
Phvul.002G018400	CGI-141-related/lipase containing protein	17.92 ±14.52	30.68 ±14.25	2.79 ±0.1	2.49 ±0.33	3.23 ±0.11	33.13 ±15.32	2.75 ±0.51	4.05 ±0.96
Phvul.002G060000	F5O11.5	1.6 ±1.26	3.46 ±1.2	0.62 ±0.09	1.02 ±0.06	0.28 ±0.17	3.07 ±0.98	0.88 ±0.06	0.8 ±0.17
Phvul.002G084700	PPR repeat (PPR) / DYW family of nucleic acid deaminases	1.25 ±0.98	1.97 ±0.99	0.49 ±0.08	0.7 ±0.17	0.3 ±0.03	2.44 ±0.89	0.78 ±0.18	0.75 ±0.03
Phvul.002G093000	DnaJ homolog subfamily C member	11.35 ±10.95	24.98 ±12.45	0.27 ±0.14	0.42 ±0.06	1.11 ±0.36	25.82 ±12.51	0.26 ±0.04	0.46 ±0.15
Phvul.002G096000	Phosphoglucomutase (α-D-glucose-1,6-bisphosphate-dependent) /	51.69 ±47.49	106.02 ±50.3	3.51 ±1.21	2.7 ±0.66	5.14 ±1.47	92.61 ±43.17	4.52 ±0.29	1.96 ±0.79
Phvul.002G110800	Histone H1	54.71 ±36.73	96.22 ±40.15	44.42 ±12.82	75.18 ±8.01	13.22 ±2.49	72.9 ±27.52	54.05 ±6.75	66.25 ±0.73

Table S18 continued

		Transcript levels (FPKM)							
		Mock inoculation				*Xap* inoculation			
**Gene ID**	**Gene Annotation**	**0 h PI**	**8 h PI**	**24 h PI**	**48 h PI**	**0 h PI**	**8 h PI**	**24 h PI**	**48 h PI**
Phvul.002G111900	Ferulate-5-hydroxylase	6.47 ±5.44	10.98 ±4.94	2.43 ±0.27	2.72 ±0.43	1.16 ±0.23	10.15 ±4.24	3.11 ±0.42	3.21 ±0.74
Phvul.002G120500	Pentatricopeptide repeat-containing protein	3.3 ±3.04	4.93 ±2.51	0.98 ±0.2	1.63 ±0.54	0.45 ±0.05	4.87 ±2.48	1.18 ±0.14	2.11 ±0.5
Phvul.002G213300	Nicotinamidase	25.61 ±22.74	50.22 ±24.07	5.53 ±0.87	12.77 ±3.23	2.87 ±0.59	38.29 ±17.51	10.34 ±1.3	14.05 ±4.65
Phvul.002G234400	Bifunctional inhibitor/lipid-transfer protein/seed storage 2S albumin superfamily protein	22.73 ±19.38	45.78 ±21.63	5.09 ±0.89	5.31 ±0.48	4.8 ±0.55	44.88 ±20.41	6.37 ±1.03	9.32 ±1.41
Phvul.002G263100	No functional annotation	22.45 ±21.56	59.19 ±29.5	1.22 ±0.55	2.14 ±0.96	1.82 ±0.8	56.76 ±29.67	1.26 ±0.18	1.04 ±0.32
Phvul.002G309600	NADP-dependent malic enzyme 1	38.45 ±30.51	68.07 ±29.46	3.79 ±0.2	3.8 ±0.32	7.35 ±0.61	66.17 ±28.94	4.03 ±0.05	5.45 ±0.91
Phvul.002G314000, Phvul.002G314100	Aldehyde dehydrogenase (NAD(P)$^{(+)}$)	21.06 ±16.07	30.24 ±11.82	6.98 ±1.49	12.96 ±2.09	4.39 ±1.17	27.98 ±11.11	10.36 ±0.42	20.64 ±3.49
Phvul.002G321800	Flavin-binding Kelch repeat F-box protein 1 (FKF1)	15.55 ±15.12	33.39 ±16.58	0.31 ±0.09	0.43 ±0.12	0.55 ±0.08	28.49 ±14.03	0.26 ±0.03	0.35 ±0.05
Phvul.002G326700	Multidrug resistance protein	36.15 ±33.95	70.22 ±34.17	1.4 ±0.07	0.81 ±0.31	2.62 ±0.7	66.33 ±32.06	1.69 ±0.17	1.08 ±0.18
Phvul.003G004300	Molecular chaperone (DnaJ superfamily)	155.73 ±154.2	300.71 ±151.93	0.57 ±0.25	0.56 ±0.08	1.04 ±0.25	292.06 ±145.9	0.51 ±0.05	0.62 ±0.12
Phvul.003G022000	Clathrin assembly protein	11.24 ±9.98	21.32 ±10.28	1.34 ±0.18	1.56 ±0.24	1.2 ±0.24	21 ±9.95	1.59 ±0.13	3.83 ±0.33

Table S18 continued

Gene ID	Gene Annotation	Transcript levels (FPKM)							
		**Mock inoculation**				***Xap* inoculation**			
**Gene ID**	**Gene Annotation**	**0 h PI**	**8 h PI**	**24 h PI**	**48 h PI**	**0 h PI**	**8 h PI**	**24 h PI**	**48 h PI**
Phvul.003G044600	Glutamate formimidoyltransferase	5.56 ±4.72	10.78 ±4.86	0.43 ±0.1	0.52 ±0.04	0.83 ±0.14	9.2 ±4.27	0.55 ±0.2	1.32 ±0.55
Phvul.003G124400	Genomic DNA, chromosome 3, P1 clone: MKP6	6.95 ±3.91	13.64 ±3.91	2.45 ±0.21	3.94 ±0.61	1.19 ±0.46	10.09 ±4.73	3.87 ±1.01	5.04 ±1.56
Phvul.003G131400	Germin-like protein subfamily 3	99.16 ±73.9	170.39 ±71.85	0.63 ±0.22	0.35 ±0.08	22.87 ±4.83	222.61 ±104.5	0.41 ±0.13	0.17 ±0.1
Phvul.003G142200	Delta(4)-3-oxosteroid 5-β-reductase	10.54 ±8.09	11.41 ±5.17	1.06 ±0.61	0.72 ±0.46	2.52 ±0.16	13.57 ±6.2	0.76 ±0.18	0.23 ±0.17
Phvul.003G148400	Allergen V5/TPX-1-related family protein-related	23.87 ±20.45	47.11 ±21.25	2.29 ±0.15	2.19 ±0.63	3.26 ±0.52	39.94 ±20.03	4.28 ±0.82	1.29 ±0.25
Phvul.003G198500	RNA-dependent RNA polymerase 2	1.54 ±1.22	2.64 ±1.06	0.8 ±0.02	0.69 ±0.14	0.34 ±0.03	2.03 ±0.79	0.68 ±0.04	0.81 ±0.06
Phvul.003G209600	β-Lactamase / Penicillinase	14.18 ±11.8	24.69 ±11.56	2.38 ±0.48	2.44 ±0.4	3.14 ±0.35	24.46 ±10.62	2.91 ±0.35	2.54 ±0.15
Phvul.003G249200	No functional annotation	4.39 ±3.43	8.39 ±3.65	2.02 ±0.18	2.15 ±0.5	0.92 ±0.22	7.58 ±3.01	2.14 ±0.2	2.38 ±0.49
Phvul.003G295900	No functional annotation	[illegible]17.81 ± 116.61	266.35 ±133	1.88 ±0.23	3.69 ±1.03	1.73 ±0.19	224.45 ±116.72	2.91 ±0.94	2.76 ±1
Phvul.004G079500	NDH-dependent cyclic electron flow 5	79.39 ±78.23	165.6 ±81.94	1.85 ±0.36	1.69 ±0.33	2.66 ±0.75	151.29 ±73.91	1.94 ±0.2	1.12 ±0.23
Phvul.004G082300	Tyrosine-specific transport protein	11.46 ±9.73	22.22 ±9.87	1.22 ±0.13	1.42 ±0.31	2.61 ±0.51	20.69 ±9.15	1.31 ±0.16	0.97 ±0.18
Phvul.004G088300	Gigantea	23.93 ±23.16	47.96 ±23.68	0.67 ±0.12	0.74 ±0.13	0.67 ±0.12	43.8 ±21.39	0.59 ±0.06	0.61 ±0.09
Phvul.004G106700	Protein of unknown function	171.12 ±161.43	346.79 ±168.52	2.44 ±0.42	2.47 ±0.59	11.26 ±2.33	328.87 ±158.55	2.72 ±0.28	1.78 ±0.51

Table S18 continued

		Transcript levels (FPKM)							
		Mock inoculation				*Xap* inoculation			
**Gene ID**	**Gene Annotation**	**0 h PI**	**8 h PI**	**24 h PI**	**48 h PI**	**0 h PI**	**8 h PI**	**24 h PI**	**48 h PI**
Phvul.004G114700	Leucine Rich Repeat (LRR_1) // Leucine rich repeat N-terminal domain (LRRNT_2)	1.92 ±1.77	4.08 ±1.98	0.12 ±0.02	0.36 ±0.16	0.18 ±0.04	4.03 ±1.97	0.19 ±0.07	1.06 ±0.28
Phvul.004G119400	No functional annotation	5.11 ±5.11	6.71 ±3.87	0.06 ±0.06	0.03 ±0.03	0 ±0	7.63 ±4	0 ±0	0.03 ±0.03
Phvul.004G133000	Member of 'GDXG' family of lipolytic enzymes	5.21 ±4.28	9.2 ±4.02	0.5 ±0.06	0.82 ±0.3	0.63 ±0.06	8.65 ±3.94	0.5 ±0.11	0.37 ±0.09
Phvul.005G032700	Dirigent protein 20-related	4.95 ±4.02	6.82 ±3.51	4.28 ±0.99	2.44 ±1.24	0.88 ±0.41	6.83 ±2.72	4.83 ±0.65	2.5 ±0.5
Phvul.005G104600	Expansin-B1-related	19.78 ±12.81	30.7 ±11.32	1.64 ±0.81	0.83 ±0.21	4.88 ±0.44	30.78 ±13.32	1.27 ±0.29	0.43 ±0.13
Phvul.005G174000	ATP:ADP antiporter, AAA family	75.56 ±68.63	148.25 ±69.69	4.85 ±0.7	9.78 ±4.25	8.3 ±0.88	151.45 ±71.25	6.78 ±1.08	5.7 ±2.98
Phvul.006G063800	No functional annotation	127.94 ±127.13	259.59 ±130.39	0.38 ±0.05	0.5 ±0.02	0.81 ±0.23	243.41 ±121.35	0.67 ±0.2	0.46 ±0.08
Phvul.006G064100	α-Glucan phosphorylase 2, cytosolic	63.6 ±58.48	130.93 ±62.67	4.14 ±0.18	4.04 ±0.31	5.38 ±0.32	124.56 ±59.83	4.41 ±0.11	3.42 ±0.16
Phvul.006G065300	Phosphomethyl-pyrimidine synthase	420.23 ±409.6	791.06 ±401.32	8.43 ±1.11	7.22 ±0.52	11.08 ±1.84	735.82 ±363.26	9.17 ±0.79	5.64 ±0.85
Phvul.006G067600	Ring finger protein 41, 151	6.45 ±4.79	12.46 ±5.42	2.38 ±0.77	3.97 ±0.85	1.6 ±0.12	10.3 ±4.77	3.14 ±0.62	3.39 ±1.3
Phvul.006G117800	Pre-mRNA 3'-end-processing factor FIP1	0.85 ±0.8	1.4 ±0.66	0.26 ±0.03	0.29 ±0.02	0.2 ±0.06	1.19 ±0.5	0.34 ±0.04	0.39 ±0.12
Phvul.006G146300	Chaperonin-like RBCX protein	74.85 ±73.93	157.04 ±78.76	0.09 ±0.05	0 ±0	0.5 ±0.2	134.64 ±67.26	0.26 ±0.11	0.14 ±0.08

Table S18 continued

		Transcript levels (FPKM)							
		Mock inoculation				*Xap* inoculation			
**Gene ID**	**Gene Annotation**	**0 h PI**	**8 h PI**	**24 h PI**	**48 h PI**	**0 h PI**	**8 h PI**	**24 h PI**	**48 h PI**
Phvul.006G151300, Phvul.006G151400	β-Glucosidase 45-related	2.2 ±1.96	3.76 ±1.71	0.77 ±0.23	0.98 ±0.08	0.31 ±0.08	3.72 ±1.62	0.86 ±0.09	1.5 ±0.11
Phvul.006G168800	Proprotein convertase subtilisin/Kexin	2.32 ±2.19	4.33 ±2	0.28 ±0.03	0.26 ±0.01	0.16 ±0.05	4.2 ±1.99	0.32 ±0.03	0.37 ±0.02
Phvul.006G183600	Dormancy/auxin associated protein (Auxin_repressed)	747.6 ±677.17	1359.88 ±644.52	178.44 ±28.08	371.03 ±80.11	70.19 ±14.64	1089.32 ±506.99	334.91 ±43.67	414.93 ±126.65
Phvul.006G207600	DnaJ homolog subfamily C member	6.39 ±6.23	13.67 ±6.84	0.08 ±0.08	0.21 ±0.12	0.32 ±0.15	12.22 ±6.8	0.18 ±0.04	0.14 ±0.06
Phvul.007G017500	Major facilitator protein	37.19 ±33.19	64.76 ±31.02	2.9 ±0.68	2.29 ±0.22	3.78 ±0.12	66.54 ±31.23	2.93 ±0.16	3.36 ±0.35
Phvul.007G021100	PPR repeat (PPR) // PPR repeat family (PPR_2)	26.08 ±26.02	54.9 ±27.38	0.02 ±0.01	0.03 ±0.02	0.15 ±0.02	50.46 ±25.12	0.04 ±0.02	0.04 ±0.02
Phvul.007G027200	B-box zinc finger (ZF-B_box) // CCT motif (CCT)	15.39 ±15.36	30.91 ±15.39	0.05 ±0.01	0.06 ±0.01	0.07 ±0.03	34.99 ±18.43	0.04 ±0.01	0.05 ±0.02
Phvul.007G042100	PPR repeat (PPR) // PPR repeat family (PPR_2)	0.76 ±0.72	1.01 ±0.5	0.31 ±0.02	0.46 ±0.12	0.11 ±0.03	1.01 ±0.46	0.26 ±0.13	0.51 ±0.1
Phvul.007G074500	Protein REVEILLE 3-related	0.9 ±0.66	2.52 ±1.18	0.33 ±0.06	0.27 ±0.09	0.1 ±0.01	1.74 ±0.62	0.2 ±0.03	0.14 ±0.03
Phvul.007G090100	Isoliquiritigenin 2'-O-methyltransferase	3.97 ±3.54	10.8 ±4.91	0.59 ±0.03	0.53 ±0.16	0.79 ±0.27	8.71 ±3.83	0.72 ±0.07	0.48 ±0.23
Phvul.007G091400	No functional annotation	26.15 ±21.24	48.38 ±22.07	3.42 ±0.33	2.15 ±0.55	5.11 ±0.86	44.15 ±18.28	2.75 ±0.18	1.81 ±0.61

Table S18 continued

		Transcript levels (FPKM)							
		Mock inoculation				*Xap* inoculation			
**Gene ID**	**Gene Annotation**	**0 h PI**	**8 h PI**	**24 h PI**	**48 h PI**	**0 h PI**	**8 h PI**	**24 h PI**	**48 h PI**
Phvul.007G135000	PPR repeat (PPR) // PPR repeat family (PPR_2)	2.08 ±1.67	3.24 ±1.52	0.69 ±0.1	1 ±0.16	0.41 ±0.1	2.9 ±1.24	0.86 ±0.12	0.95 ±0.17
Phvul.007G146900	PPR repeat (PPR) / DYW family of nucleic acid deaminases	0.7 ±0.55	0.96 ±0.51	0.23 ±0.03	0.67 ±0.24	0.09 ±0.07	0.91 ±0.42	0.46 ±0.13	0.65 ±0.14
Phvul.007G150700	Nucleolin	659.01 ±522.58	1095.81 ±480.8	52.02 ±15.09	49.85 ±9.26	147.75 ±23.13	1188.61 ±518.24	45.37 ±6.63	37.61 ±4.16
Phvul.007G214800	Drought responsive ATP-binding motif containing protein	132.58 ±130.8	227.74 ±114.62	0.63 ±0.14	0.83 ±0.16	1.82 ±0.19	248.29 ±123.38	0.6 ±0.11	0.39 ±0.09
Phvul.008G003500	Protein NRT1/ PTR family 6.1	43.52 ±31.97	82.4 ±35.48	2.49 ±0.76	1.98 ±0.18	10.37 ±0.43	76 ±32.89	2.6 ±0.32	1.56 ±0.49
Phvul.008G048400	PPR repeat (PPR) // PPR repeat family (PPR_2)	3.89 ±3.7	8.92 ±4.36	0.58 ±0.03	0.73 ±0.18	0.22 ±0.06	7.6 ±3.69	0.6 ±0.11	0.98 ±0.27
Phvul.008G148100	F-box associated	9.18 ±7.01	19.85 ±8.64	2.08 ±0.59	4.26 ±0.73	1.4 ±0.47	17.56 ±8.07	3.49 ±0.41	4.89 ±1.21
Phvul.008G222000	Outer membrane protein insertion porin family	3.95 ±3.5	8.5 ±4.12	0.34 ±0.04	0.46 ±0.12	0.69 ±0.18	8.15 ±3.87	0.35 ±0.02	0.52 ±0.16
Phvul.008G225600	No functional annotation	11.27 ±10.94	24.89 ±12.45	0.32 ±0.05	0.52 ±0.23	0.43 ±0.08	23.28 ±12.34	0.46 ±0.03	0.31 ±0.08
Phvul.008G264700	Heat shock protein 42	377.21 ±375.45	755.61 ±381.03	3.84 ±0.14	4.27 ±0.64	2.27 ±0.57	632.39 ±314.98	3.17 ±0.51	3.35 ±0.4
Phvul.009G013600	Cathepsin L	319.35 ±293.42	620.61 ±296.82	35.85 ±2.68	45.18 ±3.62	25.05 ±3.35	520.06 ±244.94	43.82 ±2.85	75.51 ±7.52

Table S18 continued

		Transcript levels (FPKM)							
		Mock inoculation				*Xap* inoculation			
**Gene ID**	**Gene Annotation**	**0 h PI**	**8 h PI**	**24 h PI**	**48 h PI**	**0 h PI**	**8 h PI**	**24 h PI**	**48 h PI**
Phvul.009G023700	RNA recognition motif	337.38 ± 258.23	632.93 ±273.95	102.56 ±4.8	119.21 ±2	71.38 ±5.34	570.89 ±248.86	96.69 ±1.24	108.65 ±0.56
Phvul.009G045000	Pseudo-response regulator 5	38.36 ±36.7	78.8 ±38.74	2.48 ±0.31	2.54 ±0.73	1.73 ±0.25	64.07 ±31.22	2.22 ±0.25	1.76 ±0.35
Phvul.009G090600	No functional annotation	4.16 ±4.16	11.68 ±5.84	0 ±0	0.19 ±0.11	0.21 ±0.16	9.58 ±4.63	0.14 ±0.07	0.24 ±0.04
Phvul.009G152200	Filament-like plant protein 3	15.39 ±12.56	30.53 ±13.61	3.18 ±0.51	4.2 ±1.07	3.68 ±0.49	27.16 ±11.82	4.75 ±0.24	3.89 ±1.27
Phvul.009G200900	Protein CHUP1, chloroplastic	13.33 ±12.42	29.49 ±14.06	0.67 ±0.21	0.62 ±0.2	1.69 ±0.36	26.17 ±12.42	0.73 ±0.08	0.41 ±0.1
Phvul.009G227100	Cathepsin H	1199.97 ±1047.1	2176.12 ±1000.87	382.64 ±59.87	527.25 ±40.82	185 ±36.4	1957.83 ±870.98	530.82 ±39.68	786.79 ±103.79
Phvul.009G227800	Germin-like protein subfamily 3	305.85 ±284.5	675.53 ±330.61	0.81 ±0.15	0.73 ±0.32	26.41 ±5.02	656.26 ±314.65	0.67 ±0.11	0.34 ±0.07
Phvul.009G257000	Serine/threonine kinase	1.16 ±0.71	0.82 ±0.34	0.32 ±0.09	0.5 ±0.06	0.2 ±0.08	1.32 ±0.53	0.17 ±0.05	0.54 ±0.24
Phvul.010G023700	F21O3.2 protein-related	122.77 ±114.18	258.19 ±123.99	10.51 ±2.75	16.12 ±3.97	9.41 ±1.45	228.36 ±109.62	12.77 ±2.29	13.68 ±4.26
Phvul.010G097300	Bestrophin, RFP-TM, chloride channel	8.25 ±6.43	16.59 ±7.81	2.56 ±0.33	2.97 ±0.23	1.36 ±0.14	13.53 ±6.21	2.68 ±0.35	2.81 ±0.35
Phvul.010G142600	IAA-amino acid hydrolase ILR1-like 1-related	14.47 ±12.34	25.98 ±11.34	3.45 ±0.36	3.19 ±0.37	2.34 ±0.43	27.24 ±12.42	3.67 ±0.78	3.89 ±0.41
Phvul.010G156300	Post-illumination chlorophyll fluorescence increase protein	436.85 ±403.16	856.03 ±411.4	28.36 ±4.08	21.94 ±0.68	38.45 ±6.62	769.96 ±365.62	25.71 ±3.02	16.25 ±2.07

Table S18 continued

		Transcript levels (FPKM)							
		Mock inoculation				*Xap* inoculation			
**Gene ID**	**Gene Annotation**	**0 h PI**	**8 h PI**	**24 h PI**	**48 h PI**	**0 h PI**	**8 h PI**	**24 h PI**	**48 h PI**
Phvul.010G164200	Bactericidal permeability-increasing BPI protein-related	8.72 ±7.08	17.53 ±7.85	1.88 ±0.12	2.32 ±0.17	1.47 ±0.13	15.58 ±7.05	1.87 ±0.18	2.66 ±0.35
Phvul.011G045100	Transcription termination factor family protein	9.7 ±8.15	17.84 ±8.09	2.26 ±0.39	1.92 ±0.25	2.28 ±0.05	16.48 ±6.95	2.01 ±0.35	1.47 ±0.17
Phvul.011G050000	Expressed protein	117.31 ±113.92	236.62 ±116.38	3.4 ±0.53	5.45 ±0.84	2.13 ±0.62	197.02 ±97.47	5.4 ±0.91	4.97 ±0.93
Phvul.011G056500	Protein C13C4.7	854.87 ±748.9	1685.46 ±856.19	61.81 ±30.64	30.85 ±13.61	95.55 ±12.36	1542.9 ±722.81	85.04 ±30.12	100.18 ±47.3
Phvul.011G056900	Lysine-specific demethylase 8	11.31 ±11.18	21.71 ±10.78	0.24 ±0.02	0.36 ±0.22	0.28 ±0.12	17.73 ±8.81	0.19 ±0.02	0.27 ±0.08
Phvul.011G062100	MYB-like DNA-binding domain	7.93 ±7.79	13.6 ±6.74	0.31 ±0.1	0.13 ±0.05	0.19 ±0.07	14.21 ±6.84	0.41 ±0.11	0.48 ±0.1
Phvul.011G090500	Nucleobase-ascorbate transporter 12	4.02 ±3.41	9.53 ±4.49	0.53 ±0.05	0.45 ±0.03	0.9 ±0.24	8.59 ±3.97	0.49 ±0.1	0.37 ±0.04
Phvul.011G138600	Anthocyanidin 3-*O*-glucoside 2''-*O*-glucosyltransferase	9.51 ±9.13	20.42 ±10.01	0.08 ±0.05	0.2 ±0.05	0.52 ±0.1	17.17 ±8.19	0.28 ±0.12	0.1 ±0.01
Phvul.011G163100	Methyltransferase-like protein 20	3.23 ±2.74	5.06 ±2.64	0.46 ±0.09	0.62 ±0.19	0.44 ±0.04	6.71 ±3.04	0.52 ±0.08	0.54 ±0.07
XLOC_006186	No functional annotation	1.22 ± 0.45	1.13 ± 0.21	1.16 ± 0.55	1.25 ± 0.14	0 ± 0	2.92 ± 2	2.86 ± 1.25	4.6 ± 2.78

Table S18 continued

		Transcript levels (FPKM)							
		Mock inoculation				*Xap* inoculation			
**Gene ID**	**Gene Annotation**	**0 h PI**	**8 h PI**	**24 h PI**	**48 h PI**	**0 h PI**	**8 h PI**	**24 h PI**	**48 h PI**
**CBB-susceptible RIL**									
Phvul.001G085200	*A. thaliana* mRNA (ORF19) from chromosome iii	935.03 ±885.18	2944.13 ±182.69	63.59 ±7.86	55.38 ±27.46	52.52 ±13.07	3098.13 ±77.18	132.07 ±33.56	70.5 ±13.14
Phvul.001G220100	Cathepsin B / Cathepsin B1	41.42 ±32.81	122.34 ±9.35	8.51 ±0.59	8.9 ±0.96	7.48 ±0.93	122.03 ±5.12	9.93 ±1.44	7.81 ±0.7
Phvul.001G226300	DnaJ homolog subfamily C member	24.86 ±23.39	76.17 ±4	0.56 ±0.18	0.77 ±0.16	0.55 ±0.16	77.9 ±2.37	0.67 ±0.04	0.56 ±0.1
Phvul.001G242900	Protein early flowering 4	122.31 ±116.77	311.07 ±17.44	5.13 ±0.46	4.99 ±1.16	2.32 ±0.42	389.03 ±43.28	5.36 ±0.39	3.96 ±0.5
Phvul.002G018400	CGI-141-related/lipase containing protein	20.13 ±17.49	54.52 ±1.13	2.3 ±0.3	2.17 ±0.18	3.05 ±0.17	60.75 ±1.07	2.51 ±0.29	2.28 ±0.21
Phvul.002G060000	F5O11.5	1.74 ±1.25	3.73 ±0.29	0.7 ±0.18	0.66 ±0.03	0.37 ±0.27	4.09 ±0.75	0.97 ±0.1	0.86 ±0.16
Phvul.002G084700	PPR repeat (PPR)/ DYW family of nucleic acid deaminases	1 ±0.88	2.37 ±0.25	0.43 ±0.11	0.63 ±0.06	0.19 ±0.03	3.08 ±0.32	0.63 ±0.14	0.55 ±0.28
Phvul.002G093000	DnaJ homolog subfamily C member	11.38 ±11.18	34.68 ±0.51	0.2 ±0.06	0.11 ±0.03	0.27 ±0.08	37.1 ±1.66	0.14 ±0.08	0.2 ±0.06
Phvul.002G096000	Phosphoglucomutase	45.04 ±42.34	146.65 ±9.64	1.07 ±0.23	1.25 ±0.32	3.91 ±1.02	147.06 ±4.52	1.91 ±0.47	1.76 ±0.05
Phvul.002G110800	Histone H1	75.93 ±58.69	200.11 ±8.89	25.55 ±1.65	47.53 ±9.6	11.28 ±2.69	186.45 ±8.46	43.79 ±13.25	33.29 ±8.39
Phvul.002G111900	Ferulate-5-hydroxylase	6.78 ±5.76	17.19 ±0.53	2.77 ±0.49	3.14 ±0.35	1.25 ±0.38	19.06 ±1.25	3.11 ±0.22	2.98 ±0.25
Phvul.002G120500	Pentatricopeptide repeat-containing protein	2.91 ±2.67	8.16 ±0.49	0.84 ±0.14	1.5 ±0.18	0.38 ±0.07	9.4 ±0.8	1.27 ±0.35	1.07 ±0.44

Table S18 continued

		Transcript levels (FPKM)							
		Mock inoculation				*Xap* inoculation			
**Gene ID**	**Gene Annotation**	**0 h PI**	**8 h PI**	**24 h PI**	**48 h PI**	**0 h PI**	**8 h PI**	**24 h PI**	**48 h PI**
Phvul.002G213300	Nicotinamidase / Nicotine deamidase	29.79 ±27.15	88.23 ±14.82	3.22 ±0.36	7.44 ±1.72	1.97 ±0.6	93.3 ±1.99	10.02 ±3.72	5.93 ±0.82
Phvul.002G234400	Bifunctional inhibitor/lipid-transfer protein/seed storage 2S albumin superfamily protein	21.53 ±18.42	65 ±4.38	3.33 ±0.58	5.42 ±1.4	4.28 ±0.37	65.04 ±2.89	5.71 ±1.48	5.46 ±0.79
Phvul.002G263100	No functional annotation	23.71 ±22	69.17 ±2.63	1.31 ±0.43	1.17 ±0.24	1.4 ±0.19	80.84 ±3.27	0.85 ±0.02	0.72 ±0.23
Phvul.002G309600	NADP-dependent malic enzyme 1	36.83 ±29.7	96.67 ±3.23	3.74 ±0.25	3.35 ±0.18	8.48 ±0.54	99.21 ±1.24	3.97 ±0.64	3.42 ±0.28
Phvul.002G314000, *Phvul.002G314100*	Aldehyde dehydrogenase (NAD(P)($^+$))	17.27 ±12.96	43.69 ±2.83	5.03 ±0.38	11.72 ±0.33	2.86 ±0.41	41.69 ±3.94	11.09 ±4.89	7.69 ±2.24
Phvul.002G321800	Flavin-binding kelch repeat F-box protein 1	18.12 ±17.59	51.53 ±1.5	0.09 ±0.02	0.14 ±0.02	0.19 ±0.06	51.07 ±2.45	0.16 ±0.06	0.08 ±0.02
Phvul.002G326700	Multidrug resistance protein	32 ±30.11	97.94 ±7.52	0.89 ±0.09	0.96 ±0.31	1.65 ±0.53	104.43 ±4.41	0.86 ±0.11	0.92 ±0.12
Phvul.003G004300	Molecular chaperone (DnaJ superfamily)	118.59 ±115.54	353.86 ±14.75	0.81 ±0.22	1.3 ±0.39	1.59 ±0.24	436.85 ±38.77	0.65 ±0.05	0.81 ±0.14
Phvul.003G022000	Clathrin assembly protein	10.92 ±9.31	29.33 ±1.26	0.97 ±0.21	2.52 ±0.68	1.61 ±0.2	32.74 ±4.51	1.35 ±0.31	1.3 ±0.05
Phvul.003G044600	Glutamate formimidoyltransferase	3.63 ±2.64	8.6 ±0.38	0.41 ±0.15	0.28 ±0.05	0.61 ±0.05	9.15 ±0.98	0.25 ±0.14	0.53 ±0.06
Phvul.003G124400	Genomic DNA, chromosome 3, P1 clone: MKP6	8.81 ±5.68	18.75 ±1.07	1.87 ±0.45	2.9 ±0.8	1.23 ±0.41	18.06 ±1.14	3.41 ±0.52	3.48 ±1.18

Table S18 continued

		Transcript levels (FPKM)							
		Mock inoculation				*Xap* inoculation			
**Gene ID**	**Gene Annotation**	**0 h PI**	**8 h PI**	**24 h PI**	**48 h PI**	**0 h PI**	**8 h PI**	**24 h PI**	**48 h PI**
Phvul.003G131400	Germin-like protein subfamily 3	76.08 ±69.28	149.08 ±34.8	0.54 ±0.03	0.05 ±0.02	7.82 ±1.52	158.5 ±17.37	0.35 ±0.05	0.44 ±0.19
Phvul.003G142200	Δ(4)-3-oxosteroid 5-β-reductase	4.83 ±3.78	14.48 ±3.75	0.7 ±0.09	0.43 ±0.16	1.2 ±0.42	16.2 ±3.53	0.96 ±0.07	0.94 ±0.47
Phvul.003G148400	Allergen V5/TPX-1-related family protein-related	15.43 ±11.05	32.42 ±5.55	2.18 ±0.57	1.5 ±0.33	2.76 ±0.69	37.27 ±5.54	6.97 ±4.72	5.84 ±3.94
Phvul.003G198500	RNA-dependent RNA polymerase 2	1.21 ±0.83	3.15 ±0.27	0.64 ±0.05	0.8 ±0.13	0.28 ±0.03	3.2 ±0.13	0.63 ±0.16	0.46 ±0.05
Phvul.003G209600	β-lactamase / Penicillinase	12.49 ±10.61	33.23 ±1.48	1.78 ±0.21	2.01 ±0.38	2.18 ±0.3	36.52 ±1.72	2.21 ±0.53	1.85 ±0.16
Phvul.003G249200	No functional annotation	4.46 ±3.66	12.45 ±0.5	1.5 ±0.16	2.03 ±0.42	0.7 ±0.27	11.53 ±0.97	1.99 ±0.63	1.89 ±0.33
Phvul.003G295900	No functional annotation	115.33 ±112.76	383.84 ±30.19	1.77 ±0.31	1.62 ±0.08	1.72 ±0.35	453.7 ±20.73	1.78 ±0.5	1.5 ±0.15
Phvul.004G079500	NDH-dependent cyclic electron flow 5	71.39 ±70	218 ±0.83	0.81 ±0.14	0.73 ±0.02	1 ±0.08	232.96 ±2.38	0.88 ±0.13	0.84 ±0.29
Phvul.004G082300	Tyrosine-specific transport protein	12.24 ±9.34	32.77 ±2.32	1.47 ±0.28	1.32 ±0.23	2.39 ±0.22	34.58 ±0.8	1.3 ±0.22	1.34 ±0.15
Phvul.004G088300	Gigantea (GI)	24.15 ±23.04	71.62 ±1.96	0.6 ±0.04	0.8 ±0.29	1.06 ±0.1	76.31 ±0.81	0.5 ±0.05	0.65 ±0.06
Phvul.004G106700	Protein of unknown function	148.43 ±139.46	446.06 ±4.75	2.88 ±0.27	2.24 ±0.08	9.12 ±0.33	486.81 ±17.67	3.08 ±0.24	2.99 ±0.38
Phvul.004G114700	Leucine Rich Repeat (LRR_1) // Leucine rich repeat N-terminal domain (LRRNT_2)	1.77 ±1.5	4.69 ±0.51	0.13 ±0.02	0.99 ±0.64	0.12 ±0.05	5.47 ±0.38	0.23 ±0.09	0.38 ±0.2
Phvul.004G119400	No functional annotation	2.5 ±2.51	8.97 ±1.2	0 ±0	0 ±0	0 ±0	11.53 ±1.18	0 ±0	0 ±0

Table S18 continued

		Transcript levels (FPKM)							
		Mock inoculation				*Xap* inoculation			
**Gene ID**	**Gene Annotation**	**0 h PI**	**8 h PI**	**24 h PI**	**48 h PI**	**0 h PI**	**8 h PI**	**24 h PI**	**48 h PI**
Phvul.004G133000	Member of 'GDXG' family of lipolytic enzymes	5.72 ±5.39	19.11 ±1	0.39 ±0.06	0.31 ±0.12	0.51 ±0.25	18.09 ±1.38	0.17 ±0.07	0.59 ±0.13
Phvul.005G032700	Dirigent protein 20-related	2.73 ±1.73	5.94 ±0.34	0.91 ±0.08	0.59 ±0.05	0.6 ±0.15	5.58 ±0.52	1 ±0.12	0.65 ±0.09
Phvul.005G104600	Expansin-B1-related	20.15 ±15.15	31.57 ±6.85	2.43 ±0.46	0.66 ±0.21	4.39 ±0.59	24.97 ±4.26	1.77 ±0.2	1.65 ±0.4
Phvul.005G174000	ATP:ADP antiporter, AAA family	72.19 ±62.22	193.57 ±6.07	4.81 ±0.94	5.43 ±2.78	10.28 ±1.03	210.12 ±4.85	8.02 ±1.94	4.36 ±1.2
Phvul.006G063800	No functional annotation	109.07 ±107.01	311.04 ±14.3	0.63 ±0.16	0.33 ±0.13	1.21 ±0.22	355.65 ±6.16	0.47 ±0.01	0.46 ±0.03
Phvul.006G064100	α-Glucan phosphorylase 2, cytosolic	51.9 ±46.54	159.27 ±7.81	4.3 ±0.16	3.99 ±0.56	5.2 ±0.24	171.81 ±1.85	4.05 ±0.23	3.72 ±0.38
Phvul.006G065300	Phosphomethyl-pyrimidine synthase	335.18 ±321.36	990.33 ±18.39	5.27 ±0.53	4.79 ±0.69	9.7 ±0.35	1117.41 ±41.12	6.23 ±0.31	5.54 ±0.4
Phvul.006G067600	Ring finger protein 41, 151	8.21 ±6.93	23.81 ±2.98	2.07 ±0.4	2.04 ±0.67	1.65 ±0.12	26.18 ±1.59	2.55 ±1.05	1.93 ±0.2
Phvul.006G117800	Pre-mRNA 3'-end-processing factor FIP1 (FIP1L1, FIP1)	0.88 ±0.72	1.98 ±0.23	0.24 ±0.08	0.18 ±0.03	0.08 ±0.02	2.31 ±0.25	0.27 ±0.01	0.3 ±0.05
Phvul.006G146300	Chaperonin-like RBCX protein	60.05 ±57.54	188.11 ±1.23	0.19 ±0.05	0.21 ±0.03	0.89 ±0.13	220.61 ±17.82	0.03 ±0.03	0.12 ±0.08
Phvul.006G151300, *Phvul.006G151400*	β-glucosidase 45-related	3.22 ±2.72	9.85 ±0.6	0.9 ±0.17	0.94 ±0.17	0.49 ±0.1	10.06 ±1.37	1.32 ±0.39	1.18 ±0.38
Phvul.006G168800	Proprotein convertase subtilisin/kexin	2.94 ±2.68	8.19 ±0.17	0.37 ±0.02	0.35 ±0.01	0.22 ±0.09	8.08 ±0.13	0.26 ±0.04	0.37 ±0.07

Table S18 continued

		Transcript levels (FPKM)							
		Mock inoculation				*Xap* inoculation			
**Gene ID**	**Gene Annotation**	**0 h PI**	**8 h PI**	**24 h PI**	**48 h PI**	**0 h PI**	**8 h PI**	**24 h PI**	**48 h PI**
Phvul.006G183600	Dormancy/auxin associated protein (Auxin_repressed)	858.7 ±781.6	2585.37 ±142.81	142.07 ±27.12	174.1 ±54.63	72.73 ±11.27	2563.23 ±122.83	283.78 ±117.96	176.72 ±22.55
Phvul.006G207600	DnaJ homolog subfamily C member	6.94 ±6.69	19.4 ±1.46	0.1 ±0.01	0.24 ±0.24	0.22 ±0.12	20.76 ±1.44	0.17 ±0.09	0.36 ±0.13
Phvul.007G017500	Major facilitator protein	35.33 ±29.92	98.92 ±6.14	3.73 ±0.3	3.15 ±0.8	4.93 ±0.46	116.81 ±10.78	2.97 ±0.27	3.02 ±0.26
Phvul.007G021100	PPR repeat (PPR) // PPR repeat family (PPR_2)	24.51 ±24.12	72.7 ±3.14	0.07 ±0.03	0.04 ±0.02	0.1 ±0.05	78.12 ±3.04	0.01 ±0.01	0.04 ±0.02
Phvul.007G027200	B-box zinc finger (ZF-B_box) // CCT motif	15.47 ±15.33	44 ±1.93	0.06 ±0.03	0.06 ±0.03	0.14 ±0.09	60.23 ±4.64	0.13 ±0.05	0.03 ±0.01
Phvul.007G042100	PPR repeat (PPR) // PPR repeat family (PPR_2)	0.71 ±0.61	1.33 ±0.16	0.22 ±0.1	0.49 ±0.13	0.11 ±0.03	1.62 ±0.31	0.17 ±0.11	0.41 ±0.14
Phvul.007G074500	Protein REVEILLE 3-related	1.23 ±1.07	3.29 ±0.61	0.26 ±0.08	0.23 ±0.03	0.16 ±0.07	3.14 ±0.21	0.27 ±0.05	0.18 ±0.03
Phvul.007G090100	Isoliquiritigenin 2'-*O*-methyltransferase	2.91 ±2.46	11.69 ±1.48	0.28 ±0.02	0.1 ±0.02	0.42 ±0.16	11.41 ±1.57	0.39 ±0.13	0.27 ±0.09
Phvul.007G091400	No functional annotation	19.85 ±13.38	47.64 ±1.25	3.48 ±0.45	2.51 ±0.49	4.96 ±1.06	57.18 ±8.72	2.22 ±0.45	3.1 ±0.55
Phvul.007G135000	PPR repeat (PPR) // PPR repeat family (PPR_2)	1.68 ±1.41	4.79 ±0.26	0.61 ±0.11	0.74 ±0.15	0.29 ±0.01	5.15 ±0.55	0.56 ±0.17	0.76 ±0.24
Phvul.007G146900	PPR repeat (PPR) / (DYW_deaminase)	0.75 ±0.35	1.18 ±0.59	0.21 ±0.12	0.66 ±0.2	0.1 ±0.04	1.61 ±0.27	0.32 ±0.01	0.35 ±0.14
Phvul.007G150700	Nucleolin (NCL, NSR1)	509.71 ±447.24	923.43 ±461.93	35.49 ±4.77	35.08 ±7.33	92.09 ±3.71	1462.25 ±49.26	34.6 ±4.82	39.54 ±8.43

Table S18 continued

		Transcript levels (FPKM)							
		Mock inoculation				*Xap* inoculation			
**Gene ID**	**Gene Annotation**	**0 h PI**	**8 h PI**	**24 h PI**	**48 h PI**	**0 h PI**	**8 h PI**	**24 h PI**	**48 h PI**
Phvul.007G214800	Drought responsive ATP-binding motif containing protein	81.55 ±78.7	162.28 ±81.17	0.75 ±0.25	0.96 ±0.15	1.48 ±0.35	299.22 ±21.15	0.75 ±0.14	0.71 ±0.12
Phvul.008G003500	Protein NRT1/ PTR family 6.1	46.9 ±37.25	86.77 ±43.54	2.18 ±0.16	1.54 ±0.24	9.72 ±0.21	133.09 ±1.26	2.75 ±0.29	2.19 ±0.32
Phvul.008G048400	PPR repeat (PPR) // PPR repeat family (PPR_2)	4.67 ±4.37	8.72 ±4.36	0.32 ±0.05	0.67 ±0.2	0.22 ±0.05	13.58 ±0.68	0.39 ±0.18	0.5 ±0.16
Phvul.008G148100	F-box associated (FBA_1) // F-box-like	11.04 ±9.08	20.6 ±10.3	2.05 ±0.15	2.57 ±0.23	1.92 ±0.43	32.6 ±0.35	2.45 ±0.57	3.13 ±0.7
Phvul.008G222000	Outer membrane protein insertion porin family (SAM50, TOB55, BamA)	3.94 ±3.55	8.36 ±4.19	0.44 ±0.06	0.41 ±0.05	0.44 ±0.01	13.35 ±0.71	0.35 ±0.08	0.36 ±0.08
Phvul.008G225600	No functional annotation	10.45 ±9.95	19.35 ±9.68	0.3 ±0.05	0.33 ±0.03	0.34 ±0.13	37.37 ±2.59	0.39 ±0.09	0.39 ±0.1
Phvul.008G264700	Heat shock protein 42	328.92 ±321.83	699.76 ±350.95	1.31 ±0.21	1.87 ±0.1	3.26 ±0.38	1194.12 ±95.48	1.82 ±0.51	1.79 ±0.25
Phvul.009G013600	Cathepsin L	323.57 ±288.69	654.49 ±333.05	23.02 ±2.99	32.49 ±3.68	30.87 ±5.55	1051.09 ±36.36	33.45 ±8.51	29.51 ±6.9
Phvul.009G023700	RNA recognition motif	299.87 ±237.27	556.6 ±278.53	70.22 ±2.26	71.01 ±2.57	61.72 ±3.52	835.11 ±9.23	74.65 ±1.89	72.97 ±3.57
Phvul.009G045000	Pseudo-response regulator 5 (PRR5)	44.99 ±41.6	81.96 ±41.04	3.1 ±0.41	2.53 ±0.37	3.51 ±0.41	131 ±3.9	3.2 ±0.09	2.61 ±0.46
Phvul.009G090600	No functional annotation	8.14 ±7.85	16.29 ±8.34	0.22 ±0.12	0.26 ±0.09	0.55 ±0.09	28.75 ±1.47	0.16 ±0.05	0.49 ±0.17

Table S18 continued

		Transcript levels (FPKM)							
		Mock inoculation				*Xap* inoculation			
**Gene ID**	**Gene Annotation**	**0 h PI**	**8 h PI**	**24 h PI**	**48 h PI**	**0 h PI**	**8 h PI**	**24 h PI**	**48 h PI**
Phvul.009G152200	Filament-like plant protein 3	16.13 ±13.48	28.43 ±14.22	3.51 ±0.19	3.18 ±0.96	3.34 ±0.41	45.77 ±1.33	5.23 ±0.88	3.71 ±0.39
Phvul.009G200900	Protein CHUP1, chloroplastic	11.88 ±11.37	23.27 ±11.64	0.35 ±0.03	0.22 ±0.04	0.76 ±0.14	37.34 ±3.19	0.59 ±0.06	0.55 ±0.05
Phvul.009G227100	Cathepsin H (CTSH)	1190.97 ±1009.11	2332 ±1176.28	270.03 ±35.48	502.68 ±85.24	213.99 ±49.55	3821.31 ±225.14	450.49 ±139.55	437.93 ±166.38
Phvul.009G227800	Germin-like protein subfamily 3	301.5 ±287.71	565.99 ±283.69	0.87 ±0.3	0.51 ±0.09	16.86 ±3.38	876.11 ±87	1.23 ±0.68	0.99 ±0.45
Phvul.009G257000	Serine/threonine kinase	0.82 ±0.47	1.07 ±0.54	0.19 ±0.09	0.31 ±0.12	0.13 ±0.03	1.51 ±0.19	0.23 ±0.08	0.22 ±0.1
Phvul.010G023700	F21O3.2 protein-related	126.49 ±115.58	248.01 ±124.1	10.49 ±1.21	10.4 ±3.37	12.9 ±2.59	416.16 ±10.84	14.67 ±2.62	11.99 ±0.75
Phvul.010G097300	Bestrophin, RFP-TM, chloride channel	7.87 ±6.69	15.25 ±7.75	2.23 ±0.39	2.62 ±0.24	1.41 ±0.07	22.22 ±1.2	1.84 ±0.25	2.2 ±0.42
Phvul.010G142600	IAA-amino acid hydrolase ILR1-like 1-related	13.29 ±9.9	23.02 ±11.55	2.84 ±0.21	3.06 ±0.64	2.71 ±0.42	34.53 ±0.54	3.53 ±0.23	3.01 ±0.22
Phvul.010G156300	Post-illumination chlorophyll fluorescence increase protein	386.94 ±348.79	777.68 ±389.57	25.44 ±1.6	23.7 ±5.09	33.47 ±4.72	1328.07 ±70.19	24.09 ±3.13	26.1 ±2.39
Phvul.010G164200	Bactericidal permeability-increasing BPI protein-related	9.01 ±7.97	17.27 ±8.69	0.77 ±0.16	1.09 ±0.3	1.29 ±0.21	25.48 ±0.66	1 ±0.08	1.22 ±0.05
Phvul.011G045100	Transcription termination factor family protein	7.77 ±6.36	14.17 ±7.09	1.4 ±0.13	1.63 ±0.16	1.72 ±0.1	22.9 ±0.38	1.73 ±0.23	1.63 ±0.23

Table S18 continued

		Transcript levels (FPKM)							
		Mock inoculation				*Xap* inoculation			
**Gene ID**	**Gene Annotation**	**0 h PI**	**8 h PI**	**24 h PI**	**48 h PI**	**0 h PI**	**8 h PI**	**24 h PI**	**48 h PI**
Phvul.011G050000	Expressed protein	109.56 ± 105.68	229.68 ±114.9	1.6 ±0.44	2 ±0.43	1.69 ±0.21	414.39 ±12.14	1.74 ±0.62	1.87 ±0.36
Phvul.011G056500	Protein C13C4.7	719.9 ±634.74	1522.89 ±785.77	26.43 ±8.04	32.64 ±6.7	97.19 ±13.01	2329.47 ±175.92	45.69 ±2.36	52.99 ±6.9
Phvul.011G056900	Lysine-specific demethylase 8 (KDM8, JMJD5)	8.53 ±8.27	19.03 ±9.64	0.16 ±0.03	0.16 ±0	0.2 ±0.08	29.43 ±2.03	0.34 ±0.08	0.19 ±0.03
Phvul.011G062100	MYB-like DNA-binding domain	7.43 ±7.07	12.79 ±6.4	0.3 ±0.1	0.24 ±0.02	0.37 ±0.03	21.99 ±2.2	0.2 ±0.06	0.39 ±0.06
Phvul.011G090500	Nucleobase-ascorbate transporter 12	4.25 ±3.83	7.84 ±3.96	0.23 ±0.01	0.37 ±0.1	0.56 ±0.1	12.39 ±0.68	0.35 ±0.1	0.37 ±0.02
Phvul.011G138600	Anthocyanidin 3-*O*-glucoside 2"-*O*-glucosyltransferase	9.09 ±8.57	22.87 ±12.41	0.09 ±0.05	0.1 ±0	0.55 ±0.08	35.3 ±2.56	0.23 ±0.06	0.14 ±0
Phvul.011G163100	Methyltransferase-like protein 20	3.19 ±2.89	4.79 ±2.41	0.49 ±0.06	0.4 ±0.08	0.37 ±0.11	6.83 ±2.1	0.34 ±0.18	0.72 ±0.25
XLOC_006186	No functional annotation	2.82 ±2.62	7.26 ±1.94	0 ±0	0 ±0	0 ±0	10.03 ±1.86	0 ±0	0 ±0

Table S19. Transcript abundance for genes upregulated in the CBB-resistant RIL and the CBB-susceptible RIL at 0 h post-inoculation (PI) with *Xanthomonas axonopodis* (*Xap*). Transcript levels for individual genes corresponding to each RIL/ inoculation treatment/ sampling time PI are represented as FPKM ± SE of three experimental replicates.

		**Transcript levels (FPKM)**							
		**Mock inoculation**				***Xap* inoculation**			
**Gene ID**	**Gene Annotation**	**0 h PI**	**8 h PI**	**24 h PI**	**48 h PI**	**0 h PI**	**8 h PI**	**24 h PI**	**48 h PI**
**CBB-resistant RIL**									
Phvul.001G157500	Prenylated RAB acceptor 1-related	4.13 ± 1.36	4.92 ±1.11	0.8 ±0.12	0.42 ±0.05	17.7 ±4.15	6.11 ±3.13	0.59 ±0.03	0.4 ±0.05
Phvul.002G077400	Glycosyltransferase 8 domain-containing protein	18.37 ± 3.15	16.06 ±2.06	11.62 ±1.2	9.79 ±0.6	79.39 ±4.49	35.96 ±21.52	13.35 ±2.01	12.71 ±4.3
Phvul.002G142600	No functional annotation	0.5 ± 0.16	0.53 ±0.06	0.63 ±0.1	0.71 ±0.14	2.62 ±0.98	0.79 ±0.47	0.68 ±0.17	0.68 ±0.16
Phvul.003G052400	Xyloglucan endotransglucosylase/ Hydrolase protein 33-related	1.8 ± 0.48	1.83 ±0.42	0.9 ±0.64	0.8 ±0.13	9.76 ±1.8	3.5 ±1.46	1.28 ±0.62	0.58 ±0.24
Phvul.003G053700	No functional annotation	15.23 ± 7.28	10.02 ±6.14	4.7 ±2.22	3.65 ±2.41	77.08 ±15.07	17.73 ±14.67	4.21 ±1.63	2.13 ±1.2
Phvul.003G137600	Xyloglucan:xyloglucosyl transferase	5.41 ± 2.91	5.45 ±2.22	8.27 ±5.31	6.26 ±1.05	32.13 ±6.5	9.41 ±5.78	7.95 ±2.12	8.15 ±0.85
Phvul.003G147600	Xyloglucan endotransglucosylase / Hydrolase protein 25-related	0.8 ± 0.06	1.13 ±0.42	0.52 ±0.25	0.47 ±0.15	11.91 ±4.18	4.17 ±2.98	0.96 ±0.44	1.03 ±0.83
Phvul.003G147700	Xyloglucan endotransglucosylase/ hydrolase protein 25-related	7.13 ± 3.17	15.38 ±5.48	6.94 ±6.66	0.67 ±0.21	68.16 ±9.84	25.49 ±10.15	2.37 ±1.37	1.07 ±0.4
Phvul.003G205700	Plant protein of unknown function	1.14 ± 0.31	1.11 ±0.36	1.11 ±0.21	1.06 ±0.18	5.45 ±0.78	1.95 ±1.11	1.14 ±0.08	0.59 ±0.21

Table S19 continued

		Transcript levels (FPKM)							
		Mock inoculation				*Xap* inoculation			
**Gene ID**	**Gene Annotation**	**0 h PI**	**8 h PI**	**24 h PI**	**48 h PI**	**0 h PI**	**8 h PI**	**24 h PI**	**48 h PI**
Phvul.003G238200	Lysine-rich arabinogalactan protein 17-related	119.22 ±42.68	94.18 ±35.61	38.48 ±10.12	28.2 ±4.76	477.75 ±77.64	165.36 ±97.56	47.37 ±8.63	14.72 ±2.98
Phvul.005G084800	No functional annotation	8.36 ± 1.96	6.65 ±0.95	5.89 ±0.54	8.47 ±0.97	36.09 ±2.68	15.69 ±10.9	8.54 ±0.48	8.79 ±2.52
Phvul.005G099500	Fasciclin-like arabinogalactan protein 13-related	31.25 ±15.62	25.2 ±8.07	9.81 ±6.34	3.75 ±0.75	152.7 ±18.65	53.35 ±33.94	6.03 ±2.16	2.25 ±0.54
Phvul.007G110600	Phosphate-induced protein 1 conserved region	24.39 ± 6.67	24.71 ±3.24	10.77 ±4.52	8.86 ±1.25	129.74 ±24.32	48.91 ±26.67	10.04 ±1.69	5.77 ±1.59
Phvul.007G186500	Protein NDRG1	12.49 ± 6.91	11.65 ±4.1	5.58 ±3.73	2.76 ±0.59	56.73 ±8.97	15.46 ±9.08	3.38 ±1.03	1.56 ±0.52
Phvul.007G215200	T14P4.7 Protein	5.58 ± 0.96	6.66 ±0.66	2.57 ±0.36	2.26 ±0.12	36.17 ±9.53	11.07 ±5.4	2.57 ±0.46	2.66 ±0.57
Phvul.008G175700	Phosphate-induced protein 1 protein, putative-related	1.11 ± 0.42	0.95 ±0.28	0.58 ±0.21	0.92 ±0.2	7.23 ±1.61	3.67 ±2.93	1.17 ±0.42	0.95 ±0.7
Phvul.009G032100	Phosphate-induced protein 1 protein, putative-related	6.63 ± 1.36	10.4 ±2.92	5.56 ±2.36	6.44 ±1.02	39.58 ±10.76	15.06 ±5.11	6.64 ±2.47	6.48 ±0.31
Phvul.009G173000	Plant protein of unknown function	1.52 ± 0.37	1.98 ±0.35	1 ±0.26	0.65 ±0.16	6.96 ±1.42	2.9 ±1.02	0.89 ±0.12	0.55 ±0.23
Phvul.009G180401	No functional annotation	24.48 ± 5.81	28.35 ±3.88	10.49 ±5.7	13.65 ±1.87	118.55 ±13.45	55.15 ±27.47	13.86 ±2.94	11.76 ±0.96
Phvul.011G024801	Expansin-like A1-related	12.74 ± 2.3	14.6 ±1.08	7.36 ±2.22	8.12 ±0.57	90.05 ±9.32	37.46 ±22.68	16.41 ±5.03	15.99 ±6.32
Phvul.011G055600	No functional annotation	3.7 ± 1.33	2.11 ±0.57	4.04 ±0.5	4.4 ±0.35	16.08 ±3.42	4.51 ±2.81	6.76 ±1.88	3.9 ±1.13
Phvul.011G214200	AXI 1 protein-like protein	1.22 ± 0.45	1.13 ±0.21	1.16 ±0.55	1.25 ±0.14	8.41 ±1.14	2.92 ±2	2.86 ±1.25	4.6 ±2.78

Table S19 continued

		Transcript levels (FPKM)							
		Mock inoculation				*Xap* inoculation			
**Gene ID**	**Gene Annotation**	**0 h PI**	**8 h PI**	**24 h PI**	**48 h PI**	**0 h PI**	**8 h PI**	**24 h PI**	**48 h PI**
**CBB-susceptible RIL**									
Phvul.001G157500	Prenylated RAB acceptor 1-related	1.59 ±0.38	3.17 ±0.29	0.59 ±0.05	0.48 ±0.15	7.08 ±3.18	2.64 ±0.32	0.49 ±0.25	0.98 ±0.46
Phvul.002G077400	Glycosyltransferase 8 domain-containing protein	14.98 ±1.7	13.44 ±0.69	11.34 ±0.62	10.22 ±1.4	78.62 ±9.1	14.98 ±1.61	10.08 ±1.33	13.56 ±3.01
Phvul.002G142600	No functional annotation	0.67 ±0.15	0.58 ±0.06	0.54 ±0.19	0.58 ±0.16	4.38 ±1.88	0.46 ±0.05	0.86 ±0.1	0.71 ±0.29
Phvul.003G052400	Xyloglucan endotransglucosylase/ Hydrolase protein 33-related	1.28 ±0.18	1 ±0.02	1.05 ±0.42	0.52 ±0.2	14.05 ±6.65	1.33 ±0.22	0.99 ±0.28	1.58 ±1
Phvul.003G053700	No functional annotation	6.85 ±2.68	3.72 ±0.4	3.56 ±1.15	3.08 ±1.82	55.77 ±16.33	1.59 ±0.59	2.07 ±0.69	6.55 ±2.48
Phvul.003G137600	Xyloglucan:xyloglucosyl transferase / Xyloglucan endotransglycosylase	4.49 ±1.44	3.04 ±0.66	5.42 ±1.81	4.69 ±1.11	29.24 ±12.62	2.8 ±0.12	4.32 ±0.64	6.07 ±2.86
Phvul.003G147600	Xyloglucan endotransglucosylase/hydrolase protein 25-related	0.48 ±0.36	0.94 ±0.08	0.27 ±0.1	0.39 ±0.23	9.75 ±3.41	0.83 ±0.2	0.13 ±0.03	1.23 ±1.03
Phvul.003G147700	Xyloglucan endotransglucosylase/hydrolase protein 25-related	4.87 ±3.89	14.79 ±2.47	1.02 ±0.63	0.78 ±0.35	60.41 ±32.59	19.46 ±4.54	0.33 ±0.08	1.55 ±1.22
Phvul.003G205700	Plant protein of unknown function	1.21 ±0.35	0.67 ±0.09	1.66 ±0.22	1.11 ±0.09	5.43 ±0.58	0.8 ±0.1	1.71 ±0.1	1.03 ±0.33
Phvul.003G238200	Lysine-rich arabinogalactan protein 17-related	81.15 ±12.66	52.32 ±5.65	50.42 ±15.18	16.49 ±2.31	448.18 ±162.03	45.52 ±4.8	41.58 ±4.47	48.26 ±15.5
Phvul.005G084800	No functional annotation	6.87 ±0.57	5.76 ±0.65	5.88 ±0.77	7.44 ±0.62	29.93 ±4.77	5.81 ±0.14	5.9 ±0.33	8.61 ±2.42

Table S19 continued

		Transcript levels (FPKM)							
		Mock inoculation				*Xap* inoculation			
**Gene ID**	**Gene Annotation**	**0 h PI**	**8 h PI**	**24 h PI**	**48 h PI**	**0 h PI**	**8 h PI**	**24 h PI**	**48 h PI**
Phvul.005G099500	Fasciclin-like arabinogalactan protein 13-related	11.68 ±2.78	10.84 ±1.84	5.88 ±3.34	1.67 ±0.45	121.41 ±56.13	10.32 ±1.85	3.91 ±0.99	4.95 ±1.87
Phvul.007G110600	Phosphate-induced protein 1 conserved region	16.55 ±2.49	20.82 ±0.91	7.69 ±1.12	6.4 ±1.15	129.75 ±29.88	20.2 ±2.74	8.63 ±1.64	8.68 ±3.13
Phvul.007G186500	Protein NDRG1	5.69 ±0.99	3.63 ±1.85	3.4 ±1.56	1.43 ±0.31	55.95 ±30.64	5.54 ±0.43	2.35 ±0.13	2.55 ±0.78
Phvul.007G215200	T14P4.7 Protein	4.36 ±0.46	3.21 ±1.63	2.67 ±0.62	2.19 ±0.46	46.18 ±25.34	5.11 ±0.44	2.28 ±0.48	2.49 ±0.28
Phvul.008G175700	Phosphate-induced protein 1 protein, putative-related	0.54 ±0.23	0.4 ±0.23	0.38 ±0.17	0.81 ±0.34	5.16 ±1.26	0.64 ±0.18	0.31 ±0.1	1.41 ±0.83
Phvul.009G032100	Phosphate-induced protein 1 protein, putative-related	6.13 ±2.57	8.84 ±4.42	3.79 ±1.3	2.2 ±0.43	44.35 ±14.54	14.04 ±1.14	3.69 ±0.43	4.62 ±1.17
Phvul.009G173000	Plant protein of unknown function	1.21 ±0.35	0.94 ±0.48	0.73 ±0.17	0.63 ±0.18	7.26 ±1.45	1.82 ±0.19	0.8 ±0.09	0.66 ±0.25
Phvul.009G180401	No functional annotation	14.72 ±5.68	17.23 ±8.63	6.6 ±1.76	7.88 ±1.11	101.24 ±16.05	32.66 ±3.07	9.12 ±1.47	9.86 ±1.52
Phvul.011G024801	Expansin-like A1-related	8.57 ±1.7	8.74 ±4.63	7.46 ±1.13	9.51 ±3.99	78.03 ±18.75	13.97 ±0.5	7.28 ±0.73	15.43 ±8.07
Phvul.011G055600	No functional annotation	2.46 ±0.62	1.08 ±0.55	2.3 ±0.17	2.11 ±0.4	14.09 ±5.1	1.54 ±0.21	2.27 ±0.13	5.84 ±3.59
Phvul.011G214200	AXI 1 protein-like protein	0.91 ±0.12	0.48 ±0.25	0.81 ±0.13	1.99 ±1.13	7.93 ±3.03	0.76 ±0.07	0.86 ±0.18	2.36 ±1.41

Table S20. Transcript abundance for a gene upregulated in the CBB-resistant RIL at 0 h post-inoculation (PI) with *Xanthomonas axonopodis* (*Xap*) and 8 h thereafter. Transcript levels for individual genes corresponding to each RIL/ inoculation treatment/ sampling time PI are represented as FPKM ± SE of three experimental replicates.

		Transcript levels (FPKM)							
		Mock inoculation				*Xap* inoculation			
**Gene ID**	**Gene Annotation**	**0 h PI**	**8 h PI**	**24 h PI**	**48 h PI**	**0 h PI**	**8 h PI**	**24 h PI**	**48 h PI**
Phvul.003G147400	Xyloglucan endotransglucosylase/ Hydrolase protein 25-related	1.06 ±0.5	0.12 ±0.07	0.12 ±0.07	0.33 ±0.09	4.94 ±0.83	2.38 ±1.98	0.48 ±0.27	0.91 ±0.55

Table S21. Transcript abundance for a gene downregulated in the CBB-resistant RIL at 0 h post-inoculation (PI) with *Xanthomonas axonopodis* (*Xap*) and upregulated 24 h thereafter.
Transcript levels for individual genes corresponding to each RIL/ inoculation treatment/ sampling time PI are represented as FPKM ± SE of three experimental replicates.

		Transcript levels (FPKM)							
		Mock inoculation				*Xap* inoculation			
**Gene ID**	**Gene Annotation**	**0 h PI**	**8 h PI**	**24 h PI**	**48 h PI**	**0 h PI**	**8 h PI**	**24 h PI**	**48 h PI**
Phvul.001G158300	No functional annotation	6.37 ± 1.84	0 ± 0	0 ± 0	6.18 ± 2.15	0 ± 0	0 ± 0	7.3 ± 4.25	19.4 ± 9.54

Table S22. Transcript abundance for genes downregulated in the CBB-resistant RIL at 0 h post-inoculation (PI) with *Xanthomonas axonopodis* (*Xap*) and upregulated 48 h PI. Transcript levels for individual genes corresponding to each RIL/ inoculation treatment/ sampling time PI are represented as FPKM ± SE of three experimental replicates.

		**Transcript levels (FPKM)**							
		**Mock inoculation**				***Xap* inoculation**			
**Gene ID**	**Gene Annotation**	**0 h PI**	**8 h PI**	**24 h PI**	**48 h PI**	**0 h PI**	**8 h PI**	**24 h PI**	**48 h PI**
Phvul.001G040300	L-type lectin-domain containing receptor kinase ix.1-related	2.8 ±2.21	2.97 ±1.09	0.65 ±0.05	0.9 ±0.23	0.55 ±0.24	2.9 ±1	1.32 ±0.31	6.13 ±1.44
Phvul.001G075400	Protein phosphatase 2	13.09 ±5.61	10.89 ±5.63	3.93 ±1.8	4.9 ±4.31	3.02 ±0.79	10.75 ±3.13	3.23 ±0.77	54.08 ±36.1
Phvul.001G145900	Dirigent protein 1-related	1.32 ±0.9	1.71 ±0.85	1.54 ±0.62	2.09 ±0.57	0 ±0	1.69 ±0.71	2.98 ±1.12	34.15 ±2.4
Phvul.003G009200	Cytochrome P450	0.66 ±0.39	0.91 ±0.33	0.19 ±0.05	0.24 ±0.06	0.11 ±0.02	0.89 ±0.28	0.34 ±0.05	3.91 ±0.63
Phvul.004G138500	Trypsin and protease inhibitor	7.43 ±5.58	10.07 ±3.69	2.1 ±0.55	0.87 ±0.38	1.56 ±0.36	8.03 ±3.35	3.85 ±1.67	8.75 ±4.77
Phvul.010G063900	Dirigent protein 19	10.25 ±5.71	9.19 ±1.45	9.47 ±3.86	11.81 ±2.6	1.74 ±0.35	10.74 ±3.74	19.77 ±3.45	198.38 ±17.65
Phvul.011G203450	VQ motif	1.13 ±0.31	0.43 ±0.12	0.77 ±0.25	1.2 ±0.71	0 ±0	1.38 ±0.38	3.33 ±2.22	31.48 ±4.47

Table S23. Transcript abundance for a gene downregulated in the CBB-resistant RIL at 0 h post-inoculation (PI) with *Xanthomonas axonopodis* (*Xap*) and upregulated in the CBB-susceptible RIL 24 h PI. Transcript levels for individual genes corresponding to each RIL/ inoculation treatment/ sampling time PI are represented as FPKM ± SE of three experimental replicates.

		**Transcript levels (FPKM)**							
		**Mock inoculation**				***Xap* inoculation**			
**Gene ID**	**Gene Annotation**	**0 h PI**	**8 h PI**	**24 h PI**	**48 h PI**	**0 h PI**	**8 h PI**	**24 h PI**	**48 h PI**
**CBB-resistant RIL**									
Phvul.003G295200	Carboxylesterase 12-related	78.58 ±66.05	112.75 ±44.92	13.62 ±0.54	26.22 ±5.24	13.97 ±1.39	105.44 ±45.14	21.94 ±3.37	70.05 ±16.1
**CBB-susceptible RIL**									
Phvul.003G295200	Carboxylesterase 12-related	28.9 ±11.37	81.91 ±18.31	5.2 ±1.15	53.37 ±7.86	8.12 ±2.33	69.48 ±6.99	28.54 ±18.45	16.14 ±6.84

Table S24. Transcript abundance for a gene downregulated in the CBB-resistant RIL at 0 h post-inoculation (PI) with *Xanthomonas axonopodis* (*Xap*) and upregulated in the CBB-susceptible RIL 48 h PI. Transcript levels for individual genes corresponding to each RIL/ inoculation treatment/ sampling time PI are represented as FPKM ± SE of three experimental replicates.

		**Transcript levels (FPKM)**							
		**Mock inoculation**				***Xap* inoculation**			
**Gene ID**	**Gene Annotation**	**0 h PI**	**8 h PI**	**24 h PI**	**48 h PI**	**0 h PI**	**8 h PI**	**24 h PI**	**48 h PI**
**CBB-resistant RIL**									
Phvul.010G144300	Acid phosphatase-related	139.23 ±138.44	131.59 ±106.68	381.59 ±225.37	79.13 ±69.37	0.6 ±0.32	123.69 ±92.98	249.52 ±63.65	20.73 ±5.36
**CBB-susceptible RIL**									
Phvul.010G144300	Acid phosphatase-related	6.62 ±3.61	20.28 ±13.99	25.84 ±17.65	3.17 ±2.33	2.82 ±2.34	39.78 ±3.48	16.64 ±7.63	20.68 ±2.73

Table S25. Transcript abundance for a gene downregulated in the CBB-resistant RIL at 0 h post-inoculation (PI) with *Xanthomonas axonopodis* (*Xap*) and upregulated 24 and 48 h PI. Transcript levels for individual genes corresponding to each RIL/ inoculation treatment/ sampling time PI are represented as FPKM ± SE of three experimental replicates.

		Transcript levels (FPKM)							
		Mock inoculation				*Xap* inoculation			
**Gene ID**	**Gene Annotation**	**0 h PI**	**8 h PI**	**24 h PI**	**48 h PI**	**0 h PI**	**8 h PI**	**24 h PI**	**48 h PI**
Phvul.008G287200, Phvul.008G287300	6'-Deoxychalcone synthase	1.24 ± 1.15	1.65 ± 0.35	0.5 ± 0.18	1 ± 0.4	0.2 ± 0.07	1.95 ± 0.94	2.49 ± 1.14	27.17 ± 2.45

Table S26. Transcript abundance for genes upregulated in both RILs at 0 h post-inoculation (PI) with *Xanthomonas axonopodis* (*Xap*), and 8 h PI in the CBB-resistant RIL. Transcript levels for individual genes corresponding to each RIL/ inoculation treatment/ sampling time PI are represented as FPKM ± SE of three experimental replicates.

		**Transcript levels (FPKM)**							
		**Mock inoculation**				***Xap* inoculation**			
**Gene ID**	**Gene Annotation**	**0 h PI**	**8 h PI**	**24 h PI**	**48 h PI**	**0 h PI**	**8 h PI**	**24 h PI**	**48 h PI**
**CBB-resistant RIL**									
Phvul.002G122200	Abscisic acid 8'-hydroxylase 1-related	5.74 ±3.05	2.31 ±1.51	3.34 ±0.74	4.18 ±1.4	53.7 ±10.28	16.42 ±15.09	8.61 ±3.63	8.53 ±4.9
Phvul.002G231500, *Phvul.002G231700*	17.6 kDa class I heat shock protein 1-related	7.13 ±1.33	5.7 ±0.7	8.83 ±1.2	8.82 ±1.52	71.49 ±40.11	27.17 ±20.85	9.87 ±0.62	14.06 ±4.53
Phvul.002G049700	F2J10.8 protein-related	13.14 ±3.58	9.96 ±2.55	5.84 ±1.51	5.35 ±0.57	189.17 ±29.9	70.09 ±63.58	7.07 ±1.81	6.1 ±2.72
Phvul.003G147500	Xyloglucan endotransglucosylase/ Hydrolase protein 25-related	1.23 ±0.54	0.44 ±0.31	0.13 ±0.06	0.31 ±0.24	18.75 ±4.31	6.79 ±6.55	1.22 ±0.44	1.35 ±0.87
Phvul.004G044100	ATP-dependent CLP protease ATP-binding subunit CLPB	1.07 ±0.28	0.64 ±0.06	1.97 ±0.14	2.55 ±0.35	11.99 ±6.6	4.15 ±3.54	2.51 ±0.28	2.29 ±0.49
Phvul.005G111100	No functional annotation	9.27 ±4.19	5.48 ±3.76	4.37 ±0.83	2.77 ±0.11	87.19 ±6.85	25.68 ±24.16	4.88 ±0.5	2.16 ±0.17
Phvul.008G016500, *Phvul.008G016600*	IQ calmodulin-binding motif / BAG domain	3.67 ±1.84	1.41 ±0.5	4.02 ±0.34	4.6 ±0.98	60.8 ±31.89	21.84 ±20.7	5.08 ±0.76	5.43 ±0.96
Phvul.008G175800, *Phvul.008G175900*, *Phvul.008G176000*	Cysteine-rich receptor-like protein kinase 28-related	28.97 ±11.9	12.23 ±4.77	8.62 ±2.74	9.86 ±4.05	260.69 ±43.9	109.18 ±101.5	23.08 ±10.42	28.77 ±21.06
Phvul.009G152500	17.6 kDa class I heat shock protein 1-related	1.96 ±0.76	0.59 ±0.21	2.02 ±0.4	1.25 ±0.15	17.66 ±8.45	5.56 ±5.25	2.84 ±0.99	2.95 ±0.62
Phvul.010G125400	Auxin responsive protein	0.6 ±0.22	0.5 ±0.16	0.22 ±0.09	0.29 ±0.06	11.99 ±3.85	3.84 ±3.39	0.23 ±0.06	0.24 ±0.12

Table S26 continued

		Transcript levels (FPKM)							
		Mock inoculation				*Xap* inoculation			
**Gene ID**	**Gene Annotation**	**0 h PI**	**8 h PI**	**24 h PI**	**48 h PI**	**0 h PI**	**8 h PI**	**24 h PI**	**48 h PI**
**CBB-susceptible RIL**									
Phvul.002G122200	Abscisic acid 8'-hydroxylase 1-related	3.08 ± 1.15	0.74 ± 0.06	3.45 ± 0.52	7.89 ± 4.41	58.93 ± 13.59	1.1 ± 0.12	3.34 ± 0.28	11.08 ± 7
Phvul.002G231500, *Phvul.002G231700*	17.6 kDa class I heat shock protein 1-related	6.16 ± 0.33	6.15 ± 0.62	5.8 ± 0.91	5.9 ± 0.12	36.48 ± 27.78	7.38 ± 0.5	4.99 ± 0.64	5.62 ± 0.38
Phvul.002G049700	F2J10.8 protein-related	8.19 ± 0.41	6.53 ± 0.35	5.29 ± 0.72	4.64 ± 1.28	186.16 ± 24.66	6.39 ± 0.45	4.56 ± 0.95	9.73 ± 5.59
Phvul.003G147500	Xyloglucan endotransglucosylase/ Hydrolase protein 25-related	0.15 ± 0.15	0.06 ± 0.03	0.11 ± 0.04	0.52 ± 0.39	12.67 ± 3.09	0.08 ± 0.04	0.08 ± 0.08	1.5 ± 1.11
Phvul.004G044100	ATP-dependent CLP protease ATP-binding subunit CLPB	0.9 ± 0.17	0.62 ± 0.05	1.58 ± 0.18	1.5 ± 0.08	6.42 ± 4.12	0.75 ± 0.05	1.49 ± 0.16	1.51 ± 0.08
Phvul.005G111100	No functional annotation	5.53 ± 1.68	3.15 ± 0.23	4.08 ± 0.7	3.51 ± 0.49	70.33 ± 7.1	2.67 ± 0.47	4.23 ± 0.54	5.48 ± 1.11
Phvul.008G016500, *Phvul.008G016600*	IQ calmodulin-binding motif /BAG domain	3.55 ± 0.91	0.97 ± 0.49	3.33 ± 0.08	3.16 ± 0.33	34.26 ± 20.21	1.3 ± 0.14	3.19 ± 0.66	3.65 ± 0.5
Phvul.008G175800, *Phvul.008G175900*, *Phvul.008G176000*	Cysteine-rich receptor-like protein kinase 28-related	18.23 ± 5.77	4.08 ± 2.04	4.97 ± 1.21	13.69 ± 8.38	253.89 ± 20.92	7.23 ± 1.04	5.54 ± 0.4	30.58 ± 21.82
Phvul.009G152500	17.6 kDa class I heat shock protein 1-related	1.16 ± 0.6	0.2 ± 0.12	2.08 ± 0.37	1.98 ± 0.23	7.82 ± 4.9	0.71 ± 0.23	2.07 ± 0.02	2.92 ± 0.66
Phvul.010G125400	Auxin responsive protein	0.57 ± 0.33	0.46 ± 0.24	0.28 ± 0.12	0.27 ± 0.09	10.87 ± 0.99	0.42 ± 0.14	0.27 ± 0.08	0.31 ± 0.12

Table S27. Transcript abundance for genes downregulated at 0 h post-inoculation (PI) with *Xanthomonas axonopodis* (*Xap*) in both RILs and upregulated 48 h PI in the CBB-resistant RIL. Transcript levels for individual genes corresponding to each RIL/ inoculation treatment/ sampling time PI are represented as FPKM ± SE of three experimental replicates.

		**Transcript levels (FPKM)**							
		**Mock inoculation**				***Xap* inoculation**			
**Gene ID**	**Gene Annotation**	**0 h PI**	**8 h PI**	**24 h PI**	**48 h PI**	**0 h PI**	**8 h PI**	**24 h PI**	**48 h PI**
**CBB-resistant RIL**									
Phvul.001G145800	Dirigent protein 1-related	1.91 ±1.37	3.16 ±1.28	2.45 ±0.53	3.06 ±0.64	0.23 ±0.14	3.08 ±1.3	3.81 ±0.72	28.96 ±2.86
Phvul.002G032866	Isoflavone reductase	1.2 ±0.72	1.12 ±0.47	1.39 ±0.7	1.55 ±0.5	0.29 ±0.1	1.79 ±0.85	2.9 ±0.23	30.4 ±2.74
Phvul.003G051700	Isoflavone synthase	2.28 ±1.44	2.45 ±0.87	3.12 ±1.45	3.09 ±1.13	0.33 ±0.12	3.07 ±1.42	5.66 ±2.34	102.17 ±4.19
Phvul.003G109100	Pathogenesis-related protein Bet V I family	248.26 ±196.46	397.11 ±159.63	117.07 ±25.98	143.12 ±17.85	53.8 ±13.12	500.28 ±241.6	235.87 ±63.19	736.34 ±116.98
Phvul.006G069300	Glutamine-dependent asparagine synthetase	3.54 ±2.56	5.52 ±2.42	2.08 ±0.89	2.24 ±0.86	0.59 ±0.27	3.51 ±2.14	3.14 ±1.65	17.76 ±4.93
**CBB-susceptible RIL**									
Phvul.001G145800	Dirigent protein 1-related	1.76 ±1.44	3.69 ±0.7	1.48 ±0.15	1.79 ±0.47	0.23 ±0.14	3.19 ±0.3	3.11 ±1.09	3.84 ±0.56
Phvul.002G032866	Isoflavone reductase	2.35 ±1.34	2.92 ±0.72	0.57 ±0.17	1.08 ±0.19	0.35 ±0.16	2.53 ±1.13	1.21 ±0.6	2.11 ±0.91
Phvul.003G051700	Isoflavone synthase	2.62 ±1.39	5 ±1.15	0.63 ±0.1	1.38 ±0.48	0.59 ±0.23	2.01 ±0.8	2.15 ±1.13	3.97 ±2.03
Phvul.003G109100	Pathogenesis-related protein Bet V I family	208.02 ±164.91	498.09 ±37.11	94.87 ±14.26	145.84 ±70.05	46.96 ±3.04	390.61 ±56.52	130.24 ±33.47	153.47 ±49.16
Phvul.006G069300	Glutamine-dependent asparagine synthetase	22.4 ±15.7	47.09 ±8.23	3.68 ±1.47	2.77 ±1	2.4 ±0.66	33.35 ±16	4.03 ±1.44	4.92 ±1.93

Table S28. Transcript abundance for a gene upregulated in the CBB-resistant and CBB-susceptible RIL at 0 h post-inoculation (PI) with *Xanthomonas axonopodis* (*Xap*) and downregulated in the CBB-resistant RIL at 24 and 48 h PI. Transcript levels for individual genes corresponding to each RIL/ inoculation treatment/ sampling time PI are represented as FPKM ± SE of three experimental replicates.

		**Transcript levels (FPKM)**							
		**Mock inoculation**				***Xap* inoculation**			
**Gene ID**	**Gene Annotation**	**0 h PI**	**8 h PI**	**24 h PI**	**48 h PI**	**0 h PI**	**8 h PI**	**24 h PI**	**48 h PI**
**CBB-resistant RIL**									
Phvul.009G089300	Ethylene-responsive transcription factor ERF003	3.99 ± 2.11	2.82 ± 2.17	5.67 ± 4.49	1.02 ± 0.24	18.84 ± 4.02	5.28 ± 4.42	1.33 ± 0.23	0.25 ± 0.13
**CBB-susceptible RIL**									
Phvul.009G089300	Ethylene-responsive transcription factor ERF003	2.65 ± 1.14	0.89 ± 0.44	5.21 ± 1.54	1.84 ± 0.57	24.52 ± 6.14	1.2 ± 0.36	2.65 ± 0.51	5.29 ± 2.53

Table S29. Transcript abundance for a gene downregulated in the CBB-resistant RIL at 0 h post-inoculation (PI) with *Xanthomonas axonopodis* (*Xap*) and upregulated 48 h PI, and downregulated in the CBB-susceptible RIL at 0 h PI and upregulated 24 h PI. Transcript levels for individual genes corresponding to each RIL/ inoculation treatment/ sampling time PI are represented as FPKM ± SE of three experimental replicates.

		**Transcript levels (FPKM)**							
		**Mock inoculation**				***Xap* inoculation**			
**Gene ID**	**Gene Annotation**	**0 h PI**	**8 h PI**	**24 h PI**	**48 h PI**	**0 h PI**	**8 h PI**	**24 h PI**	**48 h PI**
**CBB-resistant RIL**									
Phvul.006G078400	No functional annotation	43.71 ± 41.03	43.73 ± 19.76	9.36 ± 3.59	11.85 ± 2.07	3.33 ± 0.77	47.71 ± 22.29	10.49 ± 2.18	69.64 ± 10.65
**CBB-susceptible RIL**									
Phvul.006G078400	No functional annotation	18.58 ± 14.84	76.27 ± 27.64	3.27 ± 0.57	45.25 ± 7.56	1.99 ± 0.52	43.85 ± 9.98	19.35 ± 14.73	14.14 ± 9.16

Table S30. Transcript abundance for a gene upregulated in the CBB-resistant RIL at 0 h post-inoculation (PI) with *Xanthomonas axonopodis* (*Xap*) and at all sampling times thereafter, and upregulated in the CBB-susceptible RIL at 0 h PI. Transcript levels for individual genes corresponding to each RIL/ inoculation treatment/ sampling time PI are represented as FPKM ± SE of three experimental replicates.

		Transcript levels (FPKM)							
		Mock inoculation				*Xap* inoculation			
**Gene ID**	**Gene Annotation**	**0 h PI**	**8 h PI**	**24 h PI**	**48 h PI**	**0 h PI**	**8 h PI**	**24 h PI**	**48 h PI**
**CBB-resistant RIL**									
Phvul.008G207300	Late embryogenesis abundant hydroxyproline-rich glycoprotein	14.63 ±4.78	6.79 ±2.11	3.51 ±0.14	14.08 ±10.15	58.94 ±12.31	28.52 ±22.4	33.76 ±16.6	61.88 ±45.85
**CBB-susceptible RIL**									
Phvul.008G207300	Late embryogenesis abundant hydroxyproline-rich glycoprotein	7.6 ±2.92	2.2 ±1.14	2.37 ±1.17	33.92 ±20.27	53.87 ±10.22	3.97 ±0.16	2.76 ±0.37	31.96± 19.49

Table S31. Transcript abundance for a gene upregulated in the CBB-resistant RIL at 8 h and 48 h post-inoculation (PI) with *Xanthomonas axonopodis* (*Xap*). Transcript levels for individual genes corresponding to each RIL/ inoculation treatment/ sampling time PI are represented as FPKM ± SE of three experimental replicates.

		Transcript levels (FPKM)							
		Mock inoculation				*Xap* inoculation			
**Gene ID**	**Gene Annotation**	**0 h PI**	**8 h PI**	**24 h PI**	**48 h PI**	**0 h PI**	**8 h PI**	**24 h PI**	**48 h PI**
Phvul.008G249900	Peroxidase / Lactoperoxidase	0.41 ±0.37	0.24 ±0.1	0.43 ±0.24	0.27 ±0.05	0.14 ±0.11	1.07 ±0.66	0.93 ±0.42	3.85 ±0.92

Table S32. Transcript abundance for a gene downregulated in the CBB-resistant RIL at 8 h post-inoculation (PI) with *Xanthomonas axonopodis* (*Xap*) and upregulated at 48 h PI. Transcript levels for individual genes corresponding to each RIL/ inoculation treatment/ sampling time PI are represented as FPKM ± SE of three experimental replicates.

		Transcript levels (FPKM)							
		Mock inoculation				*Xap* inoculation			
**Gene ID**	**Gene Annotation**	**0 h PI**	**8 h PI**	**24 h PI**	**48 h PI**	**0 h PI**	**8 h PI**	**24 h PI**	**48 h PI**
Phvul.009G158300, Phvul.009G158400	Calcium/Calmodulin-dependent protein kinase / Microtubule-associated protein 2 kinase	4.41 ±2.29	4.98 ±2.17	0.85 ±0.15	0.85 ±0.16	4.97 ±2.93	1.16 ±0.41	2.42 ±1.62	3.58 ±3.13

Table S33. Transcript abundance for a gene upregulated in the CBB-resistant RIL at 8 and 48 h post-inoculation (PI) with *Xanthomonas axonopodis* (*Xap*) and upregulated in the CBB-susceptible RIL at 0 h post-inoculation (PI).
Transcript levels for individual genes corresponding to each RIL/ inoculation treatment/ sampling time PI are represented as FPKM ± SE of three experimental replicates.

		Transcript levels (FPKM)							
		Mock inoculation				*Xap* inoculation			
**Gene ID**	**Gene Annotation**	**0 h PI**	**8 h PI**	**24 h PI**	**48 h PI**	**0 h PI**	**8 h PI**	**24 h PI**	**48 h PI**
**CBB-resistant RIL**									
Phvul.004G122000	Dehydration-responsive element-binding protein 1a-related	1.42 ±0.88	0.16 ±0.08	0.06 ±0.06	1.65 ±1.54	5.1 ±2.04	2.71 ±2.64	3.57 ±1.99	7.01 ±5.72
**CBB-susceptible RIL**									
Phvul.004G122000	Dehydration-responsive element-binding protein 1a-related	0.27 ±0.22	0.04 ±0.03	0.04 ±0.02	5.95 ±4.04	5.92 ±2.74	0.07 ±0.03	0.04 ±0.02	4.9 ±3.52

Table S34. Transcript abundance for genes respectively upregulated and downregulated in the CBB-resistant RIL at 24 and 48 h post-inoculation (PI) with *Xanthomonas axonopodis* (*Xap*). Transcript levels for individual genes corresponding to each RIL/ inoculation treatment/ sampling time PI are represented as FPKM ± SE of three experimental replicates.

		Transcript levels (FPKM)							
		Mock inoculation				*Xap* inoculation			
**Gene ID**	**Gene Annotation**	**0 h PI**	**8 h PI**	**24 h PI**	**48 h PI**	**0 h PI**	**8 h PI**	**24 h PI**	**48 h PI**
**Upregulated**									
Phvul.001G087100	GRAM domain	4.89 ±1.11	3.49 ±1.08	3.4 ±0.14	6.09 ±2.62	9.86 ±1.76	5.46 ±2.11	17.87 ±6.66	35.55 ±22.66
Phvul.001G067400	EF-hand calcium-binding domain containing protein	1.61 ±0.87	0.88 ±0.03	0.7 ±0.16	2.57 ±1.74	3.25 ±0.62	2.05 ±1.13	5.26 ±2.13	15.98 ±9.76
Phvul.001G194700, *Phvul.001G194900*	Harpin-induced protein-like-related	31.28 ±7.01	18.72 ±6.25	11.56 ±1.56	45.65 ±33.12	36.48 ±5.38	30.28 ±10.12	85.5 ±36.2	220.26 ±116.56
Phvul.002G293000	1-Aminocyclopropane-1-carboxylate synthase 2-related	3.23 ±0.5	2.2 ±0.62	1.42 ±0.27	3.07 ±2.27	3 ±0.42	2.71 ±0.75	7 ±3.33	14.69 ±7.89
Phvul.005G115200	No functional annotation	2.62 ±0.29	3.77 ±0.95	0.68 ±0.15	1.77 ±0.48	4.16 ±0.43	4.8 ±0.65	3.15 ±1.11	7.59 ±1.1
Phvul.007G029800	Core-2/I-branching β-1,6-N-acetylglucosaminyltransferase-like protein	0.29 ±0.08	0.14 ±0.02	0.1 ±0.03	0.35 ±0.2	0.08 ±0.06	0.07 ±0.05	0.75 ±0.33	3.05 ±1.34
Phvul.009G167200	No functional annotation	1.79 ±0.51	0.85 ±0.19	0.83 ±0.17	1.85 ±1.21	0.56 ±0.24	0.86 ±0.21	4.76 ±2.24	9.05 ±6.09
Phvul.010G090100	BON1-associated protein 1-related	0.52 ±0.24	0.41 ±0.27	2.82 ±0.71	4.02 ±0.7	0.29 ±0.09	0.38 ±0.09	3.89 ±1.62	10.24 ±5.81
Phvul.010G111900	WRKY DNA -binding domain	11.23 ±1.74	13.44 ±1.39	6.85 ±2.6	11.89 ±7.04	9.7 ±2.04	13.99 ±1.54	20.41 ±7.32	39.81 ±24.09
XLOC_023351	No functional annotation	5.32 ±1.92	4.22 ±1.93	0.24 ±0.1	0.48 ±0.2	7.24 ±0.76	5.46 ±1.64	12.23 ±3.35	24.96 ±15.32
XLOC_023352	No functional annotation	20.69 ±9.43	14.41 ±5.68	3.85 ±0.52	9.44 ±5.06	18.01 ±5.3	15.74 ±4.82	34.27 ±9.71	74.15 ±47.64

Table S34 continued

		Transcript levels (FPKM)							
		Mock inoculation				*Xap* inoculation			
**Gene ID**	**Gene Annotation**	**0 h PI**	**8 h PI**	**24 h PI**	**48 h PI**	**0 h PI**	**8 h PI**	**24 h PI**	**48 h PI**
**Downregulated**									
Phvul.008G003200	Pollen proteins Ole e I like	0.02 ±0.02	0.04 ±0.03	0.86 ±0.46	0.95 ±0.46	0.03 ±0.03	0.08 ±0.05	0.21 ±0.03	0.14 ±0.03

Table S35. Transcript abundance for genes upregulated in the CBB-resistant RIL at 24 h post-inoculation (PI) with *Xanthomonas axonopodis* (*Xap*) and at 0 h PI in the CBB-susceptible RIL. Transcript levels for individual genes corresponding to each RIL/ inoculation treatment/ sampling time PI are represented as FPKM ± SE of three experimental replicates.

		**Transcript levels (FPKM)**							
		**Mock inoculation**				***Xap* inoculation**			
**Gene ID**	**Gene Annotation**	**0 h PI**	**8 h PI**	**24 h PI**	**48 h PI**	**0 h PI**	**8 h PI**	**24 h PI**	**48 h PI**
**CBB-resistant RIL**									
Phvul.001G212300	Domain of unknown function	5.39 ±2.92	1.12 ±0.44	1.42 ±0.1	6.01 ±4.53	6.05 ±1.16	2.62 ±2.2	6.46 ±3.49	12.52 ±7.42
Phvul.003G066700	No functional annotation	3.34 ±1.68	1.11 ±0.42	0.49 ±0.3	4.35 ±4.12	5.6 ±1.24	4.05 ±3.09	7.08 ±3.65	13.66 ±9.72
Phvul.005G088600	Calcium binding protein	28.33 ±9.98	13.32 ±2.59	7.18 ±1.3	31.49 ±25.79	67.07 ±25.89	43.22 ±31.75	46.24 ±21.93	95.93 ±53.37
Phvul.010G062900	Methyltransferase	5.95 ±2.15	3.05 ±0.7	3.69 ±0.46	7.44 ±3.33	21.3 ±2.99	10.71 ±8.26	15.73 ±6.67	22.11 ±14.65
**CBB-susceptible RIL**									
Phvul.001G212300	Domain of unknown function	2.33 ±0.8	0.56 ±0.28	0.97 ±0.13	5.84 ±4.65	9.82 ±4.09	0.56 ±0.2	0.85 ±0.36	9.06 ±4.82
Phvul.003G066700	No functional annotation	0.7 ±0.42	0.72 ±0.19	0.76 ±0.13	7.4 ±6.48	3.71 ±0.91	0.61 ±0.04	0.55 ±0.25	5.62 ±2.6
Phvul.005G088600	Calcium binding protein	10.6 ±4.46	7.01 ±0.48	3.89 ±1.05	57.68 ±36.25	64.87 ±19.05	7.99 ±0.95	5.5 ±1.48	43.4 ±20.55
Phvul.010G062900	Methyltransferase	4.32 ±1.23	1.53 ±0.8	4.04 ±0.06	11.82 ±7.31	23.72 ±4.99	2.64 ±0.37	3.76 ±0.36	18.5 ±10.78

Table S36. Transcript abundance for genes upregulated in the CBB-resistant RIL at 24 h post-inoculation (PI) with *Xanthomonas axonopodis* (*Xap*) and in the CBB-susceptible RIL at 48 h PI. Transcript levels for individual genes corresponding to RIL/ inoculation treatment/ sampling time PI are represented as FPKM ± SE of three experimental replicates.

		Transcript levels (FPKM)							
		Mock inoculation				*Xap* inoculation			
**Gene ID**	**Gene Annotation**	**0 h PI**	**8 h PI**	**24 h PI**	**48 h PI**	**0 h PI**	**8 h PI**	**24 h PI**	**48 h PI**
**CBB-resistant RIL**									
Phvul.003G212700	AP2 domain	0.12 ±0.12	0 ±0	0 ±0	0 ±0	0 ±0	0.05 ±0.05	0.78 ±0.47	0 ±0
Phvul.004G011800	Pectin lyase-like superfamily protein	0 ±0	0 ±0	0 ±0	0 ±0	0 ±0	0 ±0	1.48 ±0.46	0.15 ±0.03
Phvul.007G066520	AP2 domain	1.67 ±0.39	1.06 ±0.17	0.4 ±0.16	0.5 ±0.11	0.97 ±0.2	0.96 ±0.05	3.6 ±1.91	1.35 ±1.01
**CBB-susceptible RIL**									
Phvul.003G212700	AP2 domain	0 ±0	0.11 ±0.05	0 ±0	0 ±0	0.44 ±0.44	0.09 ±0.09	0.13 ±0.13	1.13 ±1.13
Phvul.004G011800	Pectin lyase-like superfamily protein	0.06 ±0.03	0.02 ±0.01	0.05 ±0.03	0 ±0	0.11 ±0.11	0 ±0	2.09 ±1.62	1.73 ±1.3
Phvul.007G066520	AP2 domain	1.22 ±0.25	1.05 ±0.32	0.6 ±0.14	0.63 ±0.26	3.17 ±2.26	0.86 ±0.07	0.38 ±0.07	4.35 ±3.78

Table S37. Transcript abundance for genes upregulated in the CBB-resistant RIL at 24 and 48 h post-inoculation (PI) with *Xanthomonas axonopodis* (*Xap*) and at 0 h PI in the CBB-susceptible RIL. Transcript levels for individual genes corresponding to each RIL/ inoculation treatment/ sampling time PI are represented as FPKM ± SE of three experimental replicates.

		Transcript levels (FPKM)							
		Mock inoculation				*Xap* inoculation			
**Gene ID**	**Gene Annotation**	**0 h PI**	**8 h PI**	**24 h PI**	**48 h PI**	**0 h PI**	**8 h PI**	**24 h PI**	**48 h PI**
**CBB-resistant RIL**									
Phvul.004G022700	Galacturonosyltransferase-like 8-related	0.95 ±0.33	0.35 ±0.14	0.13 ±0.02	0.82 ±0.73	1.88 ±0.1	1 ±0.61	1.98 ±1.28	4.04 ±2.52
XLOC_009124	No functional annotation	2 ±0.88	0.79 ±0.54	0.24 ±0.02	4.36 ±3.98	1.57 ±0.36	0.99 ±0.53	12.63 ±6.81	30.37 ±20.77
XLOC_023518	No functional annotation	1.85 ±0.6	0.52 ±0.08	0.27 ±0.09	1.14 ±0.78	1.27 ±0.36	1.59 ±0.86	3.57 ±1.95	10.64 ±7.39
**CBB-susceptible RIL**									
Phvul.004G022700	Galacturonosyltransferase-like 8-related	0.42 ±0.14	0.23 ±0.03	0.21 ±0.07	1.57 ±1.2	2.04 ±1.13	0.17 ±0.06	0.14 ±0.01	1.37 ±0.84
XLOC_009124	No functional annotation	0.52 ±0.21	0.27 ±0.03	0.21 ±0.09	15.41 ±14.6	3.36 ±2.38	0.14 ±0.05	0.19 ±0.05	13.3 ±7.66
XLOC_023518	No functional annotation	0.68 ±0.11	0.31 ±0.16	0.33 ±0.02	5.11 ±4.64	3.12 ±1.47	0.49 ±0.07	0.27 ±0.05	5.22 ±3.75

Table S38. Transcript abundance for a gene upregulated in the CBB-resistant RIL at 24 and 48 h post-inoculation (PI) with *Xanthomonas axonopodis* (*Xap*) and downregulated at 48 h PI in the CBB-susceptible RIL. Transcript levels for individual genes corresponding to each RIL/ inoculation treatment/ sampling time PI are represented as FPKM ± SE of three experimental replicates.

		Transcript levels (FPKM)							
		Mock inoculation				*Xap* inoculation			
**Gene ID**	**Gene Annotation**	**0 h PI**	**8 h PI**	**24 h PI**	**48 h PI**	**0 h PI**	**8 h PI**	**24 h PI**	**48 h PI**
**CBB-resistant RIL**									
Phvul.002G177900	Galacturonosyltransferase-like 8-related	0.1 ±0.06	0.55 ±0.33	0.11 ±0.02	0.25 ±0.05	0.24 ±0.08	0.19 ±0.1	0.62 ±0.06	4.71 ±1.74
**CBB-susceptible RIL**									
Phvul.002G177900	Galacturonosyltransferase-like 8-related	0.19 ±0.02	0.07 ±0.03	0.16 ±0.04	0.68 ±0.35	0.31 ±0.14	0.09 ±0.02	0.06 ±0.03	0.12 ±0.03

Table S39. Transcript abundance for a gene upregulated in the CBB-resistant RIL at 24 h post-inoculation (PI) with *Xanthomonas axonopodis* (*Xap*) and in the CBB-susceptible RIL at 24 and 48 h PI. Transcript levels for individual genes corresponding to each RIL/ inoculation treatment/ sampling time PI are represented as FPKM ± SE of three experimental replicates.

		Transcript levels (FPKM)							
		Mock inoculation				*Xap* inoculation			
**Gene ID**	**Gene Annotation**	**0 h PI**	**8 h PI**	**24 h PI**	**48 h PI**	**0 h PI**	**8 h PI**	**24 h PI**	**48 h PI**
**CBB-resistant RIL**									
Phvul.008G144100	β-expansin 6-related	0.13 ±0.13	0.02 ±0.02	0.11 ±0.06	0.16 ±0.09	0 ±0	0.08 ±0.06	7.51 ±1.55	0.96 ±0.19
**CBB-susceptible RIL**									
Phvul.008G144100	β-expansin 6-related	0.04 ±0.02	0.03 ±0.03	0.2 ±0.05	0.18 ±0.1	0.03 ±0.02	0.05 ±0.05	13.7 ±9	12.07 ±8.83

Table S40. Transcript abundance for a gene upregulated in both RILs at 24 and 48 h post-inoculation (PI) with *Xanthomonas axonopodis* (*Xap*). Transcript levels for individual genes corresponding to each RIL/ inoculation treatment/ sampling time PI are represented as FPKM ± SE of three experimental replicates.

		**Transcript levels (FPKM)**							
		**Mock inoculation**				***Xap* inoculation**			
**Gene ID**	**Gene Annotation**	**0 h PI**	**8 h PI**	**24 h PI**	**48 h PI**	**0 h PI**	**8 h PI**	**24 h PI**	**48 h PI**
**CBB-resistant RIL**									
Phvul.007G099700	No functional annotation	0 ±0	0.03 ±0.03	0.34 ±0.12	0.86 ±0.38	0.04 ±0.03	0.05 ±0.05	17.6 ±5.83	22.03 ±3.6
**CBB-susceptible RIL**									
Phvul.007G099700	No functional annotation	0 ±0	0.05 ±0.02	0.32 ±0.2	0.16 ±0	0 ±0	0.03 ±0.02	29.87 ±25.63	15.02 ±11.5

Table S41. Transcript abundance for genes upregulated in the CBB-resistant RIL at 48 h post-inoculation (PI) *Xanthomonas axonopodis* (*Xap*) and downregulated in the CBB-susceptible RIL at 0 h PI. Transcript levels for individual genes corresponding to each RIL/ inoculation treatment/ sampling time PI are represented as FPKM ± SE of three experimental replicates.

		**Transcript levels (FPKM)**							
		**Mock inoculation**				***Xap* inoculation**			
**Gene ID**	**Gene Annotation**	**0 h PI**	**8 h PI**	**24 h PI**	**48 h PI**	**0 h PI**	**8 h PI**	**24 h PI**	**48 h PI**
**CBB-resistant RIL**									
Phvul.001G145600	Dirigent protein 1-related	0.31 ±0.13	0.2 ±0.12	0.71 ±0.33	0.26 ±0.13	0.14 ±0.1	0.32 ±0.2	1.94 ±1.4	8.73 ±3.53
Phvul.002G038600, Phvul.002G038700, Phvul.002G038800, Phvul.002G038900, Phvul.002G039000, Phvul.002G039300	Chalcone synthase	1.28 ±0.76	1.49 ±0.14	1.8 ±0.77	1.31 ±0.46	0.34 ±0.05	1.8 ±0.69	4.12 ±1.52	26.91 ±2.34
Phvul.004G085676	Nudix hydrolase related	0.16 ±0.04	0.39 ±0.11	0.48 ±0.14	0.67 ±0.28	0.27 ±0.1	0.31 ±0.13	0.92 ±0.23	13.39 ±4.94
Phvul.006G197100, Phvul.006G197200, Phvul.006G197300	Pathogenesis-related protein 1 (PR1)	11.29 ±3.76	9.58 ±2.62	13.91 ±5.88	10 ±4.91	7.08 ±1.87	11.86 ±1.67	11.94 ±3.66	326.38 ±17.06
Phvul.008G032200	Isoflavone-7-*O*-β-glucoside 6"-*O*-malonyltransferase	4.05 ±2.97	7.02 ±2.62	0.71 ±0.08	0.99 ±0.3	1.27 ±0.08	6.37 ±2.71	1.01 ±0.17	4.78 ±1.68
Phvul.009G043200	WRKY transcription factor 38-related	0.32 ±0.18	0.58 ±0.28	0.42 ±0.09	0.37 ±0.14	0.21 ±0.06	0.31 ±0.05	0.33 ±0.09	2.33 ±1.29
Phvul.010G042200	Hydroquinone glucosyltransferase	1.61 ±1.15	2.97 ±1.2	0.87 ±0.27	1.56 ±0.5	0.64 ±0.09	3.22 ±1.59	1.67 ±0.68	8.91 ±2.7
**CBB-susceptible RIL**									
Phvul.001G145600	Dirigent protein 1-related	0.55 ±0.1	0.3 ±0.2	0.04 ±0.04	0.32 ±0.06	0 ±0	0.06 ±0.06	0.03 ±0.03	0.85 ±0.82

Table S41 continued

		Transcript levels (FPKM)							
		Mock inoculation				*Xap* inoculation			
**Gene ID**	**Gene Annotation**	**0 h PI**	**8 h PI**	**24 h PI**	**48 h PI**	**0 h PI**	**8 h PI**	**24 h PI**	**48 h PI**
Phvul.002G038600, Phvul.002G038700, Phvul.002G038800, Phvul.002G038900, Phvul.002G039000, Phvul.002G039300	Chalcone synthase	1.18 ±0.5	2.04 ±0.31	0.64 ±0.29	0.65 ±0.04	0.16 ±0.05	1.07 ±0.28	0.77 ±0.18	1.33 ±0.67
Phvul.004G085676	Nudix hydrolase related	0.76 ±0.58	0.4 ±0.08	0.18 ±0.03	3.2 ±2	0.13 ±0.04	0.18 ±0.08	0.35 ±0.24	0.89 ±0.7
Phvul.006G197100, Phvul.006G197200, Phvul.006G197300	Pathogenesis-related protein 1 (PR1)	29.26 ±14.35	33.09 ±9.95	16.54 ±14.69	16.99 ±5.87	7.23 ±3.76	14.28 ±5.12	17.66 ±13.68	24.78 ±13.82
Phvul.008G032200	Isoflavone-7-*O*-β-glucoside 6"-*O*-malonyltransferase	3.62 ±2.75	7.59 ±3.82	0.49 ±0.04	0.84 ±0.22	0.85 ±0.18	9.19 ±0.48	0.61 ±0.11	1.02 ±0.54
Phvul.009G043200	WRKY transcription factor 38-related	0.61 ±0.48	0.15 ±0.08	0.14 ±0.06	1.35 ±0.61	0.1 ±0.02	0.11 ±0.02	0.13 ±0.07	0.35 ±0.23
Phvul.010G042200	Hydroquinone glucosyltransferase	2.37 ±1.67	2.74 ±1.51	0.3 ±0.02	1.4 ±0.6	0.4 ±0.04	4.23 ±0.33	0.96 ±0.65	1.95 ±1.39

Table S42. Transcript abundance for genes upregulated in the CBB-resistant RIL at 48 h post-inoculation (PI) with *Xanthomonas axonopodis* (*Xap*) and downregulated in the CBB-susceptible RIL at 8 h PI. Transcript levels for individual genes corresponding to each RIL/ inoculation treatment/ sampling time PI are represented as FPKM ± SE of three experimental replicates.

		**Transcript levels (FPKM)**							
		**Mock inoculation**				***Xap* inoculation**			
**Gene ID**	**Gene Annotation**	**0 h PI**	**8 h PI**	**24 h PI**	**48 h PI**	**0 h PI**	**8 h PI**	**24 h PI**	**48 h PI**
**CBB-resistant RIL**									
Phvul.002G200600	Tetrahydroberberine oxidase	0.28 ±0.13	0.13 ±0.06	1.72 ±1.07	0.56 ±0.13	0 ±0	0.13 ±0.05	1.06 ±0.55	32.71 ±1.73
Phvul.004G098000	Extensin-like region	6.02 ±1.89	3.08 ±1.23	16.74 ±6.93	12.5 ±1.31	3.17 ±0.68	2.01 ±0.5	11.5 ±2.25	255.16 ±7.49
**CBB-susceptible RIL**									
Phvul.002G200600	Tetrahydroberberine oxidase	0.77 ±0.36	1.28 ±0.13	0.38 ±0.33	0.54 ±0.24	0.05 ±0.05	0.28 ±0.19	0.45 ±0.32	1 ±0.53
Phvul.004G098000	Extensin-like region	8.16 ±1.69	15.51 ±6.44	6.75 ±5.26	13.13 ±4.77	2.66 ±0.82	3.7 ±0.84	19.74 ±15.14	16.31 ±6.8

Table S43. Transcript abundance for genes upregulated in the CBB-resistant RIL at 48 h post-inoculation (PI) with *Xanthomonas axonopodis* (*Xap*) and in the CBB-susceptible RIL at 24 h PI. Transcript levels for individual genes corresponding to each RIL/ inoculation treatment/ sampling time PI are represented as FPKM ± SE of three experimental replicates.

		**Transcript levels (FPKM)**							
		**Mock inoculation**				***Xap* inoculation**			
**Gene ID**	**Gene Annotation**	**0 h PI**	**8 h PI**	**24 h PI**	**48 h PI**	**0 h PI**	**8 h PI**	**24 h PI**	**48 h PI**
**CBB-resistant RIL**									
Phvul.006G196900	Pathogenesis-related protein 1 (PR1)	3.93 ±2.54	7.62 ±2.2	1.99 ±0.42	2.32 ±0.19	1.79 ±0.72	9.2 ±2.49	2.75 ±1.06	33.99 ±7.53
Phvul.006G124600, Phvul.006G124700	FAD-binding berberine family protein-related	0.11 ±0.11	0.08 ±0.01	0.33 ±0.13	0.22 ±0.05	0.08 ±0.06	0.12 ±0.03	0.35 ±0.16	1.67 ±0.32
Phvul.001G128500	β-1,3-glucanase 1-related	32.88 ±25.47	40.37 ±14.4	41.76 ±14.98	126.99 ±13.57	8.31 ±2.77	35.24 ±16.65	89.48 ±27.66	559.47 ±136.63
**CBB-susceptible RIL**									
Phvul.006G196900	Pathogenesis-related protein 1 (PR1)	1.44 ±0.8	1.76 ±0.83	0.66 ±0.25	7.51 ±3.5	0.89 ±0.21	1.35 ±0.36	3.58 ±2.06	2.4 ±1.18
Phvul.006G124600, Phvul.006G124700	FAD-binding berberine family protein-related	0.15 ±0.08	0.16 ±0.04	0.27 ±0.05	1.56 ±0.57	0.02 ±0.02	0.14 ±0.06	1.16 ±0.99	0.49 ±0.28
Phvul.001G128500	β-1,3-glucanase 1-related	22.22 ±13.78	53.86 ±14.8	15.37 ±1.13	110.11 ±54.43	6.18 ±1.02	45.09 ±17.01	96.64 ±74.85	85.44 ±57.83

Table S44. Transcript abundance for genes that were adversely regulated across both RILs at 48 h post-inoculation (PI) with *Xanthomonas axonopodis* (*Xap*). Transcript levels for individual genes corresponding to each RIL/ inoculation treatment/ sampling time PI are represented as FPKM ± SE of three experimental replicates.

		Transcript levels (FPKM)							
		Mock inoculation				*Xap* inoculation			
**Gene ID**	**Gene Annotation**	**0 h PI**	**8 h PI**	**24 h PI**	**48 h PI**	**0 h PI**	**8 h PI**	**24 h PI**	**48 h PI**
**CBB-resistant RIL upregulated**									
Phvul.001G036600	Fructan β-(2,1)-fructosidase / Inulinase	0.08 ±0.01	0.11 ±0.06	0.09 ±0.02	0.06 ±0.04	0.15 ±0.08	0.08 ±0.03	0.08 ±0.05	0.7 ±0.29
Phvul.001G205900	Protein C41G7.9, isoform B	0.24 ±0.09	0.1 ±0.05	0.13 ±0.02	0.18 ±0.13	0.18 ±0.12	0.09 ±0.09	0.18 ±0.05	8.93 ±8.64
Phvul.001G255200	RING/U-box domain-containing protein	0.93 ±0.77	0.1 ±0.06	0.45 ±0.25	1.41 ±1.35	0.85 ±0.09	0.25 ±0.18	1.08 ±0.54	20.25 ±18.02
Phvul.002G155400	Thaumatin family	0.64 ±0.3	0.17 ±0.04	1.88 ±0.57	0.75 ±0.39	0.08 ±0.04	0.09 ±0.05	1.35 ±0.68	21.48 ±1.55
Phvul.002G309300	Ring zinc finger protein	0.3 ±0.15	0.23 ±0.06	0.28 ±0.14	0 ±0	0.14 ±0.07	0.32 ±0.16	0.06 ±0.06	0.63 ±0.27
Phvul.003G128133	Flavin-containing monooxygenase 1-related	0 ±0	0.08 ±0.04	0.4 ±0.3	0.36 ±0.15	0 ±0	0.11 ±0.03	0.41 ±0.36	5.26 ±0.34
Phvul.003G131500	12-Oxophytodienoate reductase 3	0.58 ±0.1	1.55 ±0.2	0.42 ±0.08	0.12 ±0.06	0.64 ±0.07	0.85 ±0.08	0.74 ±0.44	2.59 ±0.84
Phvul.005G053100	Glutathione S-transferase U21-related	0.64 ±0.09	0.67 ±0.21	0.32 ±0.18	0.55 ±0.14	0.74 ±0.13	0.68 ±0.17	1.21 ±0.43	5.35 ±3.44
Phvul.005G108900	Transferase family	1.18 ±0.16	1.52 ±0.78	0.58 ±0.12	1.23 ±0.14	1.46 ±0.24	1.16 ±0.16	0.9 ±0.16	5.63 ±1.8
Phvul.007G066300	No functional annotation	0.43 ±0.25	0.39 ±0.2	0.29 ±0.17	1.27 ±0.31	0.46 ±0.14	0.44 ±0.25	0.7 ±0.27	9.07 ±4
Phvul.007G111200	Calcium binding protein	1.83 ±0.37	1.62 ±0.77	1.14 ±0.05	1.4 ±0.24	2.16 ±0.65	1.52 ±0.9	1.79 ±0.47	7.84 ±4.72
Phvul.010G093500	BON1-associated protein 1-related	0.75 ±0.14	1.09 ±0.1	1.02 ±0.45	0.35 ±0.31	0.58 ±0.16	1.1 ±0.44	2 ±0.58	3.77 ±0.51

Table S44 continued

		Transcript levels (FPKM)							
		Mock inoculation				*Xap* inoculation			
**Gene ID**	**Gene Annotation**	**0 h PI**	**8 h PI**	**24 h PI**	**48 h PI**	**0 h PI**	**8 h PI**	**24 h PI**	**48 h PI**
**CBB-resistant RIL downregulated**									
Phvul.003G169100	Hydrophobic seed protein	40.5 ±15.84	36.23 ±2.21	38.49 ±23.19	17.51 ±4.98	49.37 ±15.77	39.43 ±1.84	23.64 ±3.52	3.65 ±1.59
Phvul.008G076300	4,4-dimethyl-9β,19-cyclopropylsterol-4-α-methyl oxidase (SMO1)	9.38 ±0.39	10.9 ±1.32	3.67 ±1.65	1.82 ±0.84	9.39 ±1.02	11.39 ±0.81	5.63 ±0.57	0.41 ±0.25
Phvul.011G026700	Proline-rich protein 4	1087.49 ±125.66	1139.65 ±82.56	366.15 ±245.7	118.78 ±68.29	1146.18 ±146.68	1076.04 ±33.82	258.72 ±64.09	21.66 ±5.21
**CBB-susceptible RIL upregulated**									
Phvul.002G155400	Thaumatin family	0.82 ±0.37	1.11 ±0.2	0.61 ±0.47	0.38 ±0.13	0.25 ±0.15	0.4 ±0.22	0.35 ±0.18	1.83 ±1.2
Phvul.003G131500	12-Oxophytodienoate reductase 3	0.49 ±0.1	0.61 ±0.12	0.2 ±0.02	0.11 ±0.05	0.96 ±0.13	1.01 ±0.27	0.36 ±0.07	0.66 ±0.22
Phvul.003G169100	Hydrophobic seed protein	29.92 ±3.01	29.26 ±4.95	37.43 ±13.05	4.73 ±1.89	36.37 ±5.23	20.98 ±1.5	19.28 ±9.7	21.51 ±9.65
Phvul.008G076300	4,4-dimethyl-9β,19-cyclopropylsterol-4α-methyl oxidase (SMO1)	7.21 ±2.32	7.16 ±3.66	6.54 ±2.22	0.38 ±0.09	6.59 ±1.01	7.39 ±1.52	2.91 ±1.36	3.59 ±1.75
Phvul.010G093500	BON1-associated protein 1-related	0.75 ±0.51	0.72 ±0.39	0.74 ±0.24	0.15 ±0.03	0.45 ±0.13	1.1 ±0.33	0.61 ±0.19	1.57 ±0.58
Phvul.011G026700	Proline-rich protein 4	1004.13 ±58.08	662.57 ±331.68	404.29 ±72.89	24.11 ±4.45	1109.66 ±120.05	863.29 ±204.69	235.91 ±130.63	245.5 ±131.52
**CBB-susceptible RIL downregulated**									
Phvul.001G036600	Fructan β-(2,1)-fructosidase / Inulinase	0.31 ±0.16	0.18 ±0.12	0.15 ±0.02	1.19 ±0.73	0.13 ±0.05	0.09 ±0.02	0.12 ±0.08	0.3 ±0.17

Table S44 continued

		Transcript levels (FPKM)							
		Mock inoculation				*Xap* inoculation			
**Gene ID**	**Gene Annotation**	**0 h PI**	**8 h PI**	**24 h PI**	**48 h PI**	**0 h PI**	**8 h PI**	**24 h PI**	**48 h PI**
Phvul.001G205900	Protein C41G7.9, isoform B	0.46 ±0.25	0 ±0	0.29 ±0.07	3.35 ±3.09	0.42 ±0.01	0 ±0	0.14 ±0.03	0.52 ±0.53
Phvul.001G255200	RING/U-box domain-containing protein	0.54 ±0.38	0.05 ±0.03	0.39 ±0.18	14.56 ±10.89	0.96 ±0.81	0.03 ±0.02	0.12 ±0.08	1.97 ±1.45
Phvul.002G309300	Ring zinc finger protein	0.14 ±0.14	0.36 ±0.12	0.12 ±0.06	0.61 ±0.24	0.07 ±0.08	0.48 ±0.25	0.2 ±0.2	0 ±0
Phvul.003G128133	Flavin-containing monooxygenase 1-related	0.02 ±0.02	0.01 ±0.01	0.06 ±0.05	1.7 ±1.1	0.05 ±0.03	0.14 ±0.13	0.29 ±0.18	0.34 ±0.23
Phvul.005G053100	Glutathione S-transferase U21-related	0.73 ±0.18	0.15 ±0.02	0.11 ±0.03	2.32 ±1.53	0.44 ±0.09	0.21 ±0.03	0.12 ±0.09	0.29 ±0.1
Phvul.005G108900	Transferase family	1.86 ±1.15	0.53 ±0.07	0.33 ±0.08	3.28 ±1.97	0.75 ±0.27	0.43 ±0.03	0.57 ±0.29	0.57 ±0.32
Phvul.007G066300	No functional annotation	0.75 ±0.4	0.44 ±0.14	0.06 ±0.06	7.59 ±5.42	0.07 ±0.07	0.04 ±0.04	0.73 ±0.38	1.29 ±0.92
Phvul.007G111200	Calcium binding protein	1.25 ±0.48	0.71 ±0.07	0.83 ±0.13	6.86 ±4.51	1.1 ±0.16	0.53 ±0.14	0.59 ±0.22	1.16 ±0.44

Table S45. Transcript abundance for a gene upregulated in the CBB-resistant RIL at 48 h post-inoculation (PI) with *Xanthomonas axonopodis* (*Xap*) and downregulated at 0 h PI and upregulated at 24 h PI in the CBB-susceptible RIL. Transcript levels for individual genes corresponding to each RIL/ inoculation treatment/ sampling time PI are represented as FPKM ± SE of three experimental replicates.

		**Transcript levels (FPKM)**							
		**Mock inoculation**				***Xap* inoculation**			
**Gene ID**	**Gene Annotation**	**0 h PI**	**8 h PI**	**24 h PI**	**48 h PI**	**0 h PI**	**8 h PI**	**24 h PI**	**48 h PI**
**CBB-resistant RIL**									
Phvul.001G145700	Dirigent protein 1-related	0.64 ±0.42	0.29 ±0.14	0.8 ±0.33	1.13 ±0.33	0.14 ±0.14	0.15 ±0.12	0.25 ±0.11	102.18 ±10.58
**CBB-susceptible RIL**									
Phvul.001G145700	Dirigent protein 1-related	0.53 ±0.2	0.94 ±0.74	0.27 ±0.12	1.43 ±0.81	0 ±0	0.29 ±0.23	1.38 ±0.96	1.07 ±0.64

Table S46. Transcript abundance for a gene upregulated in the CBB-resistant RIL at 48 h post-inoculation (PI) with *Xanthomonas axonopodis* (*Xap*) and downregulated at 0 h PI and upregulated at 48 h PI in the CBB-susceptible RIL. Transcript levels for individual genes corresponding to each RIL/ inoculation treatment/ sampling time PI are represented as FPKM ± SE of three experimental replicates.

		**Transcript levels (FPKM)**							
		**Mock inoculation**				***Xap* inoculation**			
**Gene ID**	**Gene Annotation**	**0 h PI**	**8 h PI**	**24 h PI**	**48 h PI**	**0 h PI**	**8 h PI**	**24 h PI**	**48 h PI**
**CBB-resistant RIL**									
Phvul.009G244100	Isoflavone 2'-hydroxylase	1.6 ±1.35	1.09 ±0.47	1.2 ±0.22	1.13 ±0.24	0.02 ±0.02	1.66 ±0.82	2.04 ±0.53	10.51 ±1.19
**CBB-susceptible RIL**									
Phvul.009G244100	Isoflavone 2'-hydroxylase	1.34 ±1.28	1.33 ±0.76	0.5 ±0.13	0.21 ±0.08	0.12 ±0.08	1.16 ±0.2	0.87 ±0.2	1.52 ±0.47

Table S47. Transcript abundance for a gene downregulated in the CBB-resistant RIL at 48 h post-inoculation (PI) with *Xanthomonas axonopodis* (*Xap*) and downregulated at 24 h PI and upregulated at 48 h PI in the CBB-susceptible RIL. Transcript levels for individual genes corresponding to each RIL/ inoculation treatment/ sampling time PI are represented as FPKM ± SE of three experimental replicates.

		Transcript levels (FPKM)							
		Mock inoculation				*Xap* inoculation			
**Gene ID**	**Gene Annotation**	**0 h PI**	**8 h PI**	**24 h PI**	**48 h PI**	**0 h PI**	**8 h PI**	**24 h PI**	**48 h PI**
**CBB-resistant RIL**									
Phvul.006G035050, Phvul.006G035100	No functional annotation	0.85 ±0.16	1.09 ±0.23	0.64 ±0.06	3.14 ±2.29	3.11 ±2.53	3.64 ±1.48	0.62 ±0.04	0.52 ±0.08
**CBB-susceptible RIL**									
Phvul.006G035050, Phvul.006G035100	No functional annotation	0.87 ±0.1	2.66 ±1.14	7.26 ±4.39	0.55 ±0.09	0.61 ±0.01	1.36 ±0.16	0.72 ±0.05	2.59 ±1.82

Table S48. Transcript abundance for a gene downregulated in the CBB-susceptible RIL at 0 h post-inoculation (PI) with *Xanthomonas axonopodis* (*Xap*) and upregulated at 8 h PI. Transcript levels for individual genes corresponding to each RIL/ inoculation treatment/ sampling time PI are represented as FPKM ± SE of three experimental replicates.

		Transcript levels (FPKM)							
		Mock inoculation				*Xap* inoculation			
**Gene ID**	**Gene Annotation**	**0 h PI**	**8 h PI**	**24 h PI**	**48 h PI**	**0 h PI**	**8 h PI**	**24 h PI**	**48 h PI**
XLOC_008103	No functional annotation	0.66 ±0.66	0 ±0	0 ±0	0.38 ±0.32	0 ±0	0.97 ±0.54	0.41 ±0.41	0 ±0

Table S49. Transcript abundance for a gene upregulated in the CBB-susceptible RIL at 0 h and 48 h post-inoculation (PI) with *Xanthomonas axonopodis* (*Xap*). Transcript levels for individual genes corresponding to each RIL/ inoculation treatment/ sampling time PI are represented as FPKM ± SE of three experimental replicates.

		Transcript levels (FPKM)							
		Mock inoculation				*Xap* inoculation			
**Gene ID**	**Gene Annotation**	**0 h PI**	**8 h PI**	**24 h PI**	**48 h PI**	**0 h PI**	**8 h PI**	**24 h PI**	**48 h PI**
Phvul.003G268200	No functional annotation	0.42 ±0.08	0.11 ±0.01	0.2 ±0.12	0.28 ±0.18	2.33 ±1.13	0.12 ±0.07	0.07 ±0.02	1.25 ±0.94

Table S50. Transcript abundance for a gene upregulated in the CBB-susceptible RIL at 0 h post-inoculation (PI) with *Xanthomonas axonopodis* (*Xap*) and at 8 and 24 h PI and downregulated at 48 h PI. Transcript levels for individual genes corresponding to each RIL/ inoculation treatment/ sampling time PI are represented as FPKM ± SE of three experimental replicates.

		Transcript levels (FPKM)							
		Mock inoculation				*Xap* inoculation			
**Gene ID**	**Gene Annotation**	**0 h PI**	**8 h PI**	**24 h PI**	**48 h PI**	**0 h PI**	**8 h PI**	**24 h PI**	**48 h PI**
XLOC_015635	No functional annotation	0 ±0	0 ±0	0 ±0	1.8 ±1.8	2.2 ±1.1	0.8 ±0.8	1.09 ±1.1	0 ±0

Table S51. Transcript abundance for genes adversely regulated (up or up and down) in the CBB-susceptible RIL at 8 h and 24 h post-inoculation (PI) with *Xanthomonas axonopodis* (*Xap*). Transcript levels for individual genes corresponding to each RIL/ inoculation treatment/ sampling time PI are represented as FPKM ± SE of three experimental replicates.

		Transcript levels (FPKM)							
		Mock inoculation				*Xap* inoculation			
**Gene ID**	**Gene Annotation**	**0 h PI**	**8 h PI**	**24 h PI**	**48 h PI**	**0 h PI**	**8 h PI**	**24 h PI**	**48 h PI**
**Upregulated**									
XLOC_020701	No functional annotation	0.7 ±0.35	0 ±0	0 ±0	1.24 ±0.25	0.39 ±0.39	1.36 ±0.39	2.8 ±0.51	1.08 ±0.54
**Downregulated 8 h PI and upregulated 24 h PI**									
Phvul.004G092694	Light-harvesting complex II chlorophyll a/b binding protein 1 (LHCB1)	5.87 ±3.49	0.66 ±0.33	0.59 ±0.52	0.5 ±0.28	2.6 ±2.03	0 ±0	2.68 ±1.94	1.26 ±1.26

Table S52. Gene ontology (GO) terms enriched across the differentially expressed genes of the CBB-susceptible RIL at 0 h post-inoculation with *Xanthomonas axonopodis* (*Xap*).

Gene ID	GO Term	Description
Phvul.001G124300	GO:0004616	Phosphogluconate dehydrogenase (decarboxylating) activity
	GO:0004735	Pyrroline-5-carboxylate reductase activity
	GO:0006098	Pentose-phosphate shunt
	GO:0006561	Proline biosynthetic process
	GO:0016491	Oxidoreductase activity
	GO:0055114	Oxidation-reduction process
Phvul.002G038600	GO:0004315	3-Oxoacyl-[acyl-carrier-protein] synthase activity
	GO:0006633	Fatty acid biosynthetic process
	GO:0008415	Metabolic process
	GO:0016020	Membrane
Phvul.002G038700	GO:0004315	3-Oxoacyl-[acyl-carrier-protein] synthase activity
	GO:0006633	Fatty acid biosynthetic process
	GO:0008415	Metabolic process
	GO:0016020	Membrane
Phvul.002G038800	GO:0008415	Metabolic process
	GO:0008610	Lipid biosynthetic process
Phvul.002G038900	GO:0004315	3-Oxoacyl-[acyl-carrier-protein] synthase activity
	GO:0006633	Fatty acid biosynthetic process
	GO:0008415	Metabolic process
	GO:0016020	Membrane
	GO:0008415	Metabolic process
	GO:0008610	Lipid biosynthetic process
Phvul.002G039300	GO:0004315	3-Oxoacyl-[acyl-carrier-protein] synthase activity
	GO:0006633	Fatty acid biosynthetic process
	GO:0008415	Metabolic process
	GO:0016020	Membrane
Phvul.002G077400	GO:0016757	Transferase activity, transferring glycosyl groups
Phvul.002G096000	GO:0005975	Carbohydrate metabolic process
	GO:0016868	Intramolecular transferase activity, phosphotransferases
Phvul.003G052400	GO:0004553	Hydrolase activity, hydrolyzing *O*-glycosyl compounds
	GO:0005618	Cell wall
	GO:0005975	Carbohydrate metabolic process
	GO:0006073	Cellular glucan metabolic process
	GO:0016762	Xyloglucan:xyloglucosyl transferase activity
	GO:0048046	Apoplast
Phvul.003G079900	GO:0004553	Hydrolase activity, hydrolyzing *O*-glycosyl compounds
	GO:0005975	Carbohydrate metabolic process

Table S52 continued

Gene ID	GO term	Description
Phvul.003G137600	GO:0004553	Hydrolase activity, hydrolyzing *O*-glycosyl compounds
	GO:0005618	Cell wall
	GO:0005975	Carbohydrate metabolic process
	GO:0006073	Cellular glucan metabolic process
	GO:0016762	Xyloglucan:xyloglucosyl transferase activity
	GO:0048046	Apoplast
Phvul.003G147300	GO:0004553	Hydrolase activity, hydrolyzing *O*-glycosyl compounds
	GO:0005618	Cell wall
	GO:0005975	Carbohydrate metabolic process
	GO:0006073	Cellular glucan metabolic process
	GO:0016762	Xyloglucan:xyloglucosyl transferase activity
	GO:0048046	Apoplast
Phvul.003G147500	GO:0004553	Hydrolase activity, hydrolyzing *O*-glycosyl compounds
	GO:0005618	Cell wall
	GO:0005975	Carbohydrate metabolic process
	GO:0006073	Cellular glucan metabolic process
	GO:0016762	Xyloglucan:xyloglucosyl transferase activity
	GO:0048046	Apoplast
Phvul.003G147600	GO:0004553	Hydrolase activity, hydrolyzing *O*-glycosyl compounds
	GO:0005618	Cell wall
	GO:0005975	Carbohydrate metabolic process
	GO:0006073	Cellular glucan metabolic process
	GO:0016762	Xyloglucan:xyloglucosyl transferase activity
	GO:0048046	Apoplast
Phvul.003G147700	GO:0004553	Hydrolase activity, hydrolyzing *O*-glycosyl compounds
	GO:0005618	Cell wall
	GO:0005975	Carbohydrate metabolic process
	GO:0006073	Cellular glucan metabolic process
	GO:0016762	Xyloglucan:xyloglucosyl transferase activity
	GO:0048046	Apoplast
Phvul.003G152300	GO:0003854	3-β-hydroxy-Δ5-steroid dehydrogenase activity
	GO:0006694	Steroid biosynthetic process
	GO:0008831	dTDP-4-dehydrorhamnose reductase activity
	GO:0009058	Biosynthetic process
	GO:0016491	Oxidoreductase activity
	GO:0044237	Cellular metabolic process
	GO:0045226	Extracellular polysaccharide biosynthetic process
	GO:0050662	Coenzyme binding
	GO:0055114	Oxidation-reduction process
Phvul.004G022700	GO:0016757	Transferase activity, transferring glycosyl groups

Table S52 continued

Gene ID	GO term	Description
Phvul.004G047900	GO:0004657	Proline dehydrogenase activity
	GO:0006537	Glutamate biosynthetic process
	GO:0006562	Proline catabolic process
	GO:0055114	Oxidation-reduction process
Phvul.004G083200	GO:0008080	N-Acetyltransferase activity
	GO:0008152	Metabolic process
Phvul.005G039200	GO:0004553	Hydrolase activity, hydrolyzing *O*-glycosyl compounds
	GO:0005975	Carbohydrate metabolic process
Phvul.005G111300	GO:0004553	Hydrolase activity, hydrolyzing *O*-glycosyl compounds
	GO:0005618	Cell wall
	GO:0005975	Carbohydrate metabolic process
	GO:0006073	Cellular glucan metabolic process
	GO:0016762	Xyloglucan:xyloglucosyl transferase activity
	GO:0048046	Apoplast
Phvul.006G064100	GO:0004645	1,4-α-Oligoglucan phosphorylase activity
	GO:0005975	Carbohydrate metabolic process
Phvul.006G065300	GO:0009228	Thiamine biosynthetic process
Phvul.006G069300	GO:0004066	Asparagine synthase (glutamine-hydrolyzing) activity
	GO:0006529	Asparagine biosynthetic process
Phvul.006G151300	GO:0004553	Hydrolase activity, hydrolyzing *O*-glycosyl compounds
	GO:0005975	Carbohydrate metabolic process
Phvul.006G151400	GO:0004553	Hydrolase activity, hydrolyzing *O*-glycosyl compounds
	GO:0005975	Carbohydrate metabolic process
Phvul.008G103900	GO:0008152	Metabolic process
	GO:0016758	Transferase activity, transferring hexosyl groups
Phvul.008G237500	GO:0004356	Glutamate-ammonia ligase activity
	GO:0006542	Glutamine biosynthetic process
	GO:0006807	Nitrogen compound metabolic process
Phvul.008G279800	GO:0016020	Membrane
	GO:0016760	Cellulose synthase (UDP-forming) activity
	GO:0030244	Cellulose biosynthetic process
Phvul.010G042200	GO:0008152	Metabolic process
	GO:0016758	Transferase activity, transferring hexosyl groups
Phvul.011G107000	GO:0004553	Hydrolase activity, hydrolyzing *O*-glycosyl compounds
	GO:0005618	Cell wall
	GO:0005975	Carbohydrate metabolic process
	GO:0006073	Cellular glucan metabolic process
	GO:0016762	Xyloglucan:xyloglucosyl transferase activity
	GO:0048046	Apoplast

Table S52 continued

Gene ID	GO term	Description
Phvul.011G138600	GO:0008152	Metabolic process
	GO:0016758	Transferase activity, transferring hexosyl groups

Table S53. Gene ontology (GO) terms enriched across the differentially expressed genes of the CBB-resistant RIL at 0 h post-inoculation with *Xanthomonas axonopodis* (*Xap*)

Gene ID	GO term	Description
Phvul.001G226300	GO:0031072	Heat shock protein binding
Phvul.002G077400	GO:0016757	Transferase activity, transferring glycosyl groups
Phvul.002G093000	GO:0031072	Heat shock protein binding
Phvul.002G096000	GO:0005975	Carbohydrate metabolic process
	GO:0016868	Intramolecular transferase activity, phosphotransferases
Phvul.002G168400	GO:0004645	1,4-α-Oligoglucan phosphorylase activity
	GO:0005975	Carbohydrate metabolic process
Phvul.003G004300	GO:0031072	Heat shock protein binding
Phvul.003G047100	GO:0008152	Metabolic process
	GO:0016758	Transferase activity, transferring hexosyl groups
Phvul.003G052400	GO:0004553	Hydrolase activity, hydrolyzing *O*-glycosyl compounds
	GO:0005618	Cell wall
	GO:0005975	Carbohydrate metabolic process
	GO:0006073	Cellular glucan metabolic process
	GO:0016762	Xyloglucan:xyloglucosyl transferase activity
	GO:0048046	Apoplast
Phvul.003G137600	GO:0004553	Hydrolase activity, hydrolyzing *O*-glycosyl compounds
	GO:0005618	Cell wall
	GO:0005975	Carbohydrate metabolic process
	GO:0006073	Cellular glucan metabolic process
	GO:0016762	Xyloglucan:xyloglucosyl transferase activity
	GO:0048046	Apoplast
Phvul.003G147400	GO:0004553	Hydrolase activity, hydrolyzing *O*-glycosyl compounds
	GO:0005618	Cell wall
	GO:0005975	Carbohydrate metabolic process
	GO:0006073	Cellular glucan metabolic process
	GO:0016762	Xyloglucan:xyloglucosyl transferase activity
	GO:0048046	Apoplast
Phvul.003G147500	GO:0004553	Hydrolase activity, hydrolyzing *O*-glycosyl compounds
	GO:0005618	Cell wall
	GO:0005975	Carbohydrate metabolic process
	GO:0006073	Cellular glucan metabolic process
	GO:0016762	Xyloglucan:xyloglucosyl transferase activity
	GO:0048046	Apoplast
Phvul.003G147600	GO:0004553	Hydrolase activity, hydrolyzing *O*-glycosyl compounds
	GO:0005618	Cell wall
	GO:0005975	Carbohydrate metabolic process

Table S53 continued

Gene ID	GO term	Description
	GO:0006073	Cellular glucan metabolic process
	GO:0016762	Xyloglucan:xyloglucosyl transferase activity
	GO:0048046	Apoplast
Phvul.003G147700	GO:0004553	Hydrolase activity, hydrolyzing *O*-glycosyl compounds
	GO:0005618	Cell wall
	GO:0005975	Carbohydrate metabolic process
	GO:0006073	Cellular glucan metabolic process
	GO:0016762	Xyloglucan:xyloglucosyl transferase activity
	GO:0048046	Apoplast
Phvul.004G129600	GO:0004866	Endopeptidase inhibitor activity
Phvul.004G129700	GO:0004866	Endopeptidase inhibitor activity
Phvul.004G129800	GO:0004866	Endopeptidase inhibitor activity
Phvul.004G129900	GO:0004866	Endopeptidase inhibitor activity
Phvul.004G130000	GO:0004866	Endopeptidase inhibitor activity
Phvul.004G137600	GO:0004866	Endopeptidase inhibitor activity
Phvul.004G138500	GO:0004866	Endopeptidase inhibitor activity
Phvul.005G001000	GO:0016020	Membrane
	GO:0016760	Cellulose synthase (UDP-forming) activity
	GO:0030244	Cellulose biosynthetic process
Phvul.005G156800	GO:0005515	Protein binding
	GO:0016702	Oxidoreductase activity, acting on single donors with incorporation of molecular oxygen, incorporation of two atoms of oxygen
	GO:0046872	Metal ion binding
	GO:0055114	Oxidation-reduction process
Phvul.005G156900	GO:0005515	Protein binding
	GO:0016702	Oxidoreductase activity, acting on single donors with incorporation of molecular oxygen, incorporation of two atoms of oxygen
	GO:0046872	Metal ion binding
	GO:0055114	Oxidation-reduction process
Phvul.006G034000	GO:0003824	Catalytic activity
	GO:0003854	3-β-hydroxy-Δ5-steroid dehydrogenase activity
	GO:0006694	Steroid biosynthetic process
	GO:0008831	dTDP-4-dehydrorhamnose reductase activity
	GO:0009058	Biosynthetic process
	GO:0044237	Cellular metabolic process
	GO:0045226	Extracellular polysaccharide biosynthetic process

Table S53 continued

Gene ID	GO term	Description
	GO:0050662	Coenzyme binding
	GO:0055114	Oxidation-reduction process
Phvul.006G064100	GO:0004645	1,4-α-Oligoglucan phosphorylase activity
	GO:0005975	Carbohydrate metabolic process
Phvul.006G151300	GO:0004553	Hydrolase activity, hydrolyzing *O*-glycosyl compounds
	GO:0005975	Carbohydrate metabolic process
Phvul.006G151400	GO:0004553	Hydrolase activity, hydrolyzing *O*-glycosyl compounds
	GO:0005975	Carbohydrate metabolic process
Phvul.006G207600	GO:0031072	Heat shock protein binding
Phvul.007G135800	GO:0008152	Metabolic process
	GO:0016758	Transferase activity, transferring hexosyl groups
Phvul.009G002200	GO:0004044	Amidophosphoribosyltransferase activity
	GO:0008152	Metabolic process
	GO:0009113	Purine nucleobase biosynthetic process
	GO:0009116	Nucleoside metabolic process
Phvul.010G134900	GO:0005515	Protein binding
	GO:0016702	Oxidoreductase activity, acting on single donors with incorporation of molecular oxygen, incorporation of two atoms of oxygen
	GO:0046872	Metal ion binding
	GO:0055114	Oxidation-reduction process
Phvul.010G135000	GO:0005515	Protein binding
	GO:0016702	Oxidoreductase activity, acting on single donors with incorporation of molecular oxygen, incorporation of two atoms of oxygen
	GO:0046872	Metal ion binding
	GO:0055114	Oxidation-reduction process
Phvul.010G162600	GO:0031072	Heat shock protein binding
Phvul.011G056500	GO:0005515	Protein binding
	GO:0016702	Oxidoreductase activity, acting on single donors with incorporation of molecular oxygen, incorporation of two atoms of oxygen
	GO:0046872	Metal ion binding
	GO:0055114	Oxidation-reduction process
Phvul.011G085200	GO:0004553	Hydrolase activity, hydrolyzing *O*-glycosyl compounds
	GO:0005618	Cell wall
	GO:0005975	Carbohydrate metabolic process
	GO:0006073	Cellular glucan metabolic process
	GO:0016762	Xyloglucan:xyloglucosyl transferase activity

Table S53 continued

Gene ID	GO term	Description
	GO:0048046	Apoplast
Phvul.011G138600	GO:0008152	Metabolic process
	GO:0016758	Transferase activity, transferring hexosyl groups
Phvul.011G167000	GO:0004553	Hydrolase activity, hydrolyzing *O*-glycosyl compounds
	GO:0005975	Carbohydrate metabolic process

Table S54. Gene ontology (GO) terms enriched across the differentially expressed genes of the CBB-resistant RIL at 24 h post-inoculation with *Xanthomonas axonopodis* (*Xap*)

Gene ID	GO term	Description
Phvul.001G203300	GO:0009055	Electron transfer activity
	GO:0015035	Protein disulfide oxidoreductase activity
	GO:0045454	Cell redox homeostasis
Phvul.002G275000	GO:0003677	DNA binding
	GO:0006355	Regulation of transcription, DNA-templated
Phvul.002G297100	GO:0003700	DNA-binding transcription factor activity
	GO:0006355	Regulation of transcription, DNA-templated
	GO:0043565	Sequence-specific DNA binding
Phvul.003G212700	GO:0003700	DNA-binding transcription factor activity
	GO:0006355	Regulation of transcription, DNA-templated
Phvul.003G292400	GO:0003700	DNA-binding transcription factor activity
	GO:0006355	Regulation of transcription, DNA-templated
Phvul.006G111700	GO:0003700	DNA-binding transcription factor activity
	GO:0006355	Regulation of transcription, DNA-templated
	GO:0043565	Sequence-specific DNA binding
Phvul.006G183100	GO:0003700	DNA-binding transcription factor activity
Phvul.007G066500	GO:0003700	DNA-binding transcription factor activity
	GO:0006355	Regulation of transcription, DNA-templated
Phvul.007G273400	GO:0003677	DNA binding
Phvul.008G235100	GO:0005509	Calcium ion binding
	GO:0005578	Extracellular matrix
	GO:0007165	Signal transduction
Phvul.009G089300	GO:0003700	DNA-binding transcription factor activity
	GO:0006355	Regulation of transcription, DNA-templated
Phvul.010G062500	GO:0003700	DNA-binding transcription factor activity
	GO:0006355	Regulation of transcription, DNA-templated
	GO:0043565	Sequence-specific DNA binding
Phvul.010G111900	GO:0003700	DNA-binding transcription factor activity
	GO:0006355	Regulation of transcription, DNA-templated
	GO:0043565	Sequence-specific DNA binding
Phvul.010G120700	GO:0003677	DNA binding
	GO:0006355	Regulation of transcription, DNA-templated
Phvul.011G095500	GO:0003677	DNA binding
	GO:0006355	Regulation of transcription, DNA-templated

Table S55. Gene ontology (GO) terms enriched across the differentially expressed genes of the CBB-resistant RIL at 48 h post-inoculation with *Xanthomonas axonopodis* (*Xap*)

Gene ID	GO term	Description
Phvul.001G039900	GO:0003700	DNA-binding transcription factor activity
	GO:0006355	Regulation of transcription, DNA-templated
	GO:0043565	Sequence-specific DNA binding
Phvul.001G040000	GO:0004672	Protein kinase activity
	GO:0005488	Binding
	GO:0005524	ATP binding
	GO:0006468	Protein phosphorylation
Phvul.001G040300	GO:0004672	Protein kinase activity
	GO:0005488	Binding
	GO:0005524	ATP binding
	GO:0006468	Protein phosphorylation
Phvul.001G040700	GO:0004672	Protein kinase activity
	GO:0005488	Binding
	GO:0005524	ATP binding
	GO:0006468	Protein phosphorylation
Phvul.001G042100	GO:0003700	DNA-binding transcription factor activity
	GO:0006355	Regulation of transcription, DNA-templated
	GO:0043565	Sequence-specific DNA binding
Phvul.001G042200	GO:0003700	DNA-binding transcription factor activity
	GO:0006355	Regulation of transcription, DNA-templated
	GO:0043565	Sequence-specific DNA binding
Phvul.001G088200	GO:0003700	DNA-binding transcription factor activity
	GO:0006355	Regulation of transcription, DNA-templated
	GO:0043565	Sequence-specific DNA binding
Phvul.001G112400	GO:0016747	Transferase activity, transferring acyl groups other than amino-acyl groups
Phvul.001G131000	GO:0003700	DNA-binding transcription factor activity
	GO:0005634	Nucleus
	GO:0006355	Regulation of transcription, DNA-templated
	GO:0043565	Sequence-specific DNA binding
Phvul.001G142000	GO:0009790	Embryo development
Phvul.001G160100	GO:0003700	DNA-binding transcription factor activity
	GO:0006355	Regulation of transcription, DNA-templated
Phvul.001G164900	GO:0005634	Nucleus
	GO:0006355	Regulation of transcription, DNA-templated
Phvul.001G192000	GO:0003677	DNA binding
	GO:0006355	Regulation of transcription, DNA-templated

Table S55 continued

Gene ID	GO term	Description
Phvul.002G014700	GO:0009055	Electron transfer activity
	GO:0016705	Oxidoreductase activity, acting on paired donors, with incorporation or reduction of molecular oxygen
	GO:0020037	Heme binding
	GO:0055114	Oxidation-reduction process
Phvul.002G019100	GO:0003700	DNA-binding transcription factor activity
	GO:0005634	Nucleus
	GO:0006355	Regulation of transcription, DNA-templated
	GO:0043565	Sequence-specific DNA binding
Phvul.002G025000	GO:0009055	Electron transfer activity
	GO:0016705	Oxidoreductase activity, acting on paired donors, with incorporation or reduction of molecular oxygen
	GO:0020037	Heme binding
	GO:0055114	Oxidation-reduction process
Phvul.002G038600	GO:0004315	3-Oxoacyl-[acyl-carrier-protein] synthase activity
	GO:0006633	Fatty acid biosynthetic process
	GO:0008415	Transferase activity, transferring acyl groups
	GO:0016020	Membrane
Phvul.002G038700	GO:0004315	3-Oxoacyl-[acyl-carrier-protein] synthase activity
	GO:0006633	Fatty acid biosynthetic process
	GO:0008415	Transferase activity, transferring acyl groups
	GO:0016020	Membrane
Phvul.002G038800	GO:0008415	Transferase activity, transferring acyl groups
	GO:0008610	Lipid biosynthetic process
Phvul.002G038900	GO:0004315	3-Oxoacyl-[acyl-carrier-protein] synthase activity
	GO:0006633	Fatty acid biosynthetic process
	GO:0008415	Transferase activity, transferring acyl groups
	GO:0016020	Membrane
Phvul.002G039000	GO:0008415	Transferase activity, transferring acyl groups
	GO:0008610	Lipid biosynthetic process
Phvul.002G039100	GO:0004315	3-Oxoacyl-[acyl-carrier-protein] synthase activity
	GO:0006633	Fatty acid biosynthetic process
	GO:0008415	Transferase activity, transferring acyl groups
	GO:0016020	Membrane
Phvul.002G039300	GO:0004315	3-Oxoacyl-[acyl-carrier-protein] synthase activity
	GO:0006633	Fatty acid biosynthetic process
	GO:0008415	Transferase activity, transferring acyl groups
	GO:0016020	Membrane

Table S55 continued

Gene ID	GO term	Description
Phvul.002G044100	GO:0008080	N-Acetyltransferase activity
	GO:0008152	Metabolic process
Phvul.002G046100	GO:0004672	Protein kinase activity
	GO:0005524	ATP binding
	GO:0005529	Carbohydrate binding
	GO:0006468	Protein phosphorylation
Phvul.002G046500	GO:0004672	Protein kinase activity
	GO:0005524	ATP binding
	GO:0005529	Carbohydrate binding
	GO:0006468	Protein phosphorylation
Phvul.002G076500	GO:0004222	Metalloendopeptidase activity
	GO:0006508	Proteolysis
	GO:0008152	Metabolic process
	GO:0008270	Zinc ion binding
Phvul.002G076600	GO:0004222	Metalloendopeptidase activity
	GO:0006508	Proteolysis
	GO:0008152	Metabolic process
	GO:0008270	Zinc ion binding
Phvul.002G144600	GO:0008270	Zinc ion binding
	GO:0016616	Oxidoreductase activity, acting on the CH-OH group of donors, NAD or NADP as acceptor
	GO:0048037	Cofactor binding
	GO:0055114	Oxidation-reduction process
Phvul.002G154600	GO:0006855	Drug transmembrane transport
	GO:0015238	Xenobiotic transmembrane transporter activity
	GO:0015297	Antiporter activity
	GO:0016020	Membrane
Phvul.002G184300	GO:0004315	3-Oxoacyl-[acyl-carrier-protein] synthase activity
	GO:0006633	Fatty acid biosynthetic process
	GO:0008415	Transferase activity, transferring acyl groups
	GO:0016020	Membrane
Phvul.002G199800	GO:0008762	UDP-N-acetylmuramate dehydrogenase activity
	GO:0016491	Oxidoreductase activity
	GO:0050660	Flavin adenine dinucleotide binding
	GO:0055114	Oxidation-reduction process
Phvul.002G200600	GO:0008762	UDP-N-acetylmuramate dehydrogenase activity
	GO:0016491	Oxidoreductase activity
	GO:0050660	Flavin adenine dinucleotide binding
	GO:0055114	Oxidation-reduction process

Table S55 continued

Gene ID	GO term	Description
Phvul.002G209400	GO:0006952	Defense response
	GO:0009607	Response to biotic stimulus
Phvul.002G209500	GO:0006952	Defense response
	GO:0009607	Response to biotic stimulus
Phvul.002G215000	GO:0004672	Protein kinase activity
	GO:0005488	Binding
	GO:0005524	ATP binding
	GO:0006468	Protein phosphorylation
Phvul.002G215100	GO:0004672	Protein kinase activity
	GO:0005515	Protein binding
	GO:0005524	ATP binding
	GO:0006468	Protein phosphorylation
Phvul.002G217900	GO:0004672	Protein kinase activity
	GO:0005515	Protein binding
	GO:0005524	ATP binding
	GO:0006468	Protein phosphorylation
Phvul.002G219300	GO:0000287	Magnesium ion binding
	GO:0008152	Metabolic process
	GO:0010333	Terpene synthase activity
	GO:0016829	Lyase activity
Phvul.002G223400	GO:0016491	Oxidoreductase activity
	GO:0050660	Flavin adenine dinucleotide binding
	GO:0055114	Oxidation-reduction process
	GO:0004672	Protein kinase activity
	GO:0005515	Protein binding
	GO:0005524	ATP binding
	GO:0006468	Protein phosphorylation
Phvul.002G265400	GO:0003700	DNA-binding transcription factor activity
	GO:0006355	Regulation of transcription, DNA-templated
	GO:0043565	Sequence-specific DNA binding
Phvul.002G285800	GO:0003700	DNA-binding transcription factor activity
	GO:0006355	Regulation of transcription, DNA-templated
	GO:0043565	Sequence-specific DNA binding
Phvul.002G318200	GO:0004672	Protein kinase activity
	GO:0005524	ATP binding
	GO:0006468	Protein phosphorylation
Phvul.002G326600	GO:0016491	Oxidoreductase activity
	GO:0016706	2-Oxoglutarate-dependent dioxygenase activity
	GO:0055114	Oxidation-reduction process

Table S55 continued

Gene ID	GO term	Description
Phvul.003G009200	GO:0009055	Electron transfer activity
	GO:0016705	Oxidoreductase activity, acting on paired donors, with incorporation or reduction of molecular oxygen
	GO:0020037	Heme binding
	GO:0055114	Oxidation-reduction process
Phvul.003G022400	GO:0030001	Metal ion transport
	GO:0046872	Metal ion binding
Phvul.003G024200	GO:0000166	Nucleotide binding
	GO:0003824	Catalytic activity
	GO:0008152	Metabolic process
	GO:0046872	Metal ion binding
Phvul.003G051700	GO:0009055	Electron transfer activity
	GO:0016705	Oxidoreductase activity, acting on paired donors, with incorporation or reduction of molecular oxygen
	GO:0020037	Heme binding
	GO:0055114	Oxidation-reduction process
Phvul.003G051900	GO:0005507	Copper ion binding
Phvul.003G074000	GO:0009055	Electron transfer activity
	GO:0009055	Electron transfer activity
	GO:0016705	Oxidoreductase activity, acting on paired donors, with incorporation or reduction of molecular oxygen
	GO:0020037	Heme binding
	GO:0055114	Oxidation-reduction process
Phvul.003G088000	GO:0006950	Response to stress
Phvul.003G096700	GO:0006950	Response to stress
Phvul.003G109000	GO:0006952	Defense response
	GO:0009607	Response to biotic stimulus
Phvul.003G109100	GO:0006952	Defense response
	GO:0009607	Response to biotic stimulus
Phvul.003G109200	GO:0006952	Defense response
	GO:0009607	Response to biotic stimulus
Phvul.003G109300	GO:0006952	Defense response
	GO:0009607	Response to biotic stimulus
Phvul.003G109600	GO:0006952	Defense response
	GO:0009607	Response to biotic stimulus
Phvul.003G109800	GO:0006952	Defense response
	GO:0009607	Response to biotic stimulus
Phvul.003G126300	GO:0016491	Oxidoreductase activity
	GO:0055114	Oxidation-reduction process

Table S55 continued

Gene ID	GO term	Description
Phvul.003G131500	GO:0010181	FMN binding
	GO:0016491	Oxidoreductase activity
	GO:0055114	Oxidation-reduction process
Phvul.003G136400	GO:0008762	UDP-N-acetylmuramate dehydrogenase activity
	GO:0009690	Cytokinin metabolic process
	GO:0016491	Oxidoreductase activity
	GO:0050660	Flavin adenine dinucleotide binding
Phvul.003G140800	GO:0006355	Regulation of transcription, DNA-templated
	GO:0030528	Transcription regulator activity
Phvul.003G154800	GO:0000902	Cell morphogenesis
	GO:0005524	ATP binding
Phvul.003G166700	GO:0005507	Copper ion binding
	GO:0009055	Electron transfer activity
Phvul.003G166800	GO:0005507	Copper ion binding
	GO:0009055	Electron transfer activity
Phvul.003G182600	GO:0006950	Response to stress
Phvul.003G187200	GO:0004672	Protein kinase activity
	GO:0005515	Protein binding
	GO:0005524	ATP binding
	GO:0006468	Protein phosphorylation
Phvul.003G212600	GO:0006855	Drug transmembrane transport
	GO:0015238	Xenobiotic transmembrane transporter activity
	GO:0015297	Antiporter activity
	GO:0016020	Membrane
Phvul.003G272900	GO:0003824	Catalytic activity
	GO:0016616	Oxidoreductase activity, acting on the CH-OH group of donors, NAD or NADP as acceptor
	GO:0044237	Cellular metabolic process
	GO:0050662	Coenzyme binding
Phvul.003G287400	GO:0008270	Zinc ion binding
	GO:0016491	Oxidoreductase activity
	GO:0055114	Oxidation-reduction process
Phvul.003G287500	GO:0008270	Zinc ion binding
	GO:0016491	Oxidoreductase activity
	GO:0055114	Oxidation-reduction process
Phvul.004G018900	GO:0004601	Peroxidase activity
	GO:0006979	Response to oxidative stress
	GO:0020037	Heme binding
	GO:0055114	Oxidation-reduction process

Table S55 continued

Gene ID	GO term	Description
Phvul.004G077400	GO:0003677	DNA binding
	GO:0006355	Regulation of transcription, DNA-templated
Phvul.004G092100	GO:0003700	DNA-binding transcription factor activity
	GO:0006355	Regulation of transcription, DNA-templated
Phvul.004G122000	GO:0003700	DNA-binding transcription factor activity
	GO:0006355	Regulation of transcription, DNA-templated
Phvul.004G141200	GO:0008060	GTPase activator activity
	GO:0008270	Zinc ion binding
	GO:0032312	Transcription regulator activity
Phvul.005G011100	GO:0000166	Nucleotide binding
	GO:0003824	Catalytic activity
	GO:0008152	Metabolic process
	GO:0046872	Metal ion binding
Phvul.005G108900	GO:0016747	Transferase activity, transferring acyl groups other than amino-acyl groups
Phvul.005G109000	GO:0016747	Transferase activity, transferring acyl groups other than amino-acyl groups
Phvul.005G111500	GO:0000287	Magnesium ion binding
	GO:0008152	Metabolic process
	GO:0010333	Terpene synthase activity
	GO:0016829	Lyase activity
Phvul.005G113800	GO:0004601	Peroxidase activity
	GO:0006979	Response to oxidative stress
	GO:0020037	Heme binding
	GO:0055114	Oxidation-reduction process
Phvul.005G155800	GO:0004568	Chitinase activity
	GO:0006032	Chitin catabolic process
	GO:0008061	Chitin binding
	GO:0016998	Cell wall macromolecule catabolic process
Phvul.005G173000	GO:0005634	Nucleus
	GO:0006355	Regulation of transcription, DNA-templated
Phvul.006G020700	GO:0004672	Protein kinase activity
	GO:0005524	ATP binding
	GO:0006468	Protein phosphorylation
Phvul.006G074600	GO:0003700	DNA-binding transcription factor activity
	GO:0006355	Regulation of transcription, DNA-templated
	GO:0043565	Sequence-specific DNA binding

Table S55 continued

Gene ID	GO term	Description
Phvul.006G079700	GO:0009055	Electron transfer activity
	GO:0016705	Oxidoreductase activity, acting on paired donors, with incorporation or reduction of molecular oxygen
	GO:0020037	Heme binding
	GO:0055114	Oxidation-reduction process
Phvul.006G090200	GO:0005506	Iron ion binding
	GO:0016021	Integral component of membrane
	GO:0050660	Flavin adenine dinucleotide binding
	GO:0050664	Oxidoreductase activity, acting on NAD(P)H, oxygen as acceptor
Phvul.006G102200	GO:0008061	Chitin binding
	GO:0042742	Defense response to bacterium
	GO:0050832	Defense response to fungus
Phvul.006G102300	GO:0042742	Defense response to bacterium
	GO:0050832	Defense response to fungus
Phvul.006G124600	GO:0008762	UDP-N-acetylmuramate dehydrogenase activity
	GO:0016491	Oxidoreductase activity
	GO:0050660	Flavin adenine dinucleotide binding
	GO:0055114	Oxidation-reduction process
Phvul.006G124700	GO:0008762	UDP-N-acetylmuramate dehydrogenase activity
	GO:0016491	Oxidoreductase activity
	GO:0050660	Flavin adenine dinucleotide binding
	GO:0055114	Oxidation-reduction process
Phvul.006G129500	GO:0004601	Peroxidase activity
	GO:0006979	Response to oxidative stress
	GO:0020037	Heme binding
	GO:0055114	Oxidation-reduction process
Phvul.006G135600	GO:0030001	Metal ion transport
	GO:0046872	Metal ion binding
Phvul.006G181300	GO:0009055	Electron transfer activity
	GO:0015035	Protein disulfide oxidoreductase activity
	GO:0045454	Cell redox homeostasis
	GO:0016702	Oxidoreductase activity, acting on single donors with incorporation of molecular oxygen, incorporation of two atoms of oxygen
	GO:0046872	Metal ion binding
	GO:0055114	Oxidation-reduction process
Phvul.006G188900	GO:0003677	DNA binding
	GO:0006355	Regulation of transcription, DNA-templated

Table S55 continued

Gene ID	GO term	Description
Phvul.006G192400	GO:0016491	Oxidoreductase activity
	GO:0050660	Flavin adenine dinucleotide binding
	GO:0055114	Oxidation-reduction process
Phvul.006G194600	GO:0004674	Protein serine/threonine kinase activity
	GO:0005524	ATP binding
	GO:0005529	Carbohydrate binding
	GO:0006468	Protein phosphorylation
Phvul.006G195600	GO:0000287	Magnesium ion binding
	GO:0008152	Metabolic process
	GO:0010333	Terpene synthase activity
	GO:0016829	Lyase activity
Phvul.006G195700	GO:0000287	Magnesium ion binding
	GO:0008152	Metabolic process
	GO:0010333	Terpene synthase activity
	GO:0016829	Lyase activity
Phvul.006G198200	GO:0004672	Protein kinase activity
	GO:0005515	Protein binding
	GO:0005524	ATP binding
	GO:0006468	Protein phosphorylation
Phvul.007G048500	GO:0004672	Protein kinase activity
	GO:0005524	ATP binding
	GO:0006468	Protein phosphorylation
Phvul.007G048600	GO:0004672	Protein kinase activity
	GO:0005524	ATP binding
	GO:0006468	Protein phosphorylation
Phvul.007G048800	GO:0004672	Protein kinase activity
	GO:0005524	ATP binding
	GO:0006468	Protein phosphorylation
Phvul.007G048900	GO:0004672	Protein kinase activity
	GO:0005524	ATP binding
	GO:0006468	Protein phosphorylation
Phvul.007G049100	GO:0004672	Protein kinase activity
	GO:0005524	ATP binding
	GO:0006468	Protein phosphorylation
Phvul.007G052500	GO:0004672	Protein kinase activity
	GO:0005524	ATP binding
	GO:0006468	Protein phosphorylation

Table S55 continued

Gene ID	GO term	Description
Phvul.007G222500	GO:0003700	DNA-binding transcription factor activity
	GO:0006355	Regulation of transcription, DNA-templated
	GO:0004672	Protein kinase activity
	GO:0005524	ATP binding
	GO:0006468	Protein phosphorylation
Phvul.007G259400	GO:0009790	Embryo development
Phvul.007G260400	GO:0004672	Protein kinase activity
	GO:0005488	Binding
	GO:0005524	ATP binding
	GO:0006468	Protein phosphorylation
Phvul.007G273000	GO:0003700	DNA-binding transcription factor activity
	GO:0006355	Regulation of transcription, DNA-templated
Phvul.007G278900	GO:0005509	Calcium ion binding
	GO:0005578	Carbohydrate binding
	GO:0007165	Signal transduction
Phvul.008G011400	GO:0000902	Cell morphogenesis
	GO:0005524	ATP binding
Phvul.008G011500	GO:0047134	Protein-disulfide reductase activity
	GO:0055114	Oxidation-reduction process
Phvul.008G011800	GO:0047134	Protein-disulfide reductase activity
	GO:0055114	Oxidation-reduction process
Phvul.008G011900	GO:0000902	Cell morphogenesis
	GO:0005524	ATP binding
Phvul.008G015800	GO:0016491	Oxidoreductase activity
	GO:0055114	Oxidation-reduction process
Phvul.008G032200	GO:0016747	Transferase activity, transferring acyl groups other than amino-acyl groups
Phvul.008G037300	GO:0005509	Calcium ion binding
	GO:0005578	Carbohydrate binding
	GO:0007165	Signal transduction
Phvul.008G040800	GO:0004222	Metalloendopeptidase activity
	GO:0006508	Proteolysis
	GO:0008270	Zinc ion binding
	GO:0016021	Integral component of membrane
Phvul.008G076300	GO:0005506	Iron ion binding
	GO:0006633	Fatty acid biosynthetic process
	GO:0016491	Oxidoreductase activity
	GO:0055114	Oxidation-reduction process

Table S55 continued

Gene ID	GO term	Description
Phvul.008G076500	GO:0006694	Steroid biosynthetic process
	GO:0016616	Oxidoreductase activity, acting on the CH-OH group of donors, NAD or NADP as acceptor
	GO:0044237	Cellular metabolic process
	GO:0050662	Coenzyme binding
Phvul.008G076600	GO:0006694	Steroid biosynthetic process
	GO:0016616	Oxidoreductase activity, acting on the CH-OH group of donors, NAD or NADP as acceptor
	GO:0044237	Cellular metabolic process
	GO:0050662	Coenzyme binding
Phvul.008G094500	GO:0004672	Protein kinase activity
	GO:0005488	Binding
	GO:0005524	ATP binding
	GO:0006468	Protein phosphorylation
Phvul.008G098500	GO:0016491	Oxidoreductase activity
	GO:0016706	2-Oxoglutarate-dependent dioxygenase activity
	GO:0055114	Oxidation-reduction process
Phvul.008G112200	GO:0005507	Copper ion binding
	GO:0016491	Oxidoreductase activity
	GO:0055114	Oxidation-reduction process
Phvul.008G127200	GO:0016491	Oxidoreductase activity
	GO:0016706	2-Oxoglutarate-dependent dioxygenase activity
	GO:0055114	Oxidation-reduction process
Phvul.008G194600	GO:0003677	DNA binding
	GO:0006355	Regulation of transcription, DNA-templated
Phvul.008G218500	GO:0004601	Peroxidase activity
	GO:0006979	Response to oxidative stress
	GO:0020037	Heme binding
	GO:0055114	Oxidation-reduction process
Phvul.008G223500	GO:0016491	Oxidoreductase activity
	GO:0016706	2-Oxoglutarate-dependent dioxygenase activity
	GO:0055114	Oxidation-reduction process
Phvul.008G248900	GO:0004672	Protein kinase activity
	GO:0005524	ATP binding
	GO:0006468	Protein phosphorylation
	GO:0007165	Signal transduction
Phvul.008G249900	GO:0004601	Peroxidase activity
	GO:0006979	Response to oxidative stress
	GO:0020037	Heme binding
	GO:0055114	Oxidation-reduction process

Table S55 Continued

Gene ID	GO term	Description
Phvul.008G285000	GO:0000166	Nucleotide binding
	GO:0046872	Metal ion binding
Phvul.008G287200	GO:0016491	Oxidoreductase activity
	GO:0055114	Oxidation-reduction process
Phvul.008G287300	GO:0016491	Oxidoreductase activity
	GO:0055114	Oxidation-reduction process
Phvul.009G043100	GO:0003700	DNA-binding transcription factor activity
	GO:0006355	Regulation of transcription, DNA-templated
	GO:0043565	Sequence-specific DNA binding
Phvul.009G043200	GO:0003700	DNA-binding transcription factor activity
	GO:0006355	Regulation of transcription, DNA-templated
	GO:0043565	Sequence-specific DNA binding
Phvul.009G046900	GO:0004672	Protein kinase activity
	GO:0005515	Protein binding
	GO:0005524	ATP binding
	GO:0006468	Protein phosphorylation
Phvul.009G080000	GO:0003700	DNA-binding transcription factor activity
	GO:0006355	Regulation of transcription, DNA-templated
	GO:0043565	Sequence-specific DNA binding
Phvul.009G087400	GO:0003700	DNA-binding transcription factor activity
	GO:0006355	Regulation of transcription, DNA-templated
	GO:0043565	Sequence-specific DNA binding
Phvul.009G089300	GO:0003700	DNA-binding transcription factor activity
	GO:0006355	Regulation of transcription, DNA-templated
Phvul.009G138900	GO:0003700	DNA-binding transcription factor activity
	GO:0006355	Regulation of transcription, DNA-templated
	GO:0043565	Sequence-specific DNA binding
Phvul.009G156300	GO:0003677	DNA binding
	GO:0006355	Regulation of transcription, DNA-templated
Phvul.009G182300	GO:0016491	Oxidoreductase activity
	GO:0055114	Oxidation-reduction process
Phvul.009G231600	GO:0006355	Regulation of transcription, DNA-templated
	GO:0030528	Transcription regulator activity
Phvul.009G235700	GO:0006355	Regulation of transcription, DNA-templated
	GO:0030528	Transcription regulator activity
Phvul.009G244100	GO:0009055	Electron transfer activity
	GO:0016705	Oxidoreductase activity, acting on paired donors, with incorporation or reduction of molecular oxygen

Table S55 continued

Gene ID	GO term	Description
Phvul.009G244100	GO:0020037	Heme binding
	GO:0055114	Oxidation-reduction process
Phvul.009G244200	GO:0009055	Electron transfer activity
	GO:0016705	Oxidoreductase activity, acting on paired donors, with incorporation or reduction of molecular oxygen
	GO:0020037	Heme binding
	GO:0055114	Oxidation-reduction process
Phvul.009G262900	GO:0005515	Protein binding
	GO:0016702	Oxidoreductase activity, acting on single donors with incorporation of molecular oxygen, incorporation of two atoms of oxygen
	GO:0046872	Metal ion binding
	GO:0055114	Oxidation-reduction process
Phvul.010G000300	GO:0016491	Oxidoreductase activity
	GO:0016706	2-Oxoglutarate-dependent dioxygenase activity
	GO:0055114	Oxidation-reduction process
Phvul.010G005900	GO:0005507	Copper ion binding
	GO:0016491	Oxidoreductase activity
	GO:0055114	Oxidation-reduction process
Phvul.010G032000	GO:0004672	Protein kinase activity
	GO:0005524	ATP binding
	GO:0005529	Carbohydrate binding
	GO:0006468	Protein phosphorylation
Phvul.010G057300	GO:0004672	Protein kinase activity
	GO:0005524	ATP binding
	GO:0005529	Carbohydrate binding
	GO:0006468	Protein phosphorylation
Phvul.010G057500	GO:0004672	Protein kinase activity
	GO:0005524	ATP binding
	GO:0005529	Carbohydrate binding
	GO:0006468	Protein phosphorylation
Phvul.010G057600	GO:0004672	Protein kinase activity
	GO:0005524	ATP binding
	GO:0005529	Carbohydrate binding
	GO:0006468	Protein phosphorylation
Phvul.010G111900	GO:0003700	DNA-binding transcription factor activity
	GO:0006355	Regulation of transcription, DNA-templated
	GO:0043565	Sequence-specific DNA binding

Table S55 continued

Gene ID	GO term	Description
Phvul.010G117200	GO:0003700	DNA-binding transcription factor activity
	GO:0005634	Nucleus
	GO:0006355	Regulation of transcription, DNA-templated
	GO:0043565	Sequence-specific DNA binding
Phvul.010G128900	GO:0009055	Electron transfer activity
	GO:0016705	Oxidoreductase activity, acting on paired donors, with incorporation or reduction of molecular oxygen
	GO:0020037	Heme binding
	GO:0055114	Oxidation-reduction process
Phvul.011G013600	GO:0000287	Magnesium ion binding
	GO:0008152	Metabolic process
	GO:0010333	Terpene synthase activity
	GO:0016829	Lyase activity
Phvul.011G035600	GO:0004672	Protein kinase activity
	GO:0005524	ATP binding
	GO:0006468	Protein phosphorylation
Phvul.011G041400	GO:0004672	Protein kinase activity
	GO:0005524	ATP binding
	GO:0005529	Carbohydrate binding
	GO:0006468	Protein phosphorylation
Phvul.011G044500	GO:0006694	Steroid biosynthetic process
	GO:0016616	Oxidoreductase activity, acting on the CH-OH group of donors, NAD or NADP as acceptor
	GO:0044237	Cellular metabolic process
	GO:0050662	Coenzyme binding
Phvul.011G147800	GO:0003677	DNA binding
	GO:0006355	Regulation of transcription, DNA-templated
Phvul.011G150400	GO:0004672	Protein kinase activity
	GO:0005524	ATP binding
	GO:0005529	Carbohydrate binding
	GO:0006468	Protein phosphorylation
Phvul.011G179600	GO:0008270	Zinc ion binding
Phvul.011G214400	GO:0004672	Protein kinase activity
	GO:0005524	ATP binding
	GO:0005529	Carbohydrate binding
	GO:0006468	Protein phosphorylation

Table S56. UHPLC-MS/MS identification of flavonols and isoflavones in acidified methanolic extracts of leaves sampled from CBB-resistant and CBB-susceptible *P. vulgaris* leaves.

Compound	Chemical Formula	Retention Time (min)	Parent Ion $[M-H]^-$	Fragment Ion(s) $[M-H]^-$
Quercetin rutinoside-xyloside (1)	$C_{32}H_{38}O_{20}$	22.46	741.1891	609.1483, 301.0355, 300.0278, 178.9989
Quercetin glucoside-xyloside (1)	$C_{26}H_{28}O_{16}$	22.7	595.1309	595.1312, 463.0536, 301.0357, 300.0279
Quercetin glucuronide-xyloside	$C_{26}H_{26}O_{17}$	22.86	609.11	301.0353, 300.0276, 178.9984, 151.0039
Quercetin glucoside-xyloside (2)	$C_{26}H_{28}O_{16}$	22.9	595.1309	595.1309, 463.0536, 301.0356, 300.0278
Kaempferol diglucoside (1)	$C_{27}H_{30}O_{16}$	23.42	609.1466	609.1465, 285.0399, 284.0324
Quercetin rutinoside-xyloside (2)	$C_{32}H_{38}O_{20}$	23.45	741.1891	741.1905, 301.0363, 300.0274
Kaempferol diglucoside (2)	$C_{27}H_{30}O_{16}$	23.68	609.1466	609.1465, 285.0399, 284.0324
Quercetin dixyloside (1)	$C_{25}H_{26}O_{15}$	24.67	565.1202	565.1201, 301.0354, 300.0275, 178.9984
Kaempferol rutinoside-xyloside	$C_{32}H_{38}O_{19}$	24.71	725.1945	725.1941, 593.1503, 285.0403, 284.0325
Quercetin 3-*O*-galactoside	$C_{21}H_{20}O_{12}$	24.74	463.0882	463.0875, 301.0349, 300.0273, 255.0286

Table S56 continued

Compound	Chemical Formula	Retention Time (min)	Parent Ion $[M-H]^-$	Fragment Ion(s) $[M-H]^-$
Quercetin 3-*O*-glucuronide	$C_{21}H_{18}O_{13}$	24.91	477.0674	301.0353, 178.9987, 151.0038
Quercetin 3-*O*-glucoside	$C_{21}H_{20}O_{12}$	24.97	463.0882	463.0880, 301.0352, 300.0274, 271.0250
Quercetin 3-*O*-rutinoside	$C_{27}H_{30}O_{16}$	25.01	609.1461	609.1465, 301.0352, 300.0274, 178.9977
Kaempferol glucoside-xyloside	$C_{32}H_{38}O_{19}$	25.24	579.1349	579.1351, 447.0923, 285.0403, 284.0326
Quercetin dixyloside (2)	$C_{25}H_{26}O_{15}$	25.28	565.1202	565.1194, 301.0350, 300.0275
Quercetin xyloside	$C_{20}H_{18}O_{11}$	25.59	433.0779	433.0747, 301.0352, 300.0276, 271.0612
Isorhamnetin glucoside-xyloside	$C_{27}H_{30}O_{16}$	25.63	609.1454	609.1470, 315.0512, 314.0432, 300.0276
Kaempferol glucuronide	$C_{21}H_{18}O_{12}$	27.34	461.0729	285.0403, 113.0247, 85.0596, 59.0136
Kaempferol rutinoside	$C_{27}H_{30}O_{15}$	27.53	593.1512	593.1514, 285.0404, 284.0325
Isorhamnetin glucuronide	$C_{22}H_{20}O_{13}$	28.16	491.0832	315.0512, 300.0275, 113.0248, 85.0297
Daidzein	$C_{15}H_{10}O_4$	28.6	253.0506	253.0510, 224.0482, 209.0612

Table S56 continued

Compound	Chemical Formula	Retention Time (min)	Parent Ion [M-H]⁻	Fragment Ion(s) [M-H]⁻
Genistein	$C_{15}H_{10}O_5$	31.59	269.0451	269.0457, 224.0475, 133.0293
Coumestrol[1]	$C_{15}H_8O_5$	33.86	267.0299	267.0299, 266.0220, 239.0351, 211.0403
Phaseollinisoflavan	$C_{20}H_{20}O_4$	38.2	323.1295	323.1292, 282.9808, 201.0924, 135.0454

[1] Identified via co-elution with an authentic coumestrol standard. All other compounds were identified on the basis of their MS parent and fragment ion data and compared to a MS/MS library for flavonols and isoflavones, and the scientific literature as described in section 4.5 of the Materials and Methods.

Figure S1. Negative ion UHPLC-MS/MS analysis of the leaves of the CBB-resistant RIL following *X. axonopodis* inoculation detected a peak (retention time = 38.2 min) with MS/MS fragmentation data matching phaseollinisoflavan

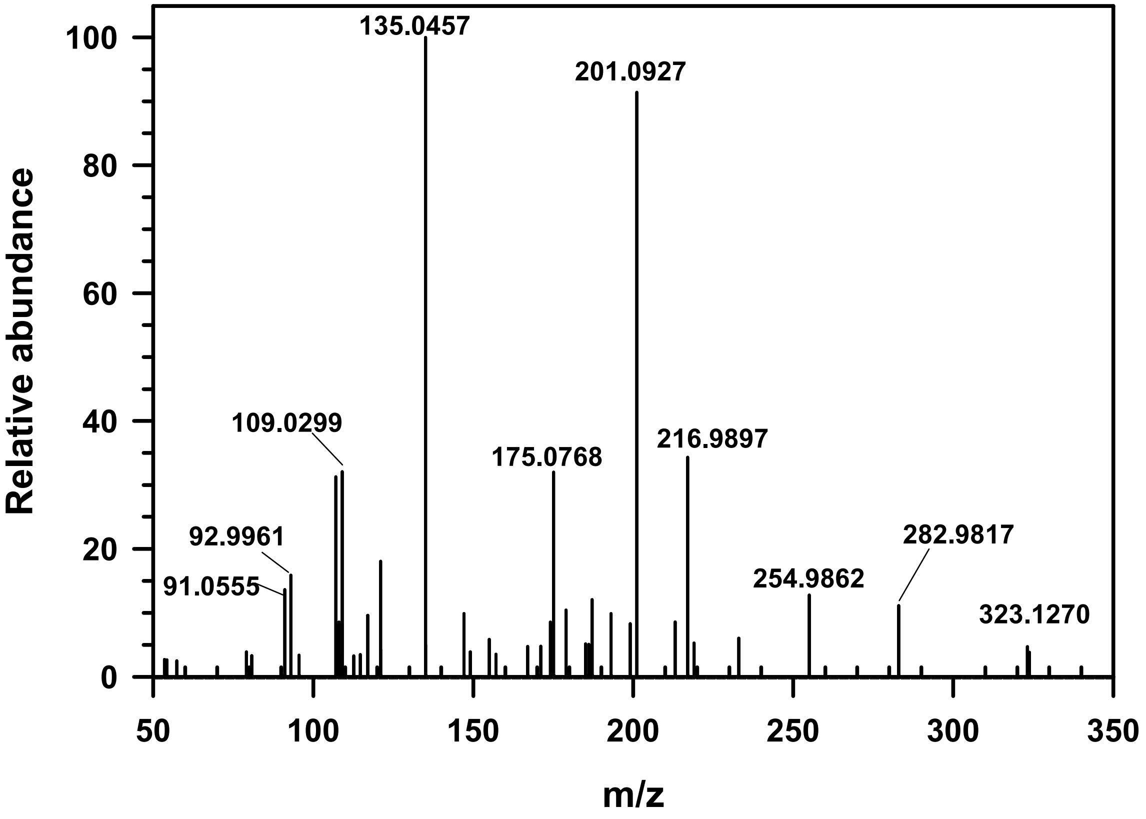

Supplement: Supplementary file 1 [file metabolites-11-00433-s001.zip › Cox et al Supplemental Information june 28.pdf]
